# Supplementary figures and images for: Clathrin-independent endocytosis and retrograde transport in cancer cells tune immune synapse organization and CD8 T cell response (part 1 of 2)
Source: eLife. 2026 Apr 22;14:RP105821. doi: 10.7554/eLife.105821 (PMC13102394; doi:10.7554/eLife.105821)

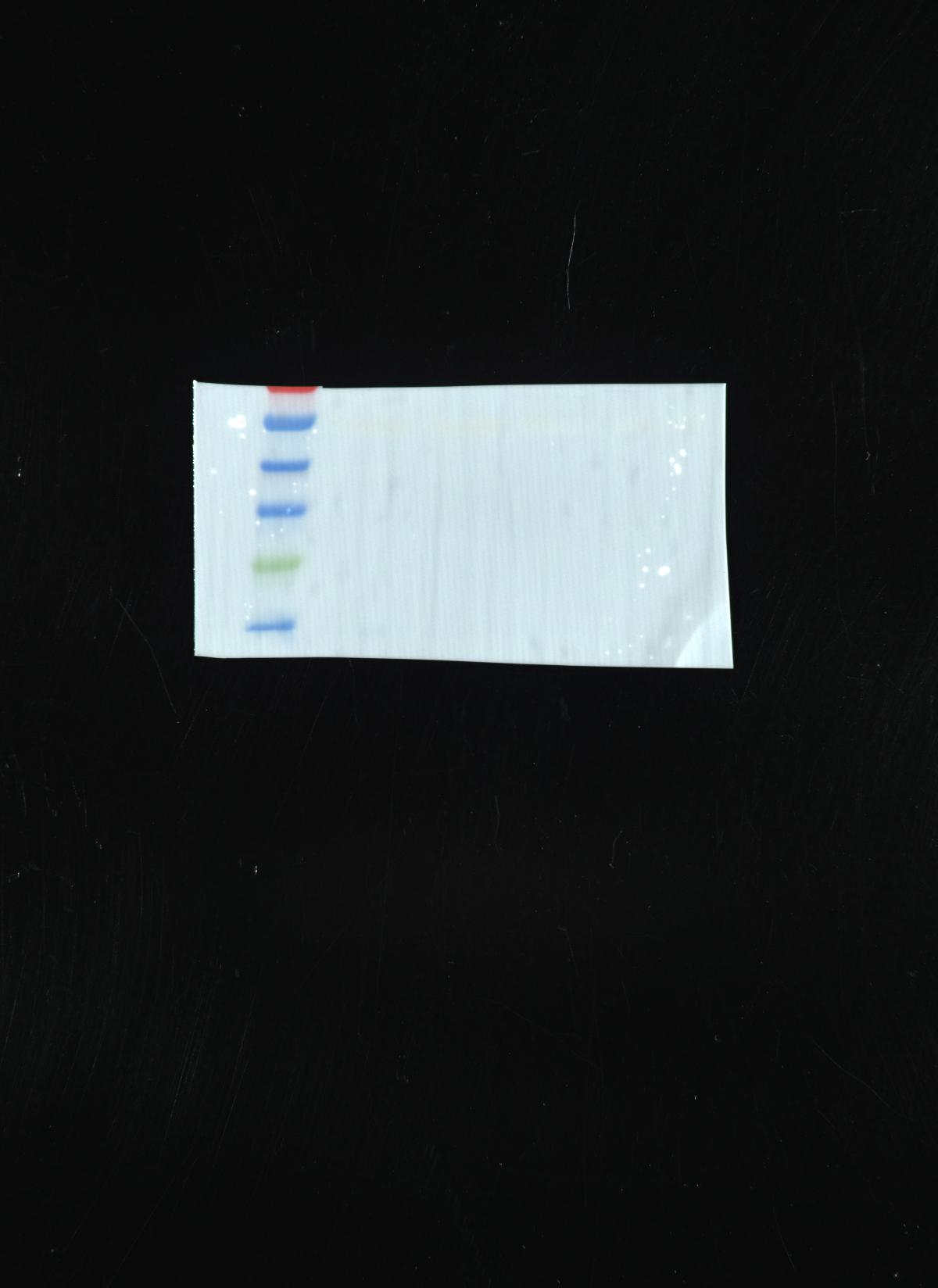

Supplement: Figure 1—source data 1. [file elife-105821-fig1-data1.zip › Figure 1-source data 1/Original files for western blot analysis displayed in Figure 1C/Tubulin_VPS26 HeLa_SNAP 20230329_125734_Ch/Tubulin_VPS26 HeLa_SNAP 20230329_125734_Ch-Marker.jpg]

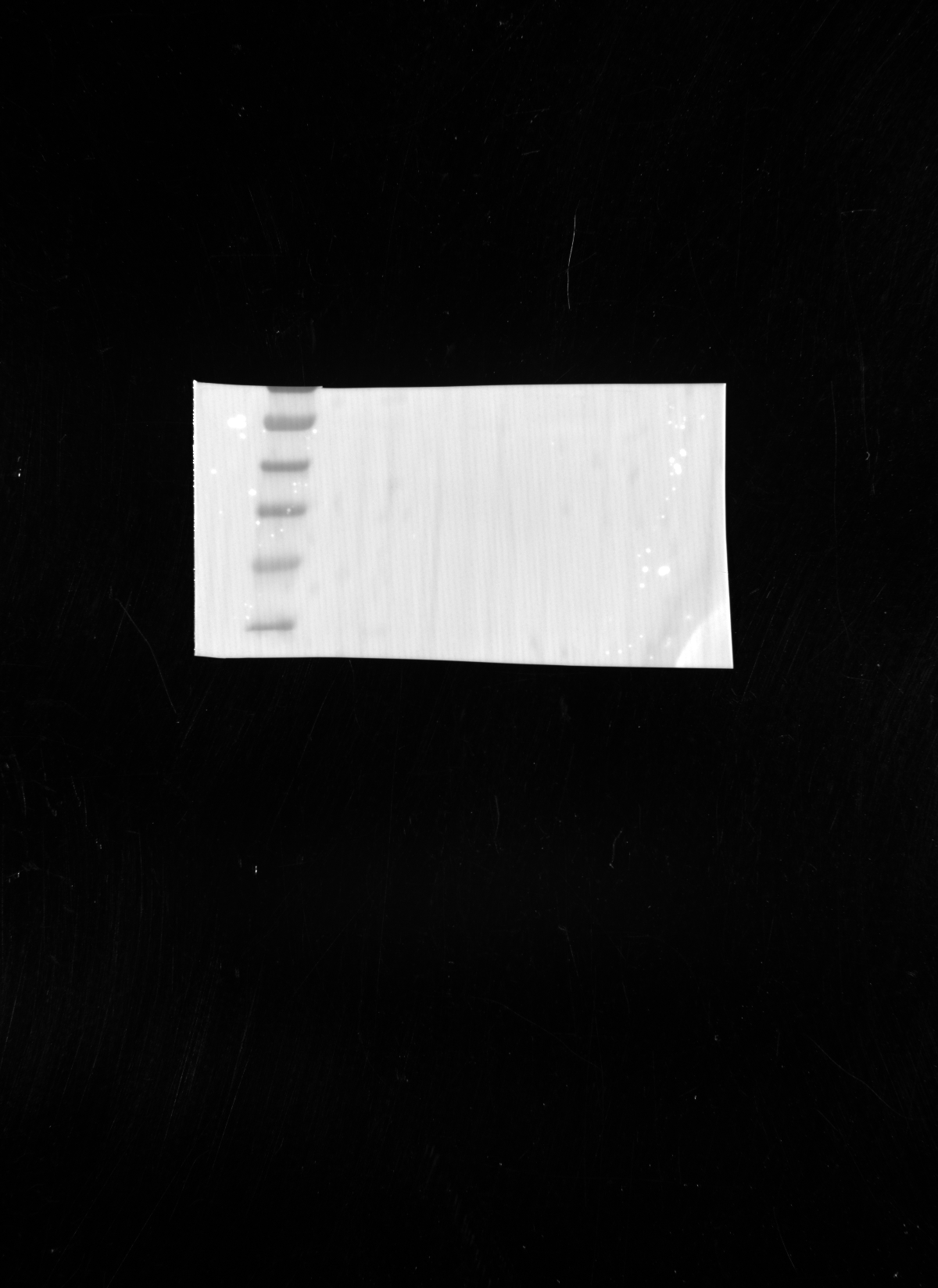

Supplement: Figure 1—source data 1. [file elife-105821-fig1-data1.zip › Figure 1-source data 1/Original files for western blot analysis displayed in Figure 1C/Tubulin_VPS26 HeLa_SNAP 20230329_125734_Ch/Tubulin_VPS26 HeLa_SNAP 20230329_125734_Ch-Marker.tif]

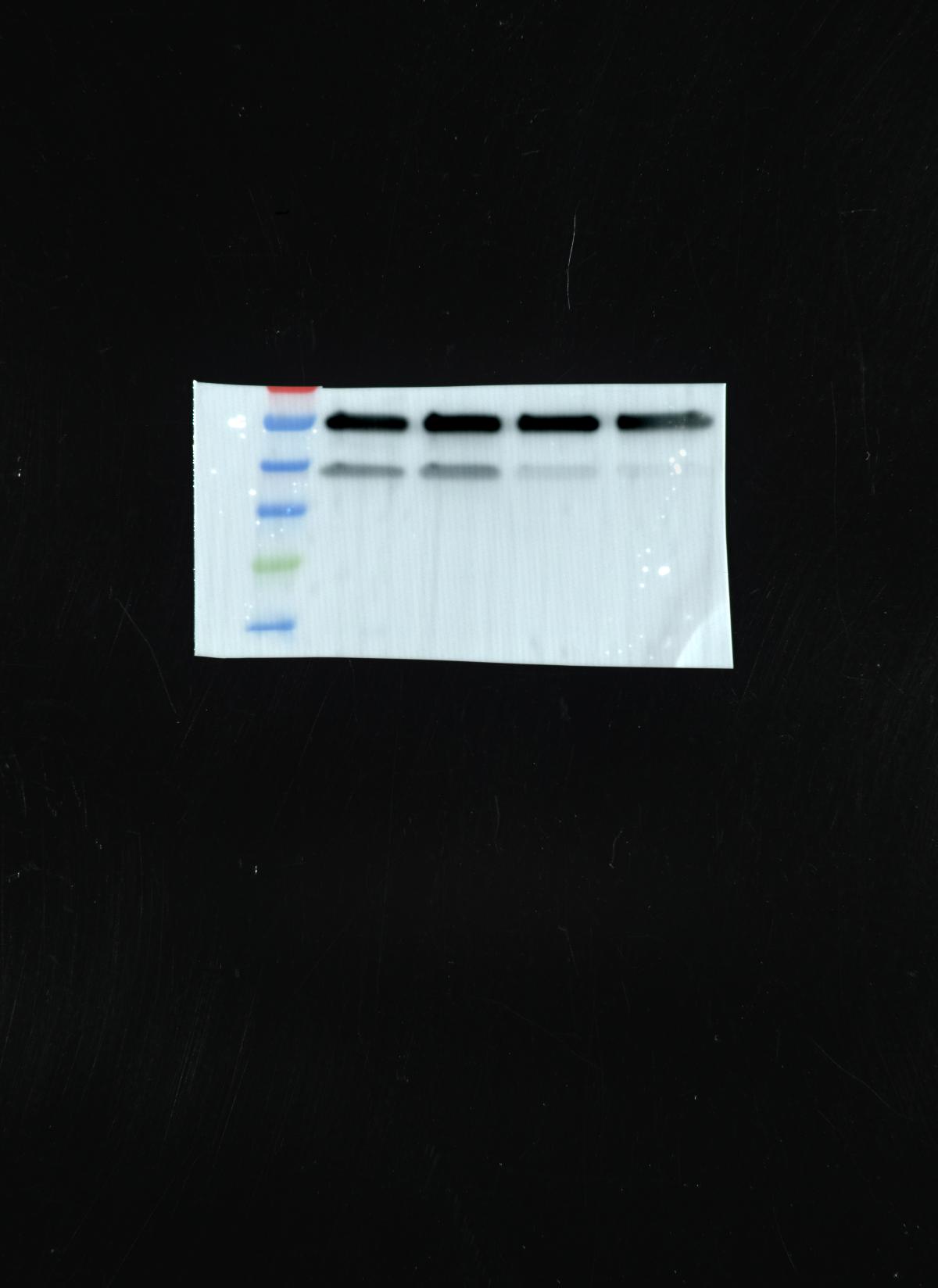

Supplement: Figure 1—source data 1. [file elife-105821-fig1-data1.zip › Figure 1-source data 1/Original files for western blot analysis displayed in Figure 1C/Tubulin_VPS26 HeLa_SNAP 20230329_125734_Ch/Tubulin_VPS26 HeLa_SNAP 20230329_125734_Ch_Chemi+Marker.jpg]

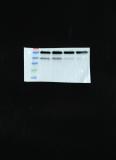

Supplement: Figure 1—source data 1. [file elife-105821-fig1-data1.zip › Figure 1-source data 1/Original files for western blot analysis displayed in Figure 1C/Tubulin_VPS26 HeLa_SNAP 20230329_125734_Ch/Tubulin_VPS26 HeLa_SNAP 20230329_125734_Ch_Thumb.jpg]

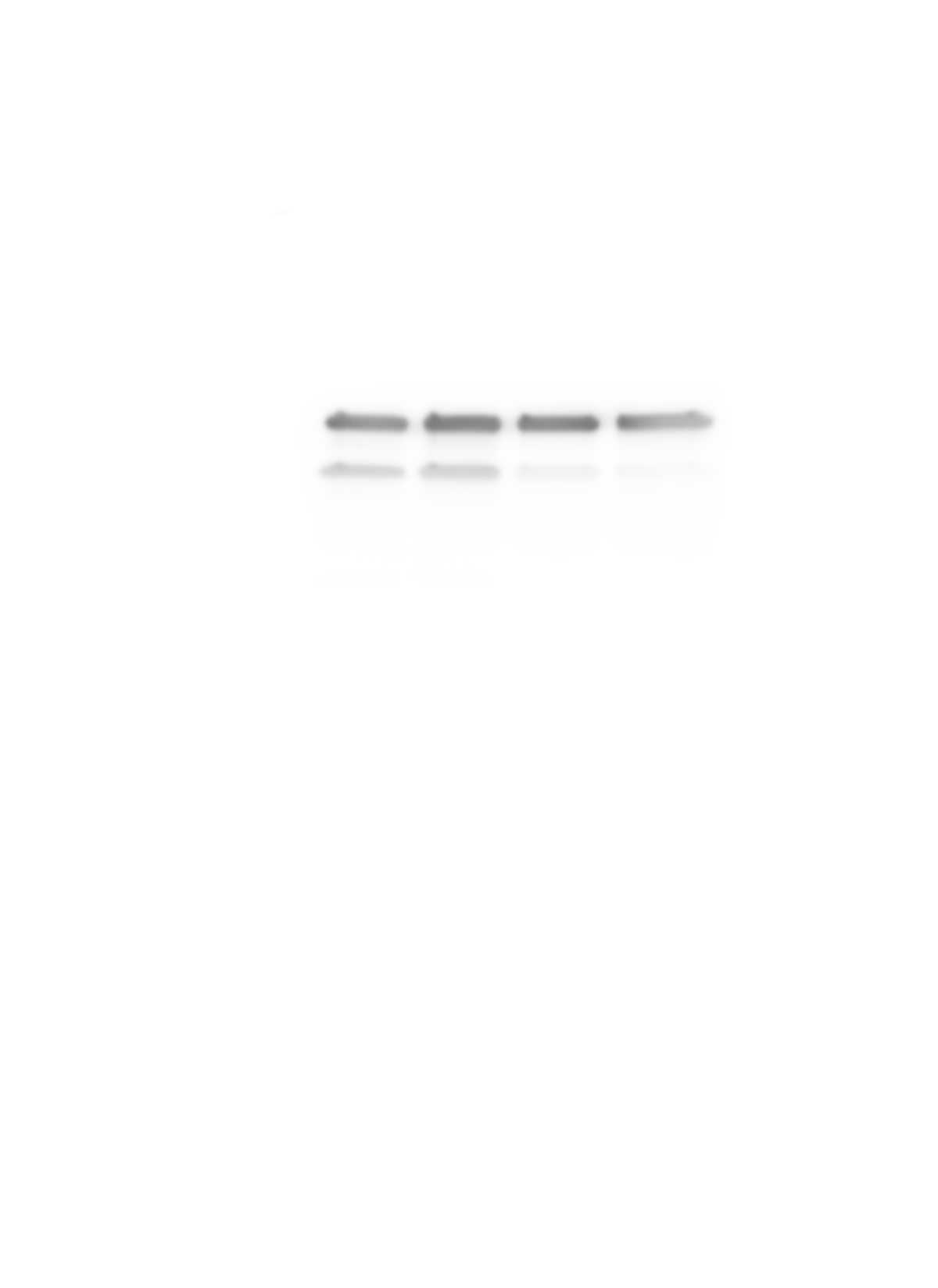

Supplement: Figure 1—source data 1. [file elife-105821-fig1-data1.zip › Figure 1-source data 1/Original files for western blot analysis displayed in Figure 1C/Tubulin_VPS26 HeLa_SNAP 20230329_125734_Ch/Tubulin_VPS26 HeLa_SNAP 20230329_125734_Ch_Chemi.tif]

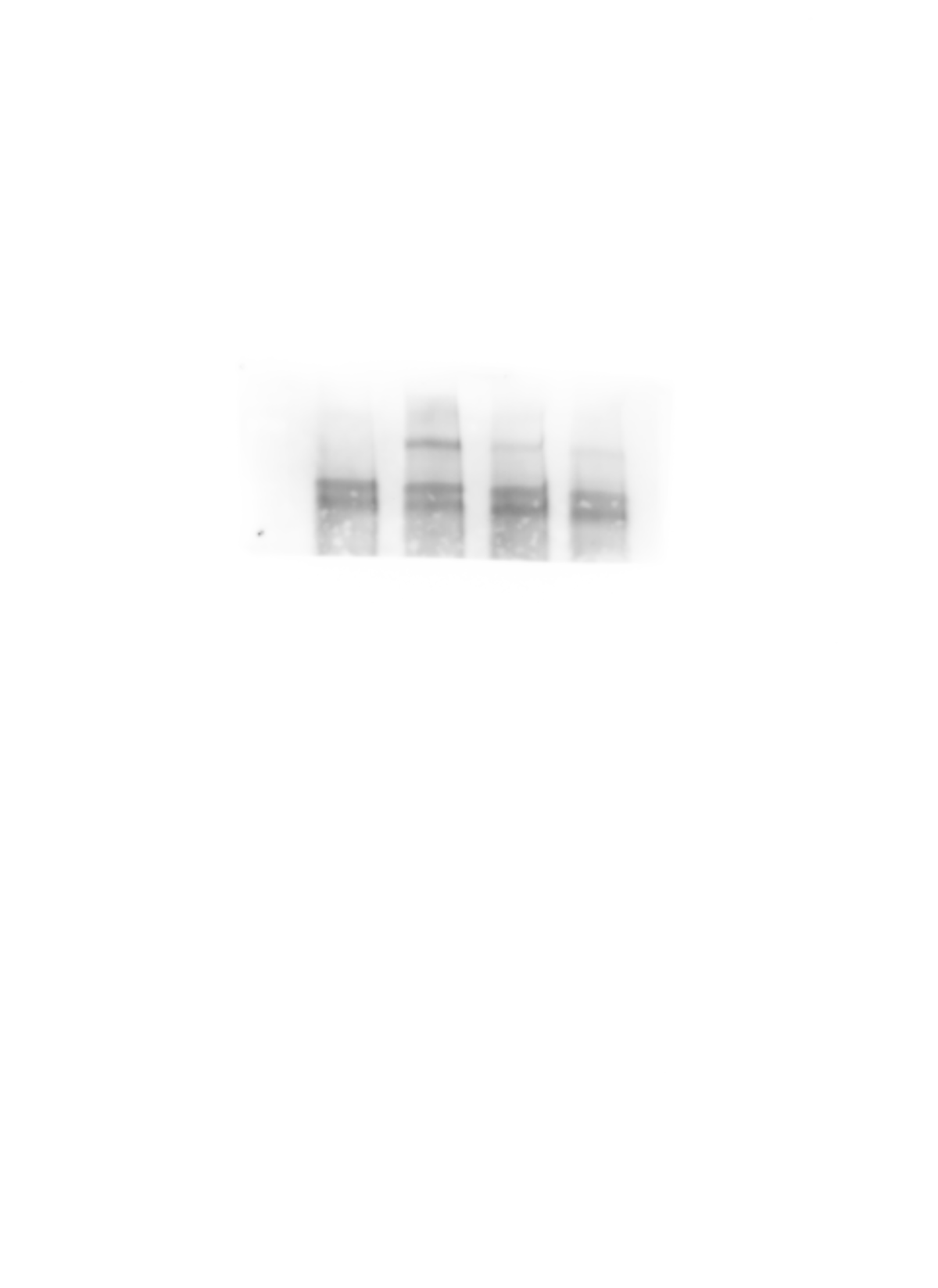

Supplement: Figure 1—source data 1. [file elife-105821-fig1-data1.zip › Figure 1-source data 1/Original files for western blot analysis displayed in Figure 1C/SNAP2 HeLa_SNAP 20230329_125009_Ch/SNAP2 HeLa_SNAP 20230329_125009_Ch_Chemi.tif]

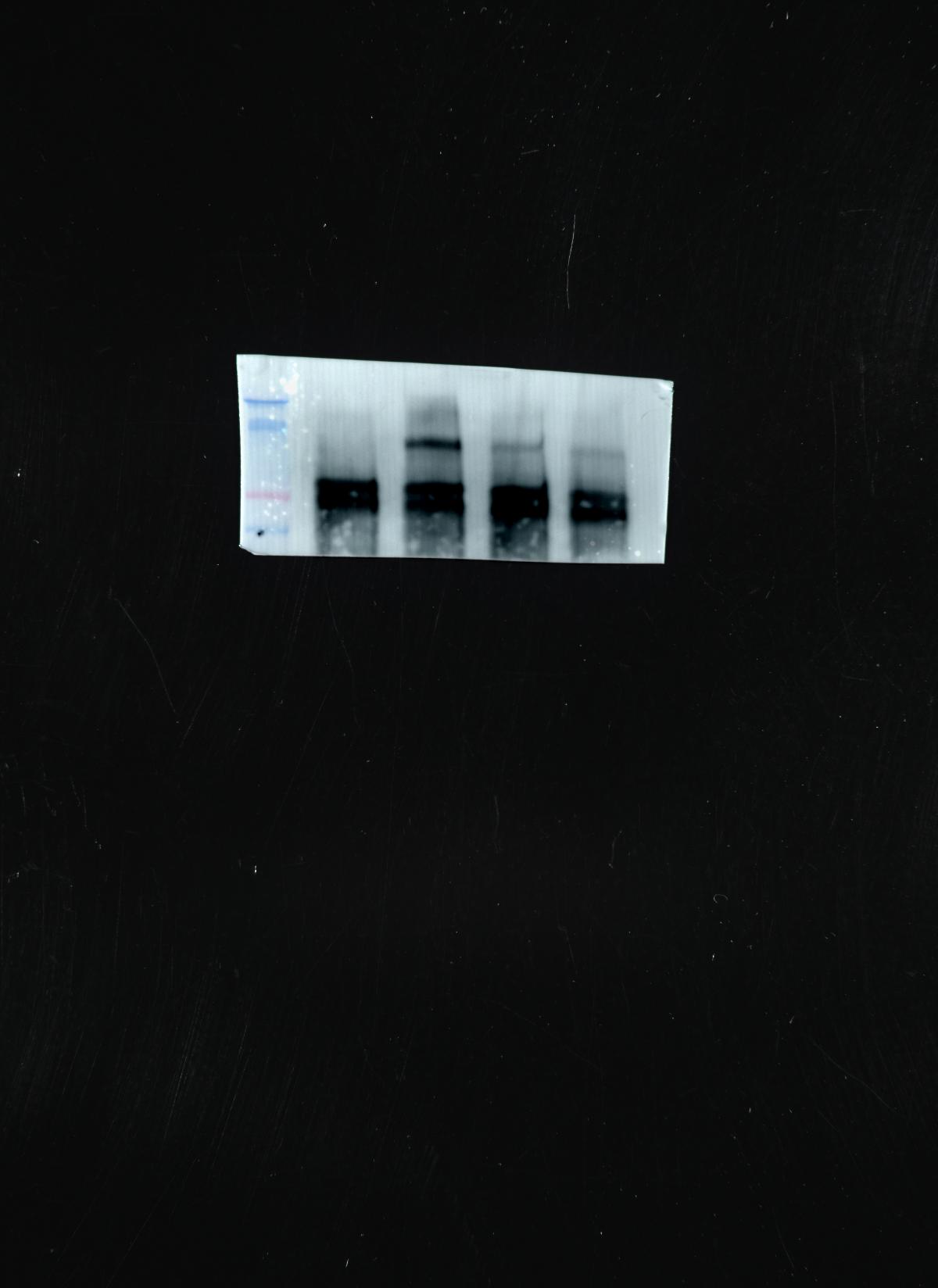

Supplement: Figure 1—source data 1. [file elife-105821-fig1-data1.zip › Figure 1-source data 1/Original files for western blot analysis displayed in Figure 1C/SNAP2 HeLa_SNAP 20230329_125009_Ch/SNAP2 HeLa_SNAP 20230329_125009_Ch_Chemi+Marker.jpg]

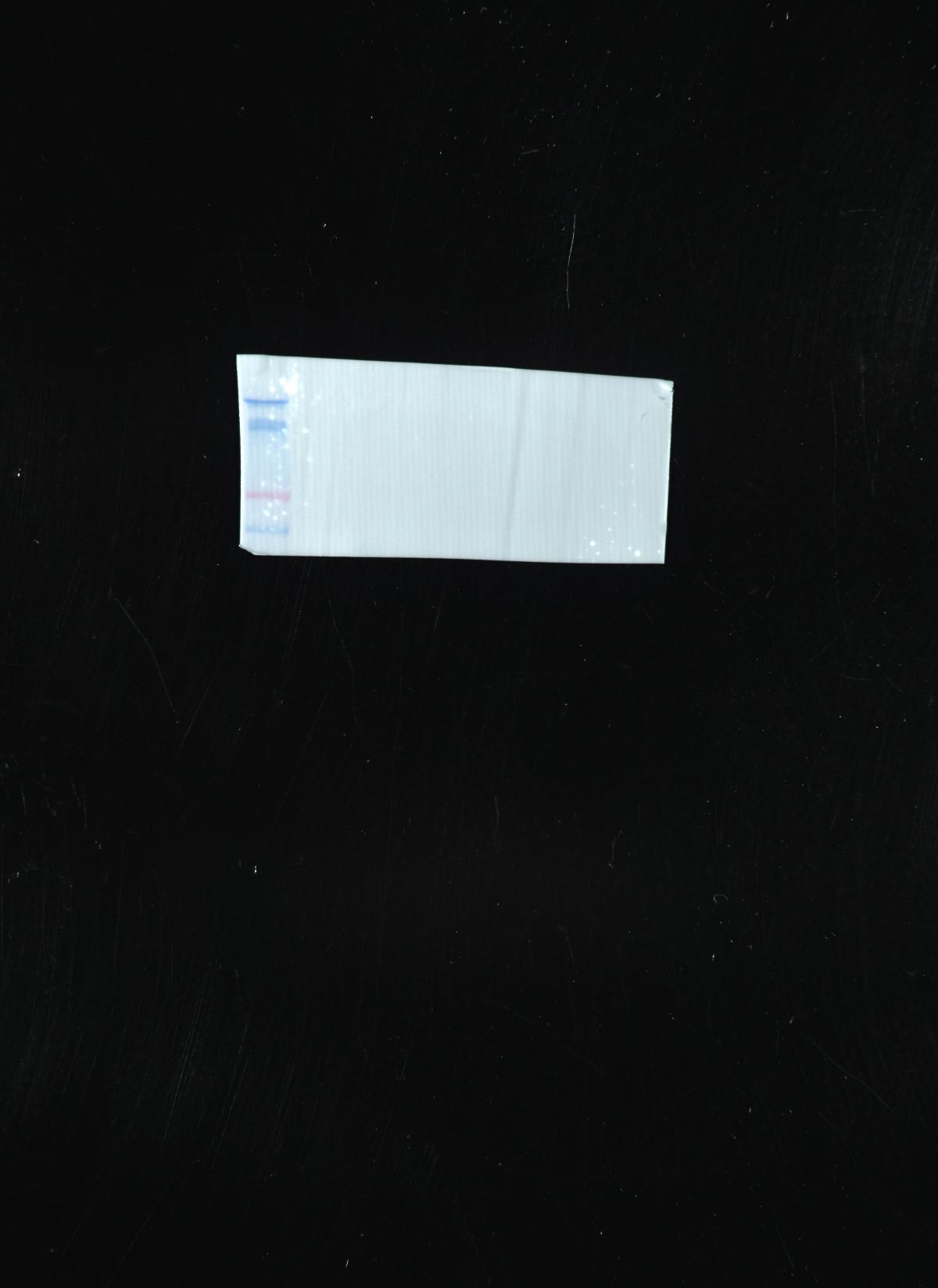

Supplement: Figure 1—source data 1. [file elife-105821-fig1-data1.zip › Figure 1-source data 1/Original files for western blot analysis displayed in Figure 1C/SNAP2 HeLa_SNAP 20230329_125009_Ch/SNAP2 HeLa_SNAP 20230329_125009_Ch-Marker.jpg]

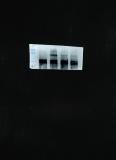

Supplement: Figure 1—source data 1. [file elife-105821-fig1-data1.zip › Figure 1-source data 1/Original files for western blot analysis displayed in Figure 1C/SNAP2 HeLa_SNAP 20230329_125009_Ch/SNAP2 HeLa_SNAP 20230329_125009_Ch_Thumb.jpg]

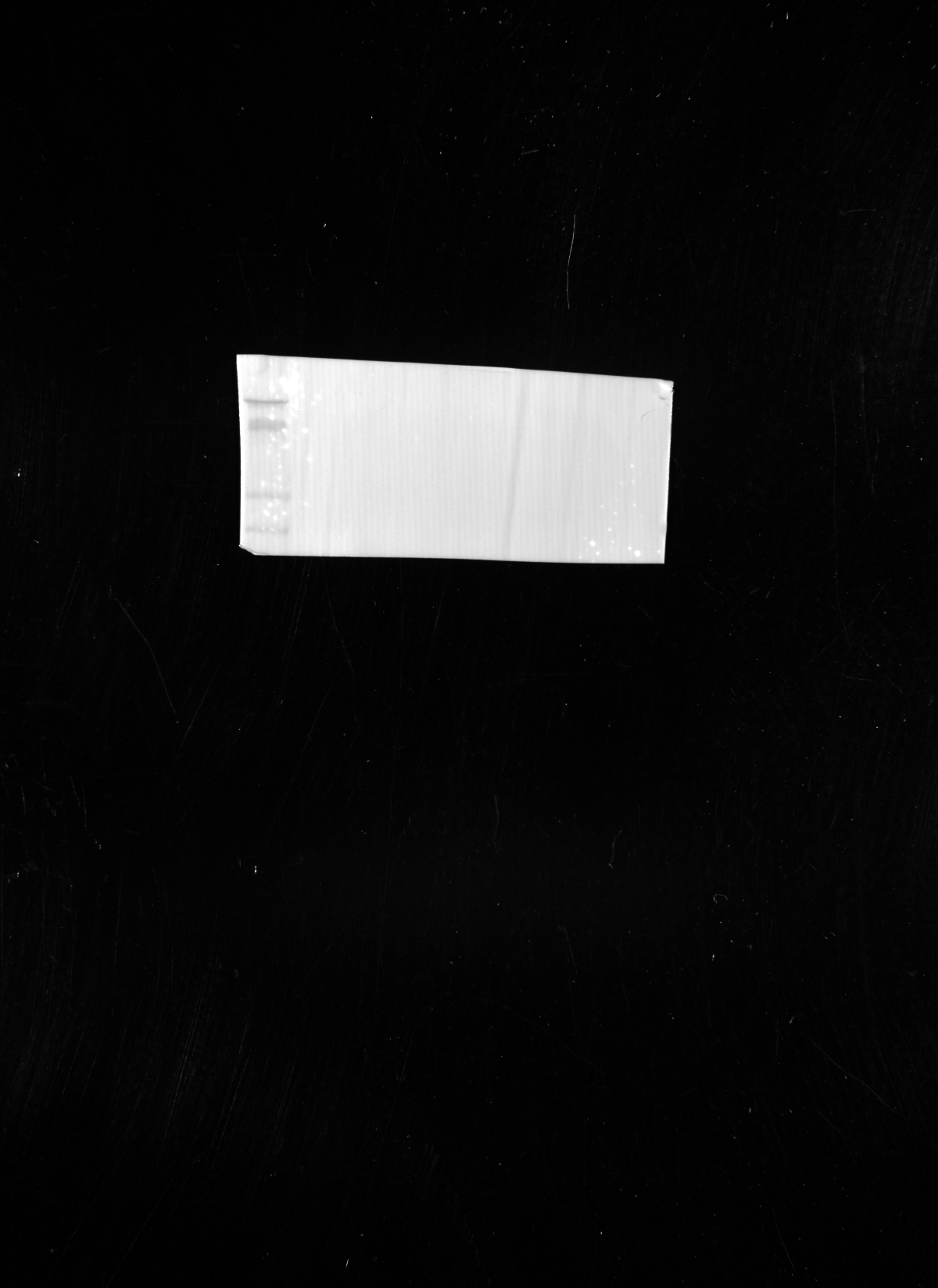

Supplement: Figure 1—source data 1. [file elife-105821-fig1-data1.zip › Figure 1-source data 1/Original files for western blot analysis displayed in Figure 1C/SNAP2 HeLa_SNAP 20230329_125009_Ch/SNAP2 HeLa_SNAP 20230329_125009_Ch-Marker.tif]

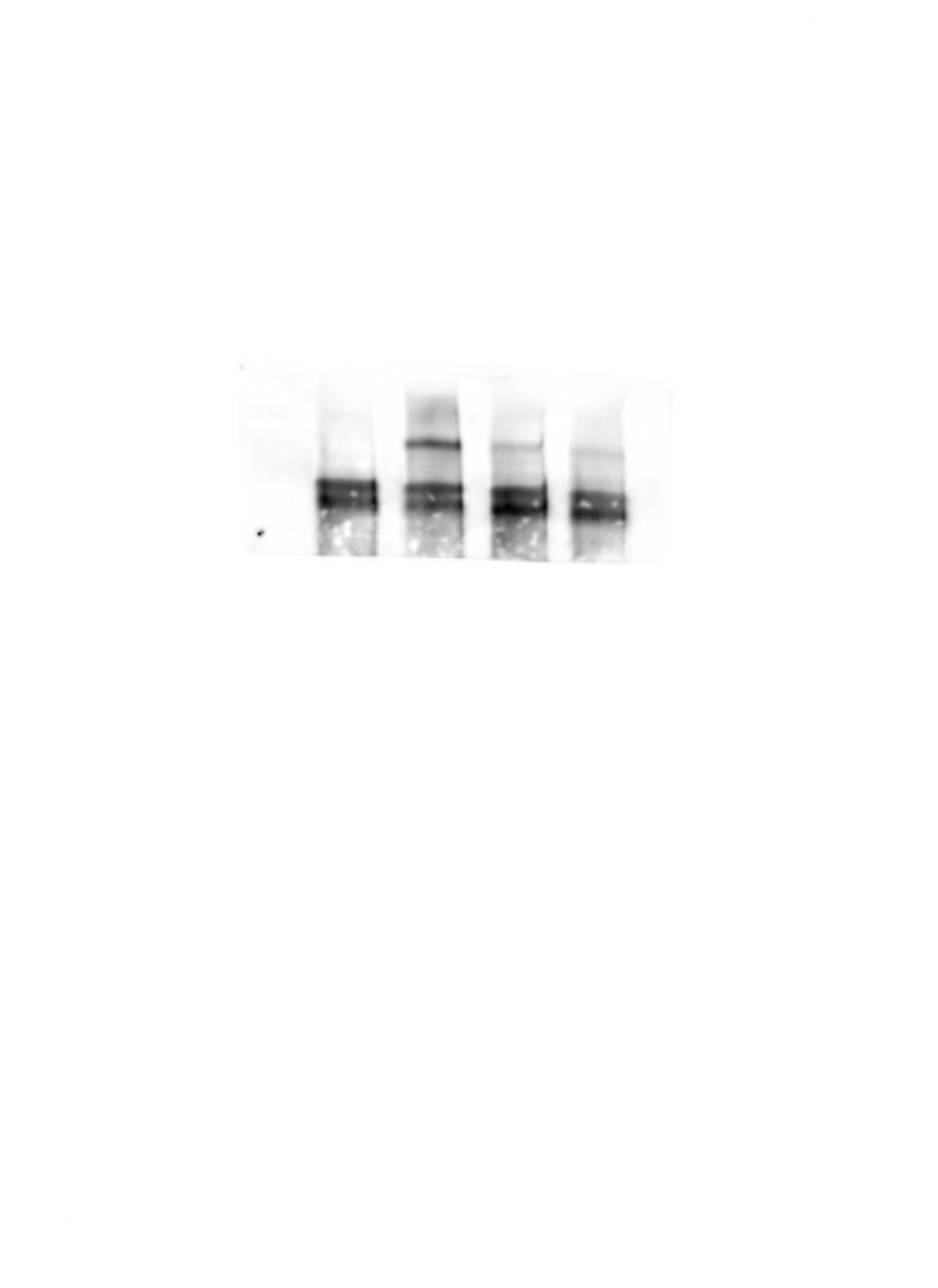

Supplement: Figure 1—source data 1. [file elife-105821-fig1-data1.zip › Figure 1-source data 1/Original files for western blot analysis displayed in Figure 1C/SNAP2 HeLa_SNAP 20230329_125009_Ch/SNAP2 HeLa_SNAP 20230329_125009_Ch_Chemi-1.tif]

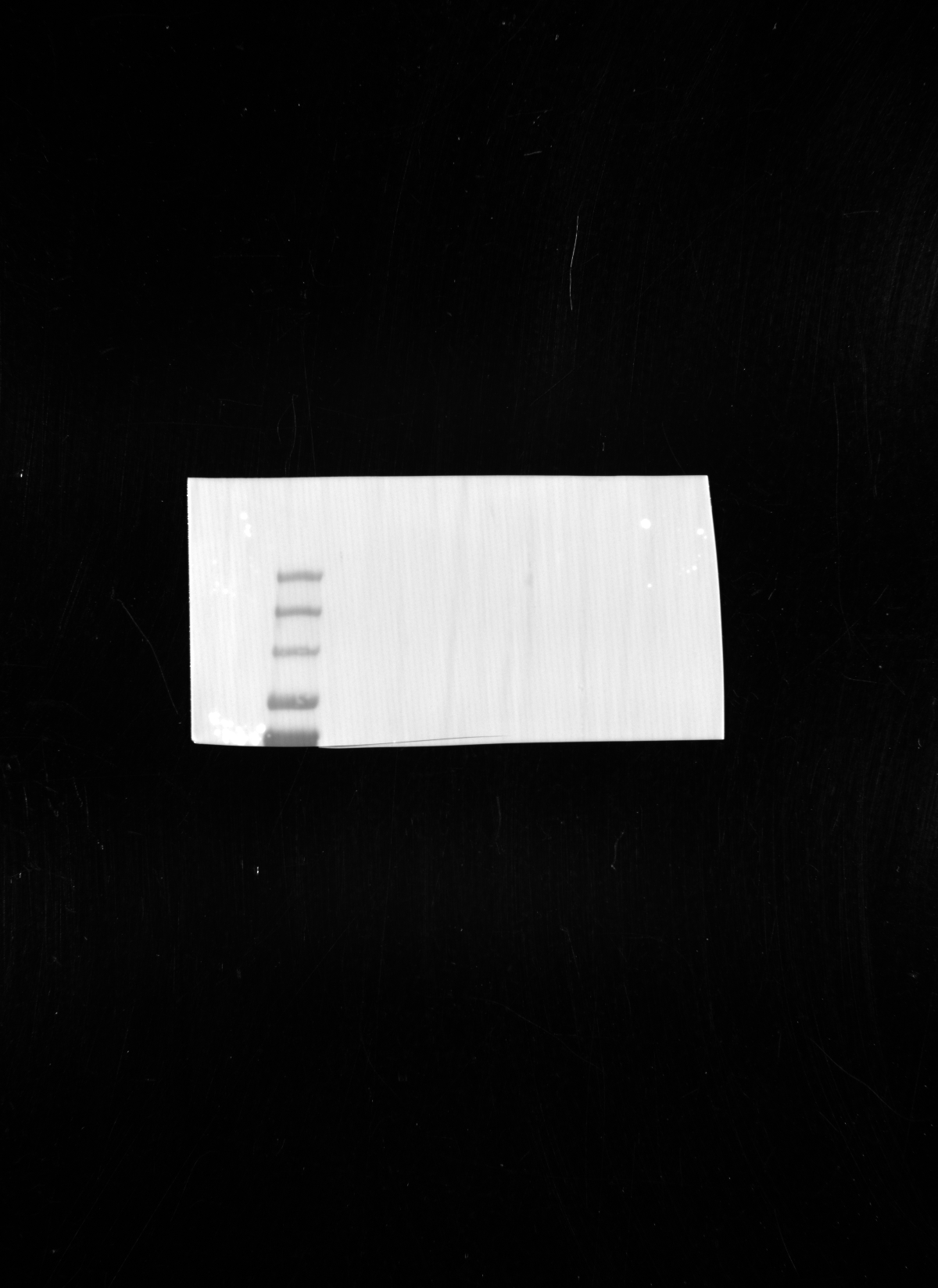

Supplement: Figure 1—source data 1. [file elife-105821-fig1-data1.zip › Figure 1-source data 1/Original files for western blot analysis displayed in Figure 1C/VPS35 HeLa_SNAP 20230329_124153_Ch/VPS35 HeLa_SNAP 20230329_124153_Ch-Marker.tif]

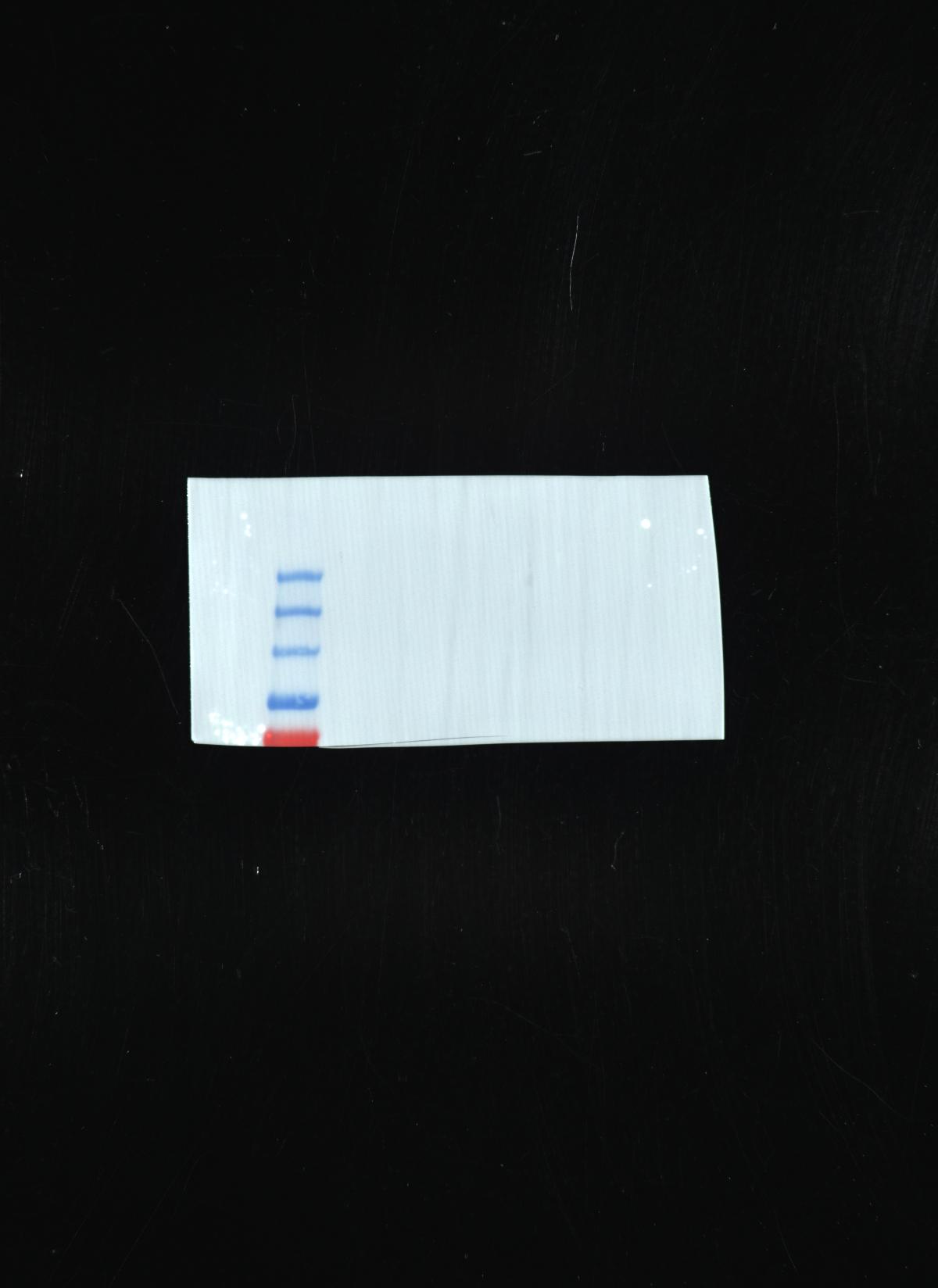

Supplement: Figure 1—source data 1. [file elife-105821-fig1-data1.zip › Figure 1-source data 1/Original files for western blot analysis displayed in Figure 1C/VPS35 HeLa_SNAP 20230329_124153_Ch/VPS35 HeLa_SNAP 20230329_124153_Ch-Marker.jpg]

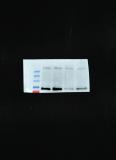

Supplement: Figure 1—source data 1. [file elife-105821-fig1-data1.zip › Figure 1-source data 1/Original files for western blot analysis displayed in Figure 1C/VPS35 HeLa_SNAP 20230329_124153_Ch/VPS35 HeLa_SNAP 20230329_124153_Ch_Thumb.jpg]

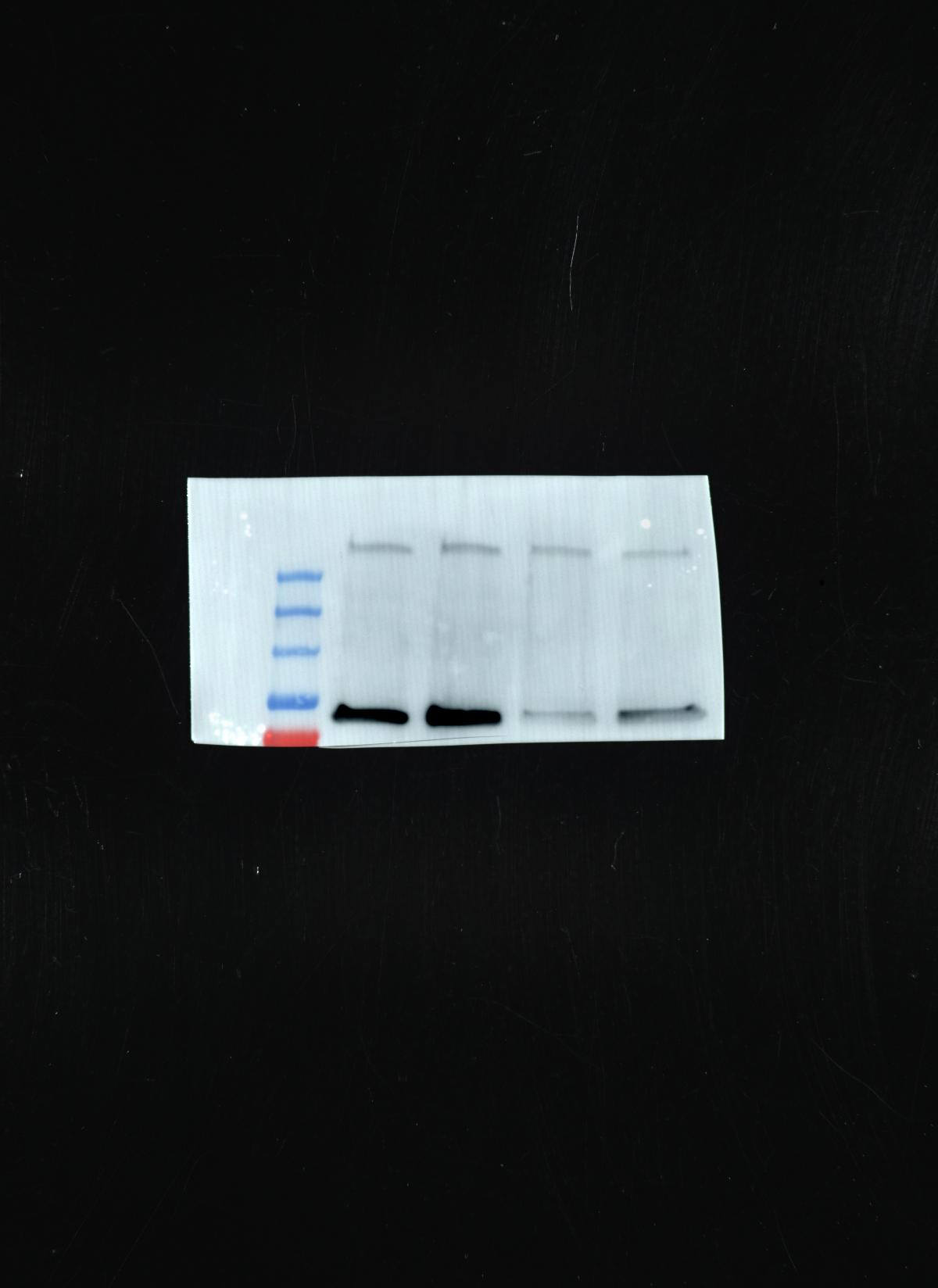

Supplement: Figure 1—source data 1. [file elife-105821-fig1-data1.zip › Figure 1-source data 1/Original files for western blot analysis displayed in Figure 1C/VPS35 HeLa_SNAP 20230329_124153_Ch/VPS35 HeLa_SNAP 20230329_124153_Ch_Chemi+Marker.jpg]

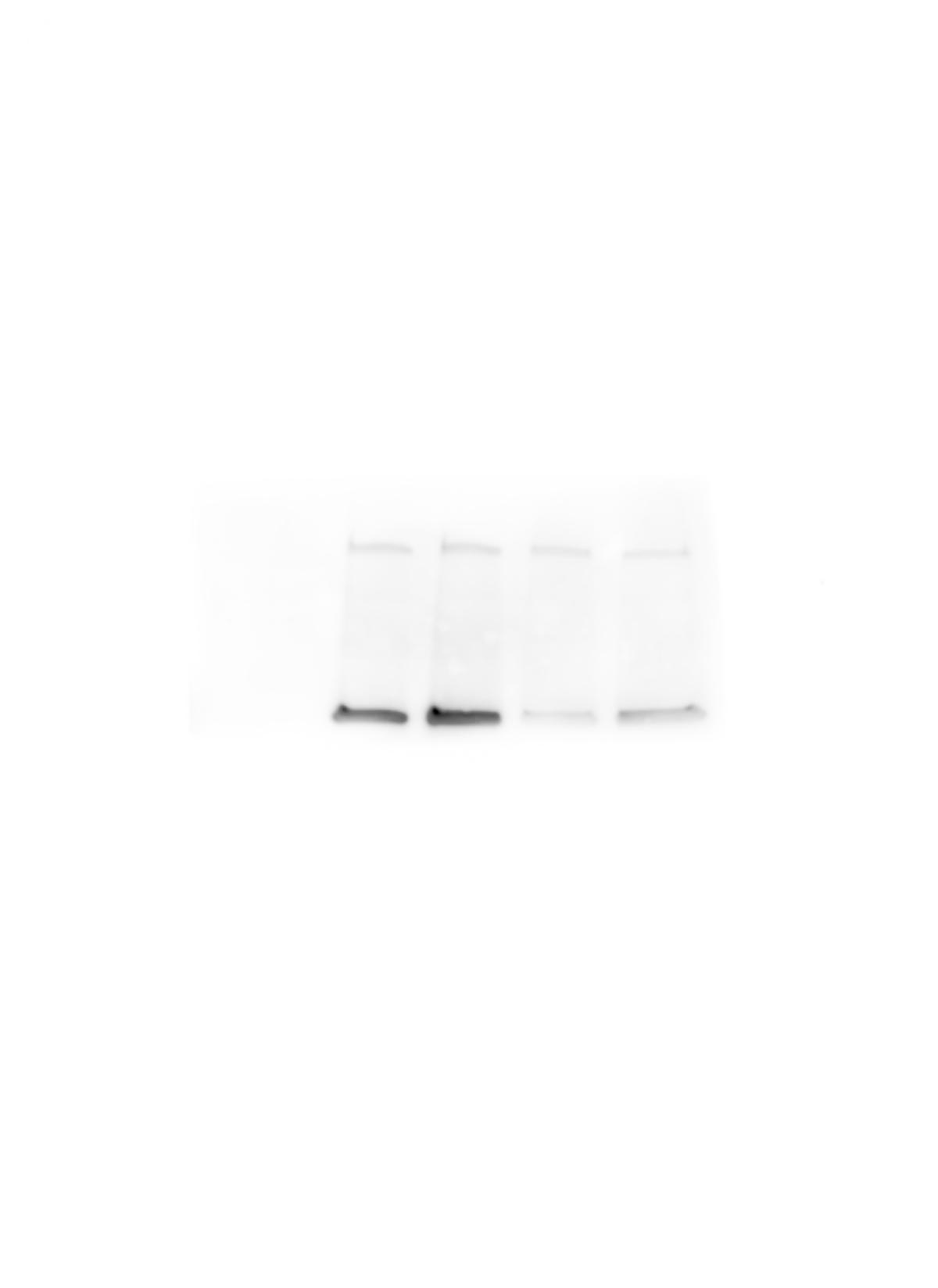

Supplement: Figure 1—source data 1. [file elife-105821-fig1-data1.zip › Figure 1-source data 1/Original files for western blot analysis displayed in Figure 1C/VPS35 HeLa_SNAP 20230329_124153_Ch/VPS35 HeLa_SNAP 20230329_124153_Ch_Chemi.tif]

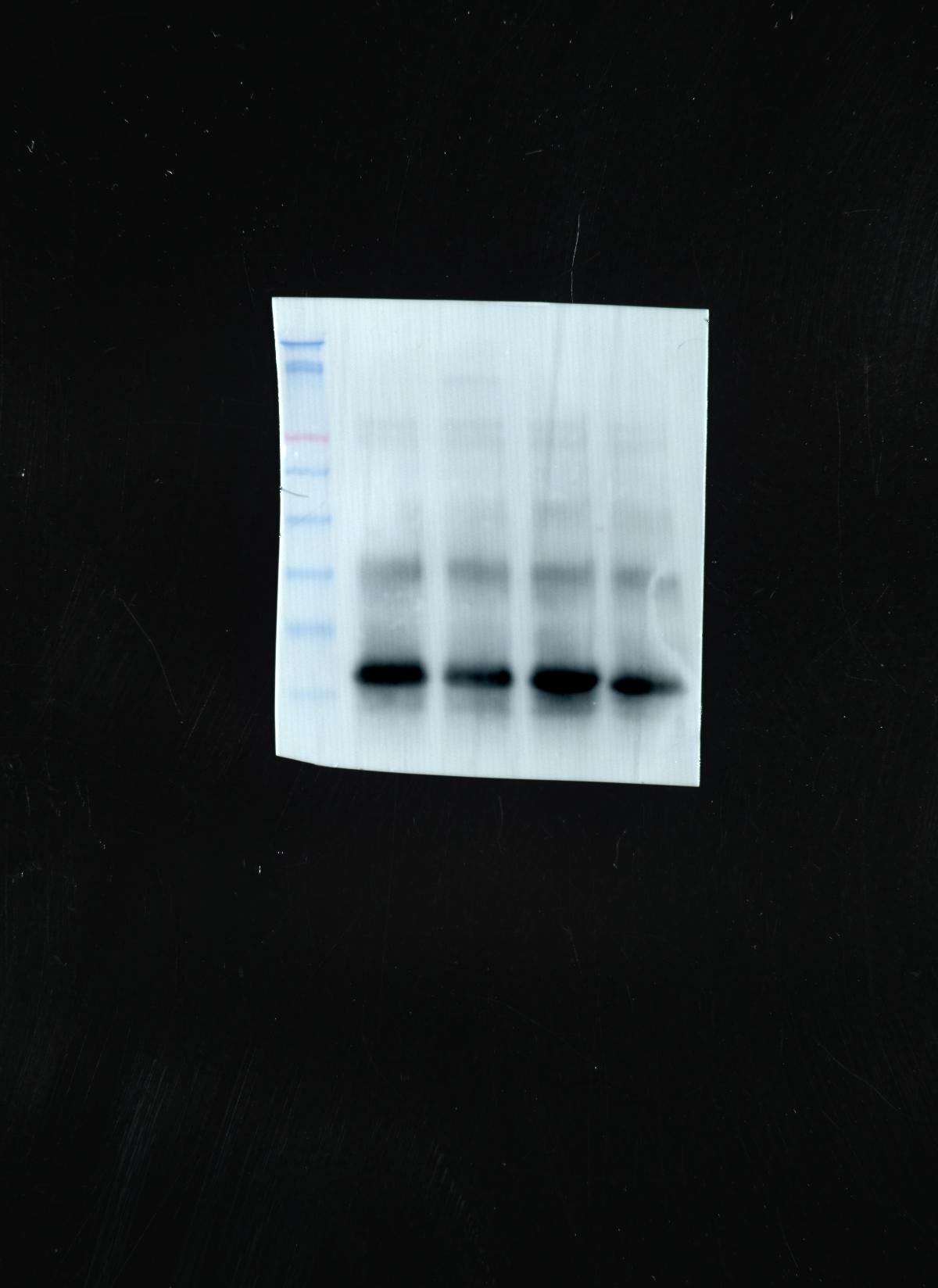

Supplement: Figure 1—source data 1. [file elife-105821-fig1-data1.zip › Figure 1-source data 1/Original files for western blot analysis displayed in Figure 1C/SNAP1 HeLa_SNAP 20230329_124655_Ch/SNAP1 HeLa_SNAP 20230329_124655_Ch_Chemi+Marker.jpg]

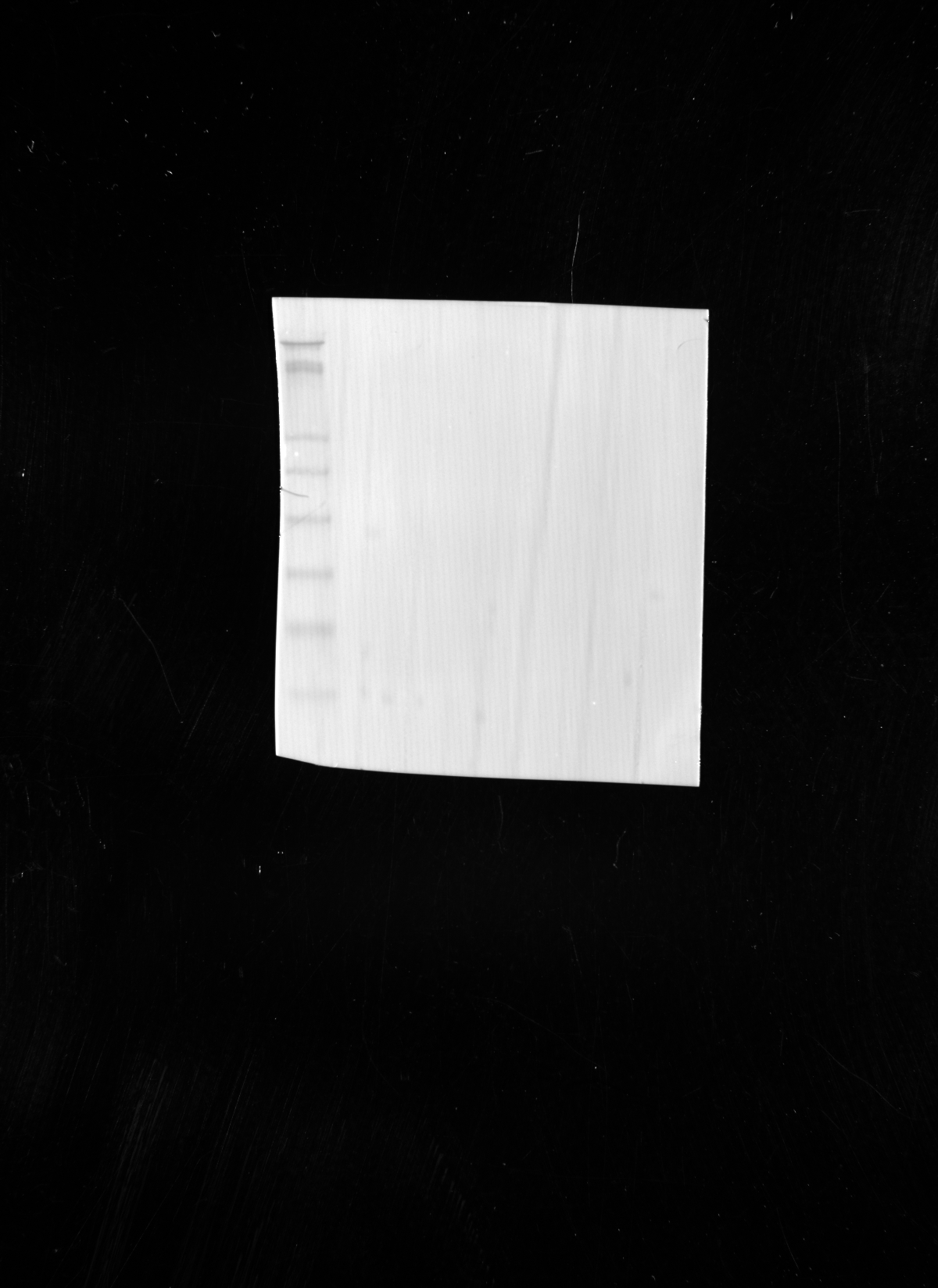

Supplement: Figure 1—source data 1. [file elife-105821-fig1-data1.zip › Figure 1-source data 1/Original files for western blot analysis displayed in Figure 1C/SNAP1 HeLa_SNAP 20230329_124655_Ch/SNAP1 HeLa_SNAP 20230329_124655_Ch-Marker.tif]

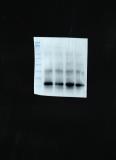

Supplement: Figure 1—source data 1. [file elife-105821-fig1-data1.zip › Figure 1-source data 1/Original files for western blot analysis displayed in Figure 1C/SNAP1 HeLa_SNAP 20230329_124655_Ch/SNAP1 HeLa_SNAP 20230329_124655_Ch_Thumb.jpg]

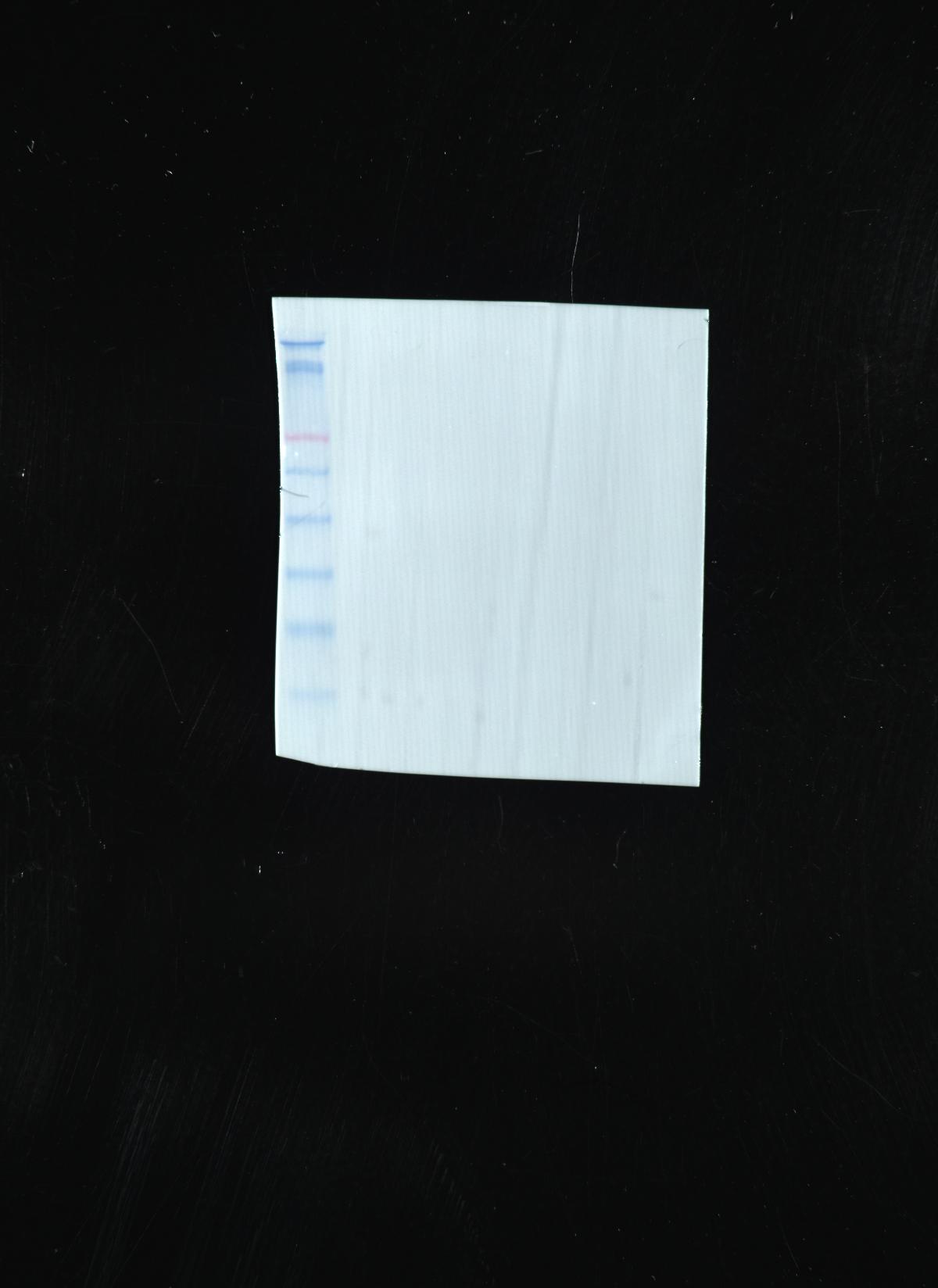

Supplement: Figure 1—source data 1. [file elife-105821-fig1-data1.zip › Figure 1-source data 1/Original files for western blot analysis displayed in Figure 1C/SNAP1 HeLa_SNAP 20230329_124655_Ch/SNAP1 HeLa_SNAP 20230329_124655_Ch-Marker.jpg]

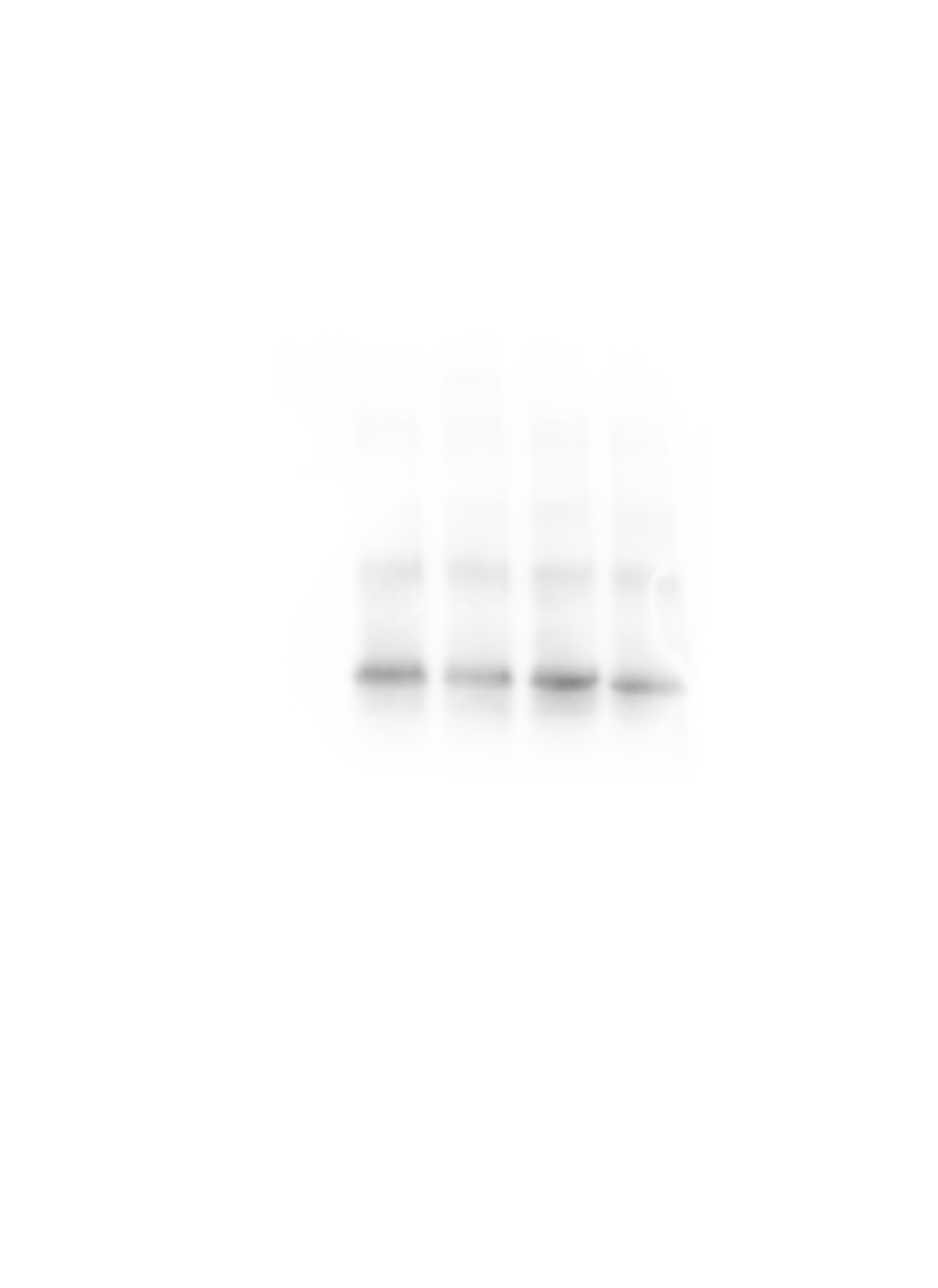

Supplement: Figure 1—source data 1. [file elife-105821-fig1-data1.zip › Figure 1-source data 1/Original files for western blot analysis displayed in Figure 1C/SNAP1 HeLa_SNAP 20230329_124655_Ch/SNAP1 HeLa_SNAP 20230329_124655_Ch_Chemi.tif]

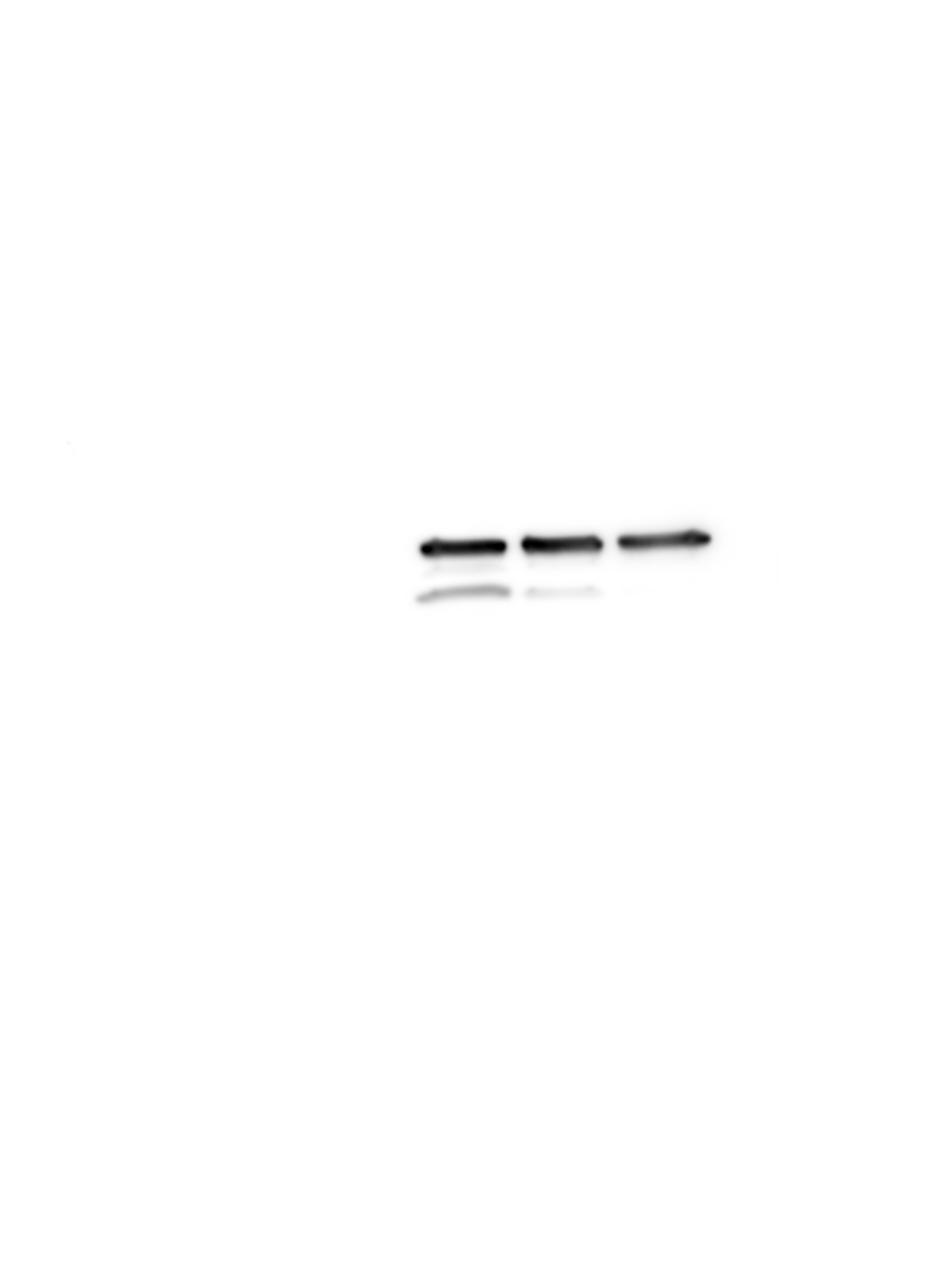

Supplement: Figure 1—source data 1. [file elife-105821-fig1-data1.zip › Figure 1-source data 1/Original files for western blot analysis displayed in Figure 1D/Tubulin Vps26 HeLa 20231122_115356_Ch/Tubulin Vps26 HeLa 20231122_115356_Ch_Chemi-2.tif]

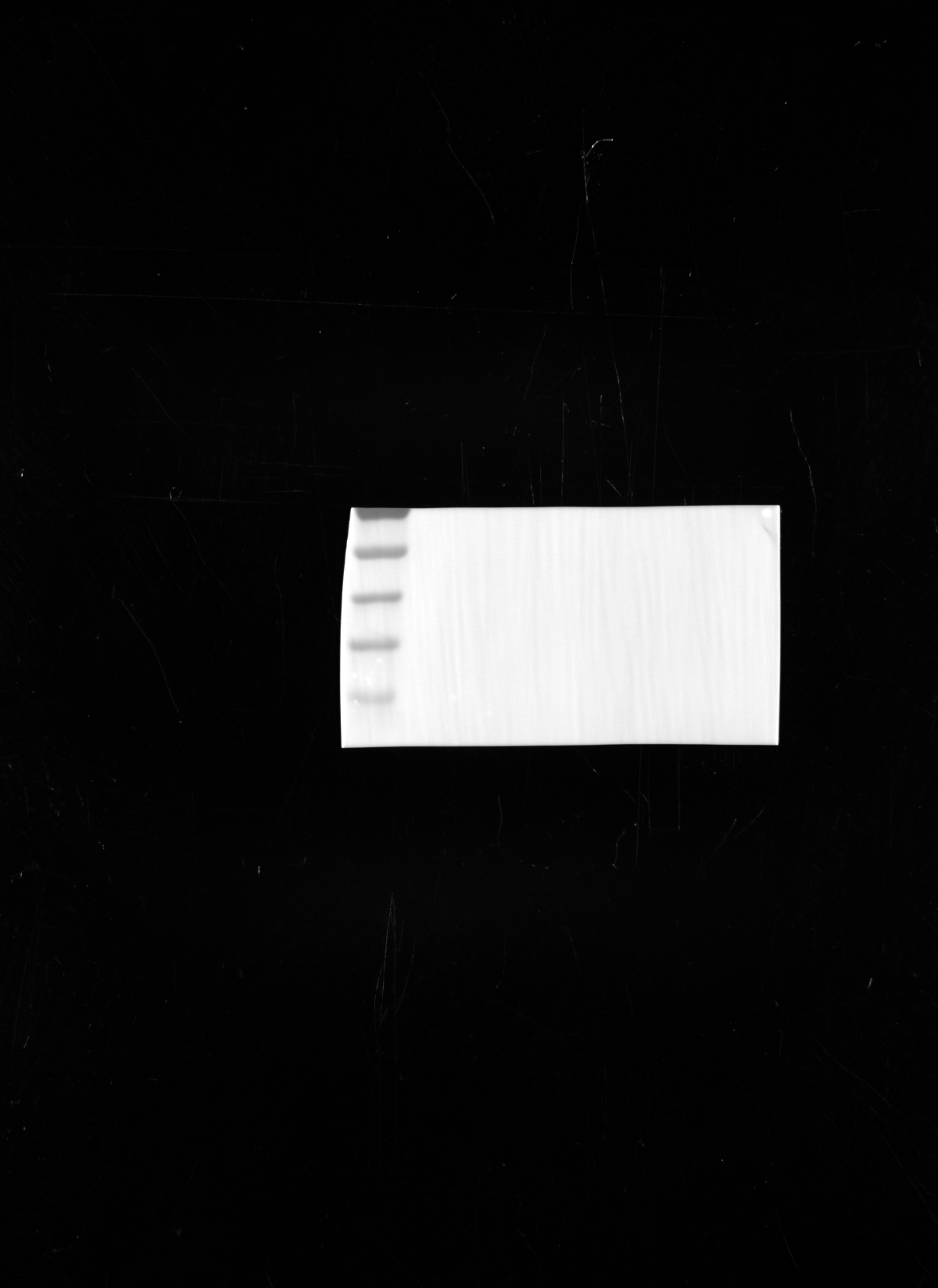

Supplement: Figure 1—source data 1. [file elife-105821-fig1-data1.zip › Figure 1-source data 1/Original files for western blot analysis displayed in Figure 1D/Tubulin Vps26 HeLa 20231122_115356_Ch/Tubulin Vps26 HeLa 20231122_115356_Ch-Marker.tif]

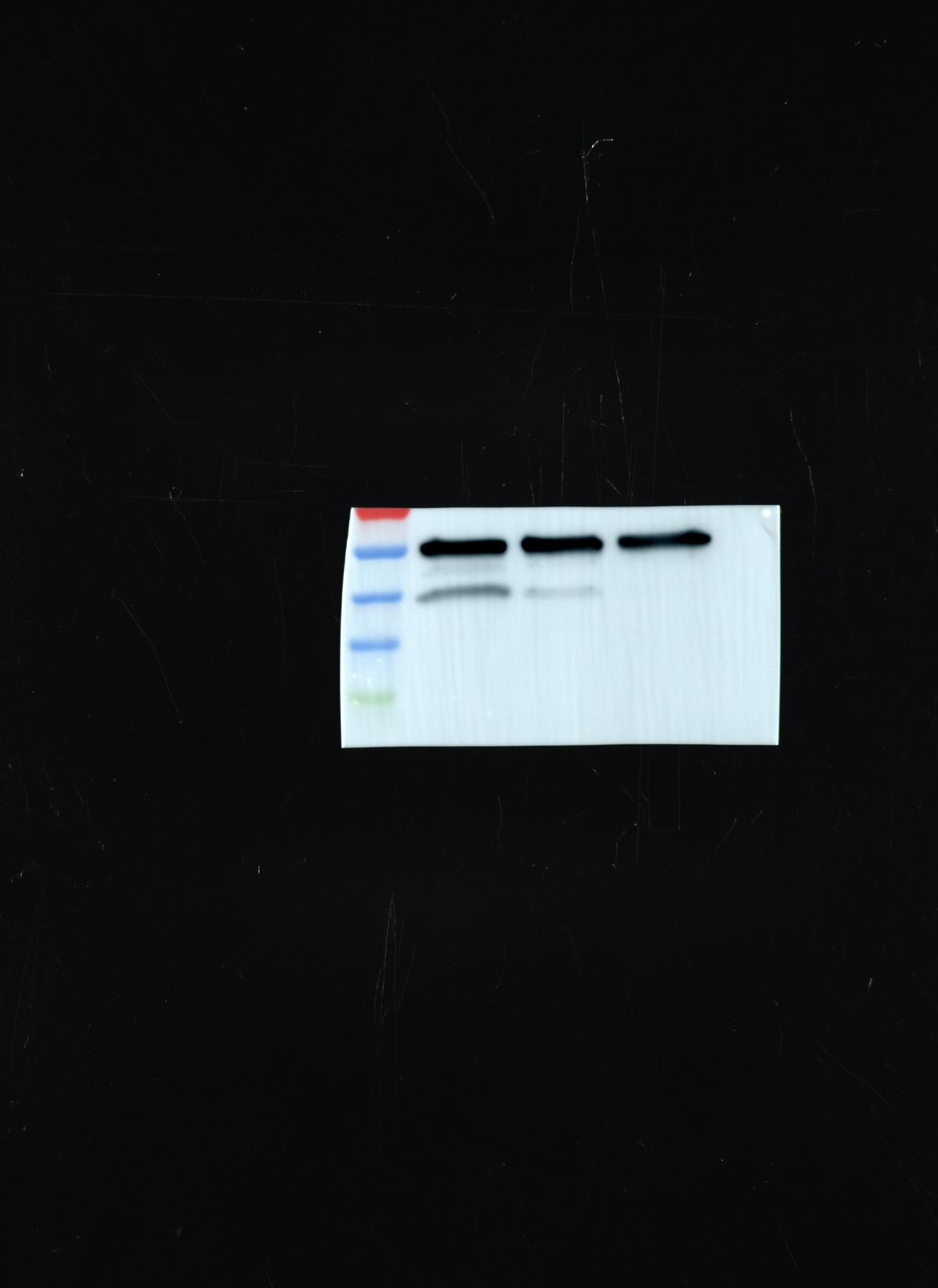

Supplement: Figure 1—source data 1. [file elife-105821-fig1-data1.zip › Figure 1-source data 1/Original files for western blot analysis displayed in Figure 1D/Tubulin Vps26 HeLa 20231122_115356_Ch/Tubulin Vps26 HeLa 20231122_115356_Ch_Chemi+Marker.jpg]

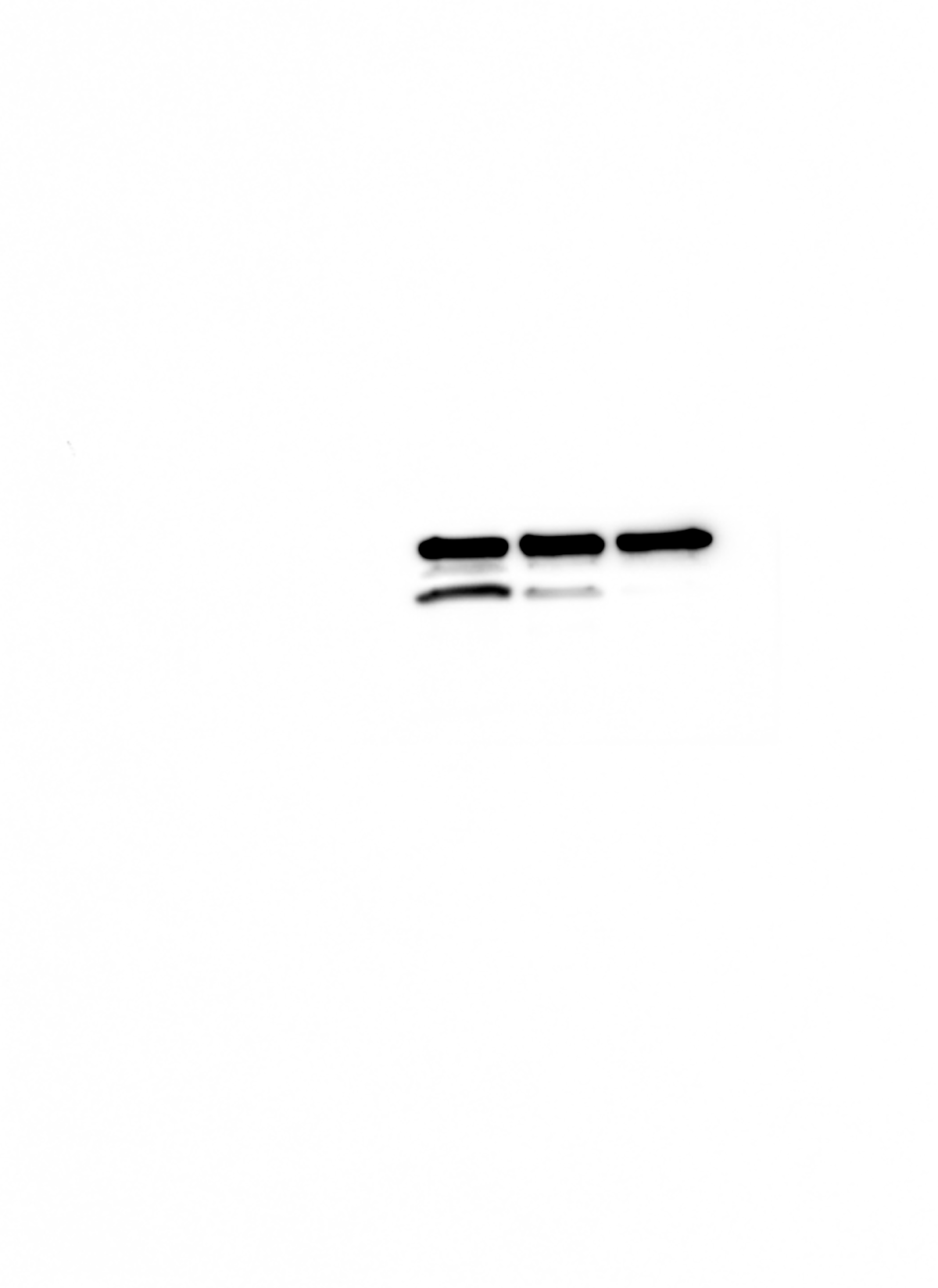

Supplement: Figure 1—source data 1. [file elife-105821-fig1-data1.zip › Figure 1-source data 1/Original files for western blot analysis displayed in Figure 1D/Tubulin Vps26 HeLa 20231122_115356_Ch/Vps26 HeLa 20231122_115356_Ch_Chemi-2.tif]

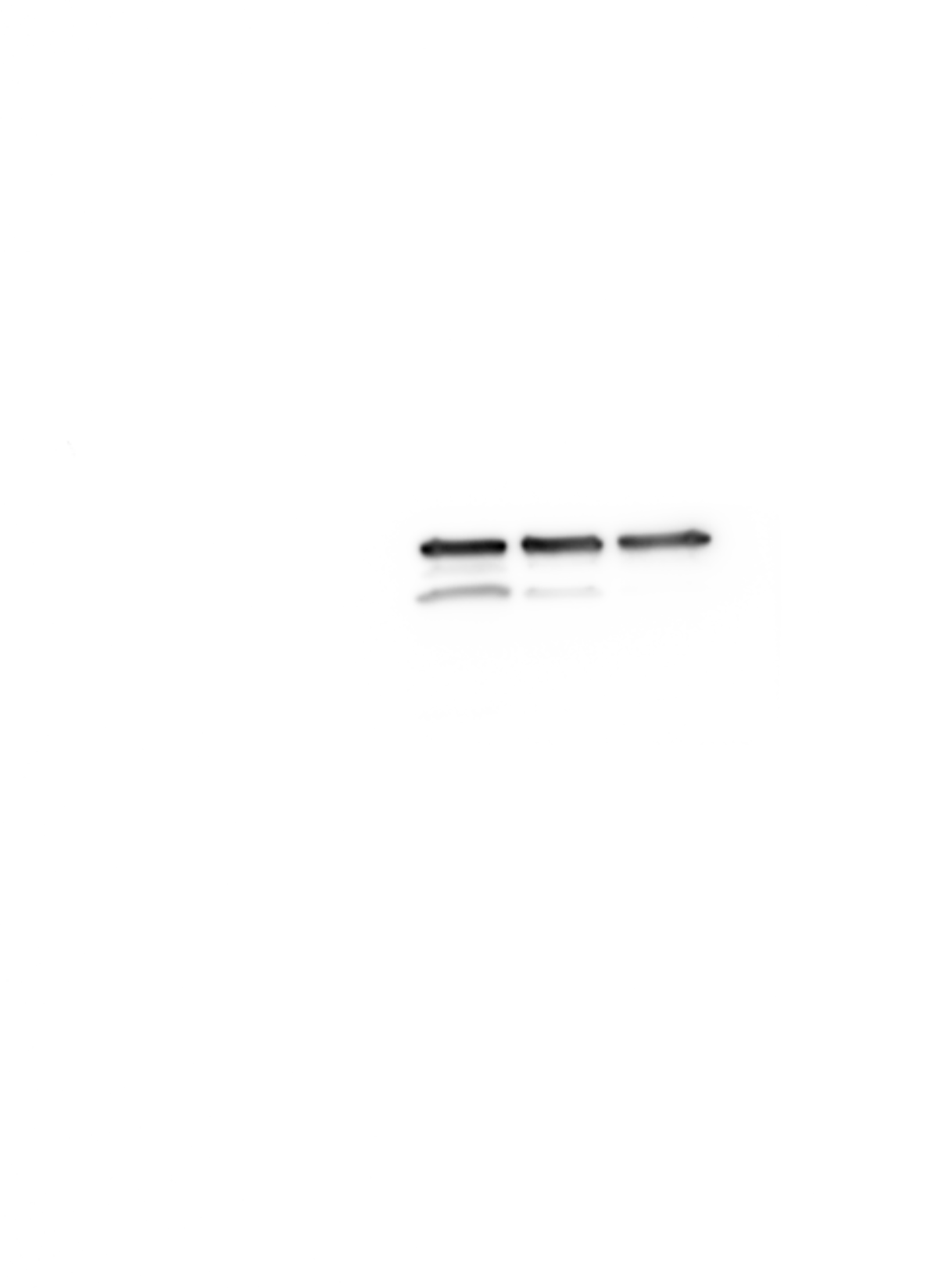

Supplement: Figure 1—source data 1. [file elife-105821-fig1-data1.zip › Figure 1-source data 1/Original files for western blot analysis displayed in Figure 1D/Tubulin Vps26 HeLa 20231122_115356_Ch/Tubulin Vps26 HeLa 20231122_115356_Ch_Chemi.tif]

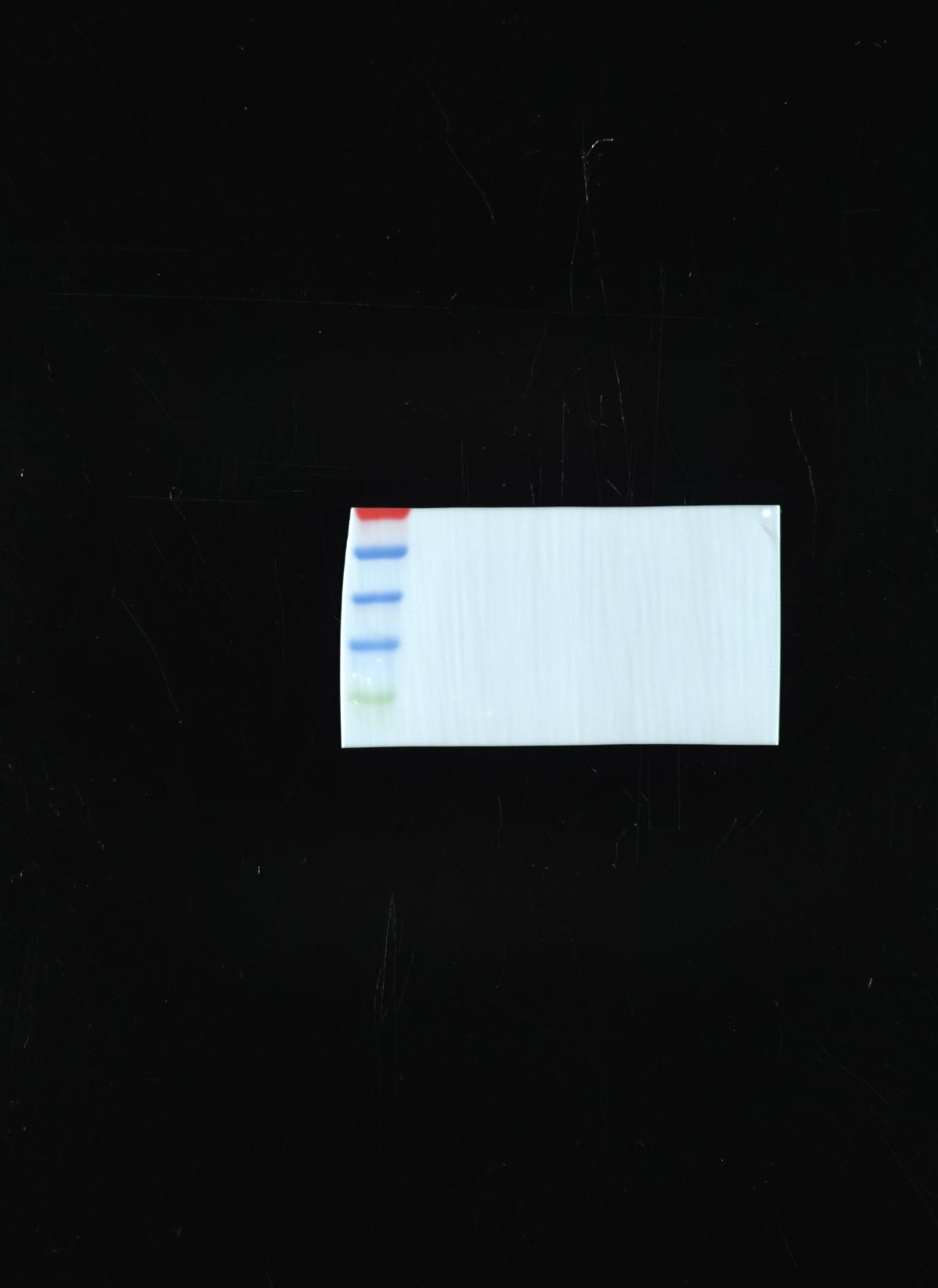

Supplement: Figure 1—source data 1. [file elife-105821-fig1-data1.zip › Figure 1-source data 1/Original files for western blot analysis displayed in Figure 1D/Tubulin Vps26 HeLa 20231122_115356_Ch/Tubulin Vps26 HeLa 20231122_115356_Ch-Marker.jpg]

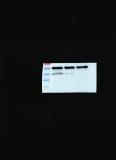

Supplement: Figure 1—source data 1. [file elife-105821-fig1-data1.zip › Figure 1-source data 1/Original files for western blot analysis displayed in Figure 1D/Tubulin Vps26 HeLa 20231122_115356_Ch/Tubulin Vps26 HeLa 20231122_115356_Ch_Thumb.jpg]

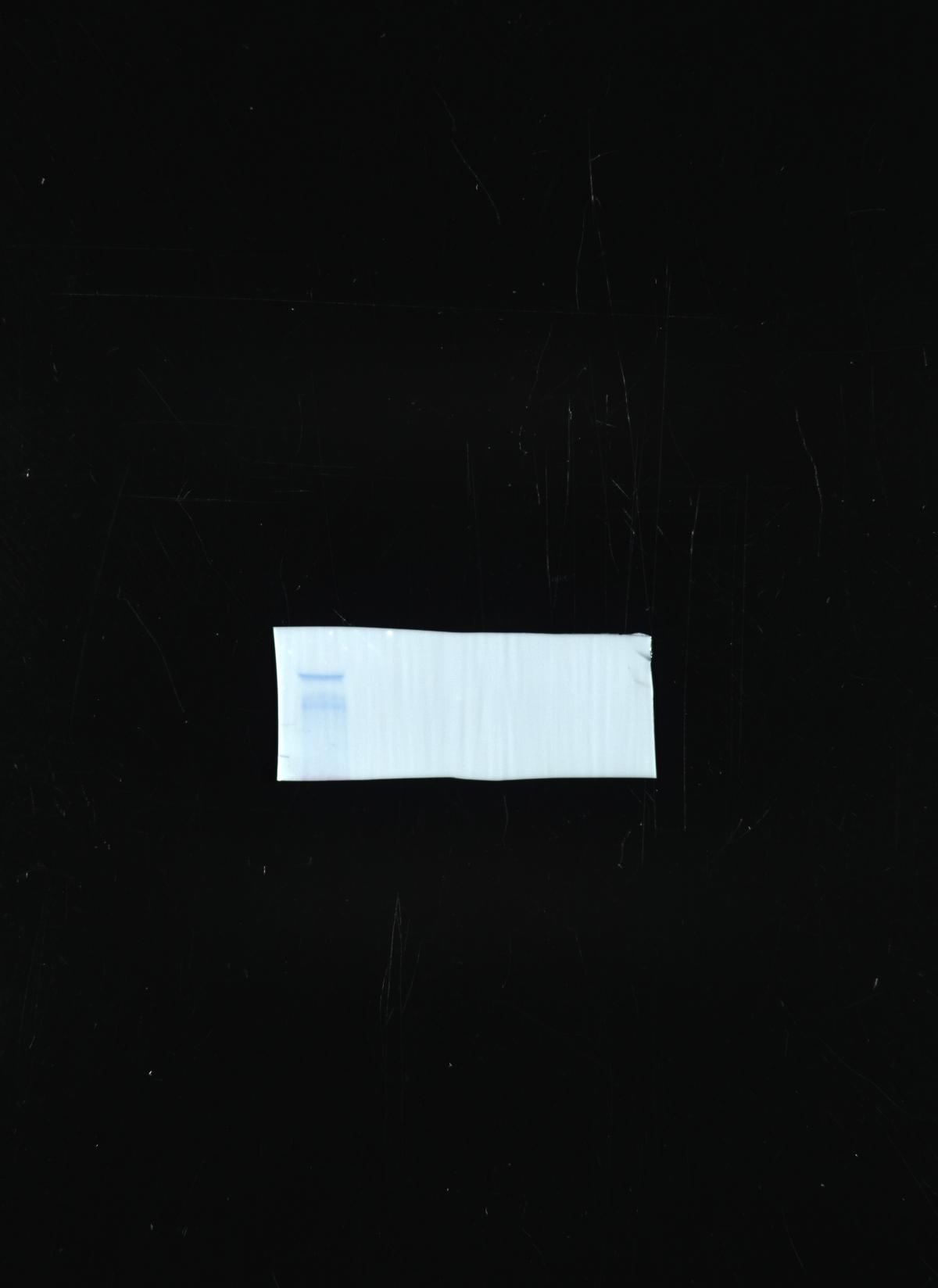

Supplement: Figure 1—source data 1. [file elife-105821-fig1-data1.zip › Figure 1-source data 1/Original files for western blot analysis displayed in Figure 1D/SNAP HeLa 20231122_115832_Ch/SNAP HeLa 20231122_115832_Ch-Marker.jpg]

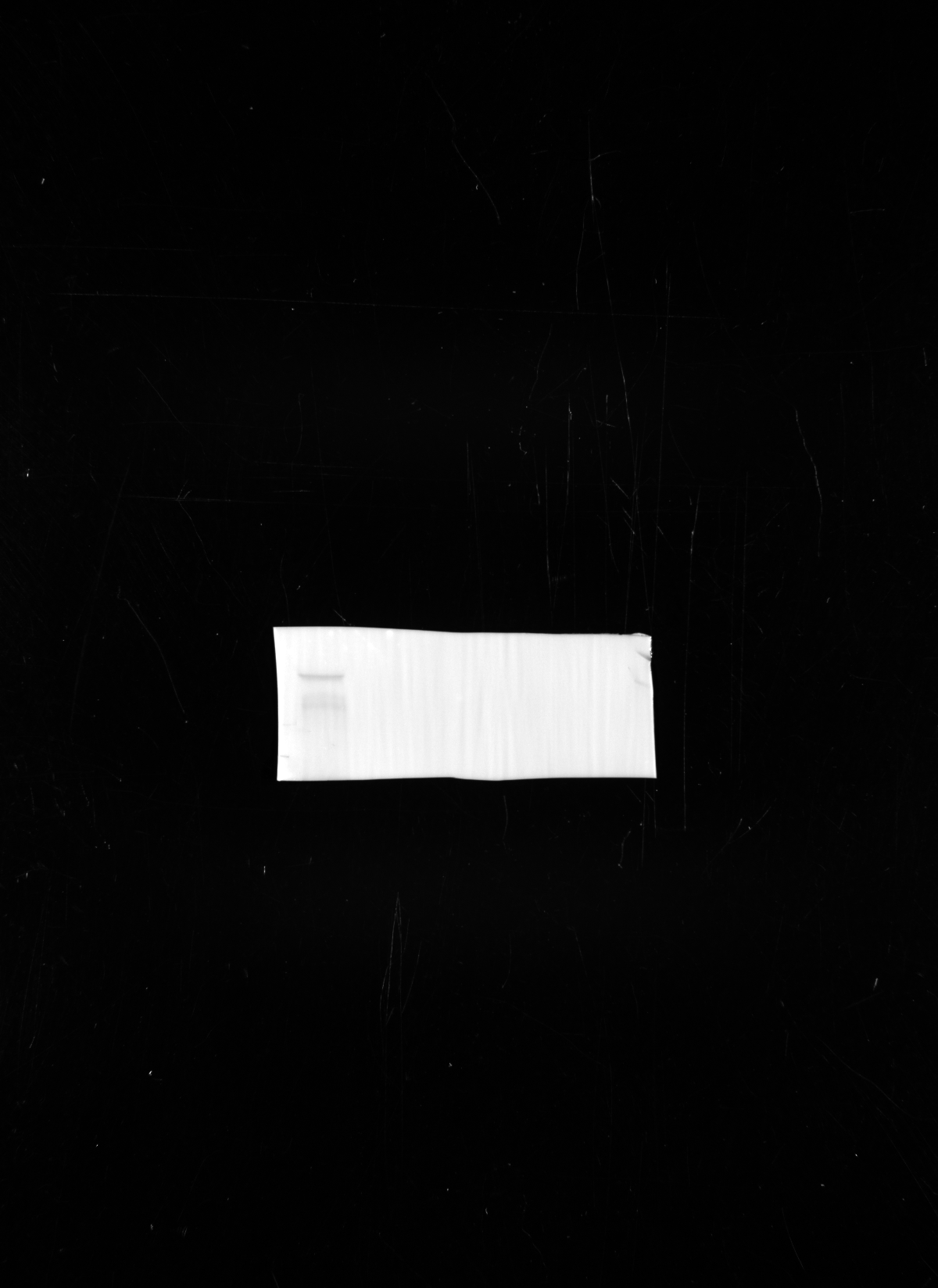

Supplement: Figure 1—source data 1. [file elife-105821-fig1-data1.zip › Figure 1-source data 1/Original files for western blot analysis displayed in Figure 1D/SNAP HeLa 20231122_115832_Ch/SNAP HeLa 20231122_115832_Ch-Marker.tif]

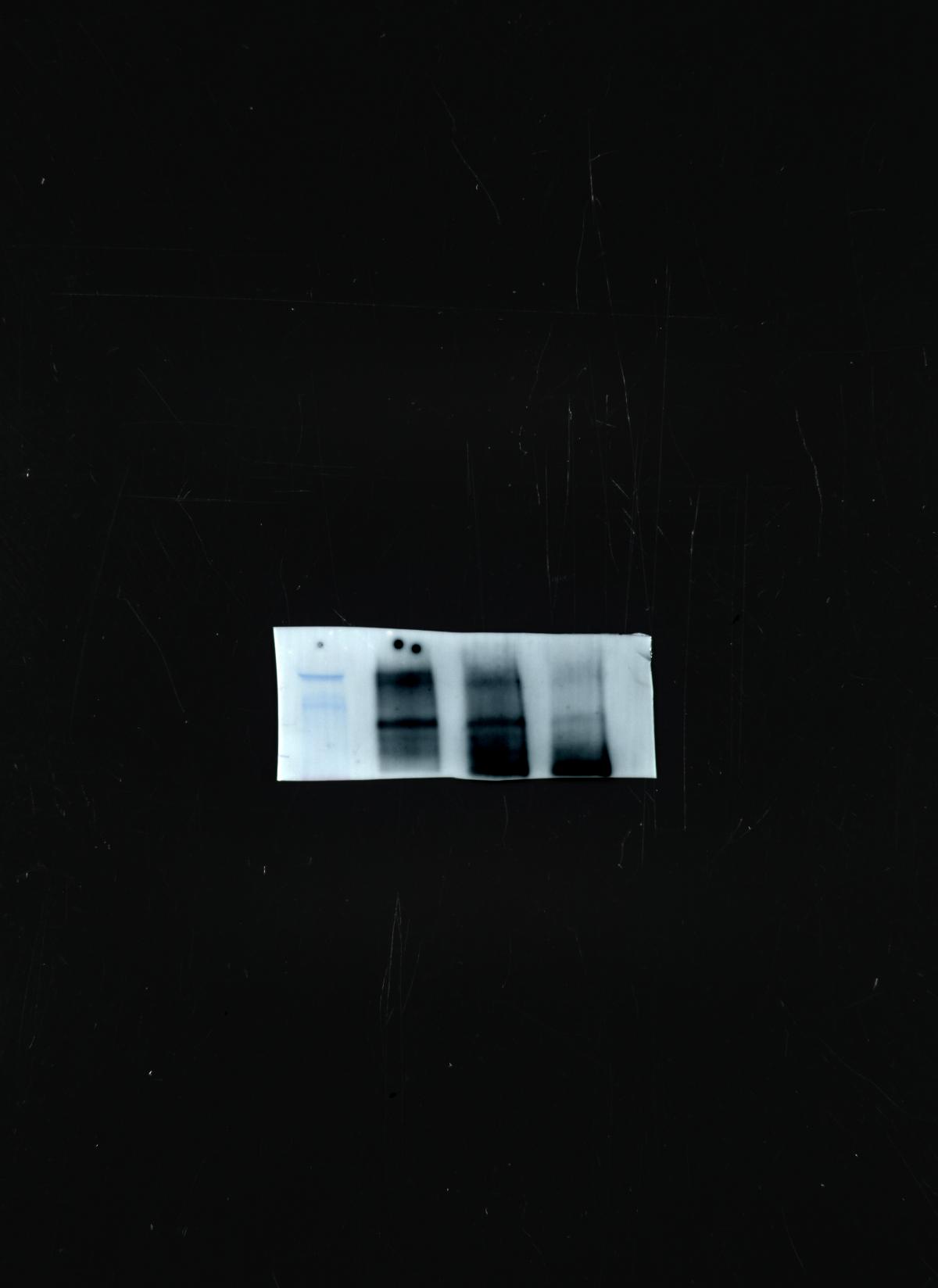

Supplement: Figure 1—source data 1. [file elife-105821-fig1-data1.zip › Figure 1-source data 1/Original files for western blot analysis displayed in Figure 1D/SNAP HeLa 20231122_115832_Ch/SNAP HeLa 20231122_115832_Ch_Chemi+Marker.jpg]

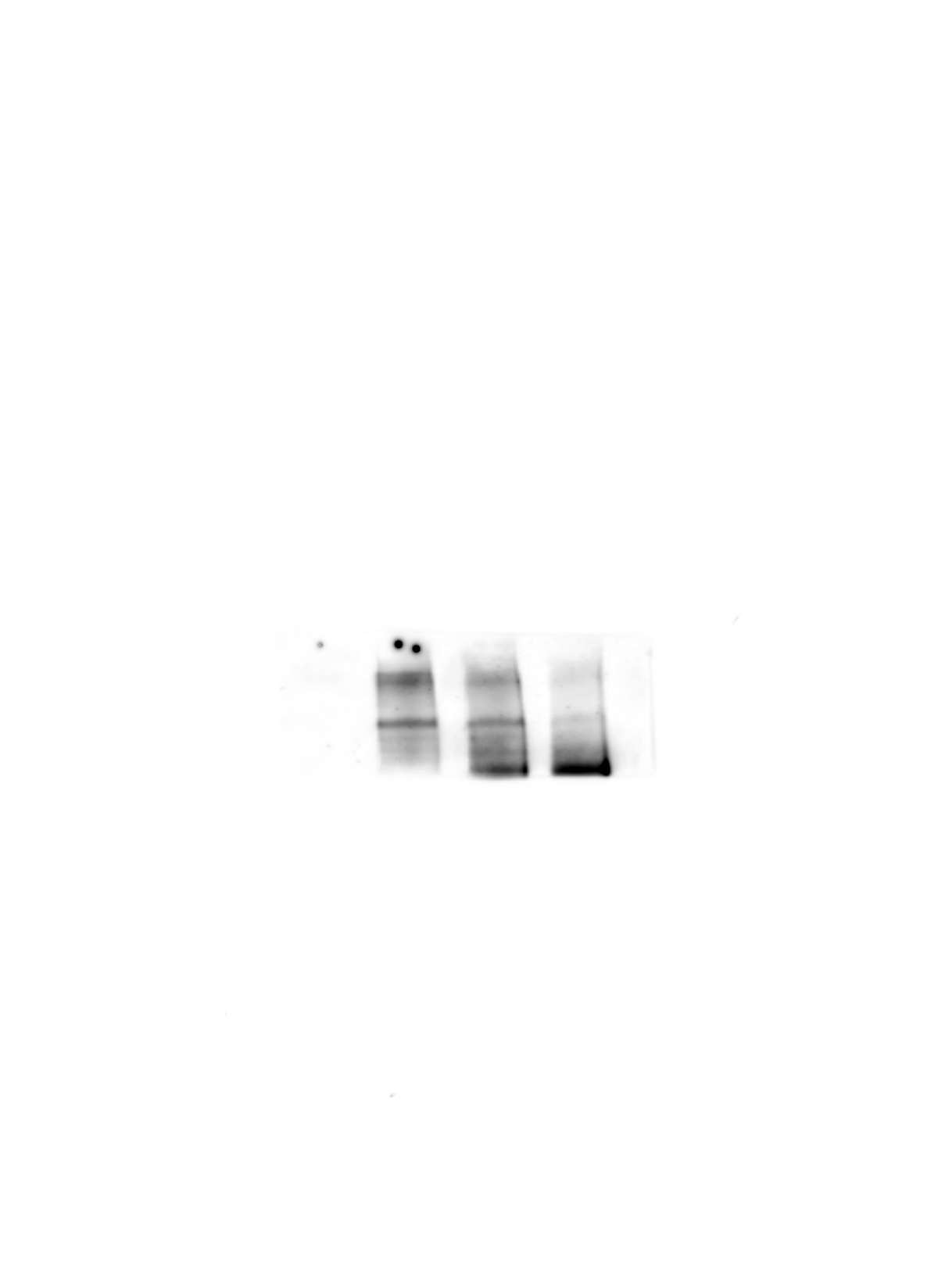

Supplement: Figure 1—source data 1. [file elife-105821-fig1-data1.zip › Figure 1-source data 1/Original files for western blot analysis displayed in Figure 1D/SNAP HeLa 20231122_115832_Ch/SNAP HeLa 20231122_115832_Ch_Chemi-2.tif]

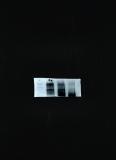

Supplement: Figure 1—source data 1. [file elife-105821-fig1-data1.zip › Figure 1-source data 1/Original files for western blot analysis displayed in Figure 1D/SNAP HeLa 20231122_115832_Ch/SNAP HeLa 20231122_115832_Ch_Thumb.jpg]

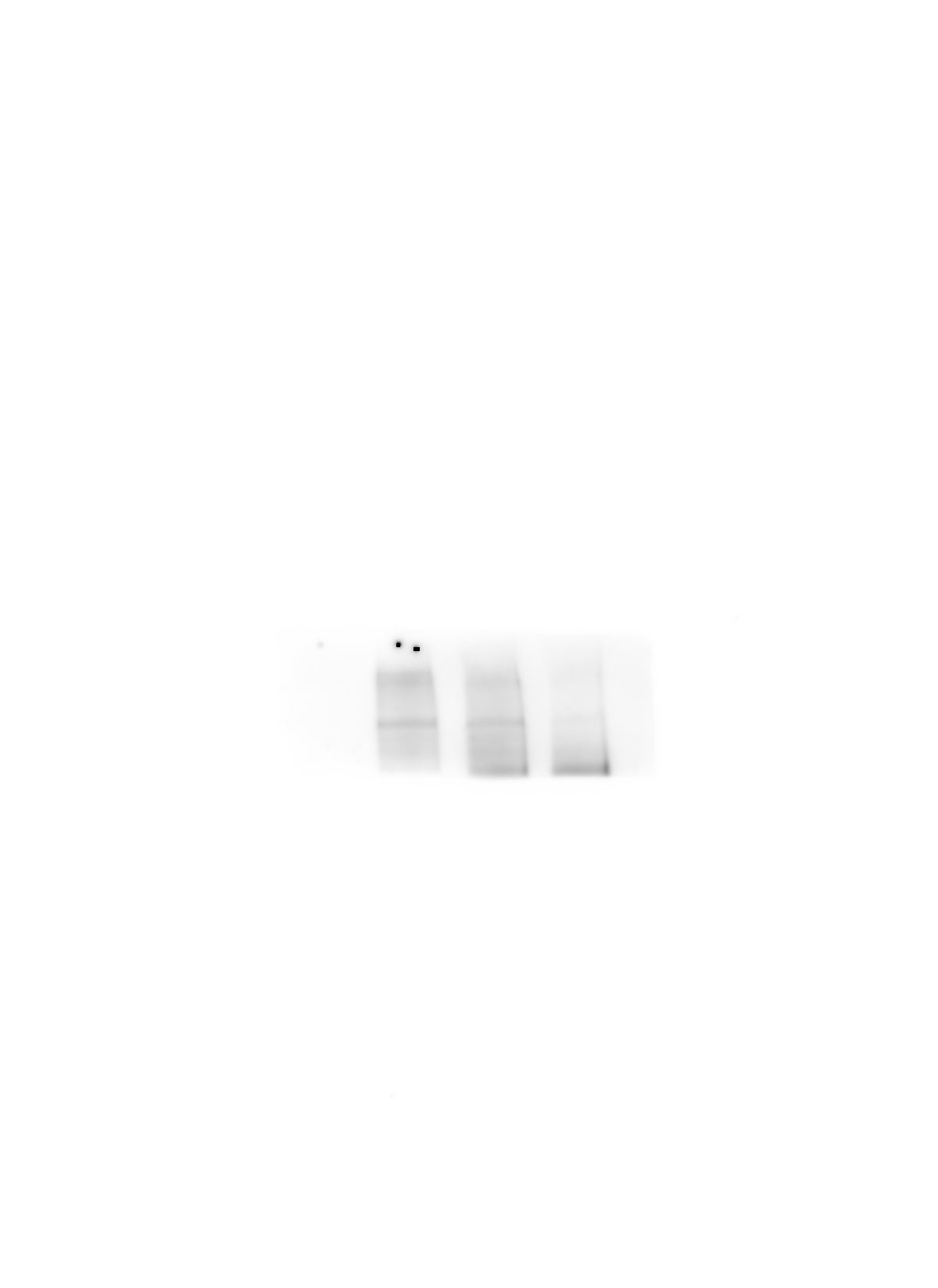

Supplement: Figure 1—source data 1. [file elife-105821-fig1-data1.zip › Figure 1-source data 1/Original files for western blot analysis displayed in Figure 1D/SNAP HeLa 20231122_115832_Ch/SNAP HeLa 20231122_115832_Ch_Chemi.tif]

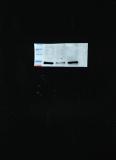

Supplement: Figure 1—source data 1. [file elife-105821-fig1-data1.zip › Figure 1-source data 1/Original files for western blot analysis displayed in Figure 1D/Vps35 HeLa 20231122_121235_Ch/Vps35 HeLa 20231122_121235_Ch_Thumb.jpg]

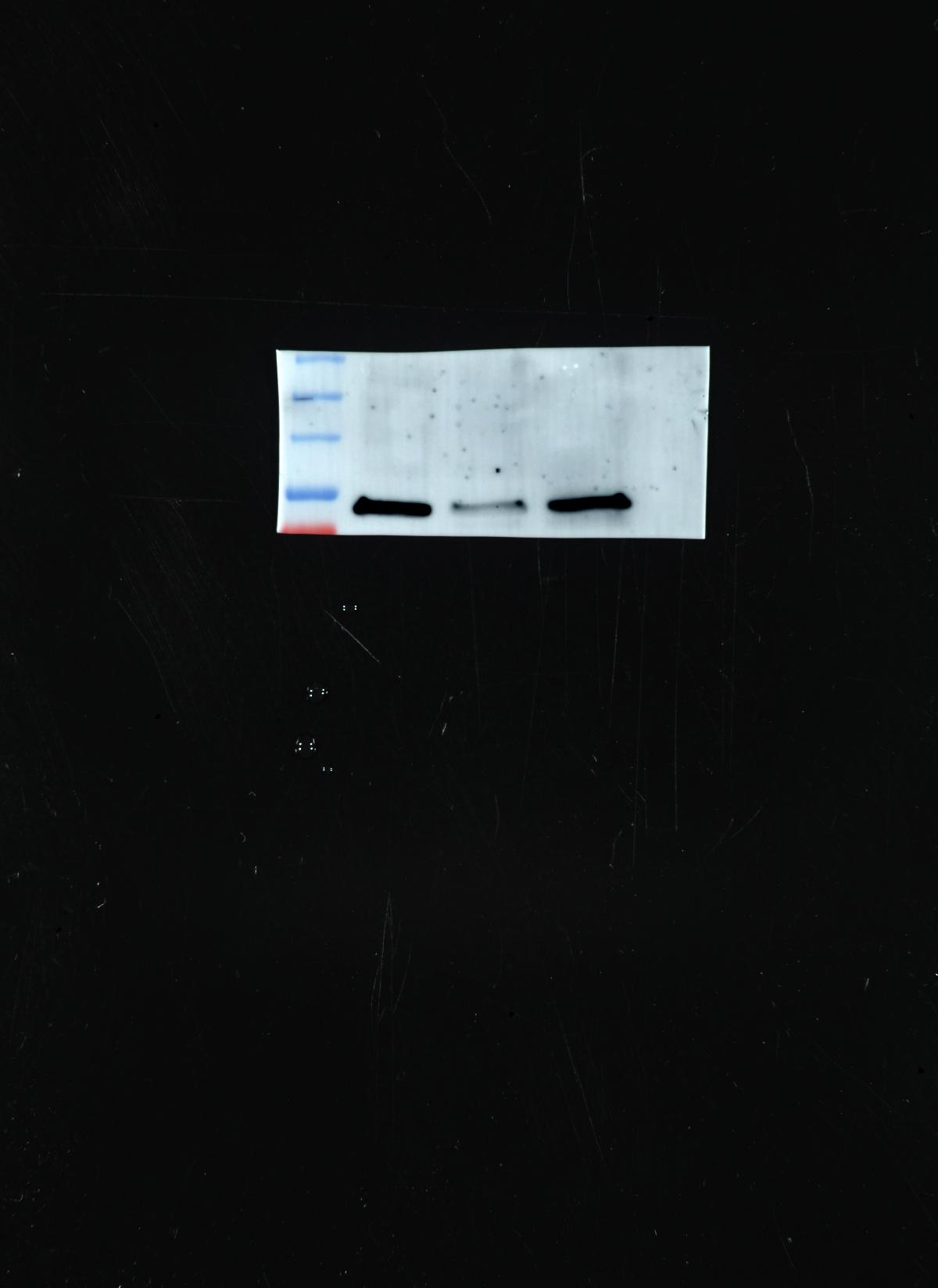

Supplement: Figure 1—source data 1. [file elife-105821-fig1-data1.zip › Figure 1-source data 1/Original files for western blot analysis displayed in Figure 1D/Vps35 HeLa 20231122_121235_Ch/Vps35 HeLa 20231122_121235_Ch_Chemi+Marker.jpg]

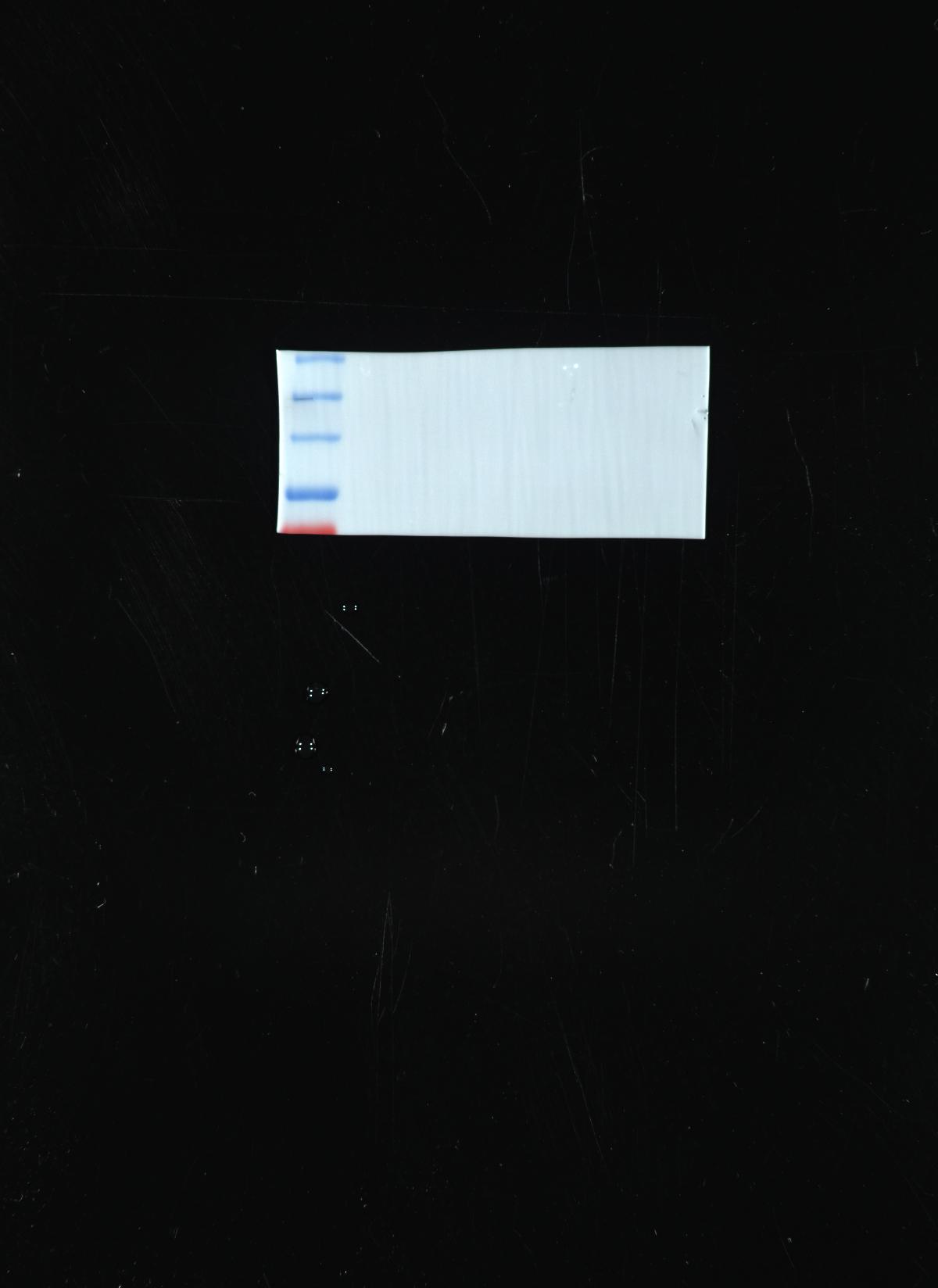

Supplement: Figure 1—source data 1. [file elife-105821-fig1-data1.zip › Figure 1-source data 1/Original files for western blot analysis displayed in Figure 1D/Vps35 HeLa 20231122_121235_Ch/Vps35 HeLa 20231122_121235_Ch-Marker.jpg]

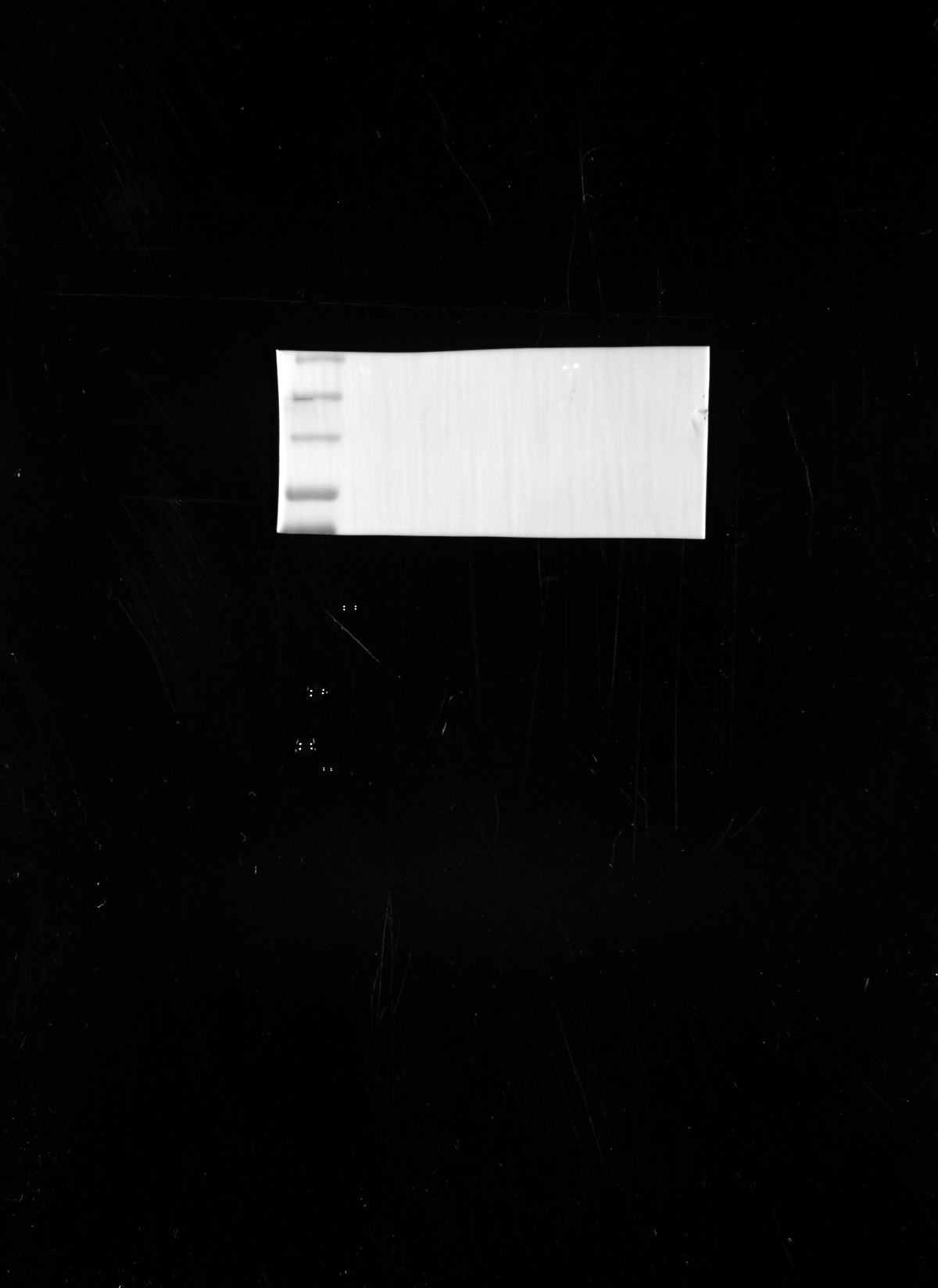

Supplement: Figure 1—source data 1. [file elife-105821-fig1-data1.zip › Figure 1-source data 1/Original files for western blot analysis displayed in Figure 1D/Vps35 HeLa 20231122_121235_Ch/Vps35 HeLa 20231122_121235_Ch-Marker.tif]

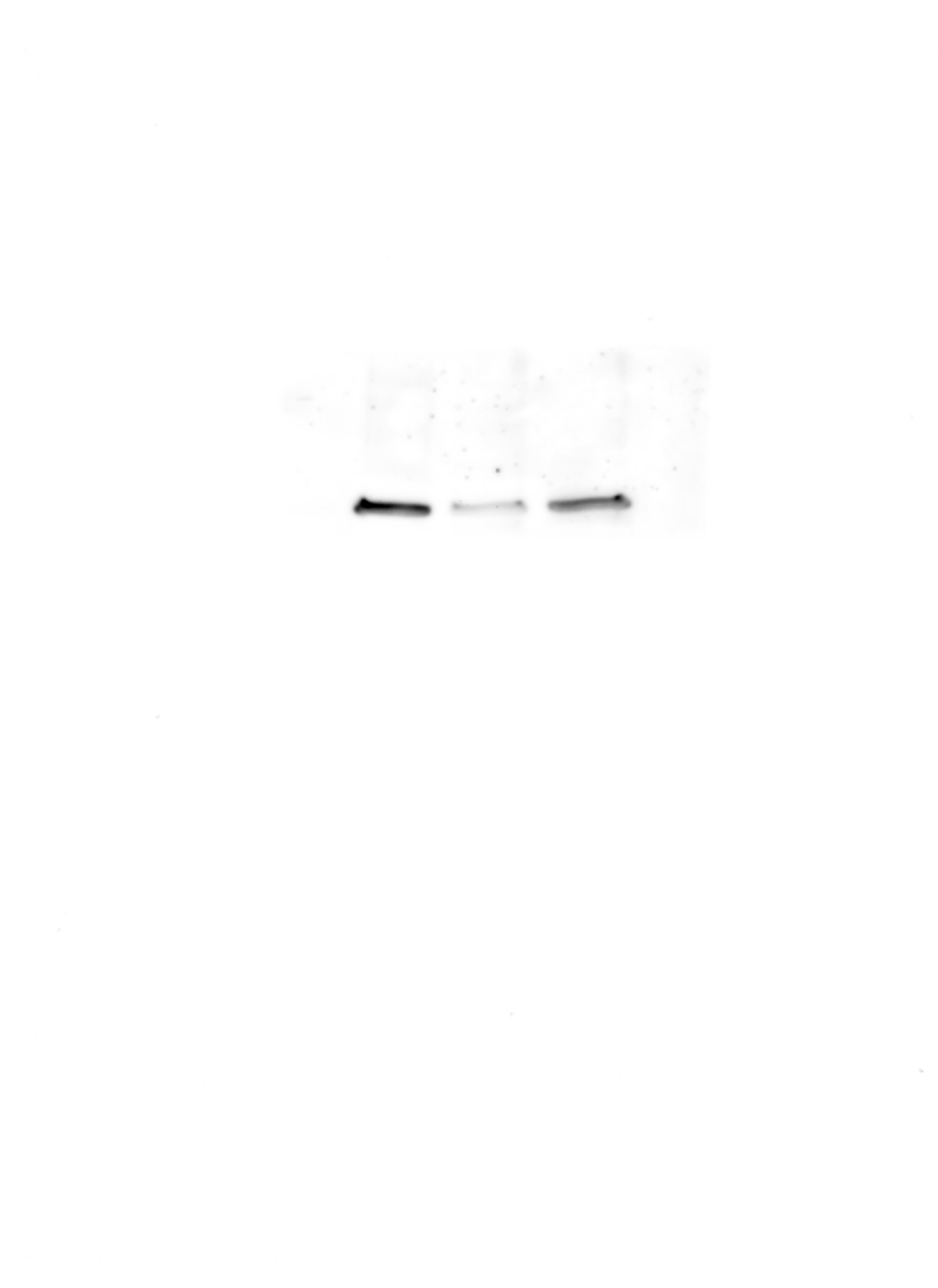

Supplement: Figure 1—source data 1. [file elife-105821-fig1-data1.zip › Figure 1-source data 1/Original files for western blot analysis displayed in Figure 1D/Vps35 HeLa 20231122_121235_Ch/Vps35 HeLa 20231122_121235_Ch_Chemi-2.tif]

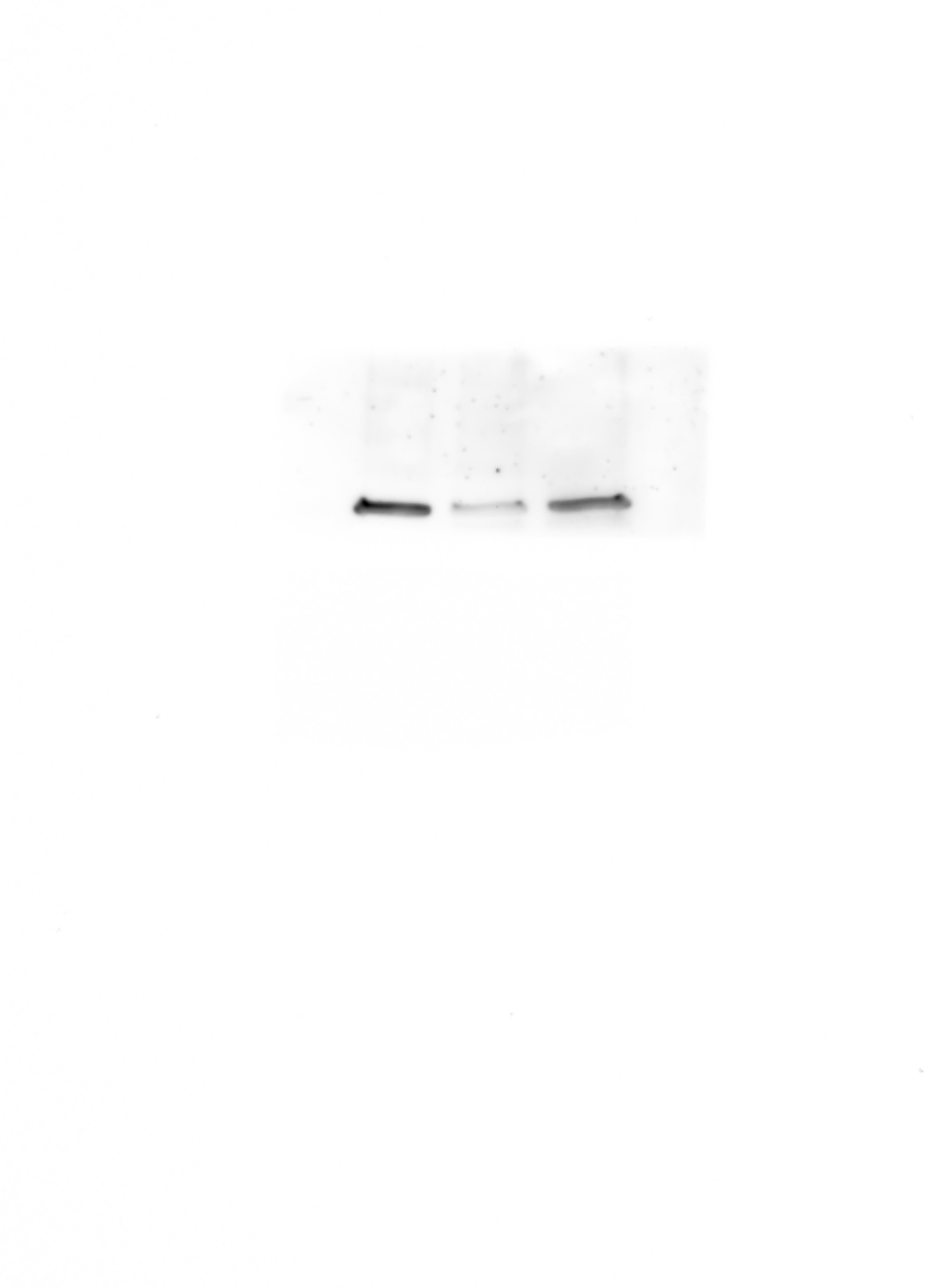

Supplement: Figure 1—source data 1. [file elife-105821-fig1-data1.zip › Figure 1-source data 1/Original files for western blot analysis displayed in Figure 1D/Vps35 HeLa 20231122_121235_Ch/Vps35 HeLa 20231122_121235_Ch_Chemi.tif]

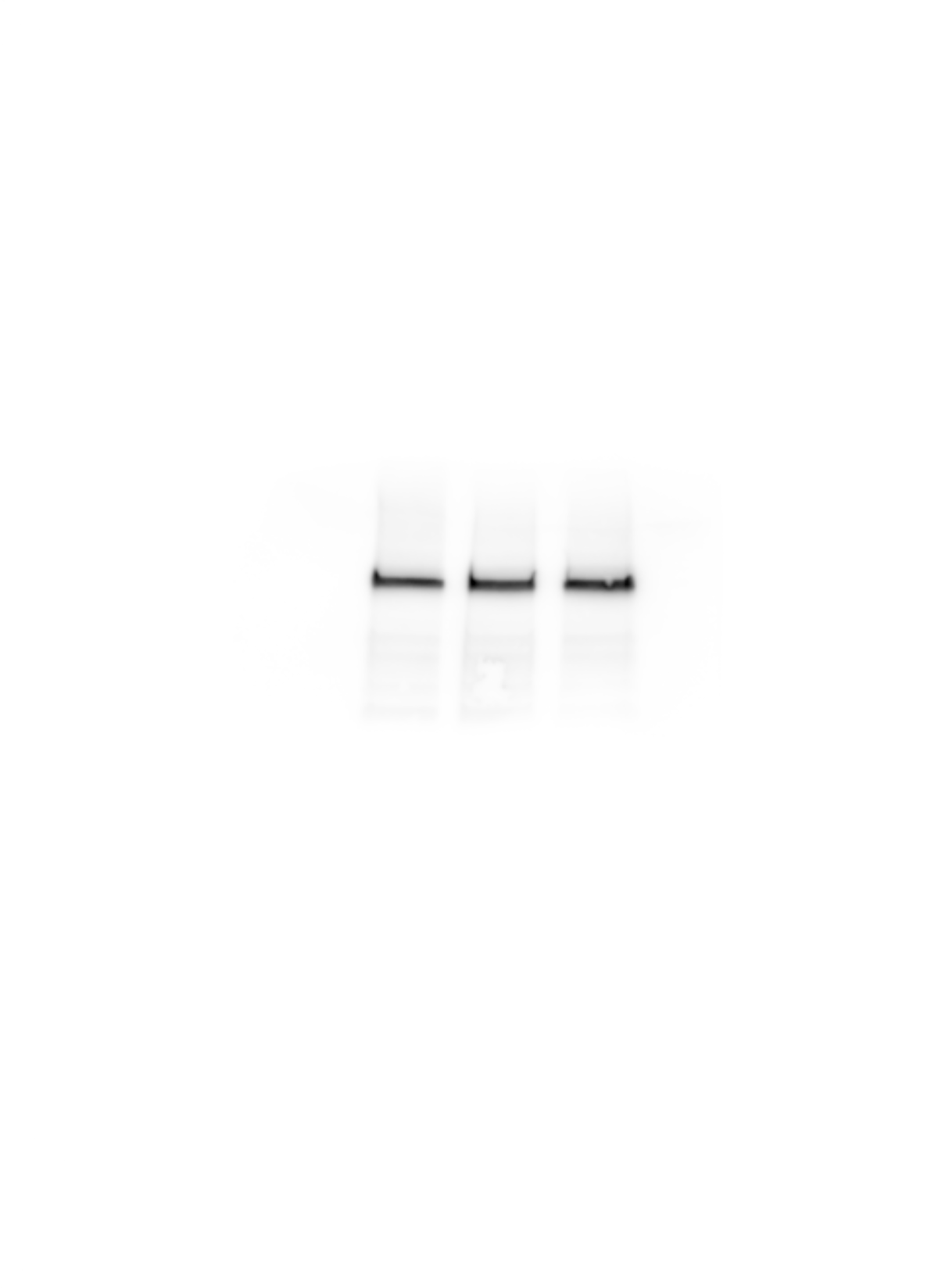

Supplement: Figure 1—source data 1. [file elife-105821-fig1-data1.zip › Figure 1-source data 1/Original files for western blot analysis displayed in Figure 1E/clathrin hela_snap 20230322_114923_Ch/clathrin hela_snap 20230322_114923_Ch_Chemi.tif]

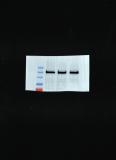

Supplement: Figure 1—source data 1. [file elife-105821-fig1-data1.zip › Figure 1-source data 1/Original files for western blot analysis displayed in Figure 1E/clathrin hela_snap 20230322_114923_Ch/clathrin hela_snap 20230322_114923_Ch_Thumb.jpg]

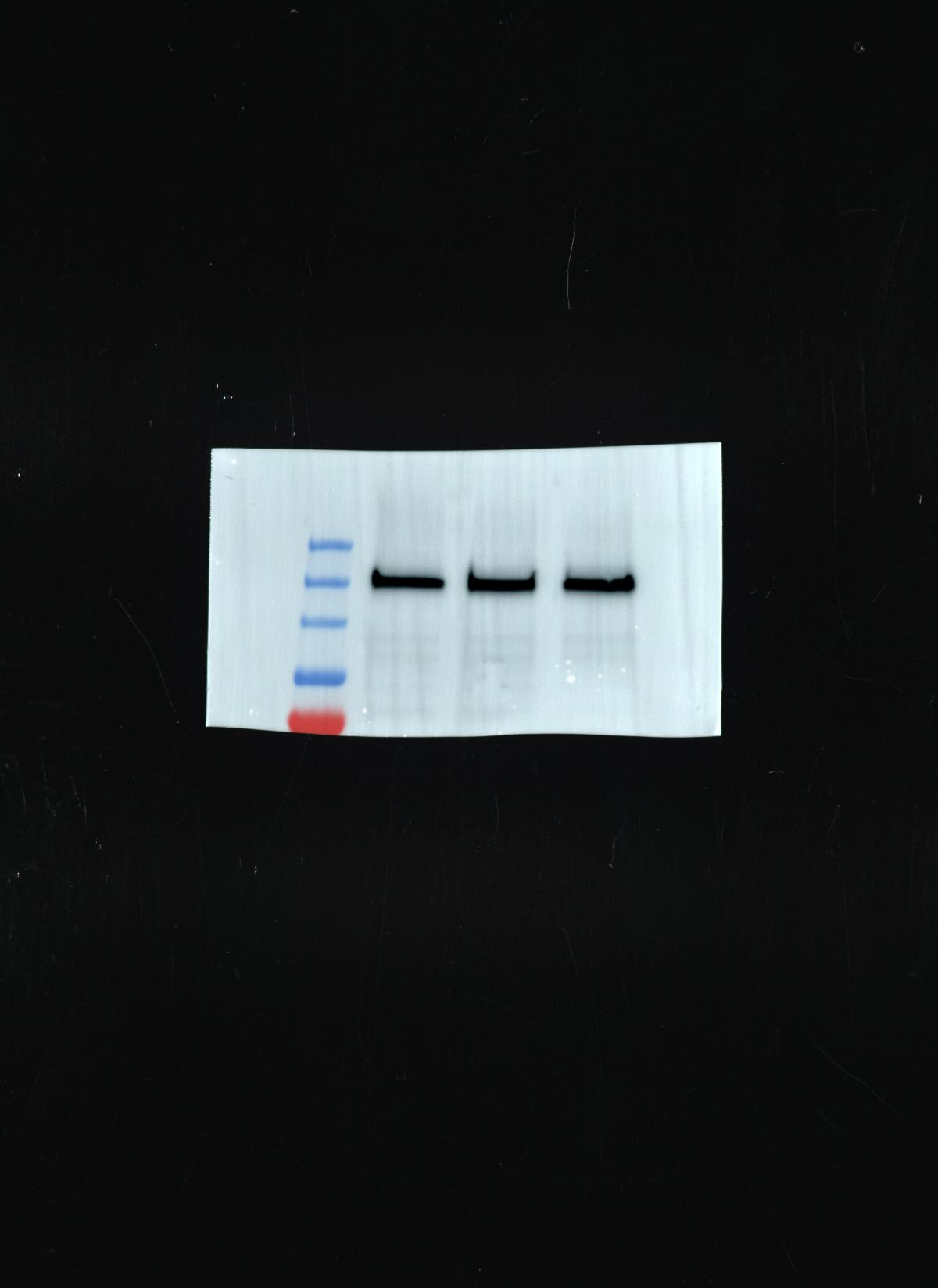

Supplement: Figure 1—source data 1. [file elife-105821-fig1-data1.zip › Figure 1-source data 1/Original files for western blot analysis displayed in Figure 1E/clathrin hela_snap 20230322_114923_Ch/clathrin hela_snap 20230322_114923_Ch_Chemi+Marker.jpg]

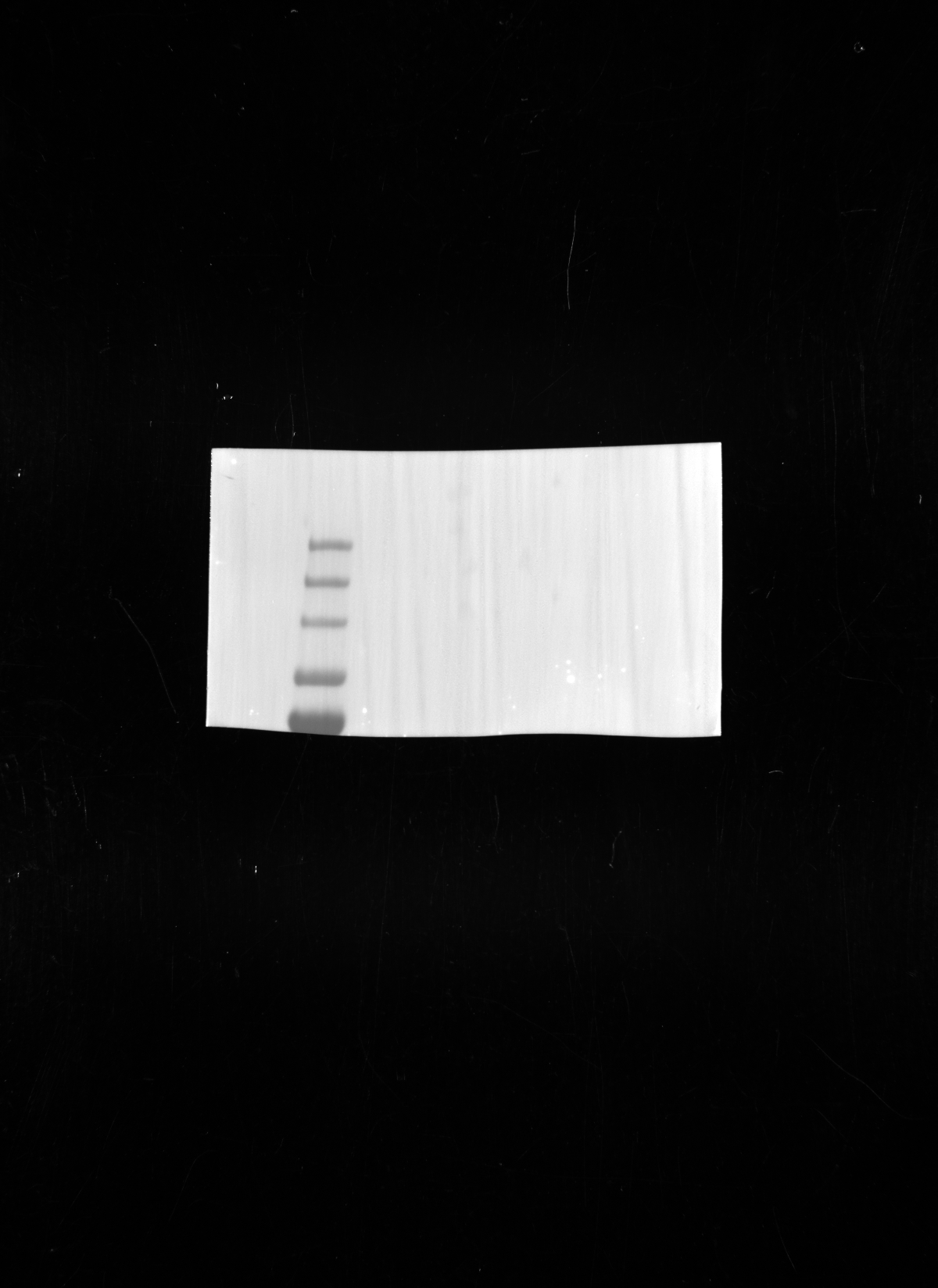

Supplement: Figure 1—source data 1. [file elife-105821-fig1-data1.zip › Figure 1-source data 1/Original files for western blot analysis displayed in Figure 1E/clathrin hela_snap 20230322_114923_Ch/clathrin hela_snap 20230322_114923_Ch-Marker.tif]

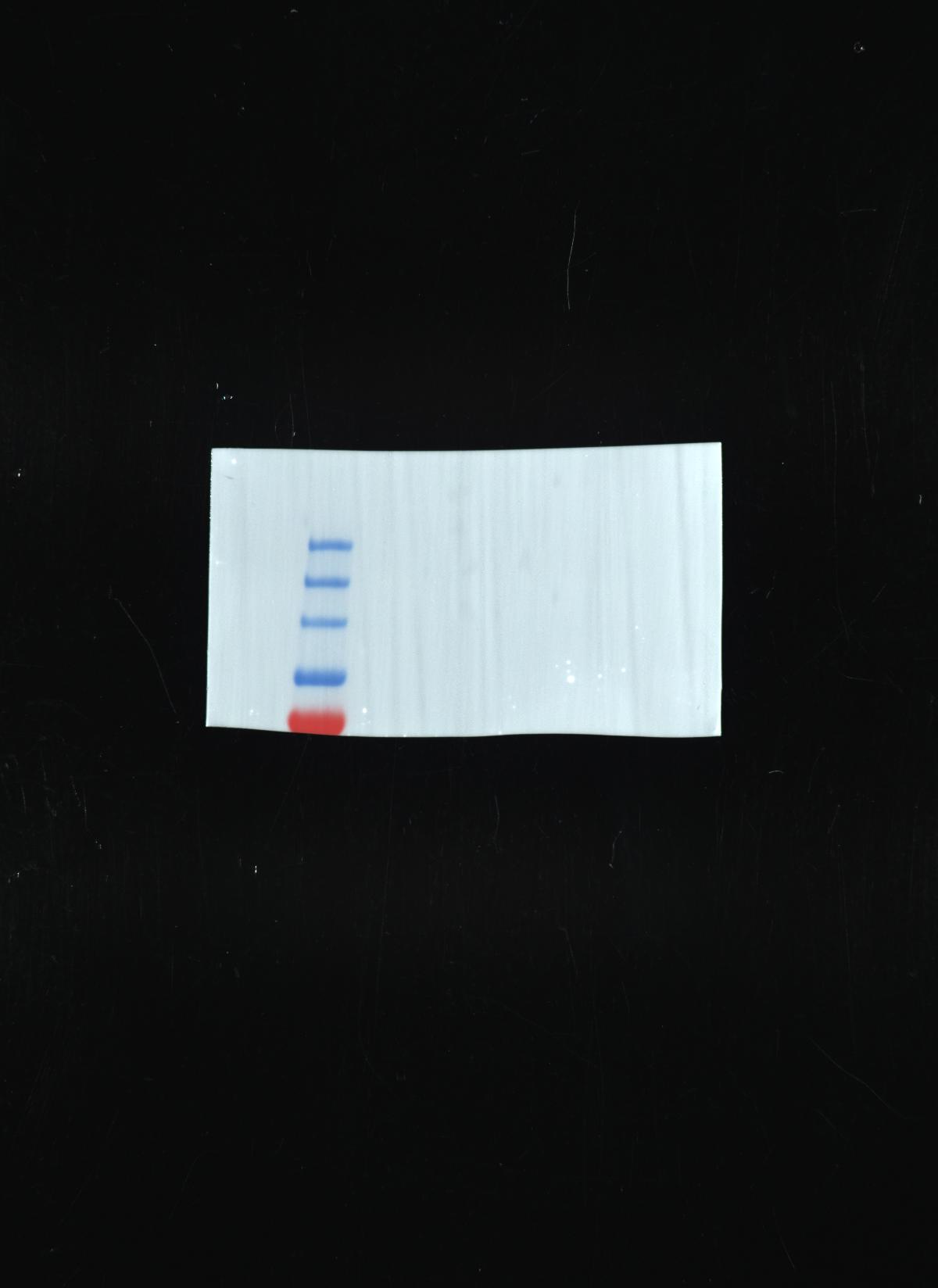

Supplement: Figure 1—source data 1. [file elife-105821-fig1-data1.zip › Figure 1-source data 1/Original files for western blot analysis displayed in Figure 1E/clathrin hela_snap 20230322_114923_Ch/clathrin hela_snap 20230322_114923_Ch-Marker.jpg]

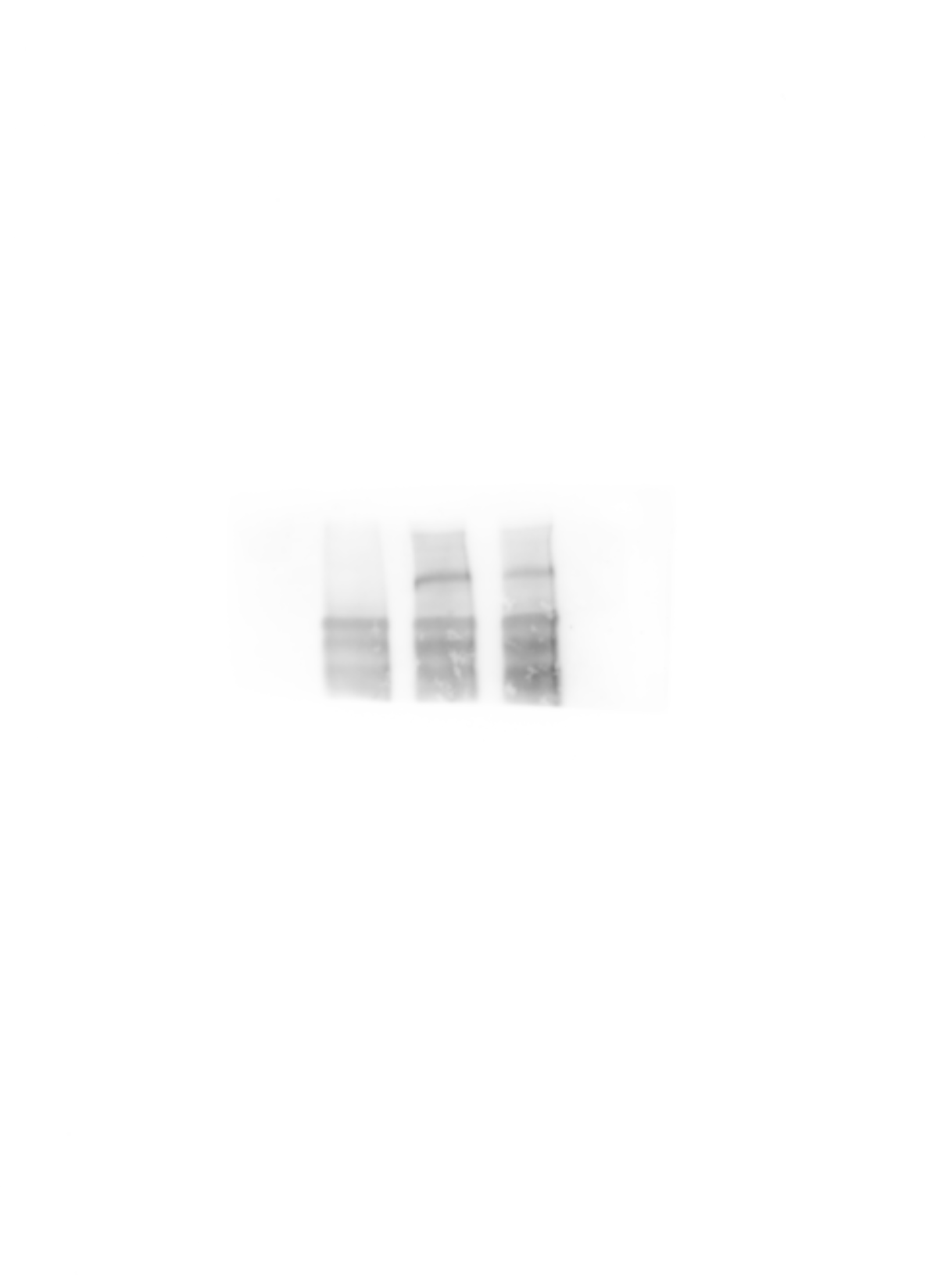

Supplement: Figure 1—source data 1. [file elife-105821-fig1-data1.zip › Figure 1-source data 1/Original files for western blot analysis displayed in Figure 1E/SNAP3 hela_snap 20230322_142556_Ch/SNAP3 hela_snap 20230322_142556_Ch_Chemi.tif]

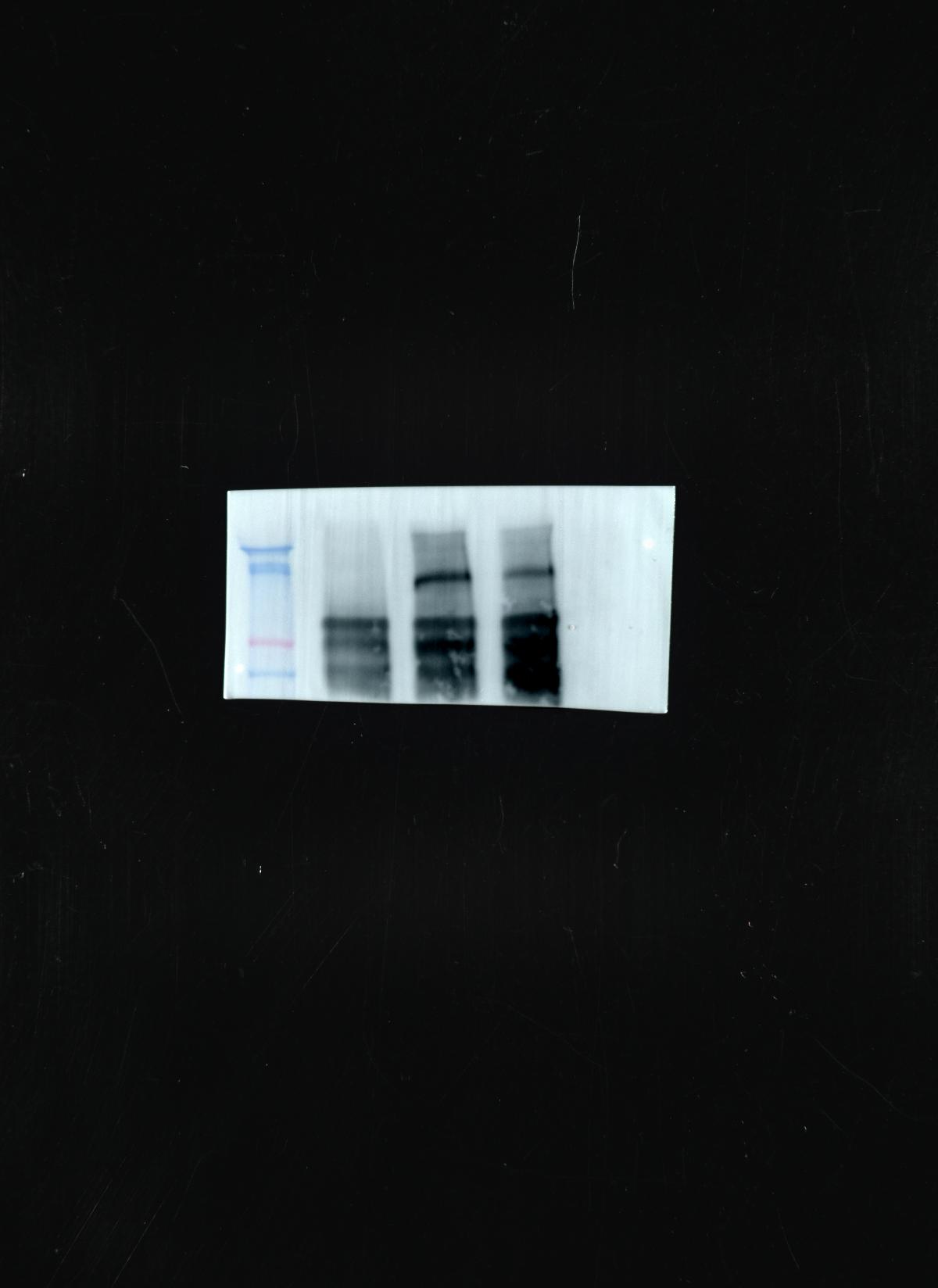

Supplement: Figure 1—source data 1. [file elife-105821-fig1-data1.zip › Figure 1-source data 1/Original files for western blot analysis displayed in Figure 1E/SNAP3 hela_snap 20230322_142556_Ch/SNAP3 hela_snap 20230322_142556_Ch_Chemi+Marker.jpg]

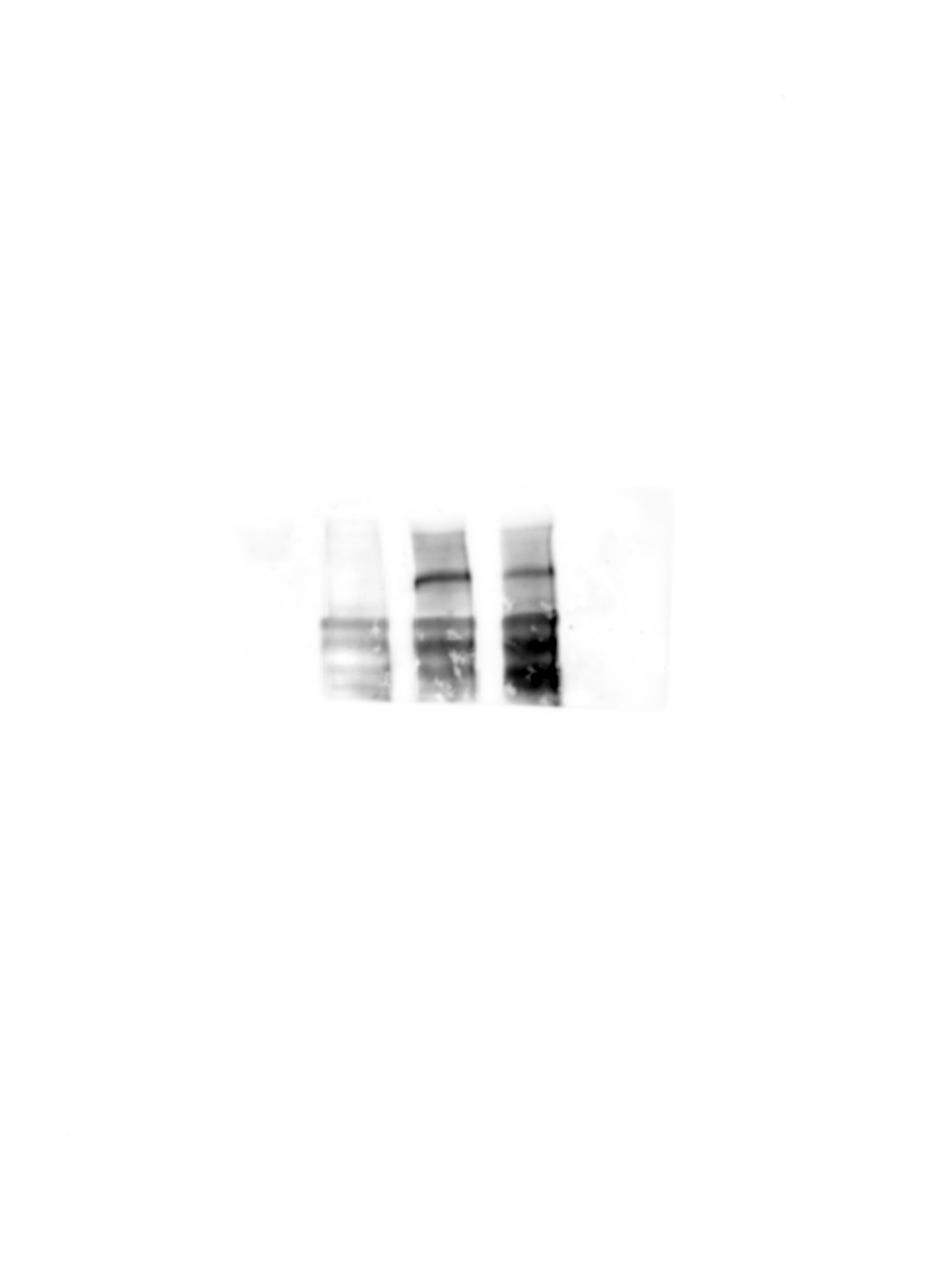

Supplement: Figure 1—source data 1. [file elife-105821-fig1-data1.zip › Figure 1-source data 1/Original files for western blot analysis displayed in Figure 1E/SNAP3 hela_snap 20230322_142556_Ch/SNAP3 hela_snap 20230322_142556_Ch_Chemi.png]

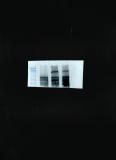

Supplement: Figure 1—source data 1. [file elife-105821-fig1-data1.zip › Figure 1-source data 1/Original files for western blot analysis displayed in Figure 1E/SNAP3 hela_snap 20230322_142556_Ch/SNAP3 hela_snap 20230322_142556_Ch_Thumb.jpg]

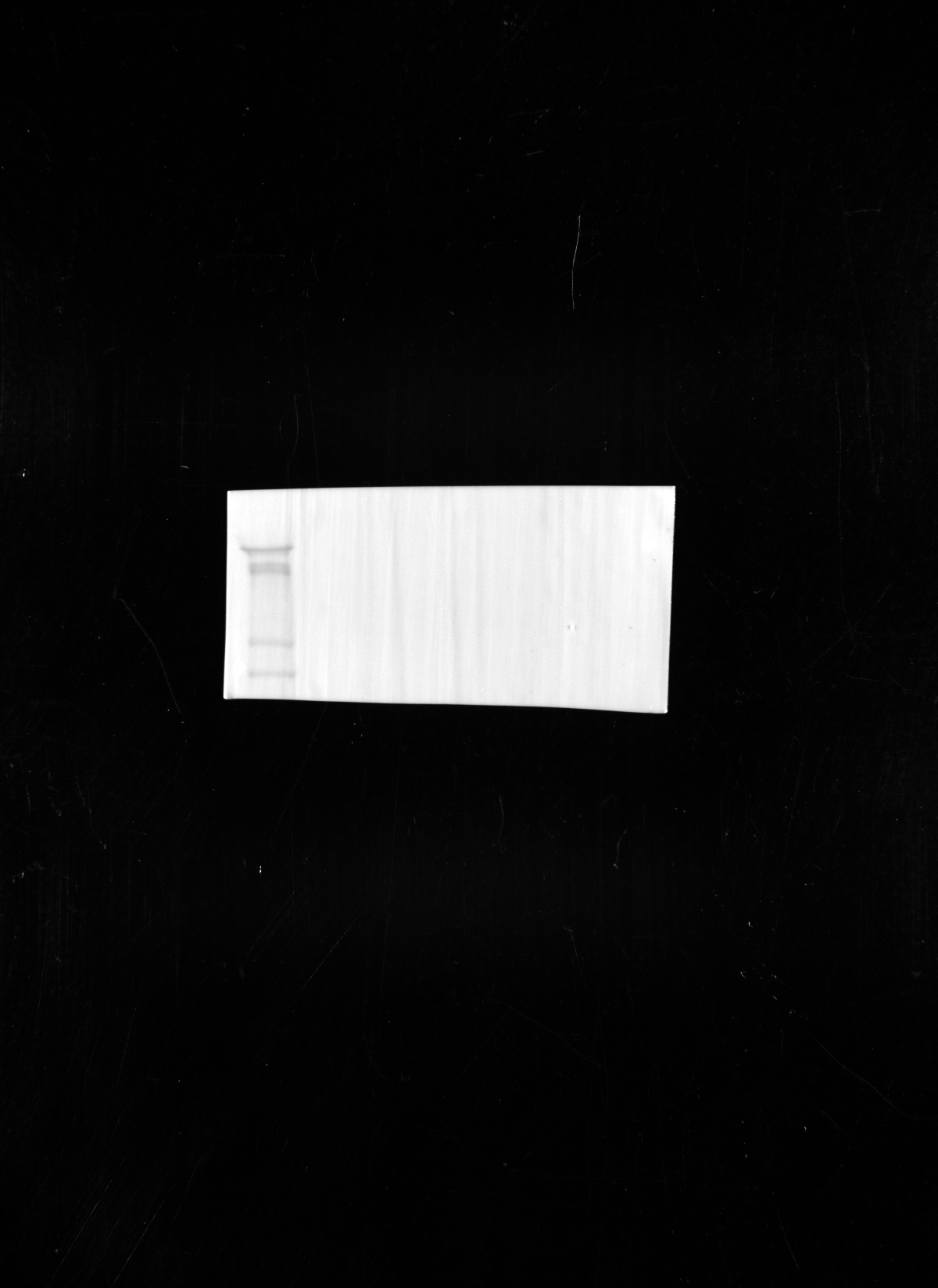

Supplement: Figure 1—source data 1. [file elife-105821-fig1-data1.zip › Figure 1-source data 1/Original files for western blot analysis displayed in Figure 1E/SNAP3 hela_snap 20230322_142556_Ch/SNAP3 hela_snap 20230322_142556_Ch-Marker.tif]

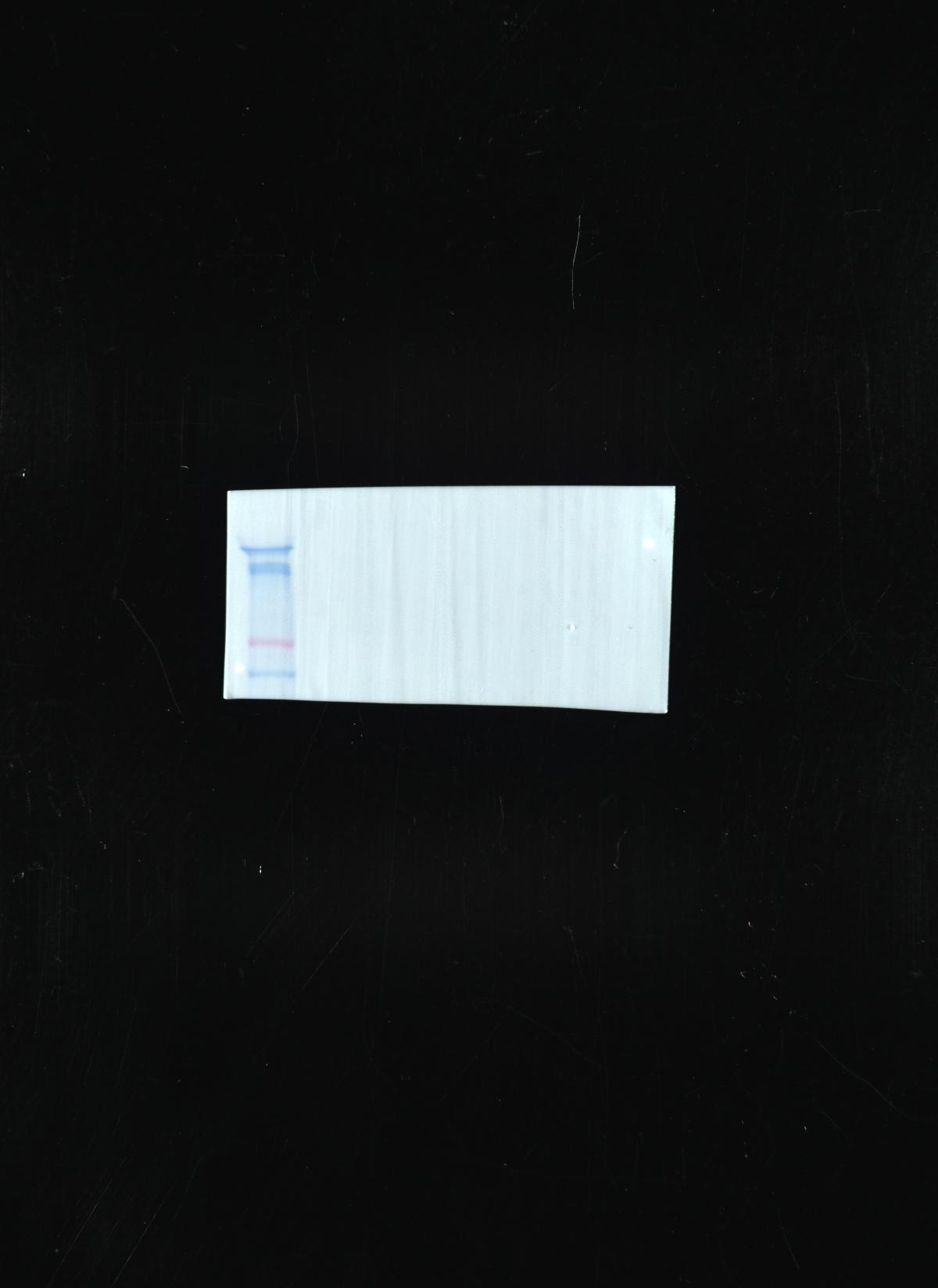

Supplement: Figure 1—source data 1. [file elife-105821-fig1-data1.zip › Figure 1-source data 1/Original files for western blot analysis displayed in Figure 1E/SNAP3 hela_snap 20230322_142556_Ch/SNAP3 hela_snap 20230322_142556_Ch-Marker.jpg]

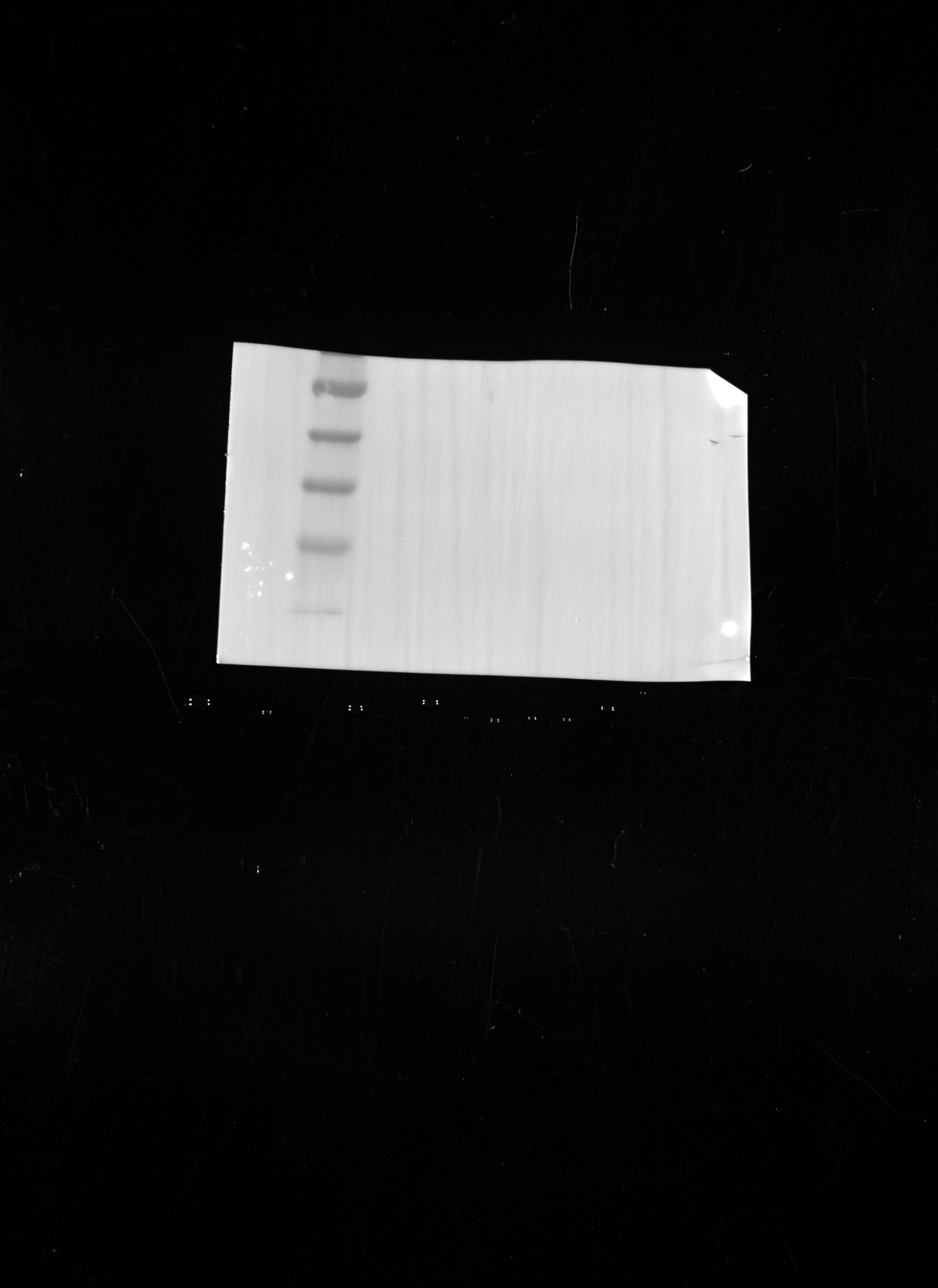

Supplement: Figure 1—source data 1. [file elife-105821-fig1-data1.zip › Figure 1-source data 1/Original files for western blot analysis displayed in Figure 1E/EA3 hela_snap 20230322_115332_Ch/EA3 hela_snap 20230322_115332_Ch-Marker.tif]

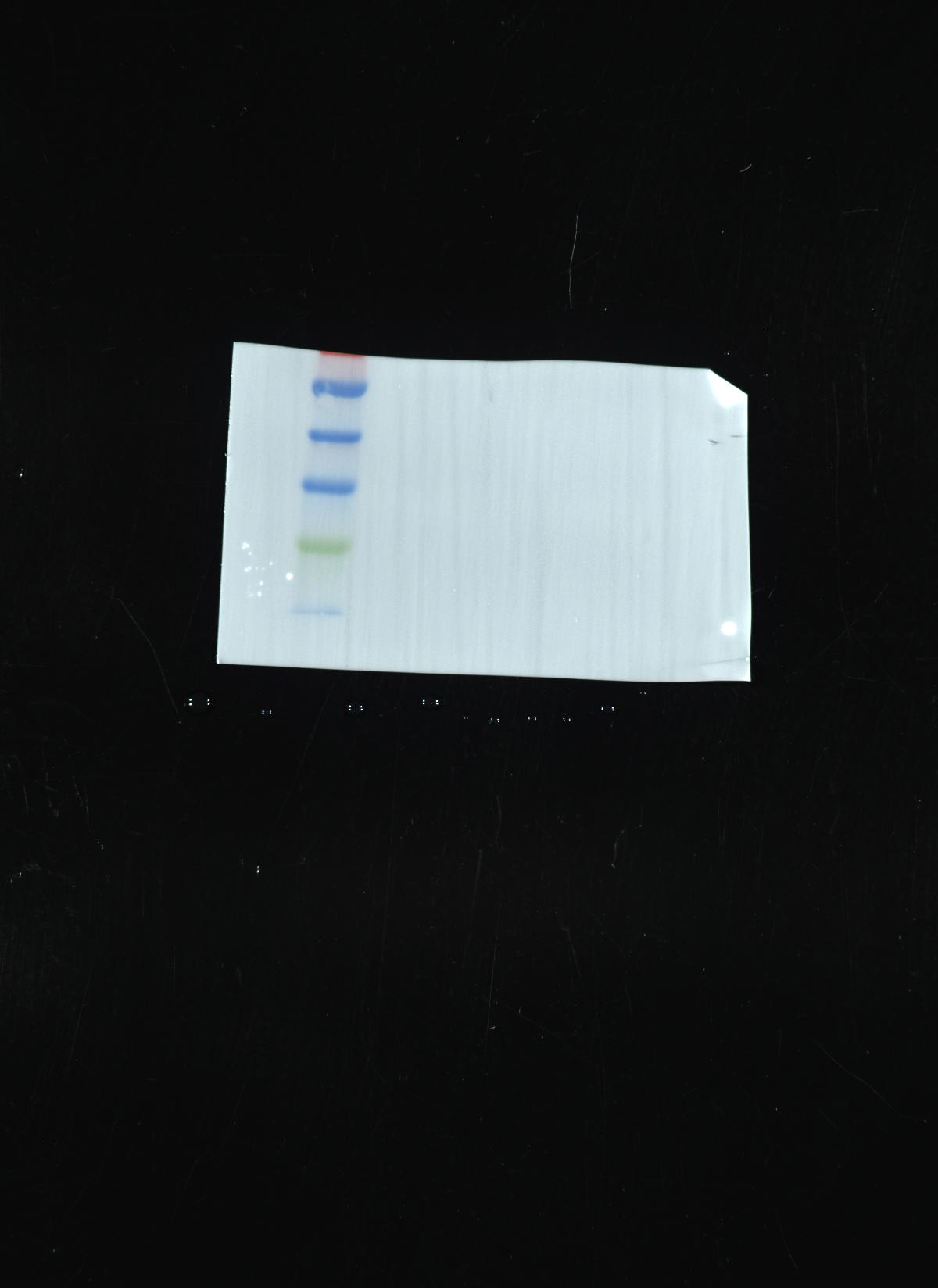

Supplement: Figure 1—source data 1. [file elife-105821-fig1-data1.zip › Figure 1-source data 1/Original files for western blot analysis displayed in Figure 1E/EA3 hela_snap 20230322_115332_Ch/EA3 hela_snap 20230322_115332_Ch-Marker.jpg]

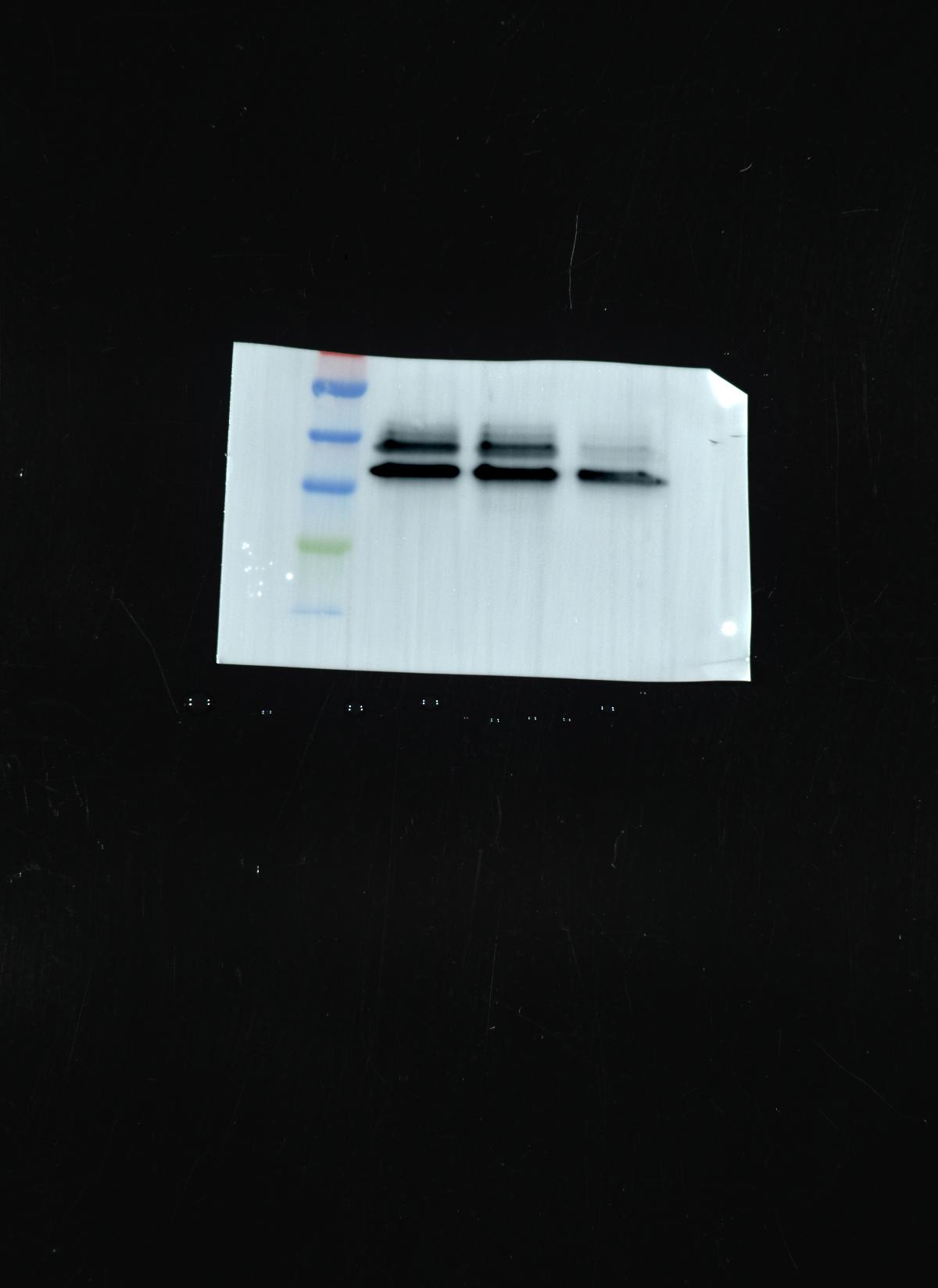

Supplement: Figure 1—source data 1. [file elife-105821-fig1-data1.zip › Figure 1-source data 1/Original files for western blot analysis displayed in Figure 1E/EA3 hela_snap 20230322_115332_Ch/EA3 hela_snap 20230322_115332_Ch_Chemi+Marker.jpg]

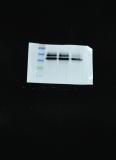

Supplement: Figure 1—source data 1. [file elife-105821-fig1-data1.zip › Figure 1-source data 1/Original files for western blot analysis displayed in Figure 1E/EA3 hela_snap 20230322_115332_Ch/EA3 hela_snap 20230322_115332_Ch_Thumb.jpg]

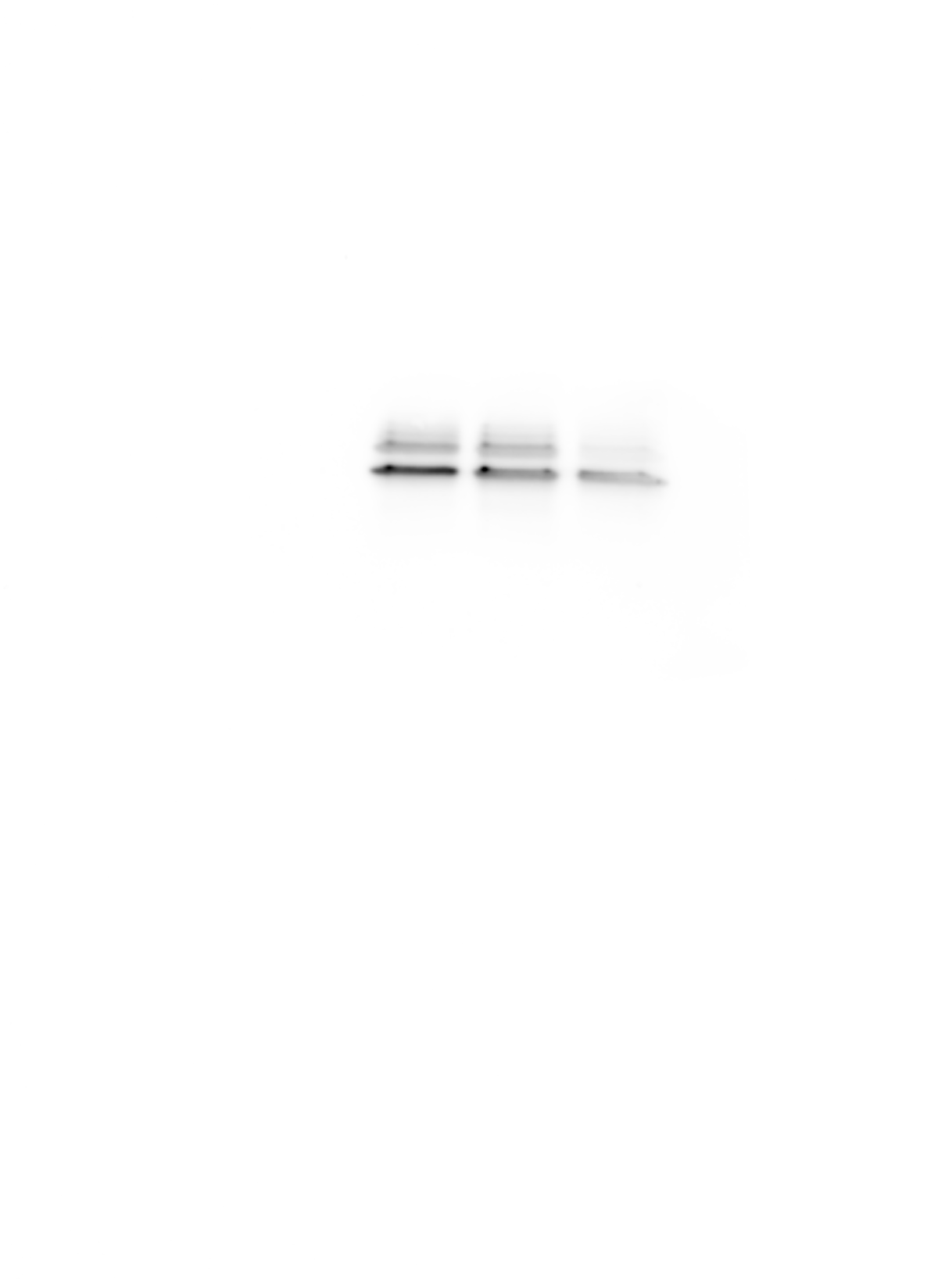

Supplement: Figure 1—source data 1. [file elife-105821-fig1-data1.zip › Figure 1-source data 1/Original files for western blot analysis displayed in Figure 1E/EA3 hela_snap 20230322_115332_Ch/EA3 hela_snap 20230322_115332_Ch_Chemi.tif]

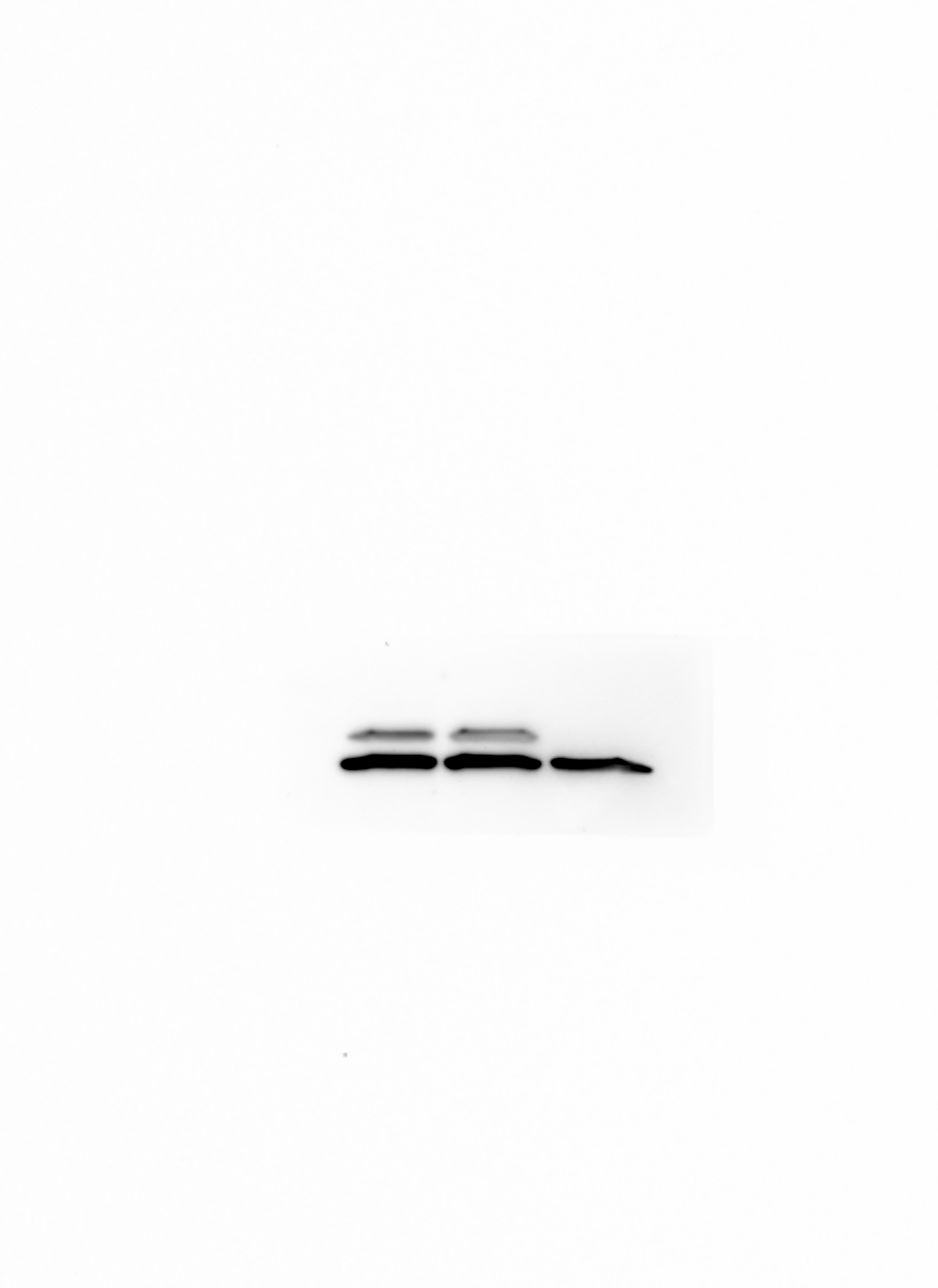

Supplement: Figure 1—source data 1. [file elife-105821-fig1-data1.zip › Figure 1-source data 1/Original files for western blot analysis displayed in Figure 1F/EA3 20231115_113608_Ch/EA3 20231115_113608_Ch_Chemi-1.tif]

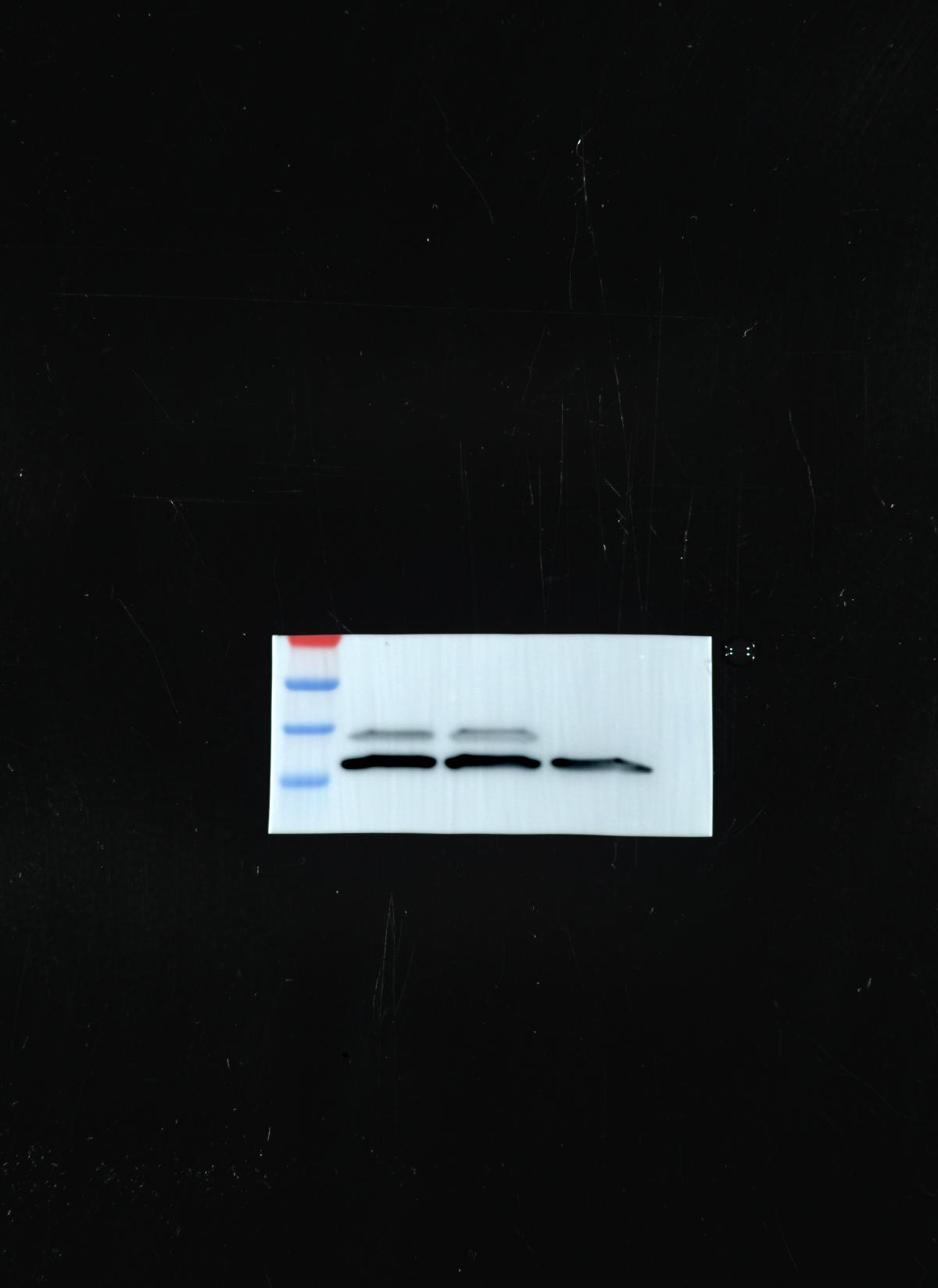

Supplement: Figure 1—source data 1. [file elife-105821-fig1-data1.zip › Figure 1-source data 1/Original files for western blot analysis displayed in Figure 1F/EA3 20231115_113608_Ch/EA3 20231115_113608_Ch_Chemi+Marker.jpg]

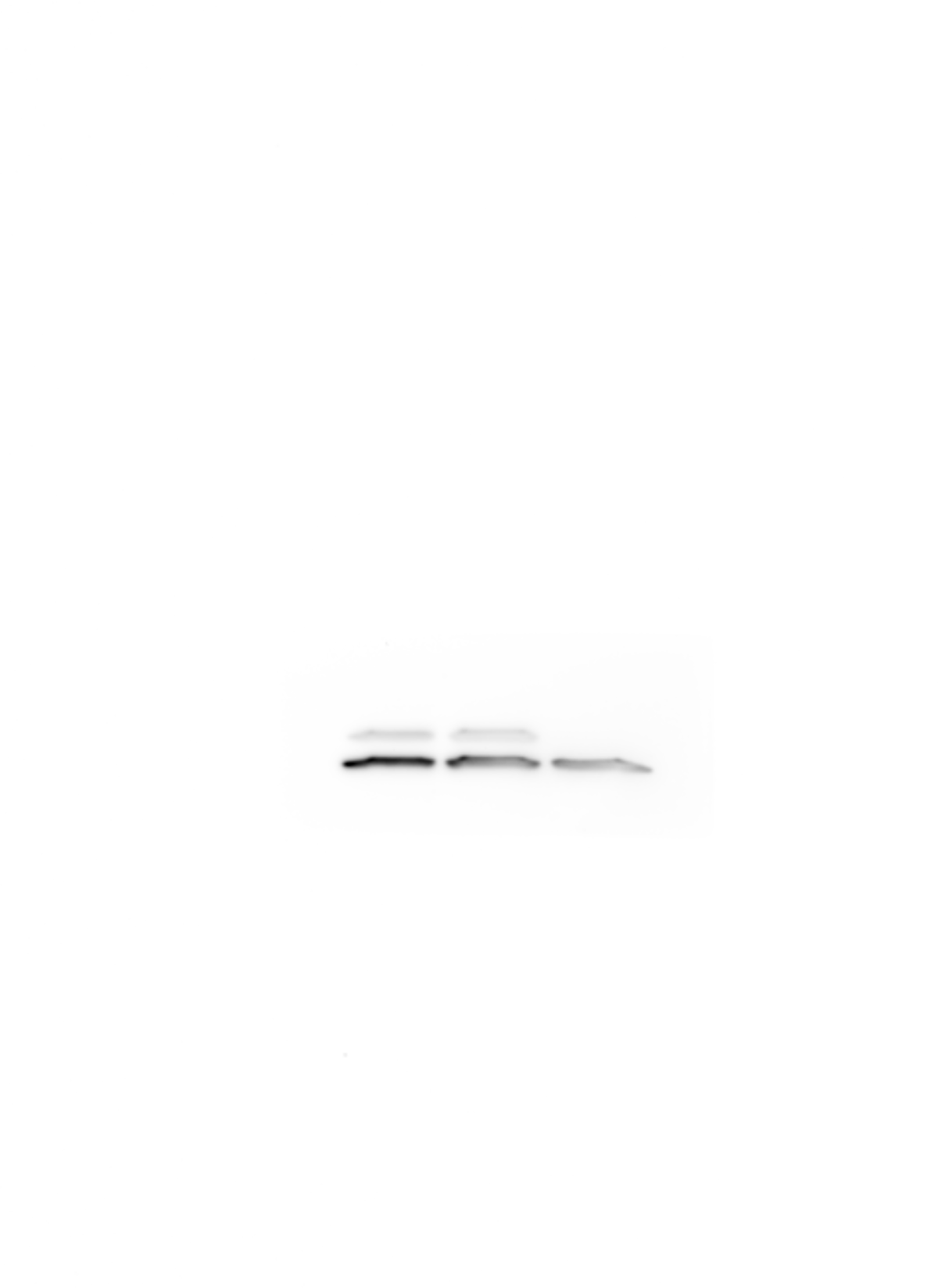

Supplement: Figure 1—source data 1. [file elife-105821-fig1-data1.zip › Figure 1-source data 1/Original files for western blot analysis displayed in Figure 1F/EA3 20231115_113608_Ch/EA3 20231115_113608_Ch_Chemi.tif]

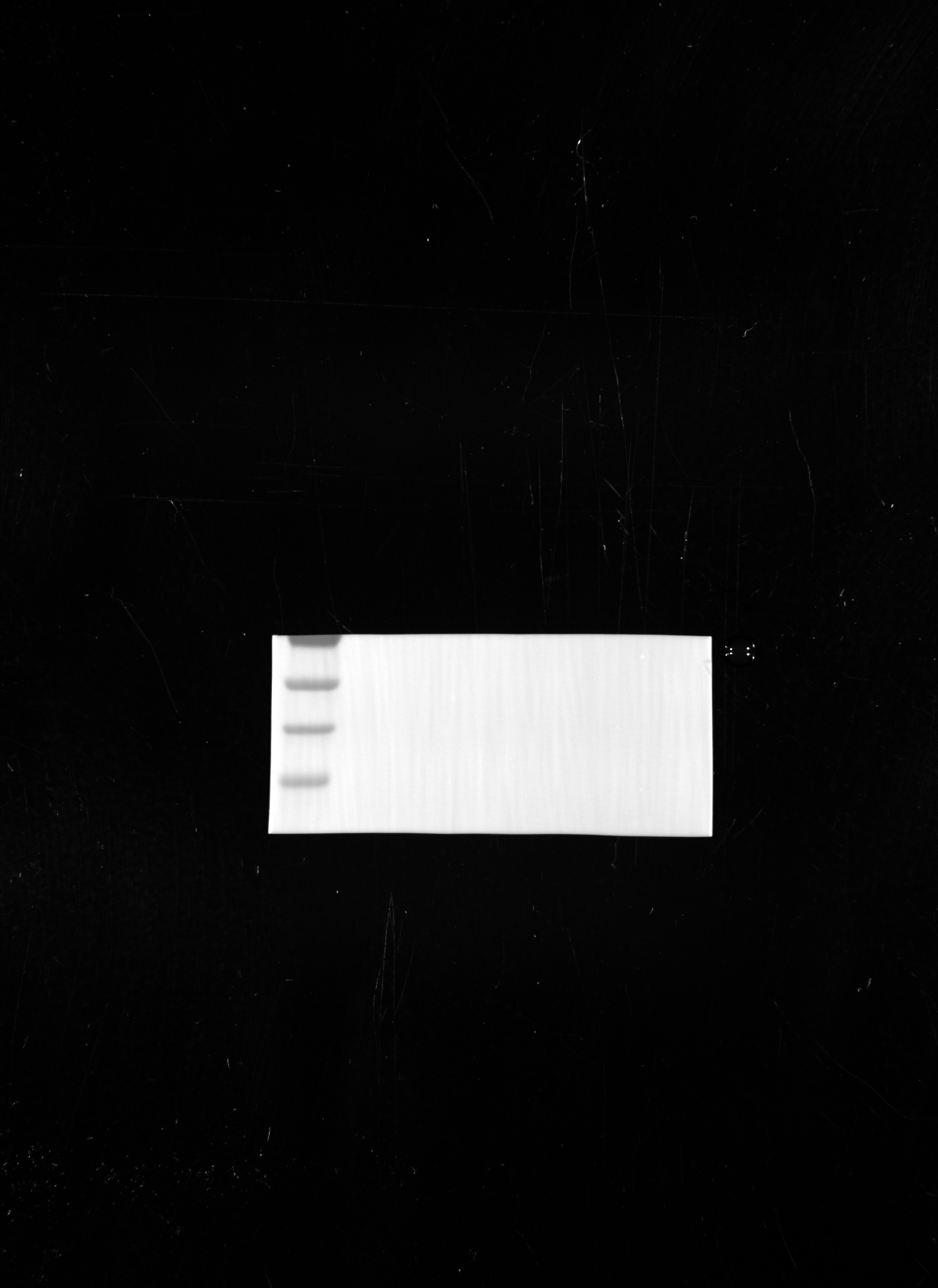

Supplement: Figure 1—source data 1. [file elife-105821-fig1-data1.zip › Figure 1-source data 1/Original files for western blot analysis displayed in Figure 1F/EA3 20231115_113608_Ch/EA3 20231115_113608_Ch-Marker.tif]

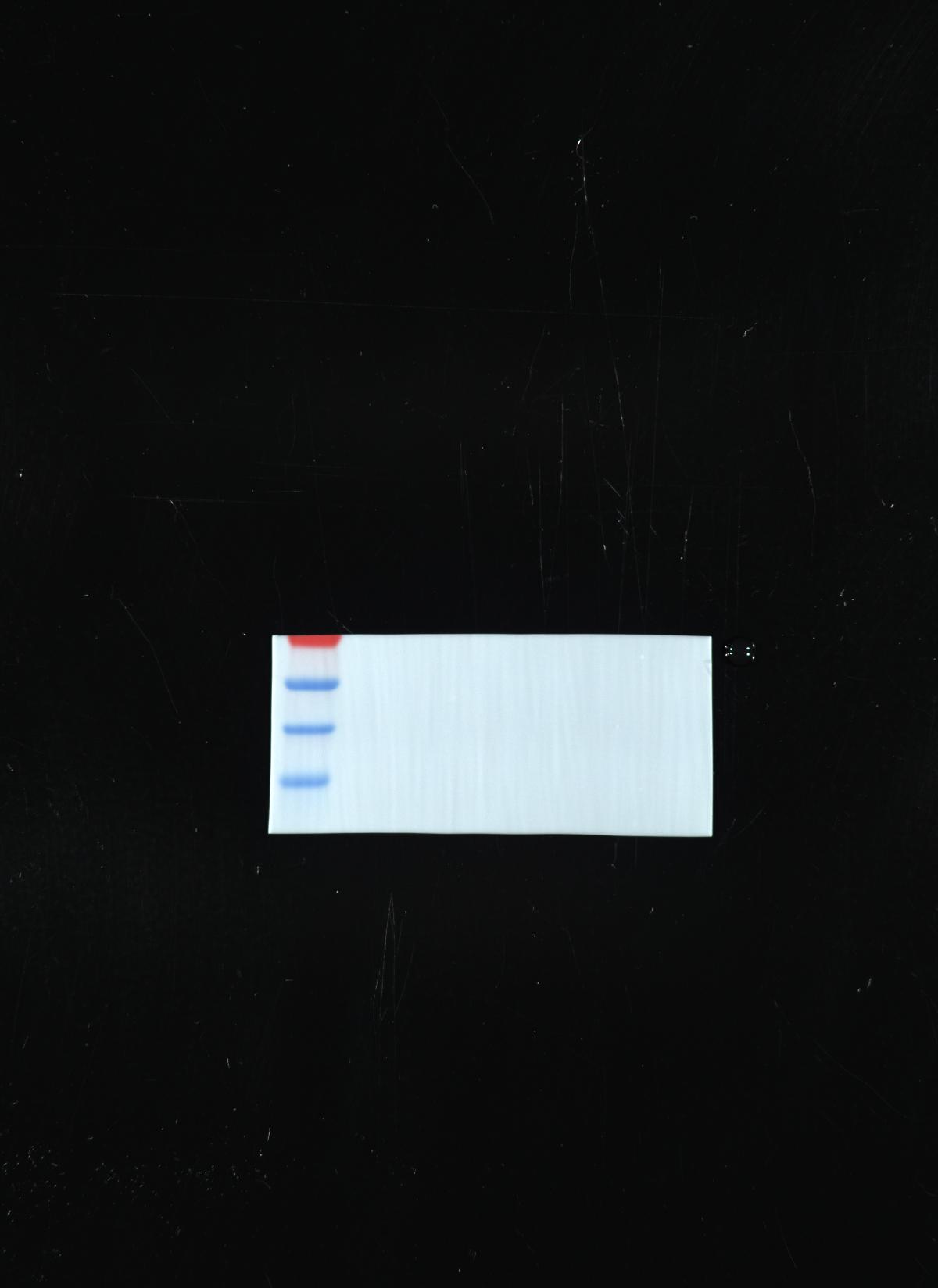

Supplement: Figure 1—source data 1. [file elife-105821-fig1-data1.zip › Figure 1-source data 1/Original files for western blot analysis displayed in Figure 1F/EA3 20231115_113608_Ch/EA3 20231115_113608_Ch-Marker.jpg]

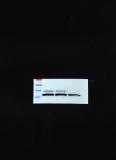

Supplement: Figure 1—source data 1. [file elife-105821-fig1-data1.zip › Figure 1-source data 1/Original files for western blot analysis displayed in Figure 1F/EA3 20231115_113608_Ch/EA3 20231115_113608_Ch_Thumb.jpg]

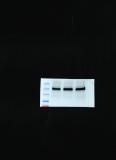

Supplement: Figure 1—source data 1. [file elife-105821-fig1-data1.zip › Figure 1-source data 1/Original files for western blot analysis displayed in Figure 1F/CHC 20231115_113224_Ch/CHC 20231115_113224_Ch_Thumb.jpg]

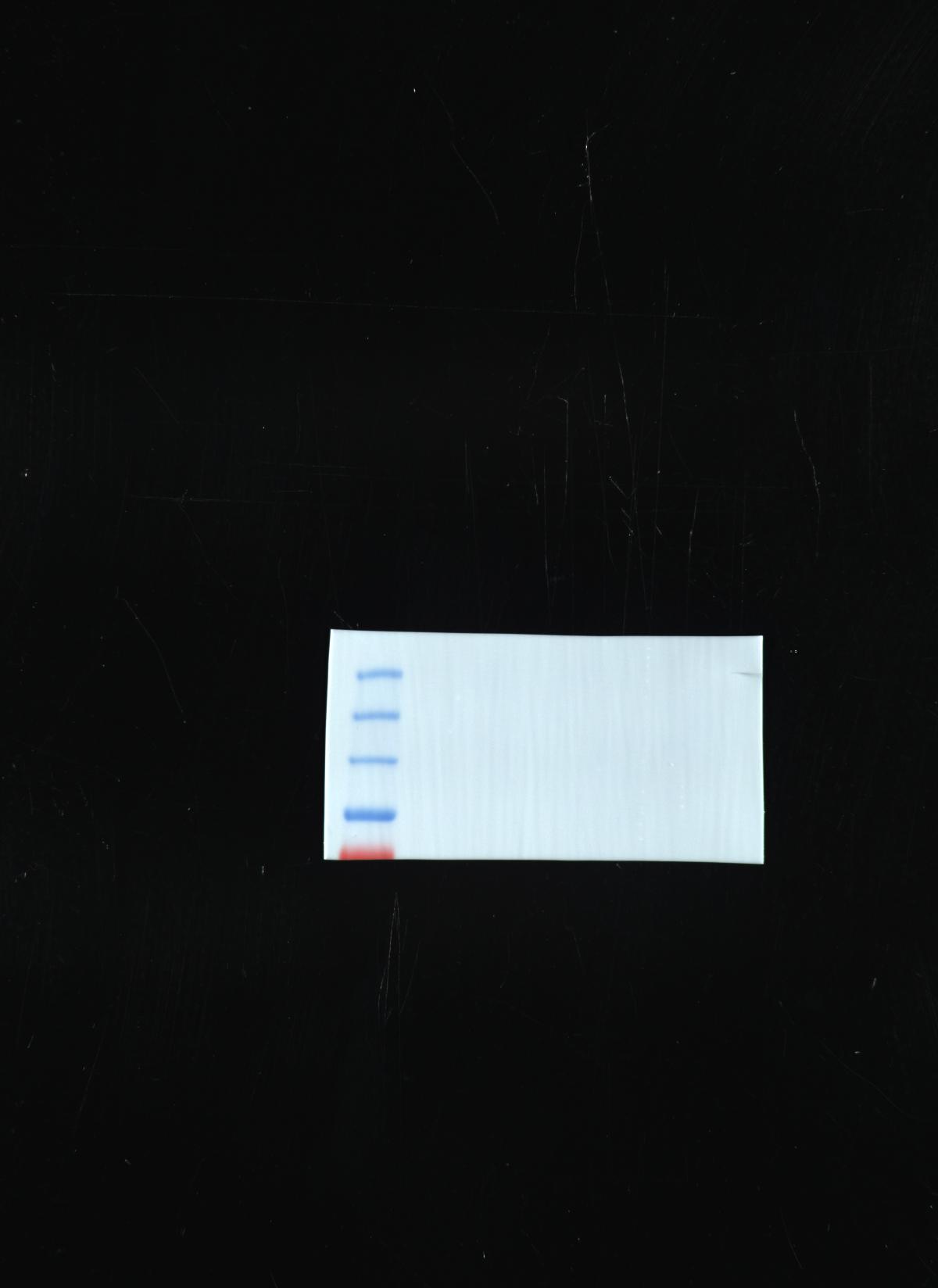

Supplement: Figure 1—source data 1. [file elife-105821-fig1-data1.zip › Figure 1-source data 1/Original files for western blot analysis displayed in Figure 1F/CHC 20231115_113224_Ch/CHC 20231115_113224_Ch-Marker.jpg]

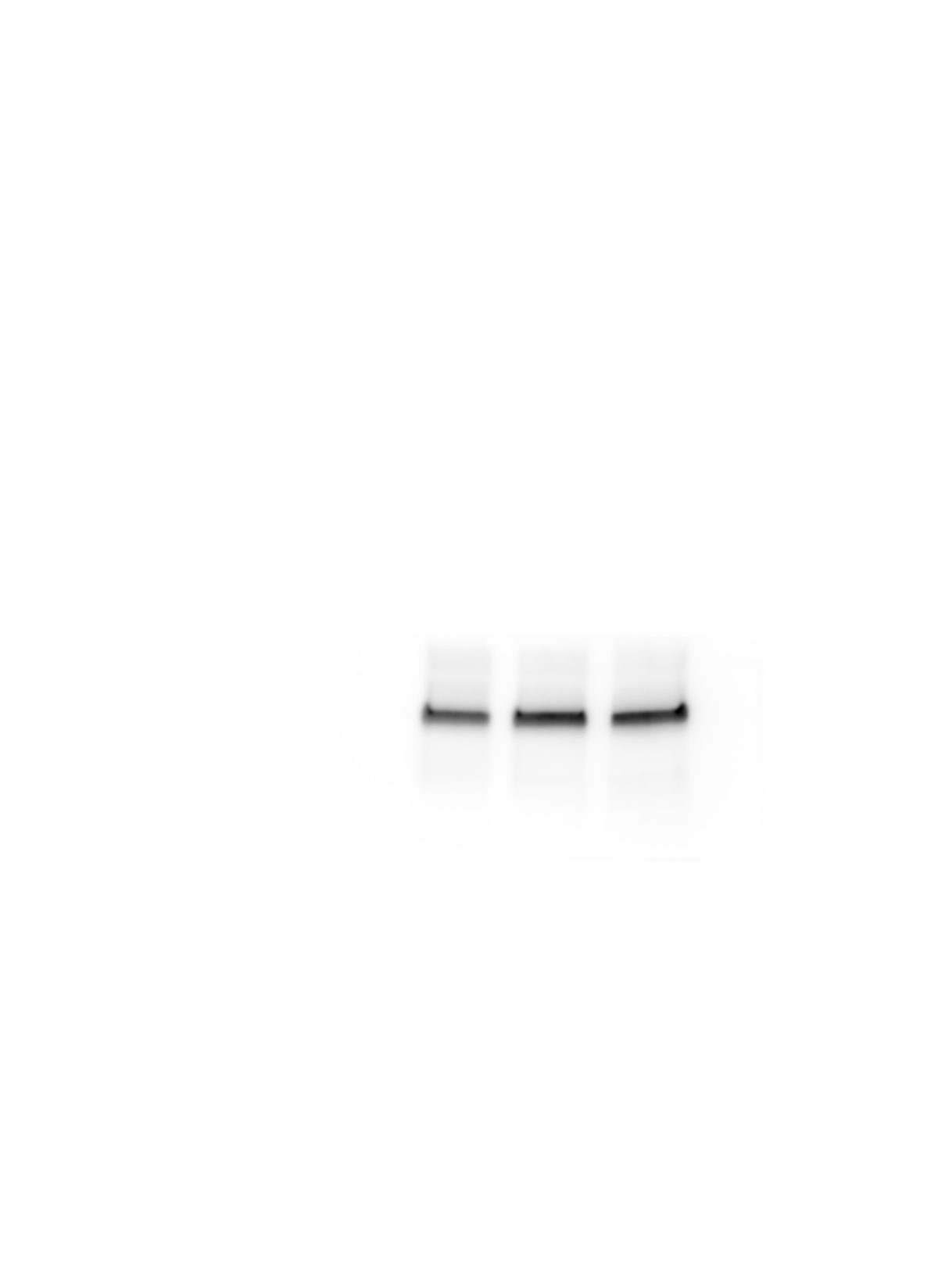

Supplement: Figure 1—source data 1. [file elife-105821-fig1-data1.zip › Figure 1-source data 1/Original files for western blot analysis displayed in Figure 1F/CHC 20231115_113224_Ch/CHC 20231115_113224_Ch_Chemi.tif]

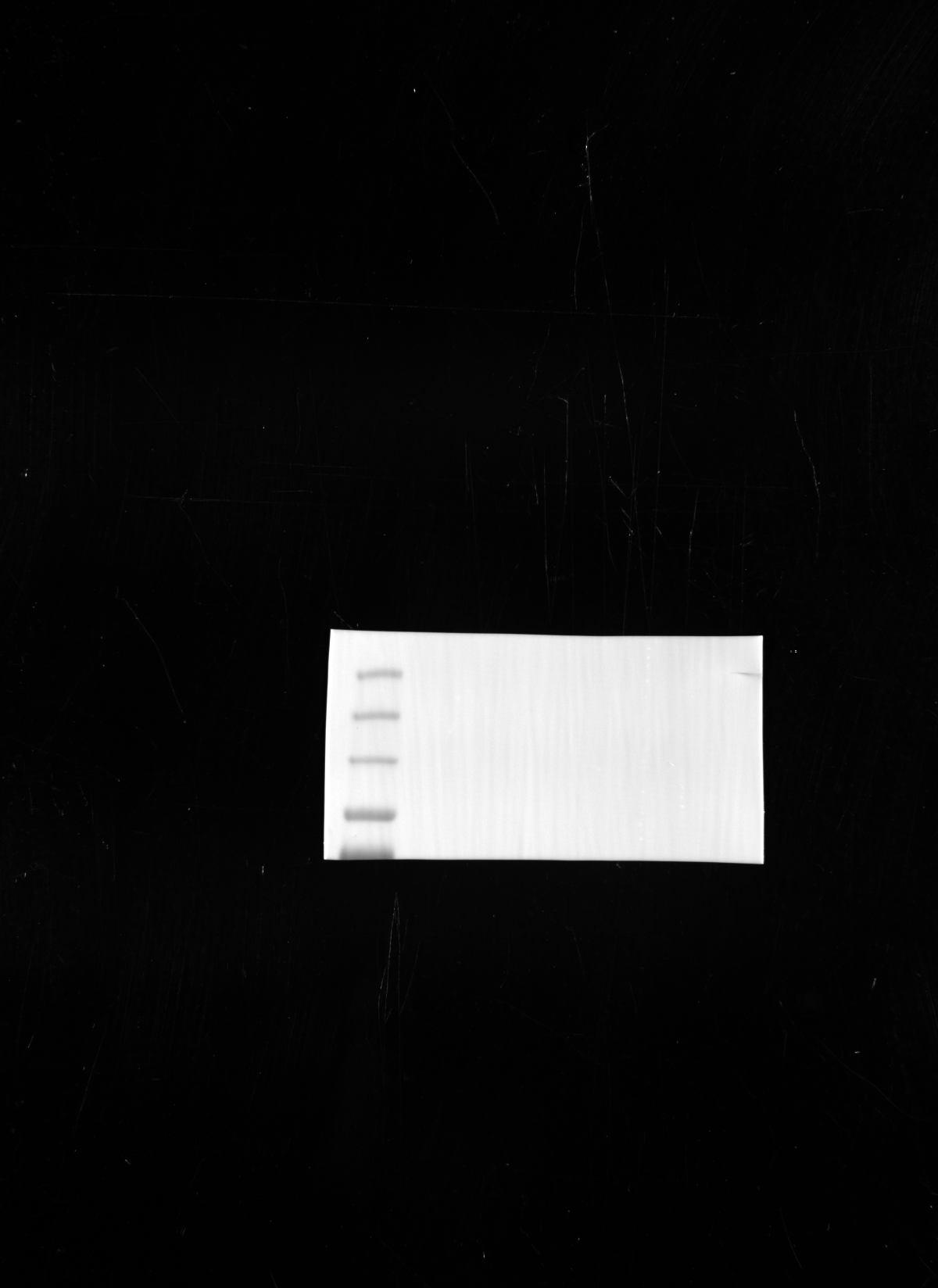

Supplement: Figure 1—source data 1. [file elife-105821-fig1-data1.zip › Figure 1-source data 1/Original files for western blot analysis displayed in Figure 1F/CHC 20231115_113224_Ch/CHC 20231115_113224_Ch-Marker.tif]

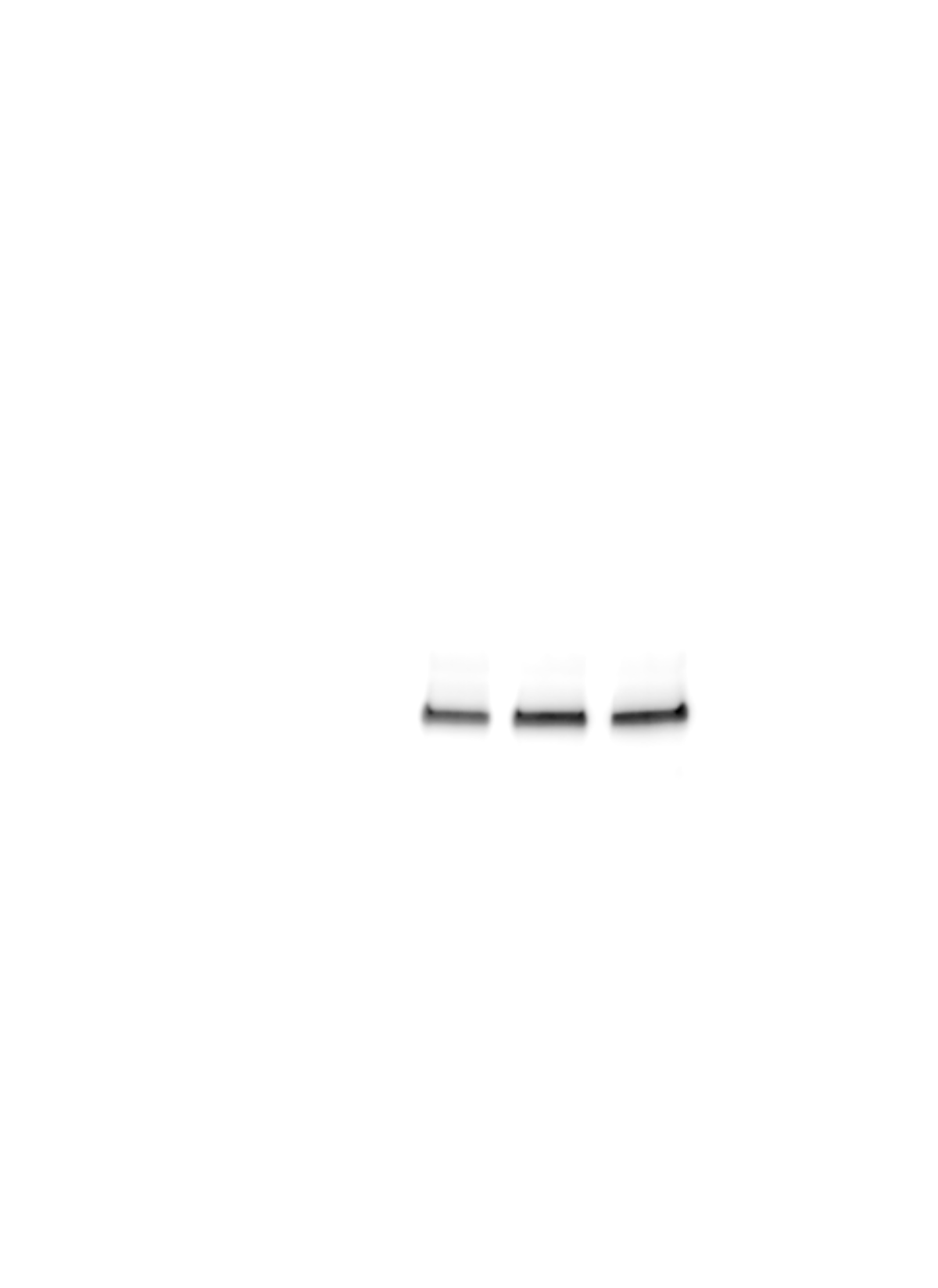

Supplement: Figure 1—source data 1. [file elife-105821-fig1-data1.zip › Figure 1-source data 1/Original files for western blot analysis displayed in Figure 1F/CHC 20231115_113224_Ch/CHC 20231115_113224_Ch_Chemi-1.tif]

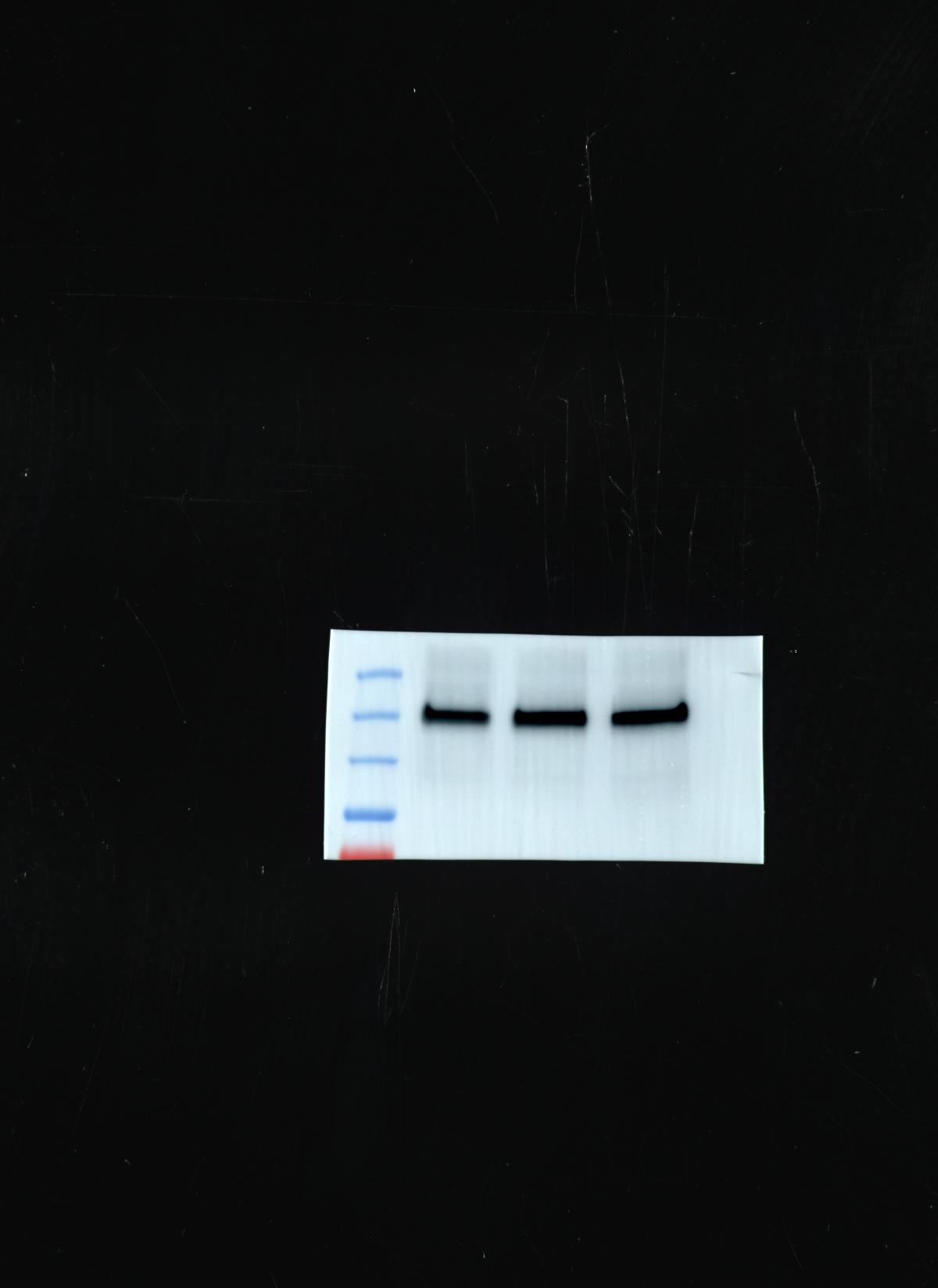

Supplement: Figure 1—source data 1. [file elife-105821-fig1-data1.zip › Figure 1-source data 1/Original files for western blot analysis displayed in Figure 1F/CHC 20231115_113224_Ch/CHC 20231115_113224_Ch_Chemi+Marker.jpg]

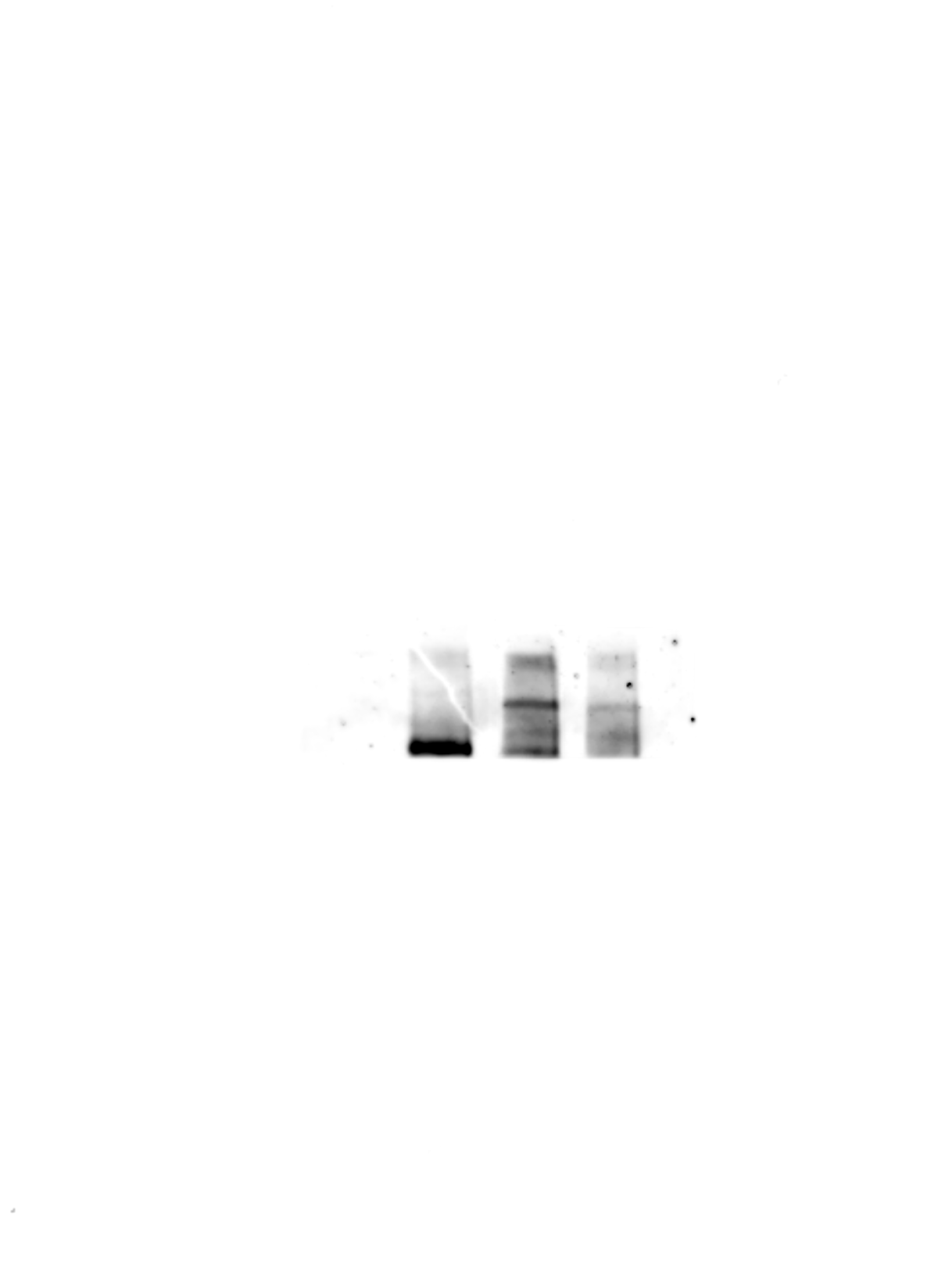

Supplement: Figure 1—source data 1. [file elife-105821-fig1-data1.zip › Figure 1-source data 1/Original files for western blot analysis displayed in Figure 1F/SNAP 20231115_114228_Ch/SNAP 20231115_114228_Ch_Chemi-1.tif]

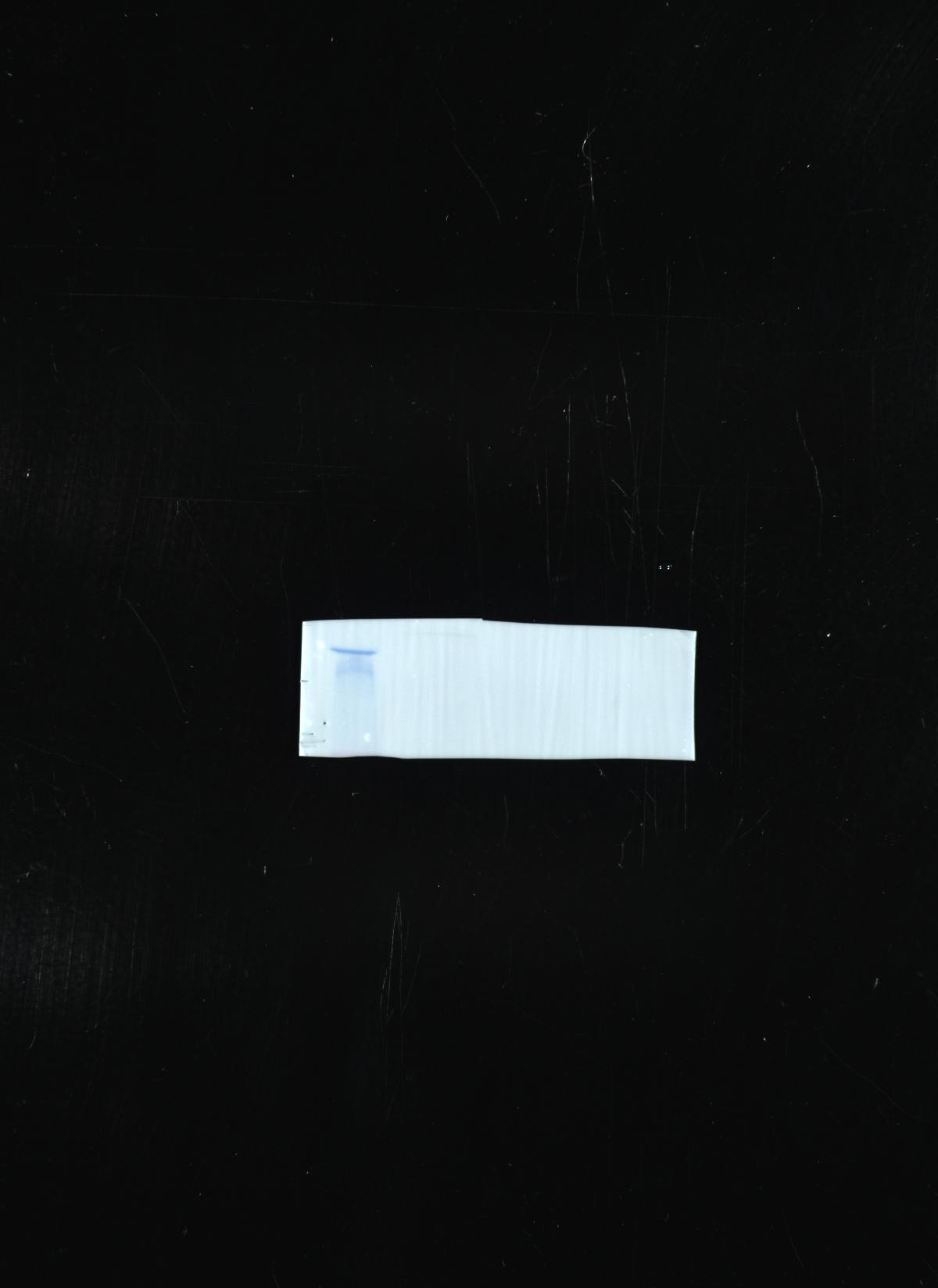

Supplement: Figure 1—source data 1. [file elife-105821-fig1-data1.zip › Figure 1-source data 1/Original files for western blot analysis displayed in Figure 1F/SNAP 20231115_114228_Ch/SNAP 20231115_114228_Ch-Marker.jpg]

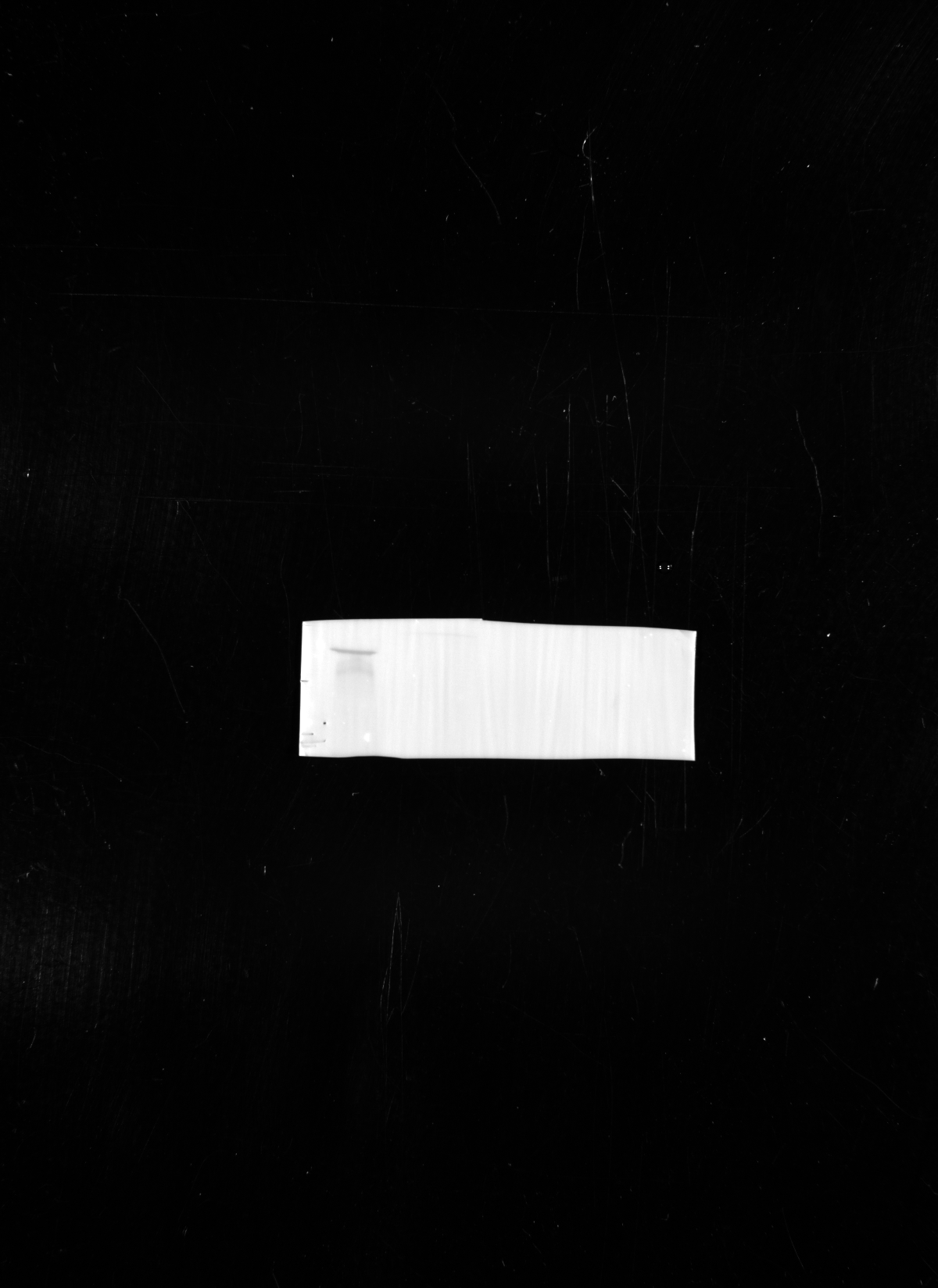

Supplement: Figure 1—source data 1. [file elife-105821-fig1-data1.zip › Figure 1-source data 1/Original files for western blot analysis displayed in Figure 1F/SNAP 20231115_114228_Ch/SNAP 20231115_114228_Ch-Marker.tif]

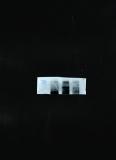

Supplement: Figure 1—source data 1. [file elife-105821-fig1-data1.zip › Figure 1-source data 1/Original files for western blot analysis displayed in Figure 1F/SNAP 20231115_114228_Ch/SNAP 20231115_114228_Ch_Thumb.jpg]

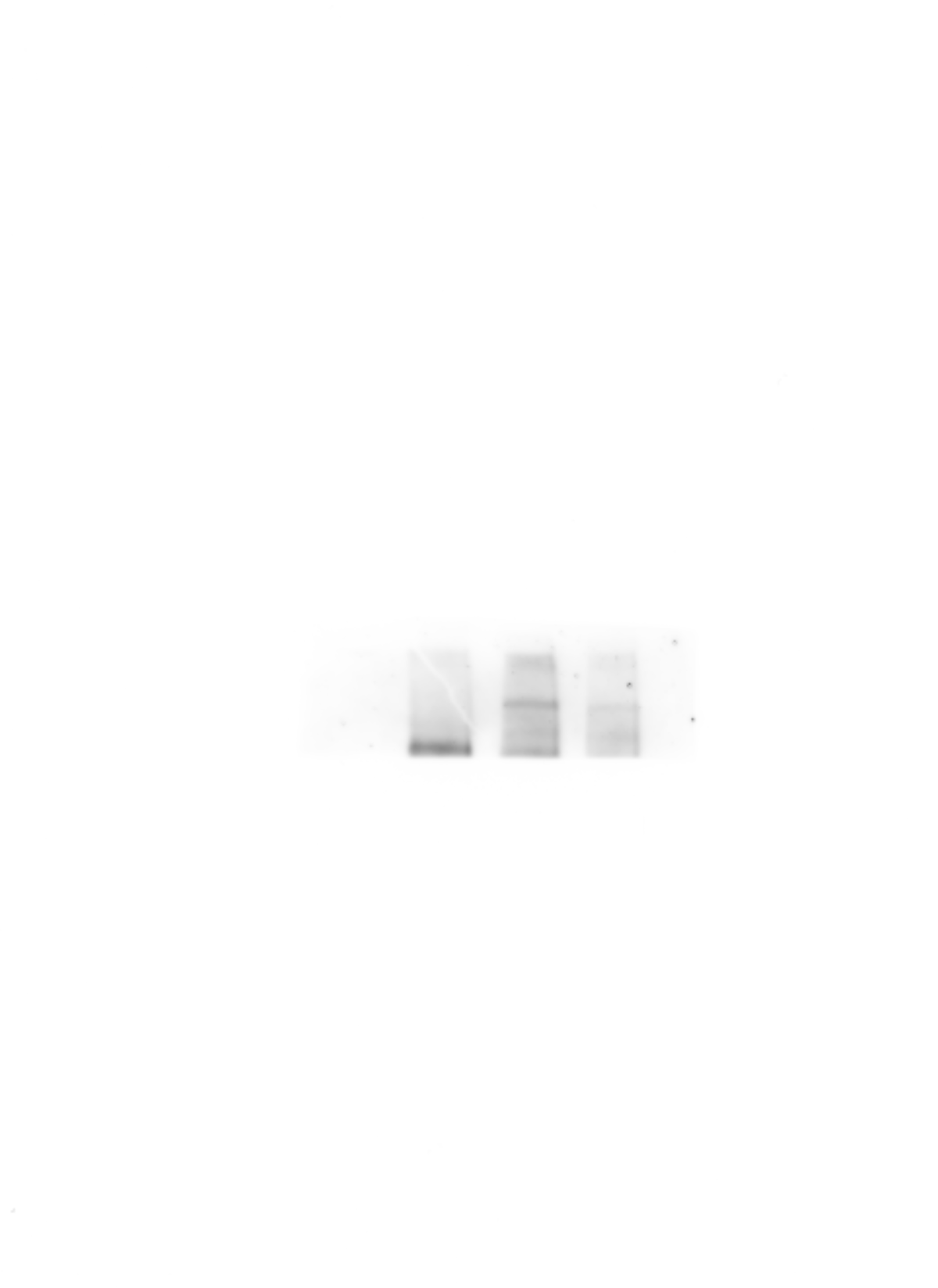

Supplement: Figure 1—source data 1. [file elife-105821-fig1-data1.zip › Figure 1-source data 1/Original files for western blot analysis displayed in Figure 1F/SNAP 20231115_114228_Ch/SNAP 20231115_114228_Ch_Chemi.tif]

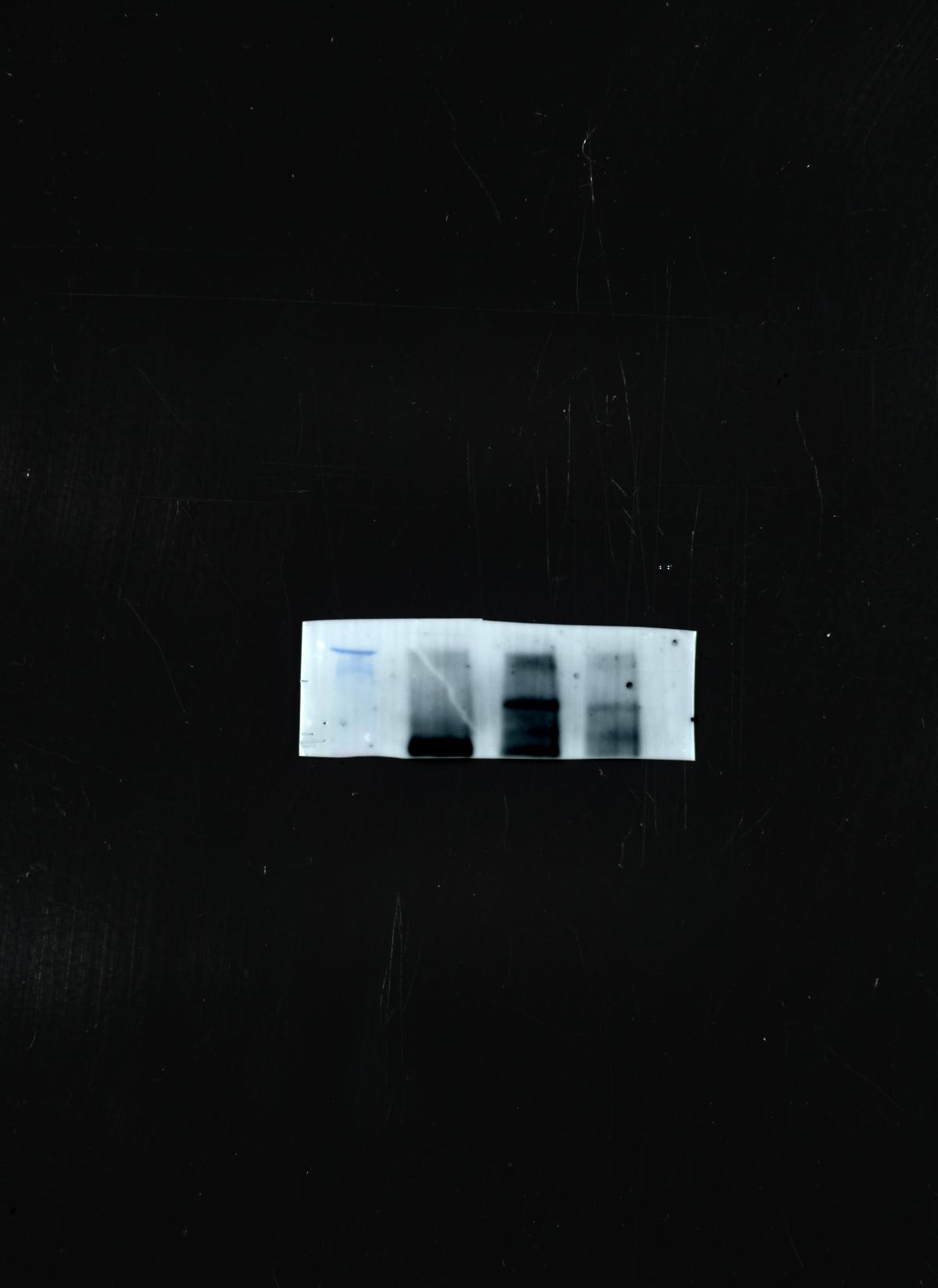

Supplement: Figure 1—source data 1. [file elife-105821-fig1-data1.zip › Figure 1-source data 1/Original files for western blot analysis displayed in Figure 1F/SNAP 20231115_114228_Ch/SNAP 20231115_114228_Ch_Chemi+Marker.jpg]

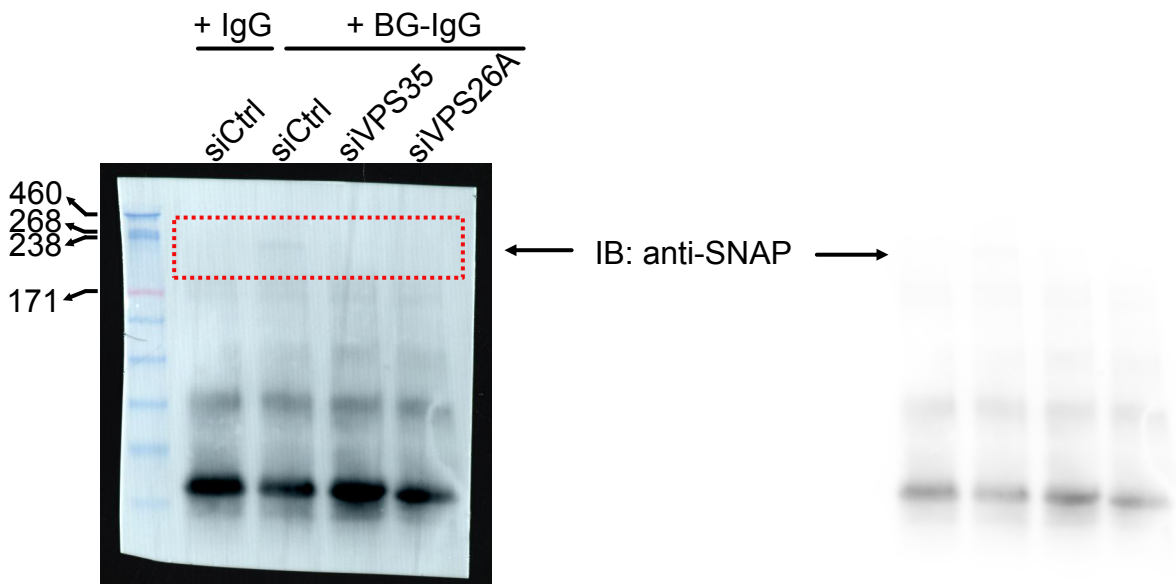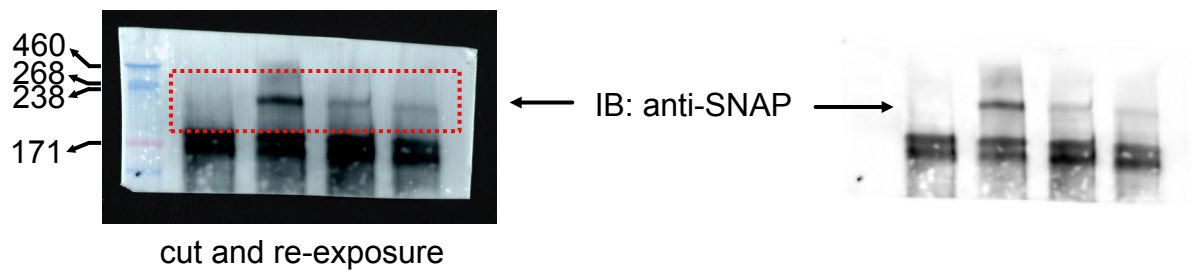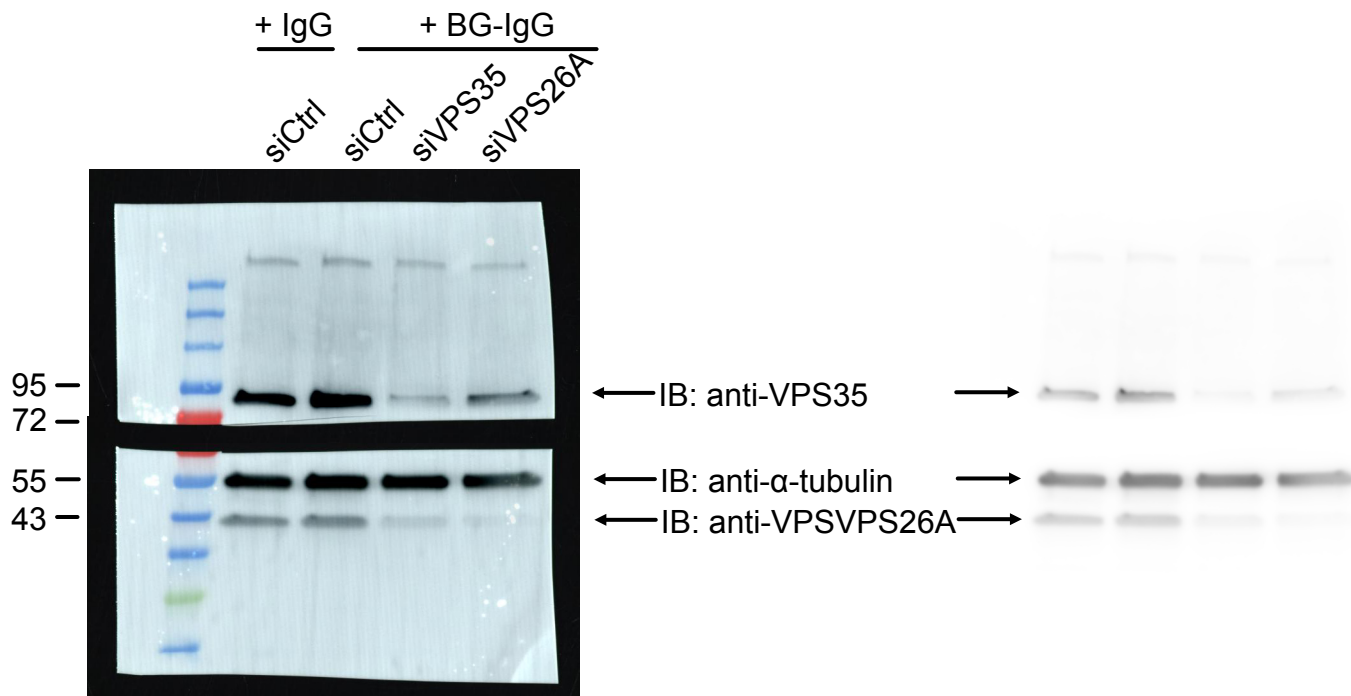

Supplement: Figure 1—source data 2. [file elife-105821-fig1-data2.zip › Figure 1-source data 2/PDF file containing original western blots for Figure 1C.pdf]

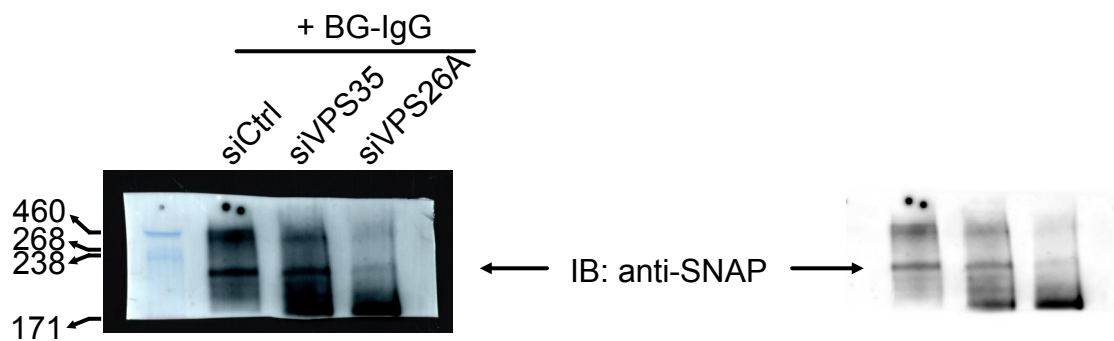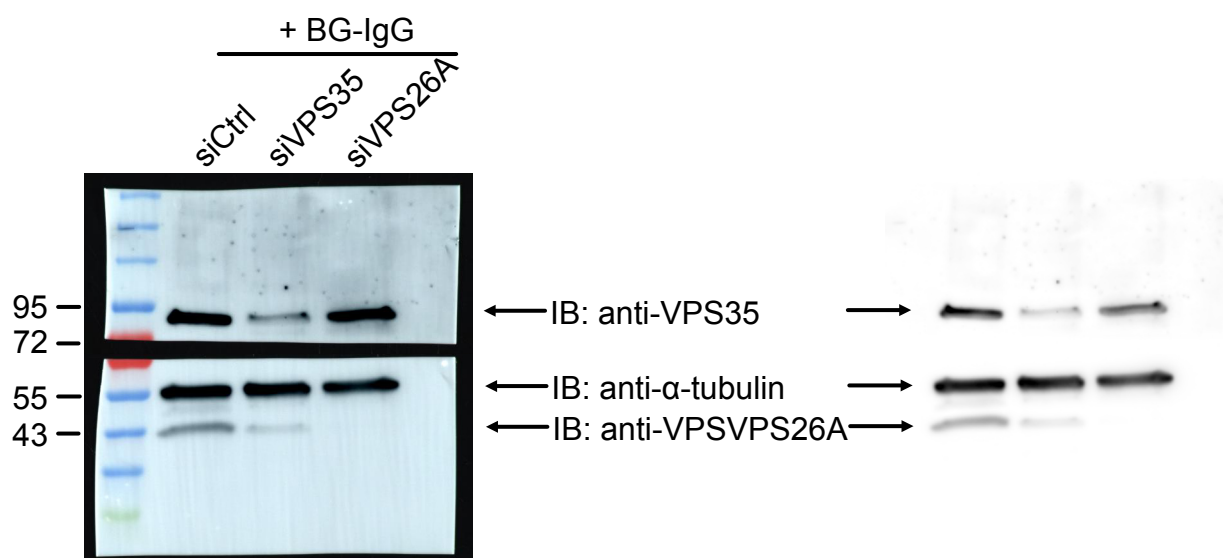

Supplement: Figure 1—source data 2. [file elife-105821-fig1-data2.zip › Figure 1-source data 2/PDF file containing original western blots for Figure 1D.pdf]

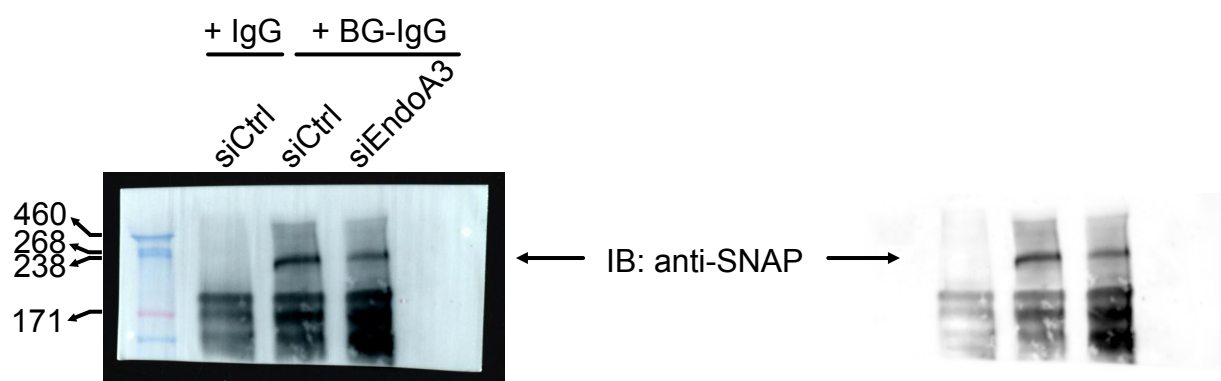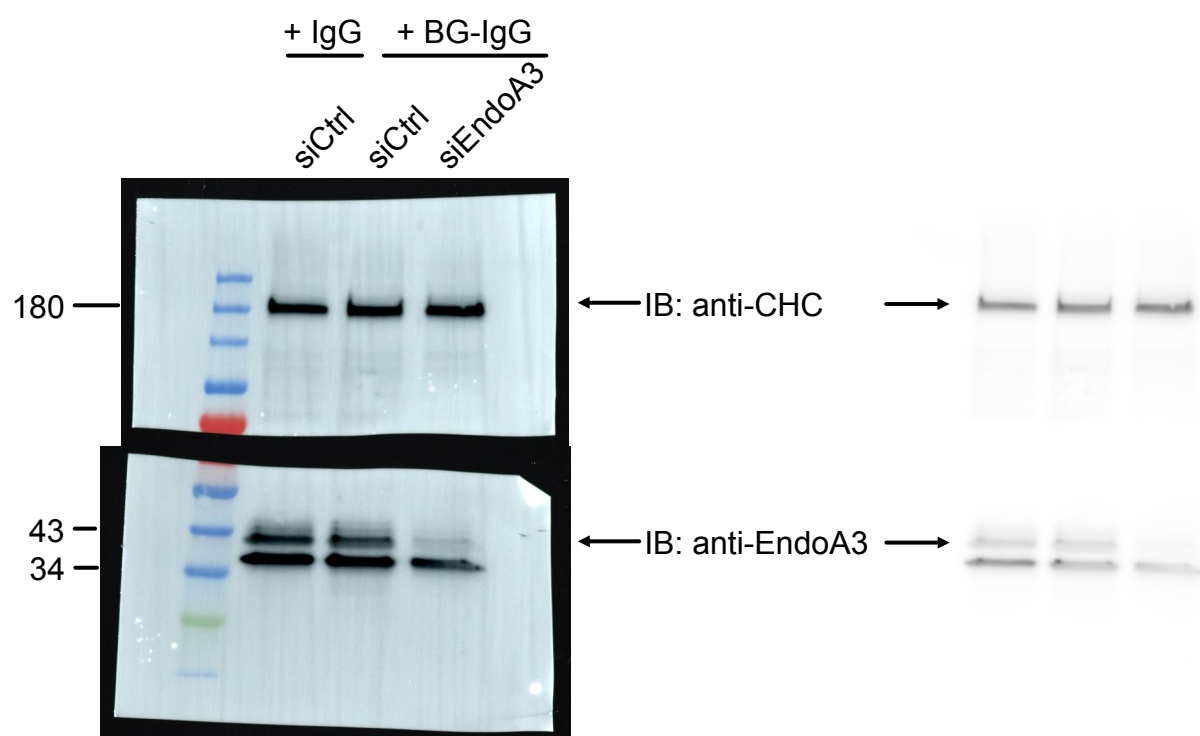

Supplement: Figure 1—source data 2. [file elife-105821-fig1-data2.zip › Figure 1-source data 2/PDF file containing original western blots for Figure 1E.pdf]

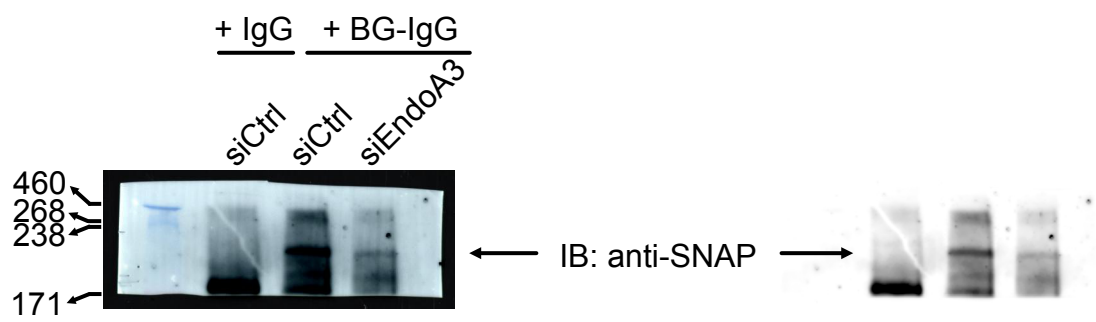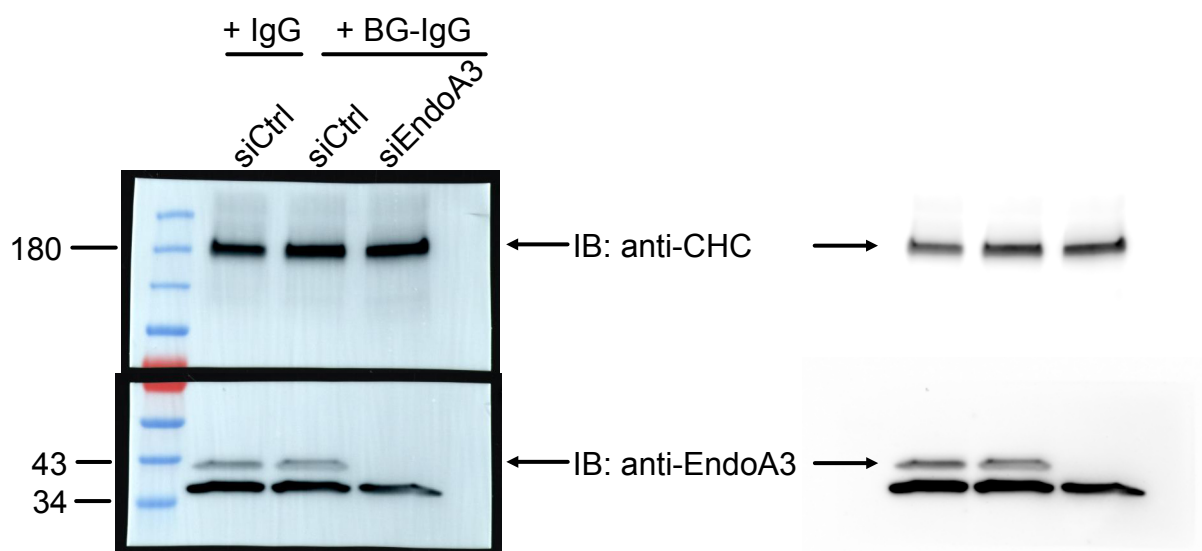

Supplement: Figure 1—source data 2. [file elife-105821-fig1-data2.zip › Figure 1-source data 2/PDF file containing original western blots for Figure 1F.pdf]

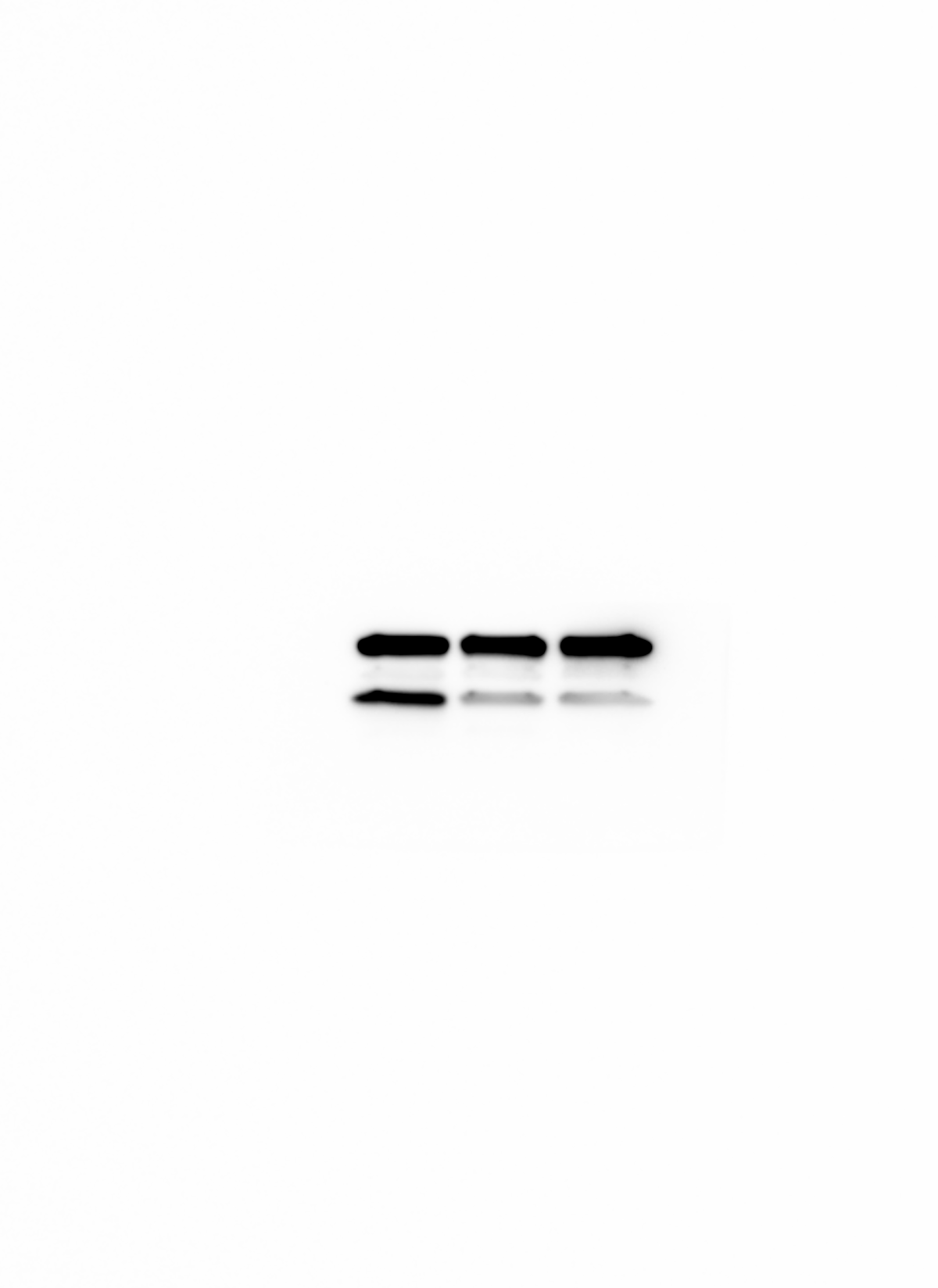

Supplement: Figure 1—figure supplement 1—source data 1. [file elife-105821-fig1-figsupp1-data1.zip › Figure 1-figure supplement 1-source data 1/Original files for western blot analysis displayed in Figure 1-figure supplement 1D/Tubulin Vps26 Dauv 20231122_114931_Ch/Vps26 Dauv 20231122_114931_Ch_Chemi-1.tif]

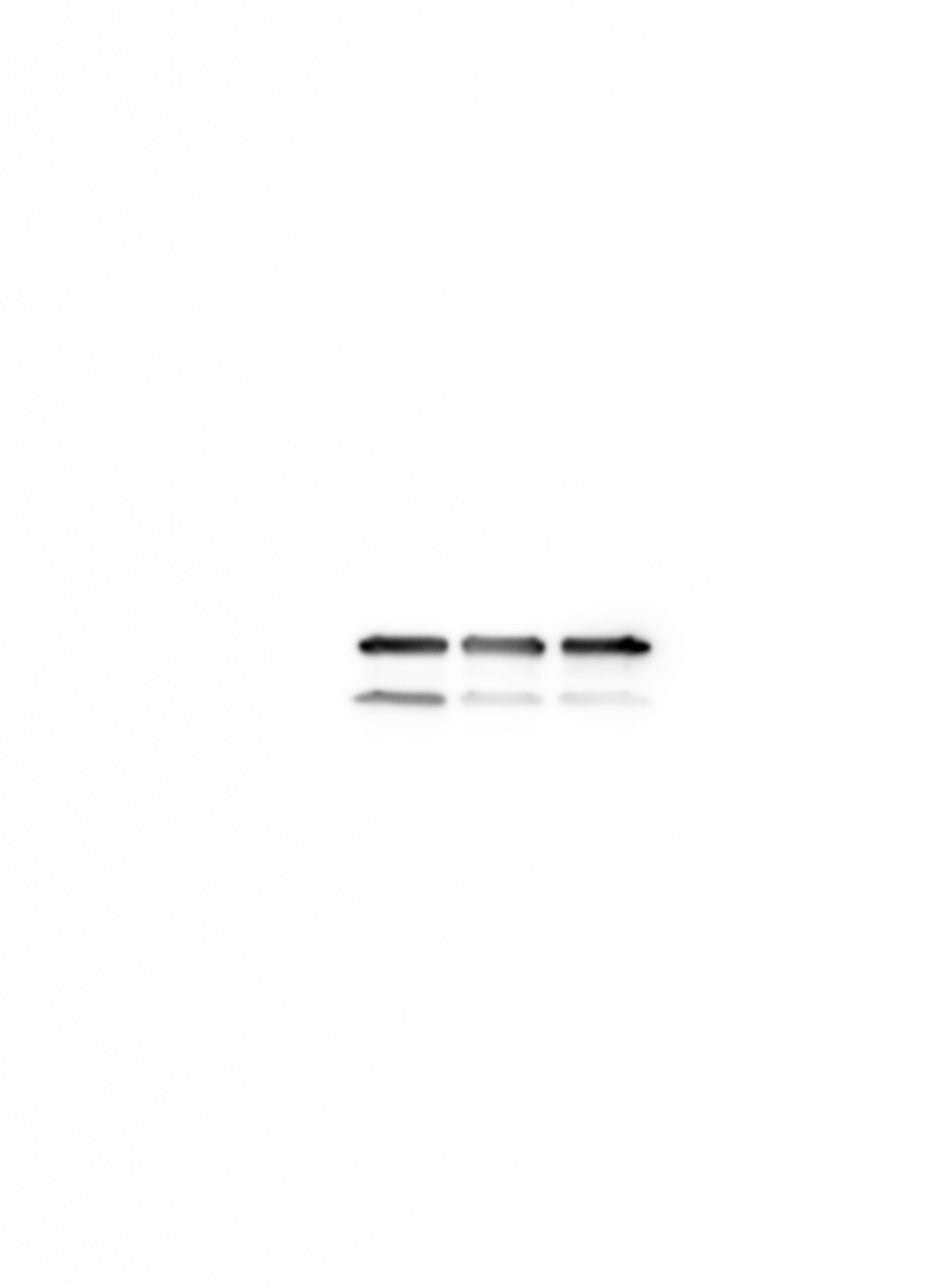

Supplement: Figure 1—figure supplement 1—source data 1. [file elife-105821-fig1-figsupp1-data1.zip › Figure 1-figure supplement 1-source data 1/Original files for western blot analysis displayed in Figure 1-figure supplement 1D/Tubulin Vps26 Dauv 20231122_114931_Ch/Tubulin Vps26 Dauv 20231122_114931_Ch_Chemi-1.tif]

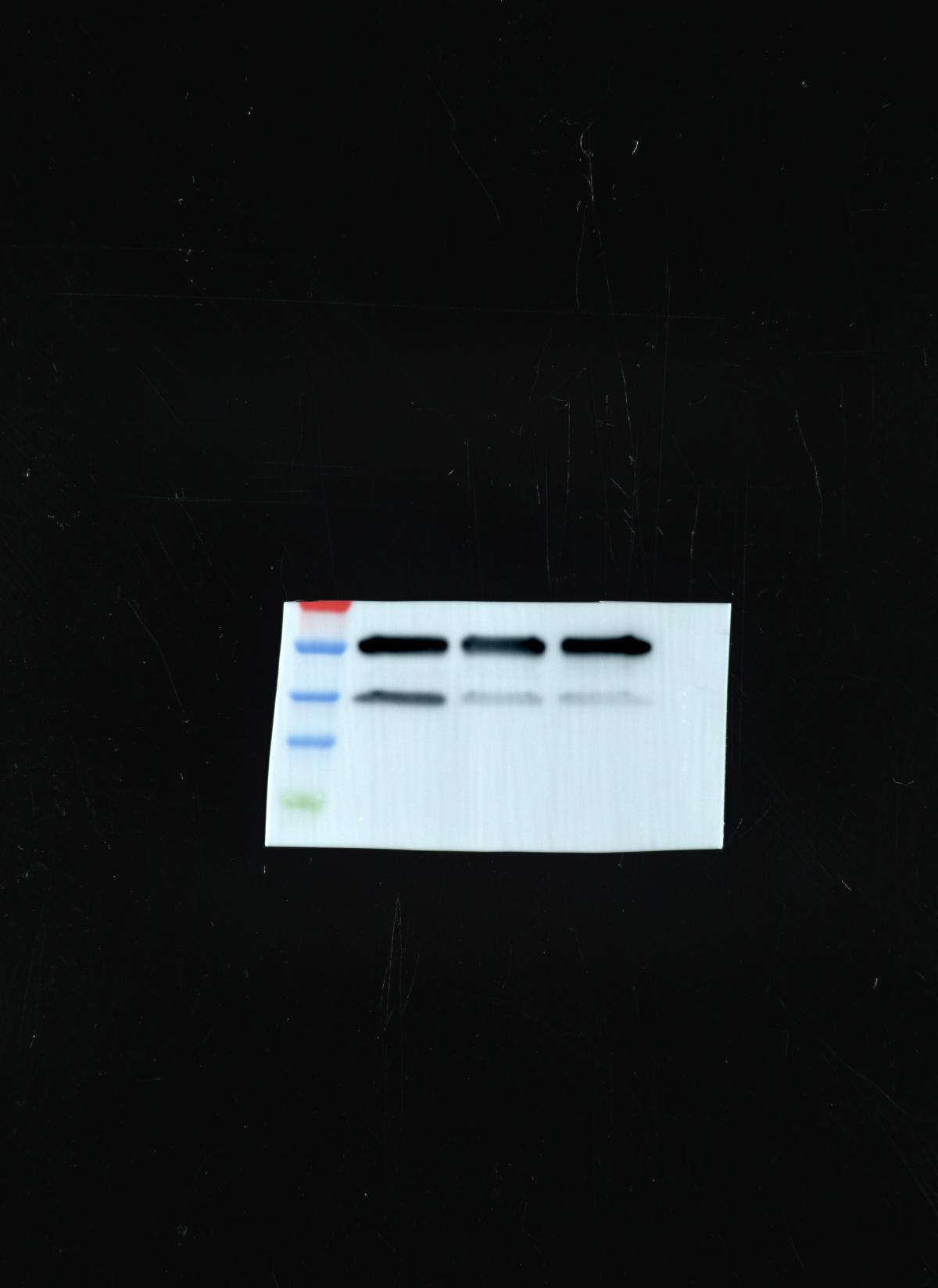

Supplement: Figure 1—figure supplement 1—source data 1. [file elife-105821-fig1-figsupp1-data1.zip › Figure 1-figure supplement 1-source data 1/Original files for western blot analysis displayed in Figure 1-figure supplement 1D/Tubulin Vps26 Dauv 20231122_114931_Ch/Tubulin Vps26 Dauv 20231122_114931_Ch_Chemi+Marker.jpg]

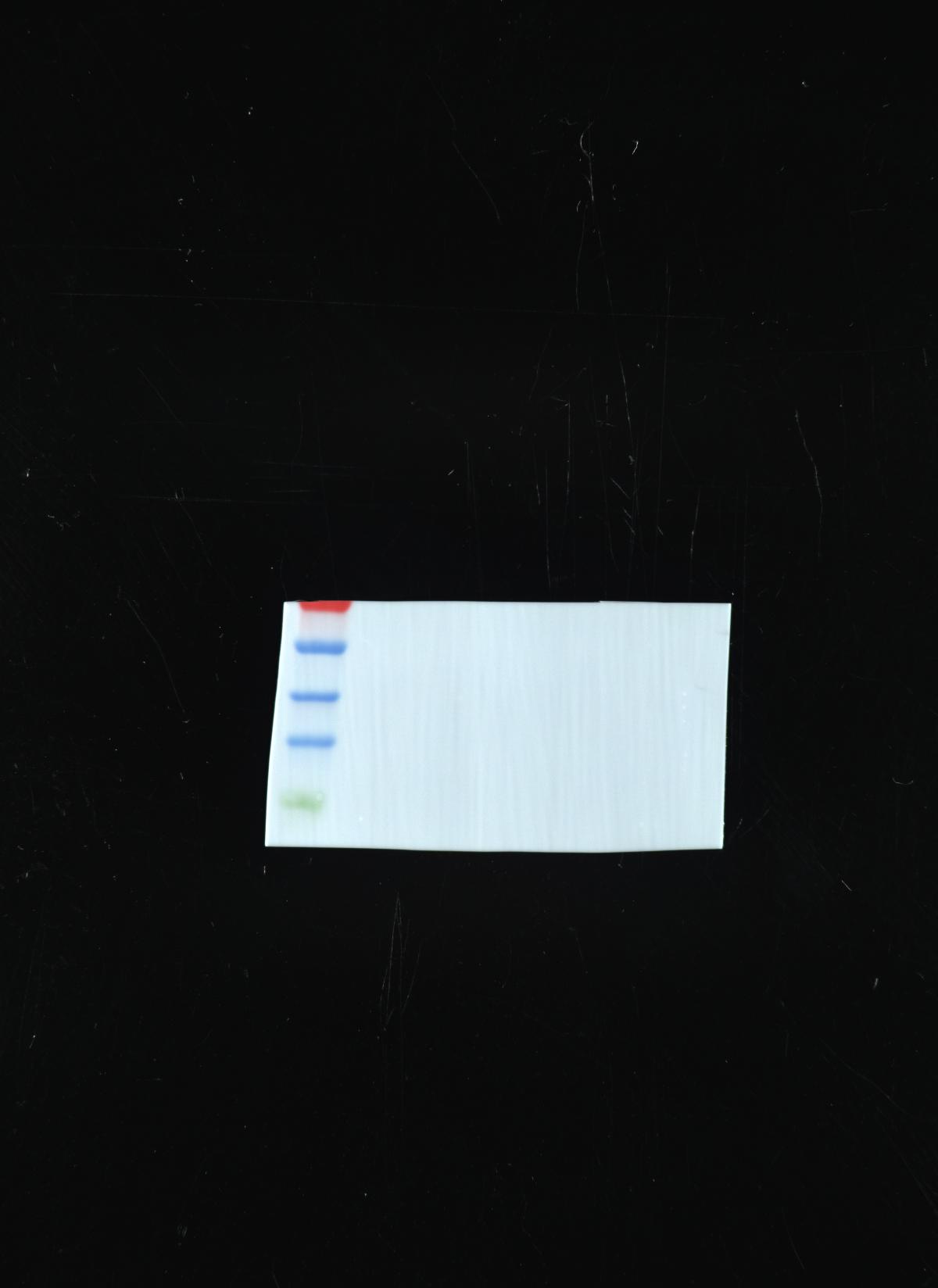

Supplement: Figure 1—figure supplement 1—source data 1. [file elife-105821-fig1-figsupp1-data1.zip › Figure 1-figure supplement 1-source data 1/Original files for western blot analysis displayed in Figure 1-figure supplement 1D/Tubulin Vps26 Dauv 20231122_114931_Ch/Tubulin Vps26 Dauv 20231122_114931_Ch-Marker.jpg]

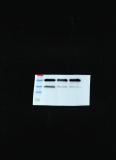

Supplement: Figure 1—figure supplement 1—source data 1. [file elife-105821-fig1-figsupp1-data1.zip › Figure 1-figure supplement 1-source data 1/Original files for western blot analysis displayed in Figure 1-figure supplement 1D/Tubulin Vps26 Dauv 20231122_114931_Ch/Tubulin Vps26 Dauv 20231122_114931_Ch_Thumb.jpg]

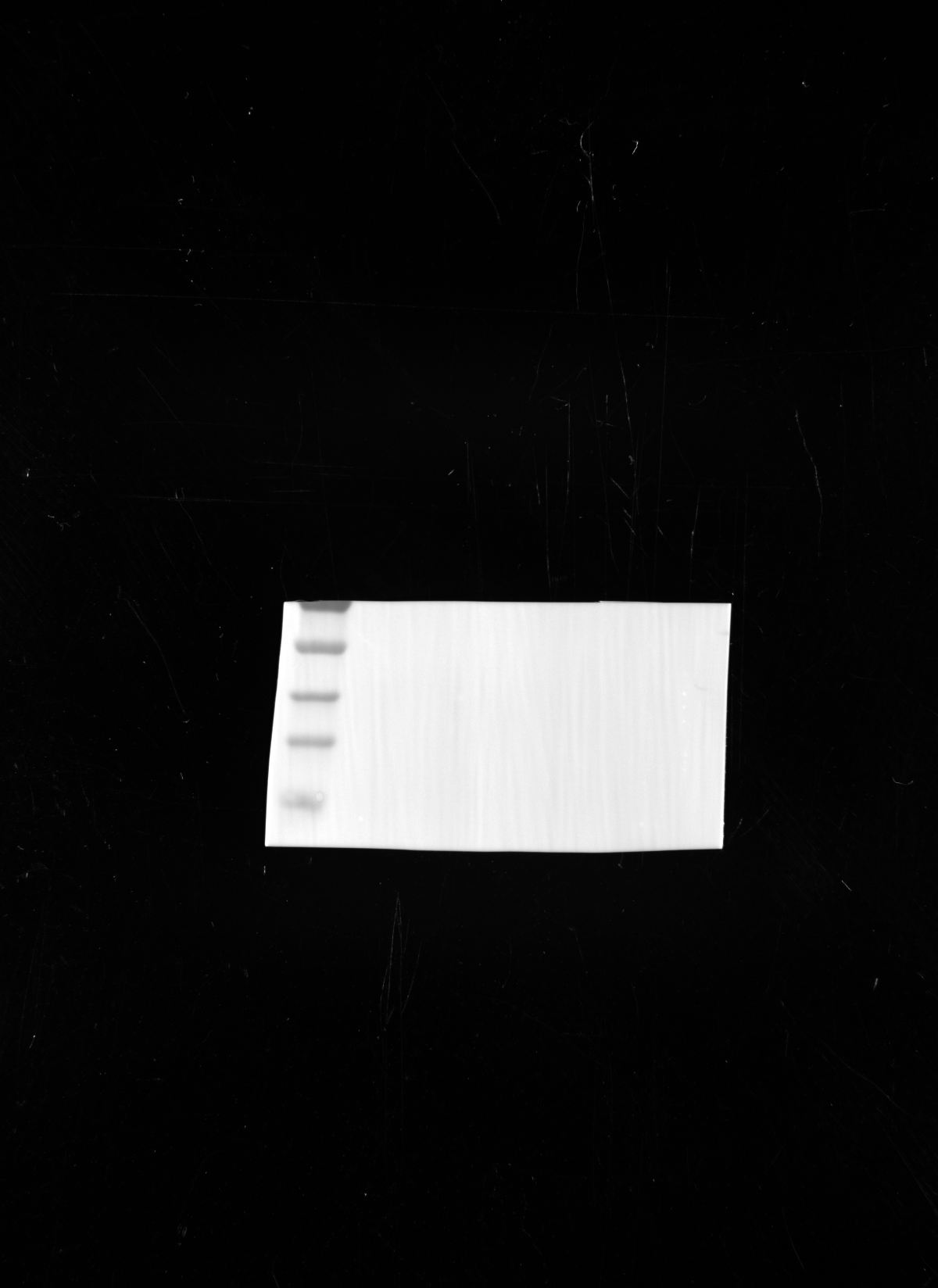

Supplement: Figure 1—figure supplement 1—source data 1. [file elife-105821-fig1-figsupp1-data1.zip › Figure 1-figure supplement 1-source data 1/Original files for western blot analysis displayed in Figure 1-figure supplement 1D/Tubulin Vps26 Dauv 20231122_114931_Ch/Tubulin Vps26 Dauv 20231122_114931_Ch-Marker.tif]

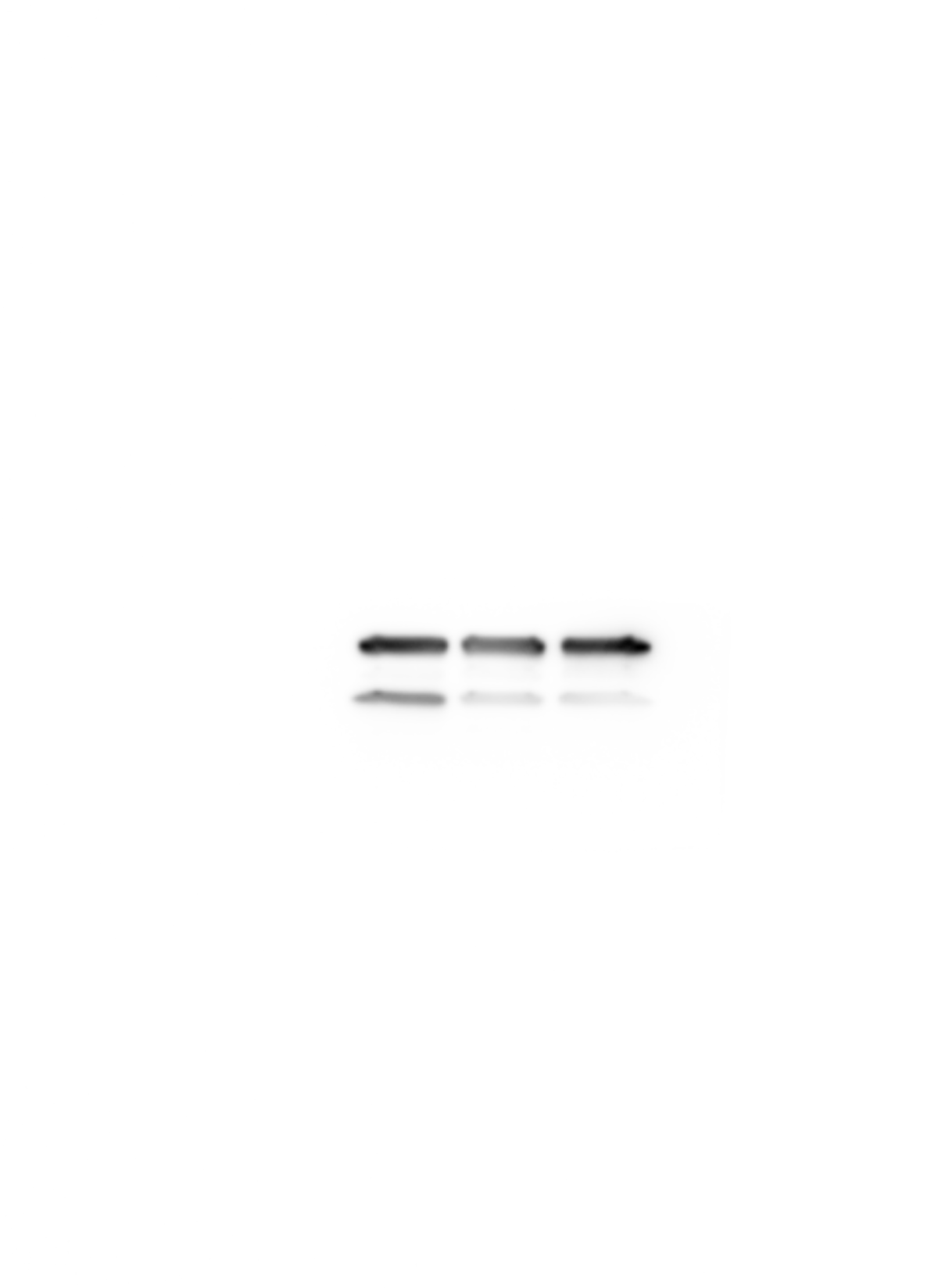

Supplement: Figure 1—figure supplement 1—source data 1. [file elife-105821-fig1-figsupp1-data1.zip › Figure 1-figure supplement 1-source data 1/Original files for western blot analysis displayed in Figure 1-figure supplement 1D/Tubulin Vps26 Dauv 20231122_114931_Ch/Tubulin Vps26 Dauv 20231122_114931_Ch_Chemi.tif]

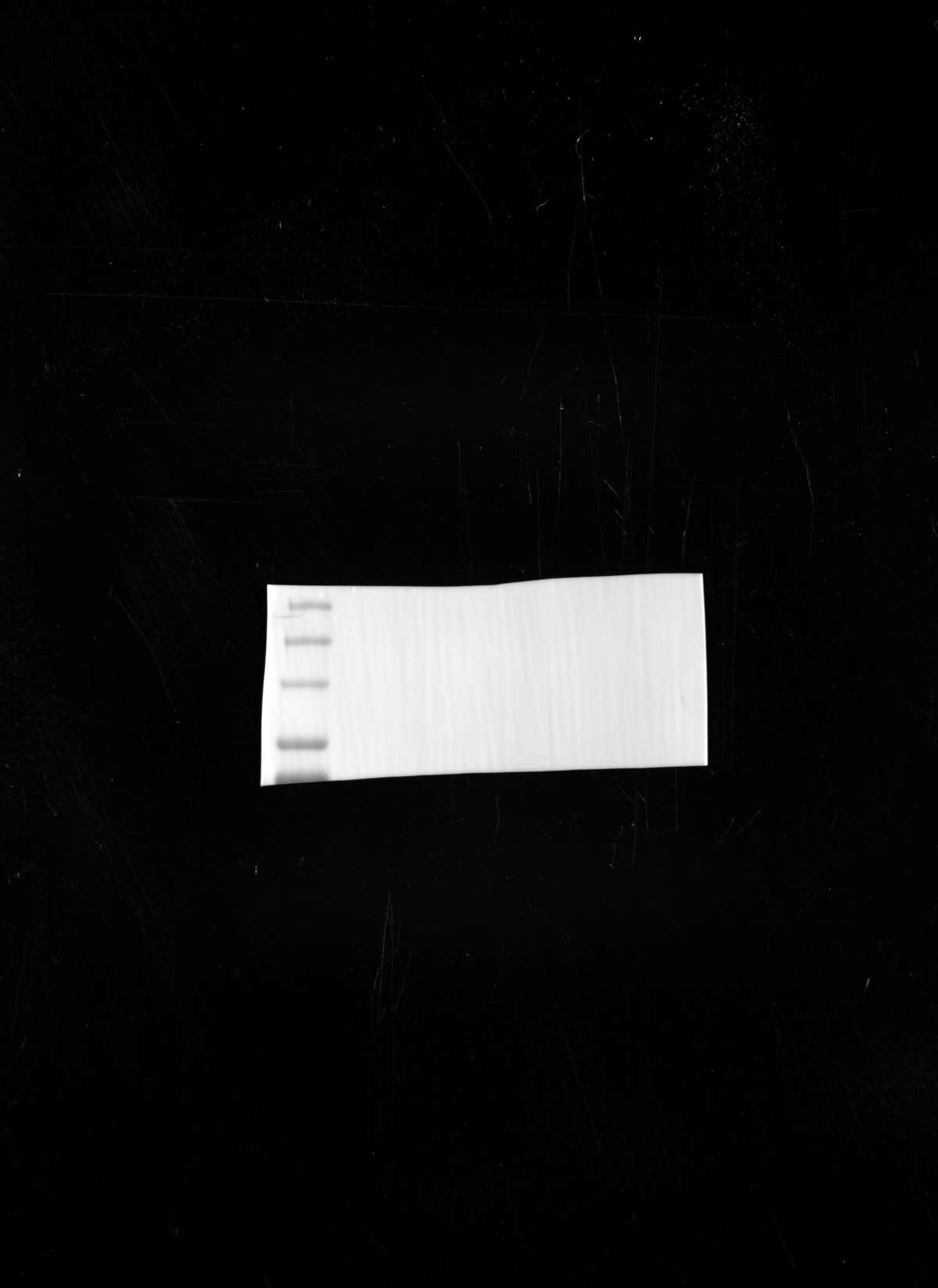

Supplement: Figure 1—figure supplement 1—source data 1. [file elife-105821-fig1-figsupp1-data1.zip › Figure 1-figure supplement 1-source data 1/Original files for western blot analysis displayed in Figure 1-figure supplement 1D/Vps35 Dauv 20231122_121824_Ch/Vps35 Dauv 20231122_121824_Ch-Marker.tif]

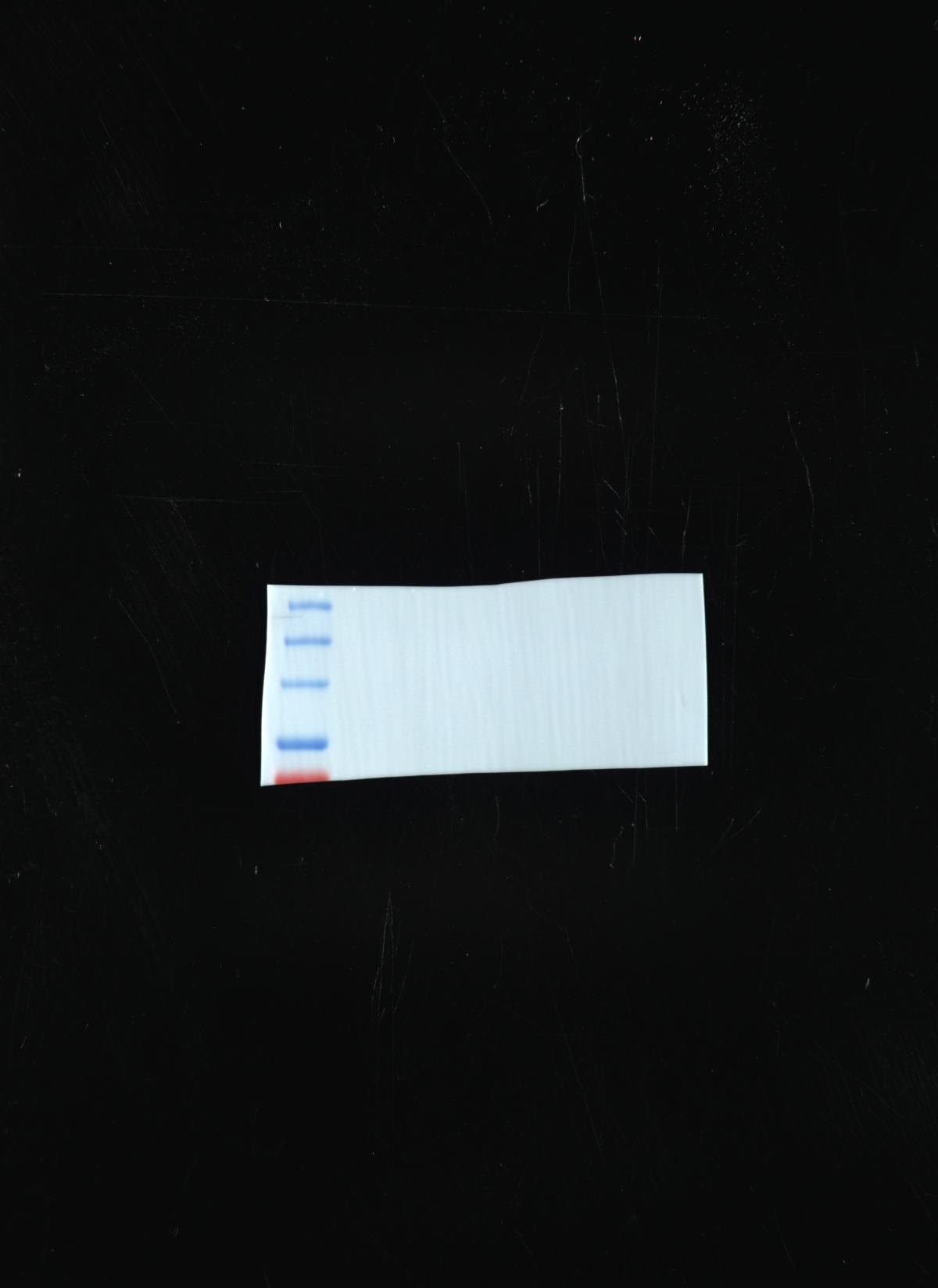

Supplement: Figure 1—figure supplement 1—source data 1. [file elife-105821-fig1-figsupp1-data1.zip › Figure 1-figure supplement 1-source data 1/Original files for western blot analysis displayed in Figure 1-figure supplement 1D/Vps35 Dauv 20231122_121824_Ch/Vps35 Dauv 20231122_121824_Ch-Marker.jpg]

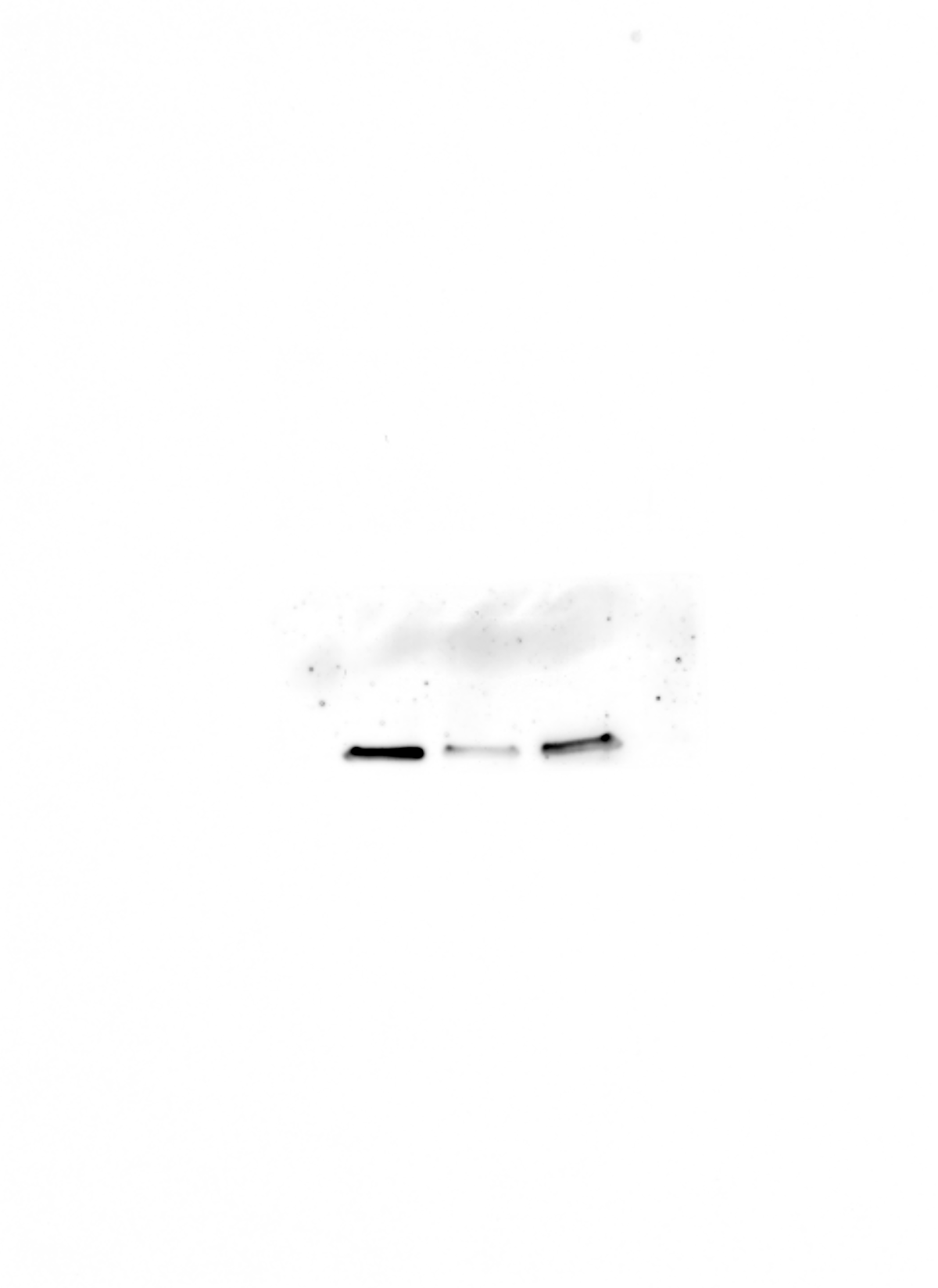

Supplement: Figure 1—figure supplement 1—source data 1. [file elife-105821-fig1-figsupp1-data1.zip › Figure 1-figure supplement 1-source data 1/Original files for western blot analysis displayed in Figure 1-figure supplement 1D/Vps35 Dauv 20231122_121824_Ch/Vps35 Dauv 20231122_121824_Ch_Chemi-2.tif]

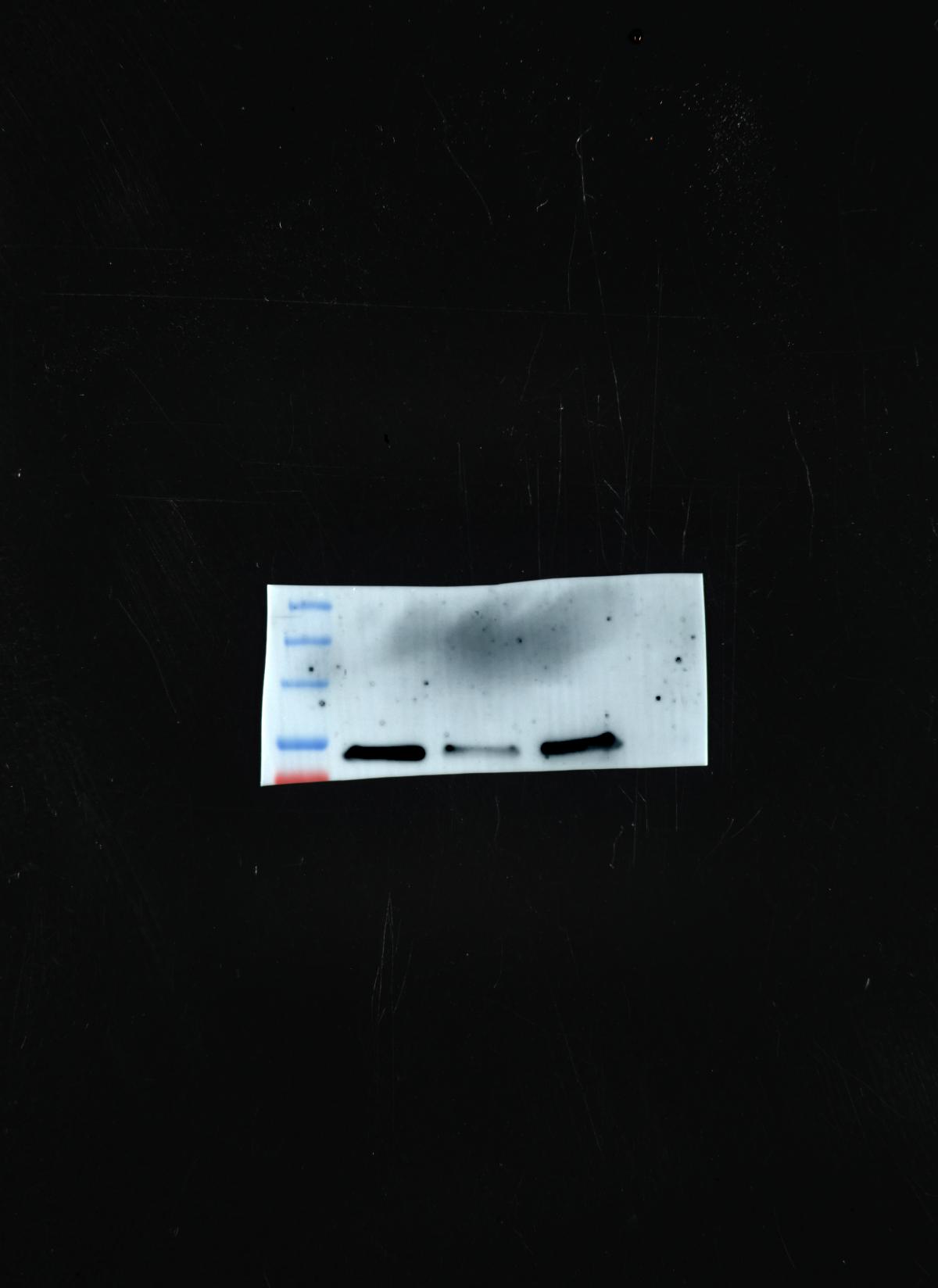

Supplement: Figure 1—figure supplement 1—source data 1. [file elife-105821-fig1-figsupp1-data1.zip › Figure 1-figure supplement 1-source data 1/Original files for western blot analysis displayed in Figure 1-figure supplement 1D/Vps35 Dauv 20231122_121824_Ch/Vps35 Dauv 20231122_121824_Ch_Chemi+Marker.jpg]

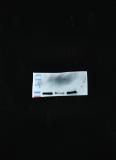

Supplement: Figure 1—figure supplement 1—source data 1. [file elife-105821-fig1-figsupp1-data1.zip › Figure 1-figure supplement 1-source data 1/Original files for western blot analysis displayed in Figure 1-figure supplement 1D/Vps35 Dauv 20231122_121824_Ch/Vps35 Dauv 20231122_121824_Ch_Thumb.jpg]

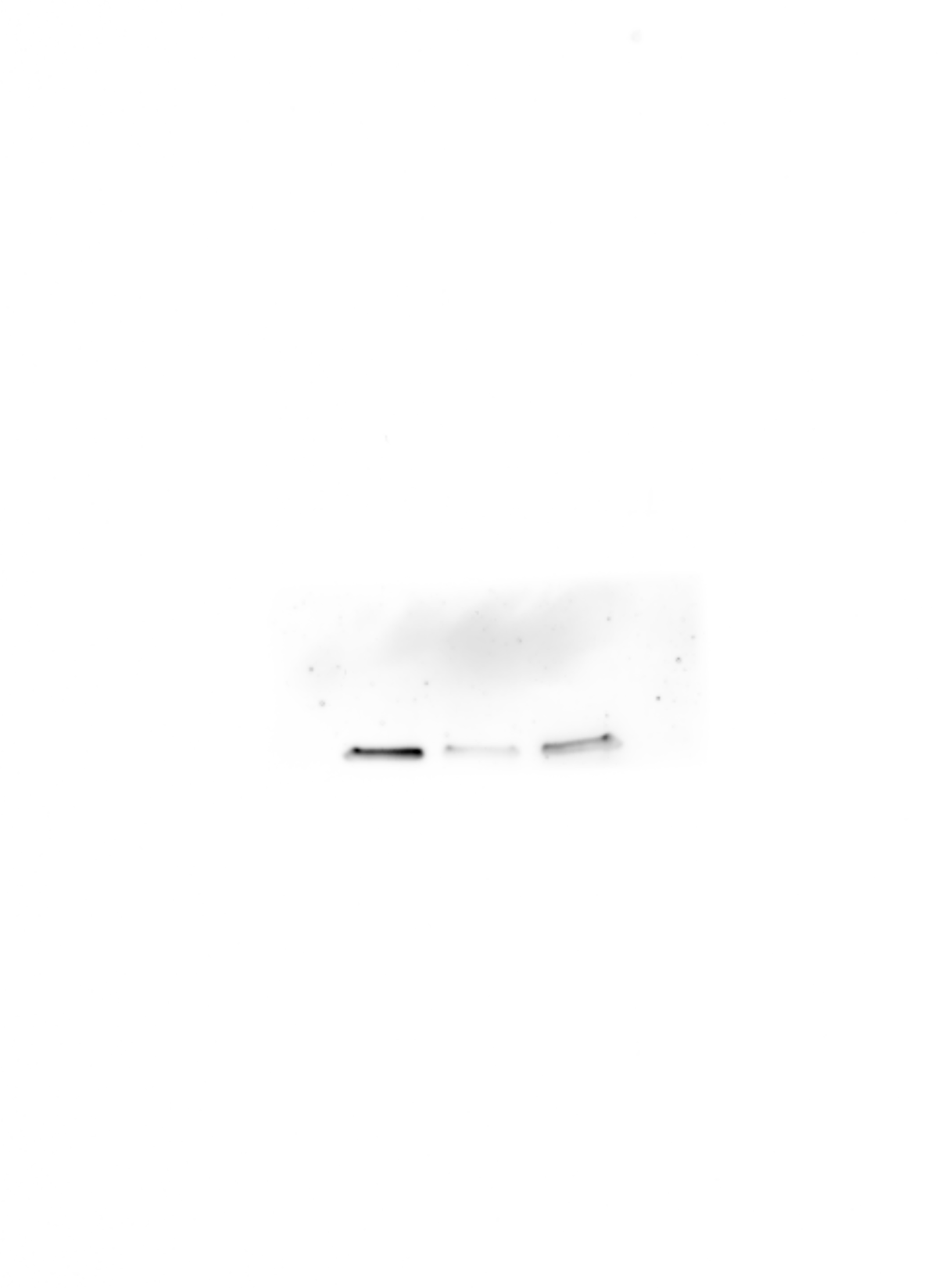

Supplement: Figure 1—figure supplement 1—source data 1. [file elife-105821-fig1-figsupp1-data1.zip › Figure 1-figure supplement 1-source data 1/Original files for western blot analysis displayed in Figure 1-figure supplement 1D/Vps35 Dauv 20231122_121824_Ch/Vps35 Dauv 20231122_121824_Ch_Chemi.tif]

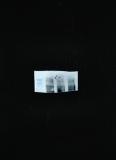

Supplement: Figure 1—figure supplement 1—source data 1. [file elife-105821-fig1-figsupp1-data1.zip › Figure 1-figure supplement 1-source data 1/Original files for western blot analysis displayed in Figure 1-figure supplement 1D/SNAP Dauv 20231122_120625-06_Ch/SNAP Dauv 20231122_120625-06_Ch_Thumb.jpg]

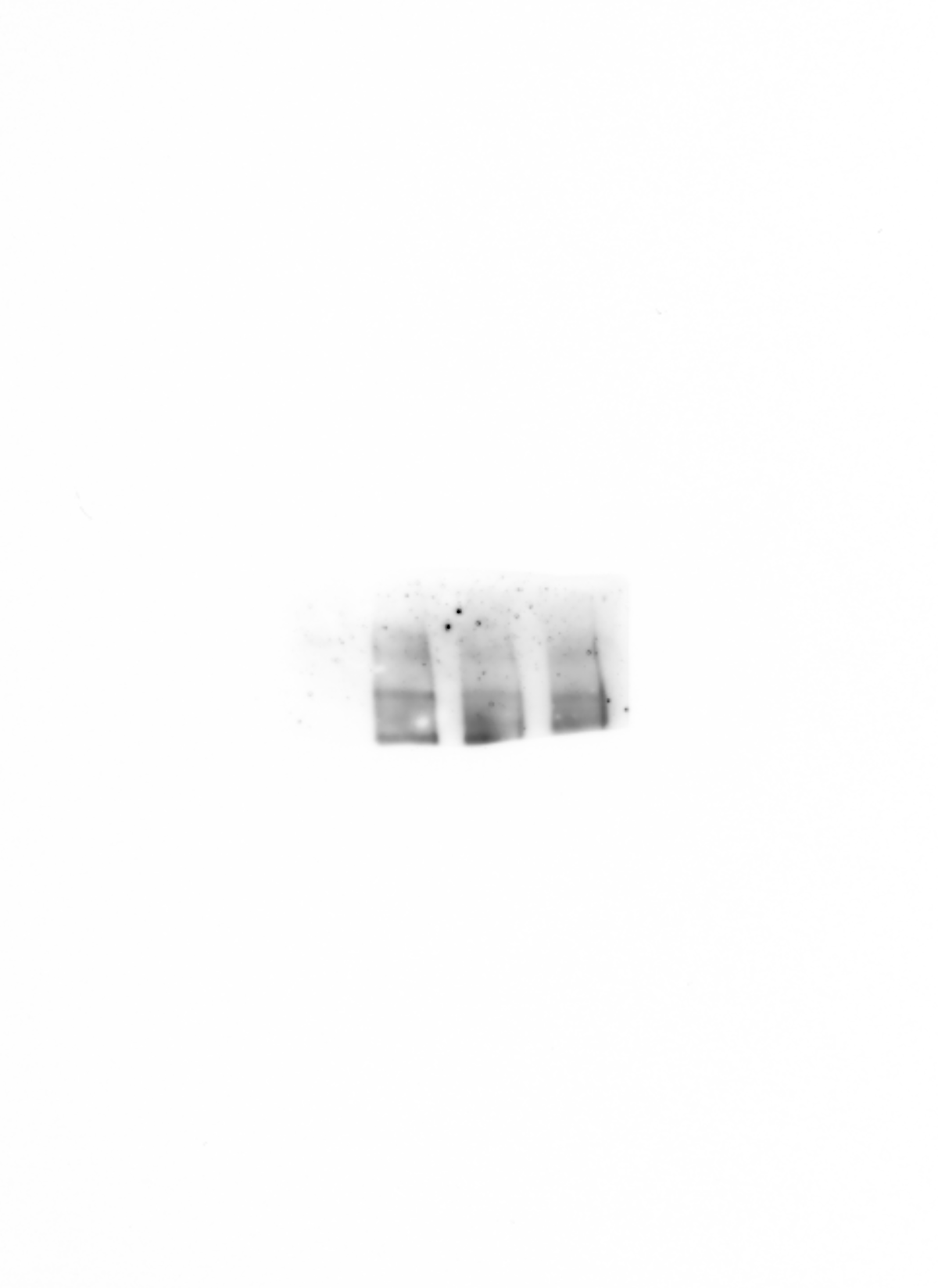

Supplement: Figure 1—figure supplement 1—source data 1. [file elife-105821-fig1-figsupp1-data1.zip › Figure 1-figure supplement 1-source data 1/Original files for western blot analysis displayed in Figure 1-figure supplement 1D/SNAP Dauv 20231122_120625-06_Ch/SNAP Dauv 20231122_120625-06_Ch_Chemi.tif]

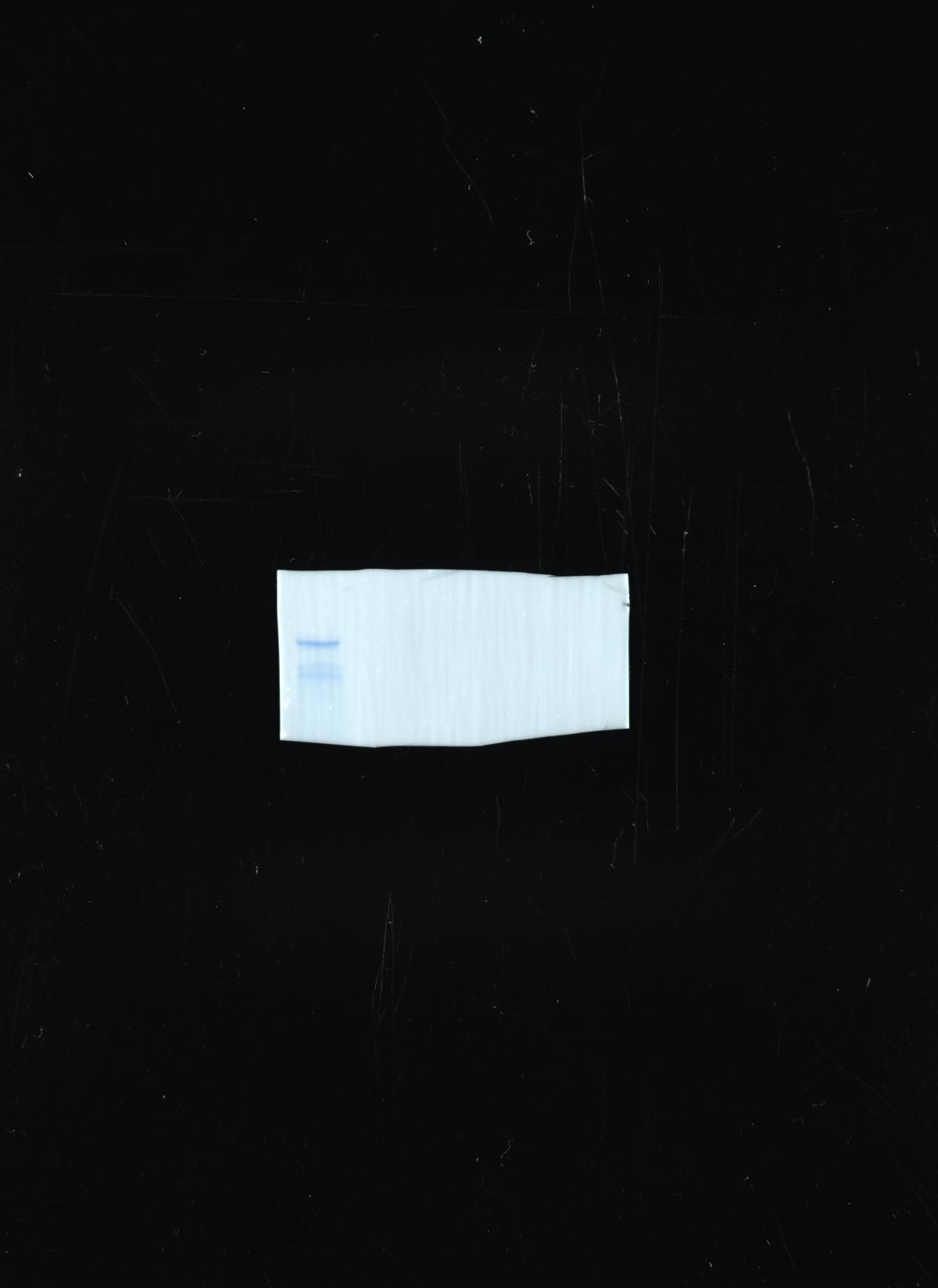

Supplement: Figure 1—figure supplement 1—source data 1. [file elife-105821-fig1-figsupp1-data1.zip › Figure 1-figure supplement 1-source data 1/Original files for western blot analysis displayed in Figure 1-figure supplement 1D/SNAP Dauv 20231122_120625-06_Ch/SNAP Dauv 20231122_120625-06_Ch-Marker.jpg]

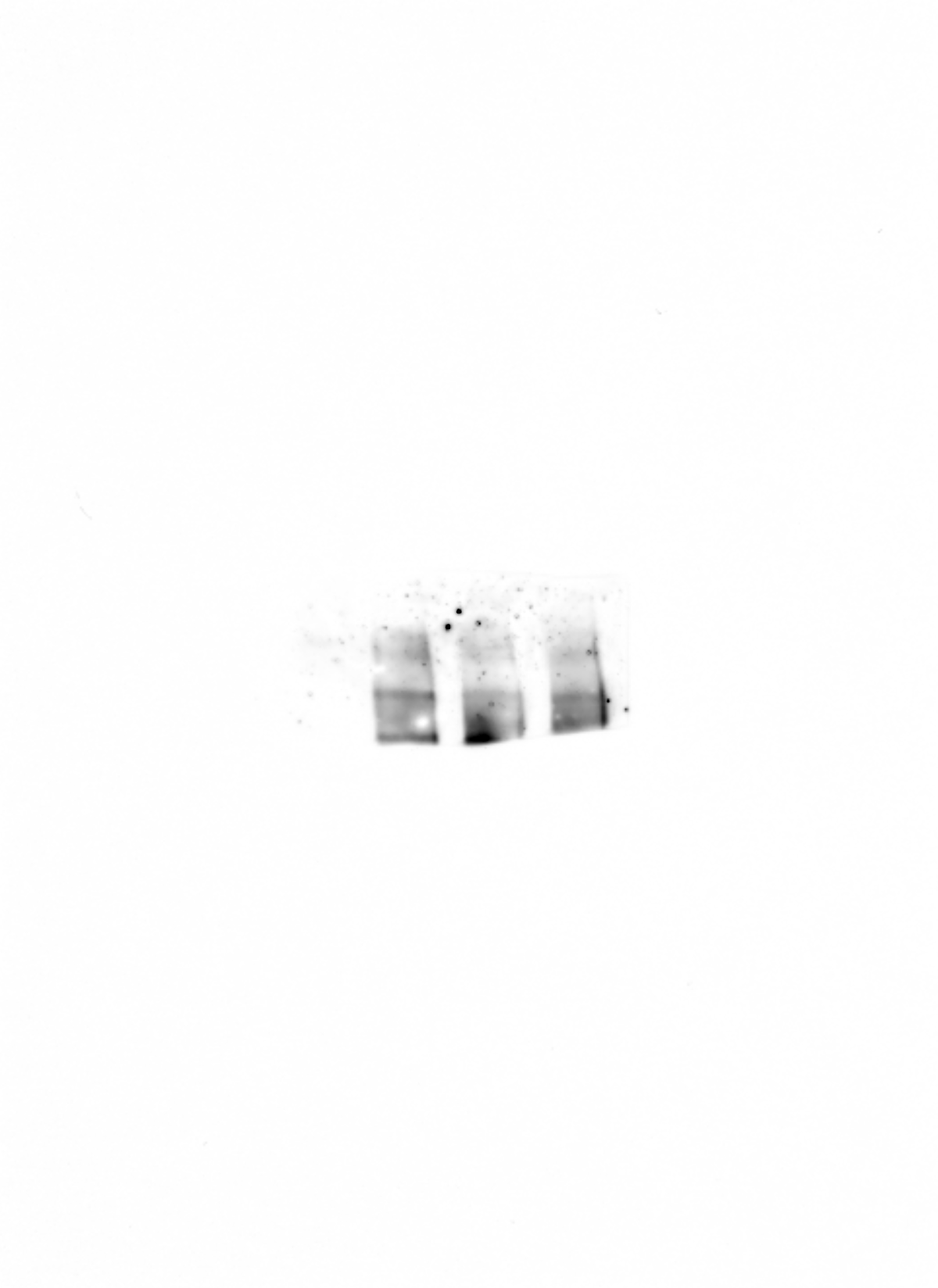

Supplement: Figure 1—figure supplement 1—source data 1. [file elife-105821-fig1-figsupp1-data1.zip › Figure 1-figure supplement 1-source data 1/Original files for western blot analysis displayed in Figure 1-figure supplement 1D/SNAP Dauv 20231122_120625-06_Ch/SNAP Dauv 20231122_120625-06_Ch_Chemi-2.tif]

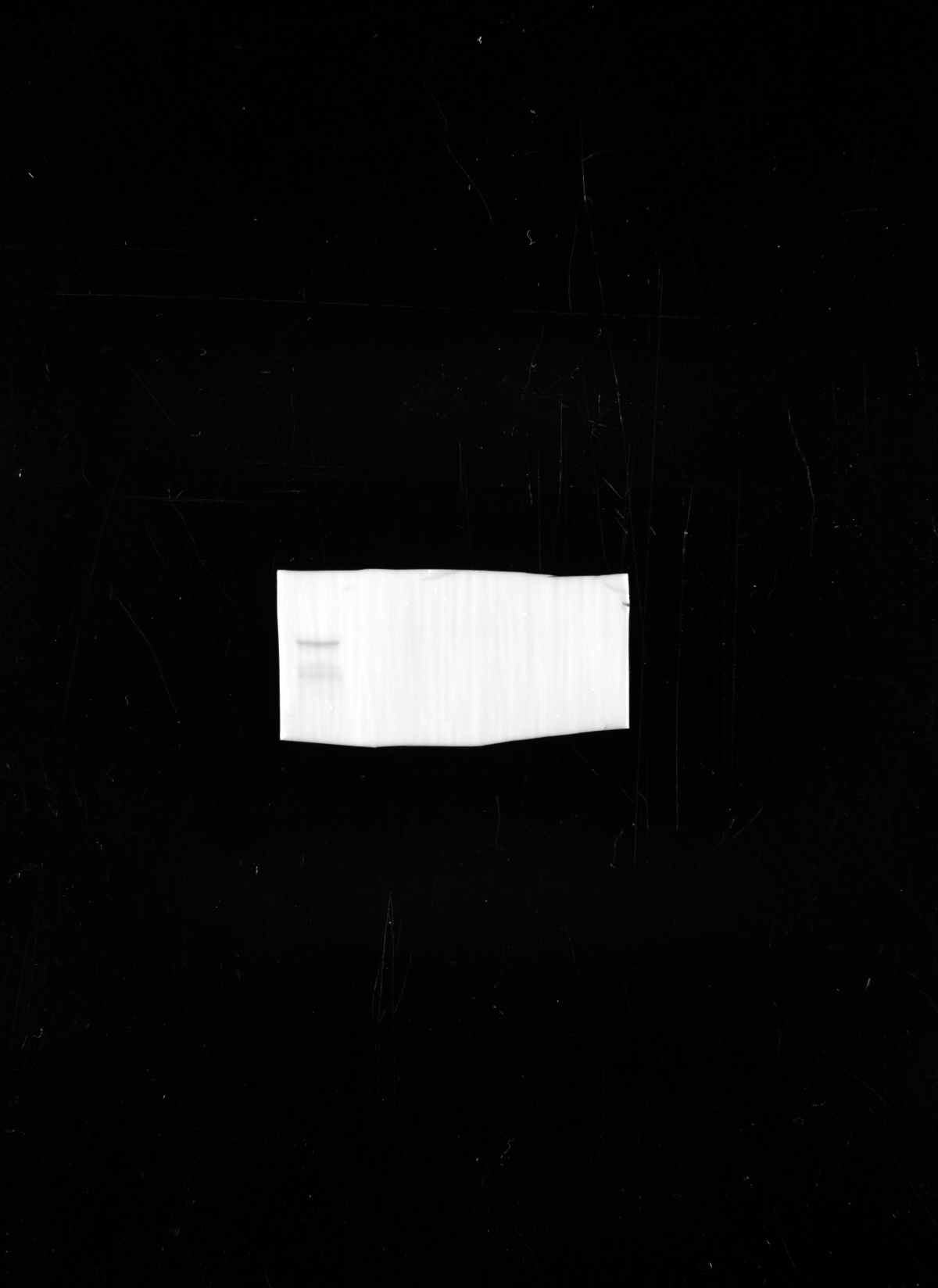

Supplement: Figure 1—figure supplement 1—source data 1. [file elife-105821-fig1-figsupp1-data1.zip › Figure 1-figure supplement 1-source data 1/Original files for western blot analysis displayed in Figure 1-figure supplement 1D/SNAP Dauv 20231122_120625-06_Ch/SNAP Dauv 20231122_120625-06_Ch-Marker.tif]

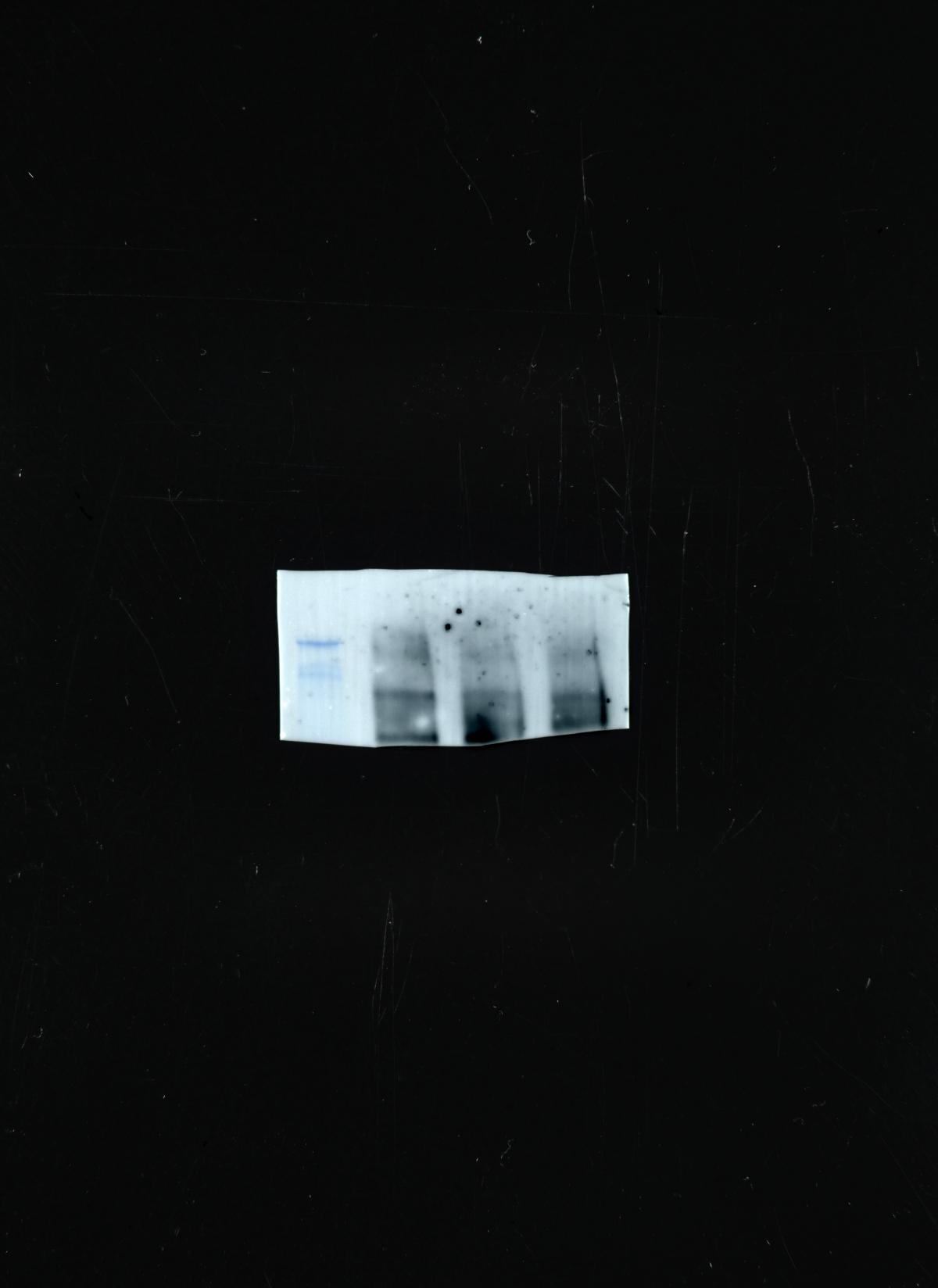

Supplement: Figure 1—figure supplement 1—source data 1. [file elife-105821-fig1-figsupp1-data1.zip › Figure 1-figure supplement 1-source data 1/Original files for western blot analysis displayed in Figure 1-figure supplement 1D/SNAP Dauv 20231122_120625-06_Ch/SNAP Dauv 20231122_120625-06_Ch_Chemi+Marker.jpg]

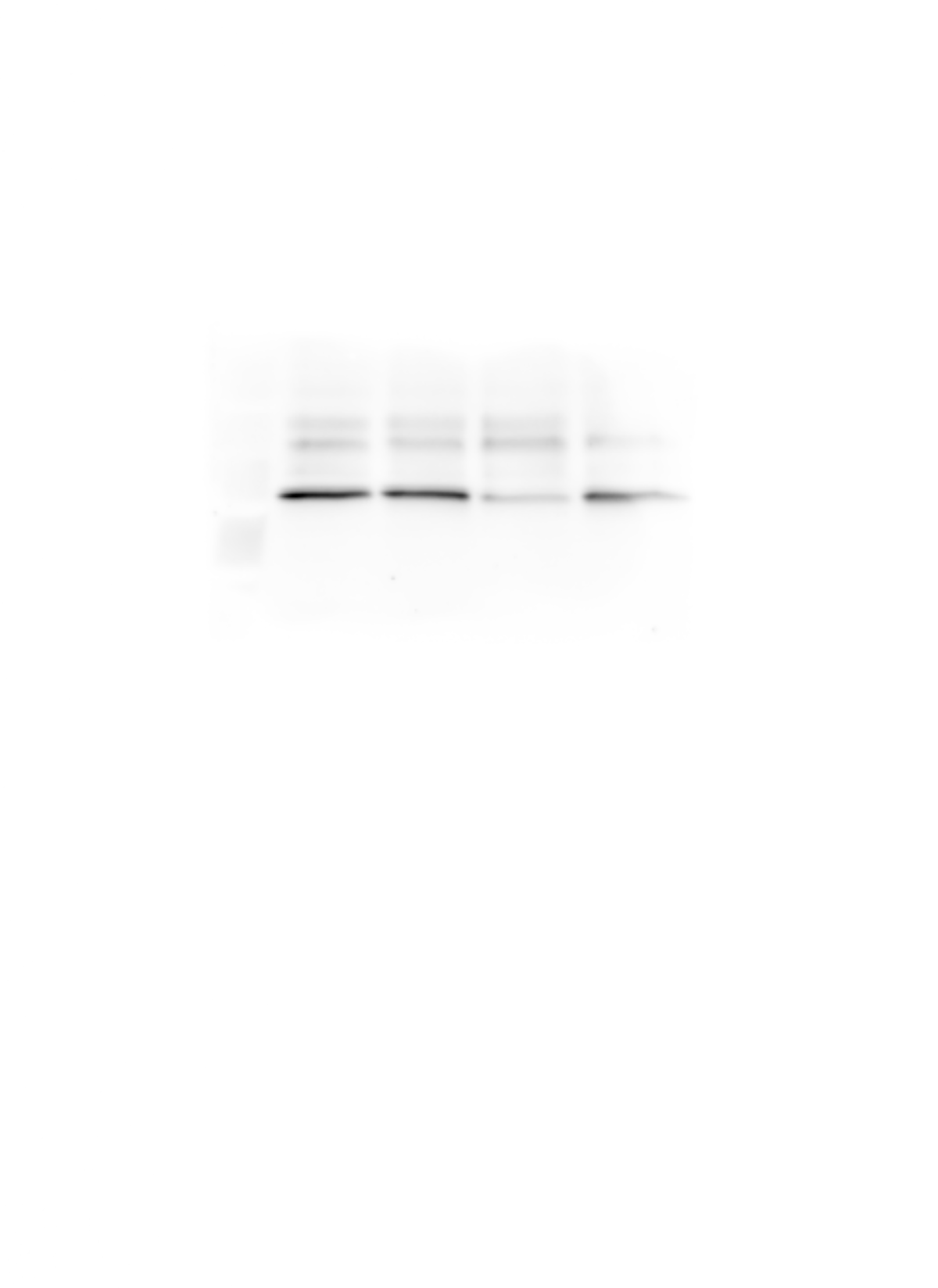

Supplement: Figure 1—figure supplement 1—source data 1. [file elife-105821-fig1-figsupp1-data1.zip › Figure 1-figure supplement 1-source data 1/Original files for western blot analysis displayed in Figure 1-figure supplement 1F/Rab6 SNAPexp_chemi 20230510_121618_Ch/Rab6 SNAPexp_chemi 20230510_121618_Ch_Chemi.tif]

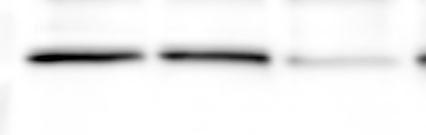

Supplement: Figure 1—figure supplement 1—source data 1. [file elife-105821-fig1-figsupp1-data1.zip › Figure 1-figure supplement 1-source data 1/Original files for western blot analysis displayed in Figure 1-figure supplement 1F/Rab6 SNAPexp_chemi 20230510_121618_Ch/Rab6 SNAPexp_chemi 20230510_121618_Ch_Chemi-1.tif]

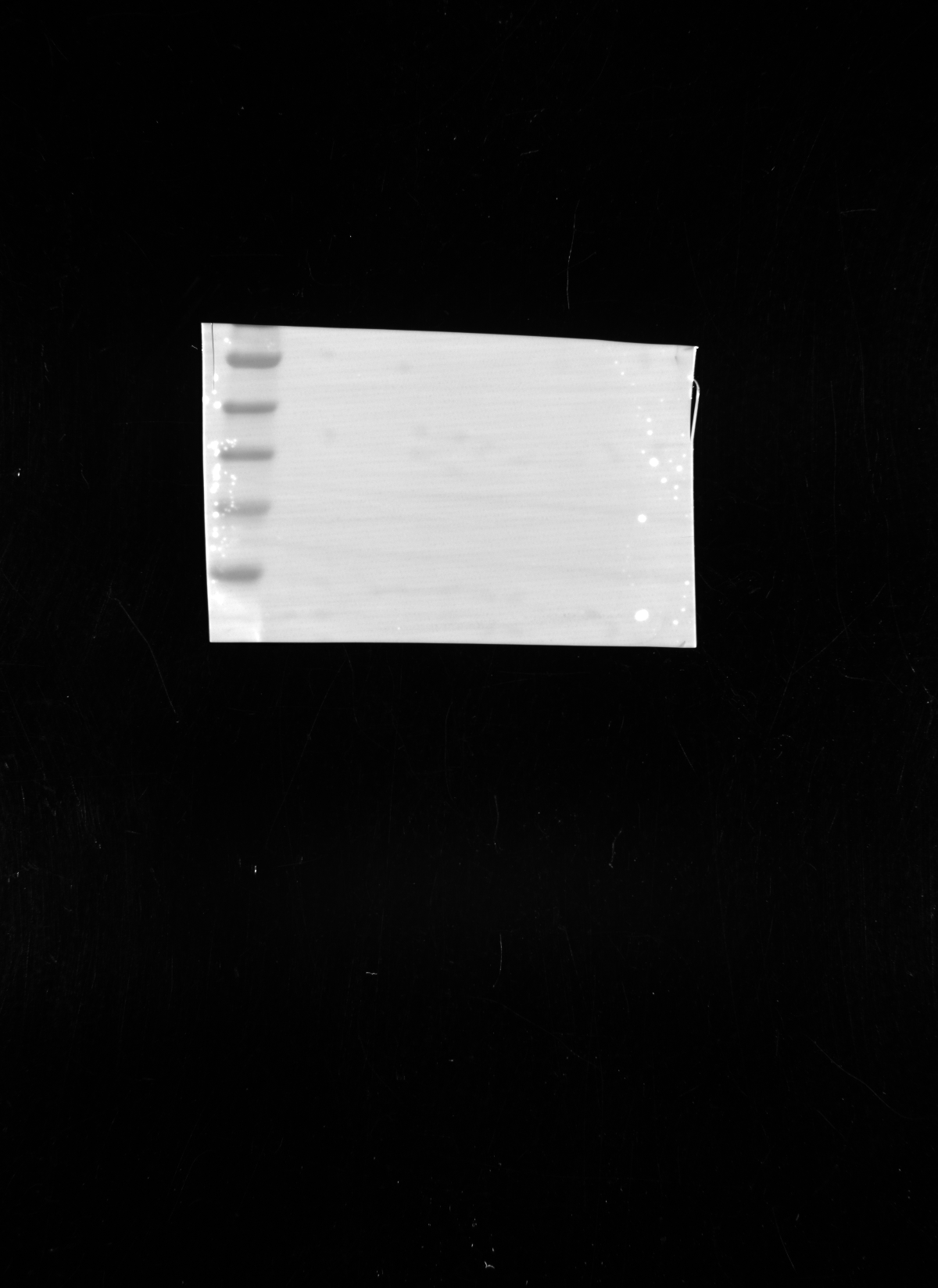

Supplement: Figure 1—figure supplement 1—source data 1. [file elife-105821-fig1-figsupp1-data1.zip › Figure 1-figure supplement 1-source data 1/Original files for western blot analysis displayed in Figure 1-figure supplement 1F/Rab6 SNAPexp_chemi 20230510_121618_Ch/Rab6 SNAPexp_chemi 20230510_121618_Ch-Marker.tif]
