# Supplementary figures and images for: Clathrin-independent endocytosis and retrograde transport in cancer cells tune immune synapse organization and CD8 T cell response (part 2 of 2)
Source: eLife. 2026 Apr 22;14:RP105821. doi: 10.7554/eLife.105821 (PMC13102394; doi:10.7554/eLife.105821)

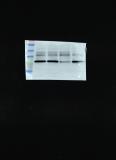

Supplement: Figure 1—figure supplement 1—source data 1. [file elife-105821-fig1-figsupp1-data1.zip › Figure 1-figure supplement 1-source data 1/Original files for western blot analysis displayed in Figure 1-figure supplement 1F/Rab6 SNAPexp_chemi 20230510_121618_Ch/Rab6 SNAPexp_chemi 20230510_121618_Ch_Thumb.jpg]

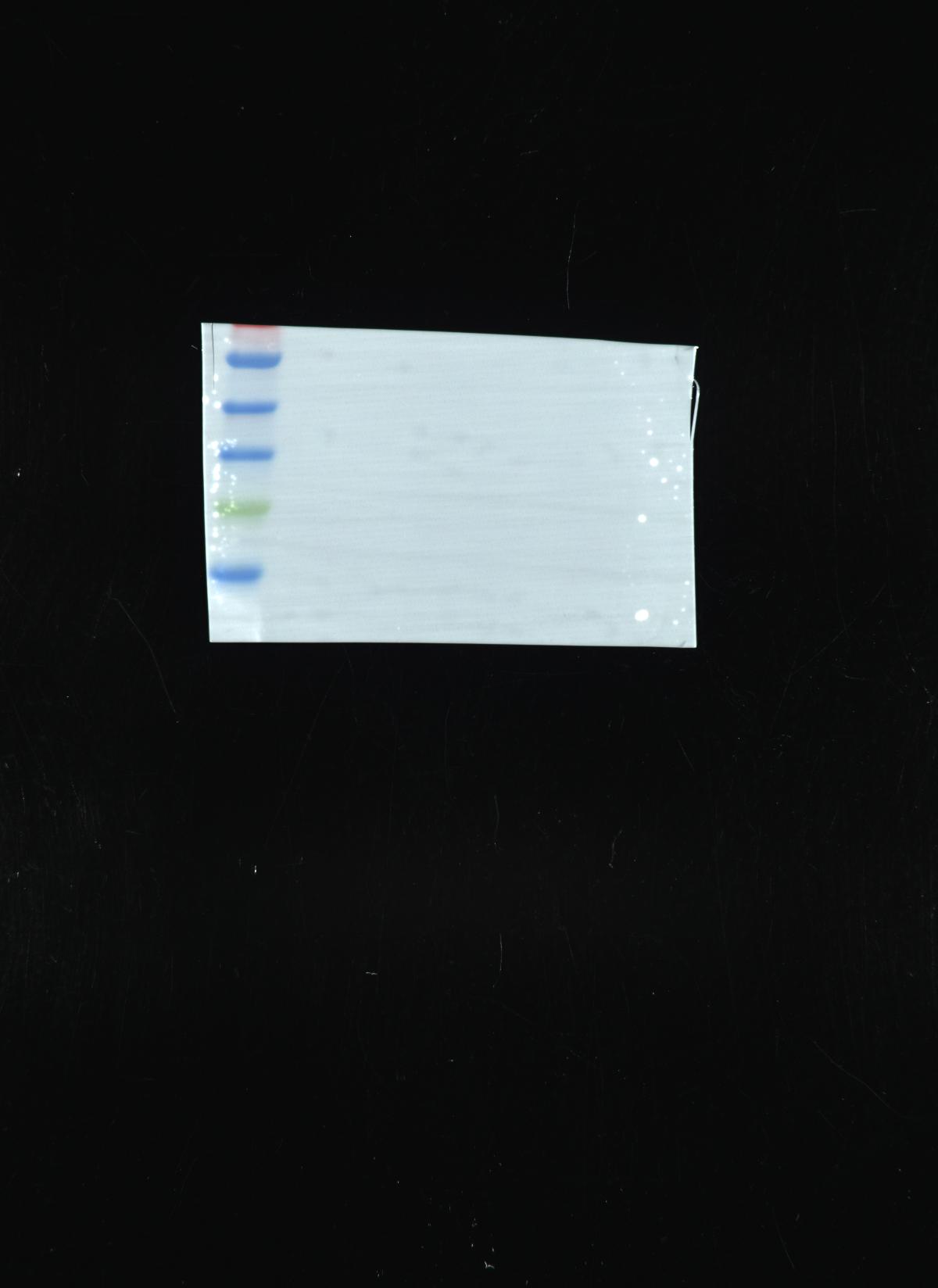

Supplement: Figure 1—figure supplement 1—source data 1. [file elife-105821-fig1-figsupp1-data1.zip › Figure 1-figure supplement 1-source data 1/Original files for western blot analysis displayed in Figure 1-figure supplement 1F/Rab6 SNAPexp_chemi 20230510_121618_Ch/Rab6 SNAPexp_chemi 20230510_121618_Ch-Marker.jpg]

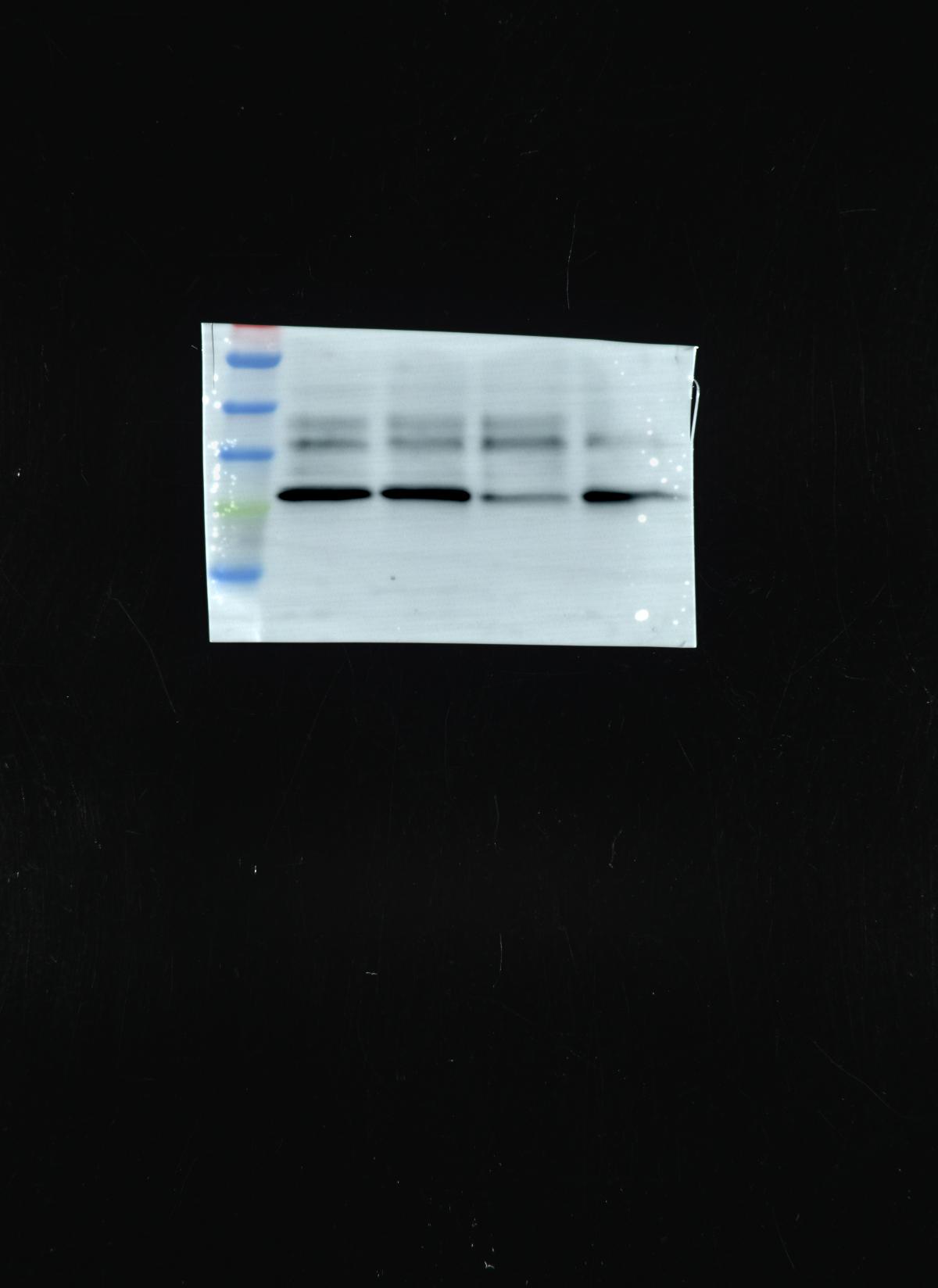

Supplement: Figure 1—figure supplement 1—source data 1. [file elife-105821-fig1-figsupp1-data1.zip › Figure 1-figure supplement 1-source data 1/Original files for western blot analysis displayed in Figure 1-figure supplement 1F/Rab6 SNAPexp_chemi 20230510_121618_Ch/Rab6 SNAPexp_chemi 20230510_121618_Ch_Chemi+Marker.jpg]

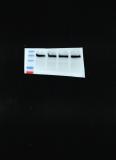

Supplement: Figure 1—figure supplement 1—source data 1. [file elife-105821-fig1-figsupp1-data1.zip › Figure 1-figure supplement 1-source data 1/Original files for western blot analysis displayed in Figure 1-figure supplement 1F/Clathrin SNAPexp_chemi 20230510_121318_Ch/Clathrin SNAPexp_chemi 20230510_121318_Ch_Thumb.jpg]

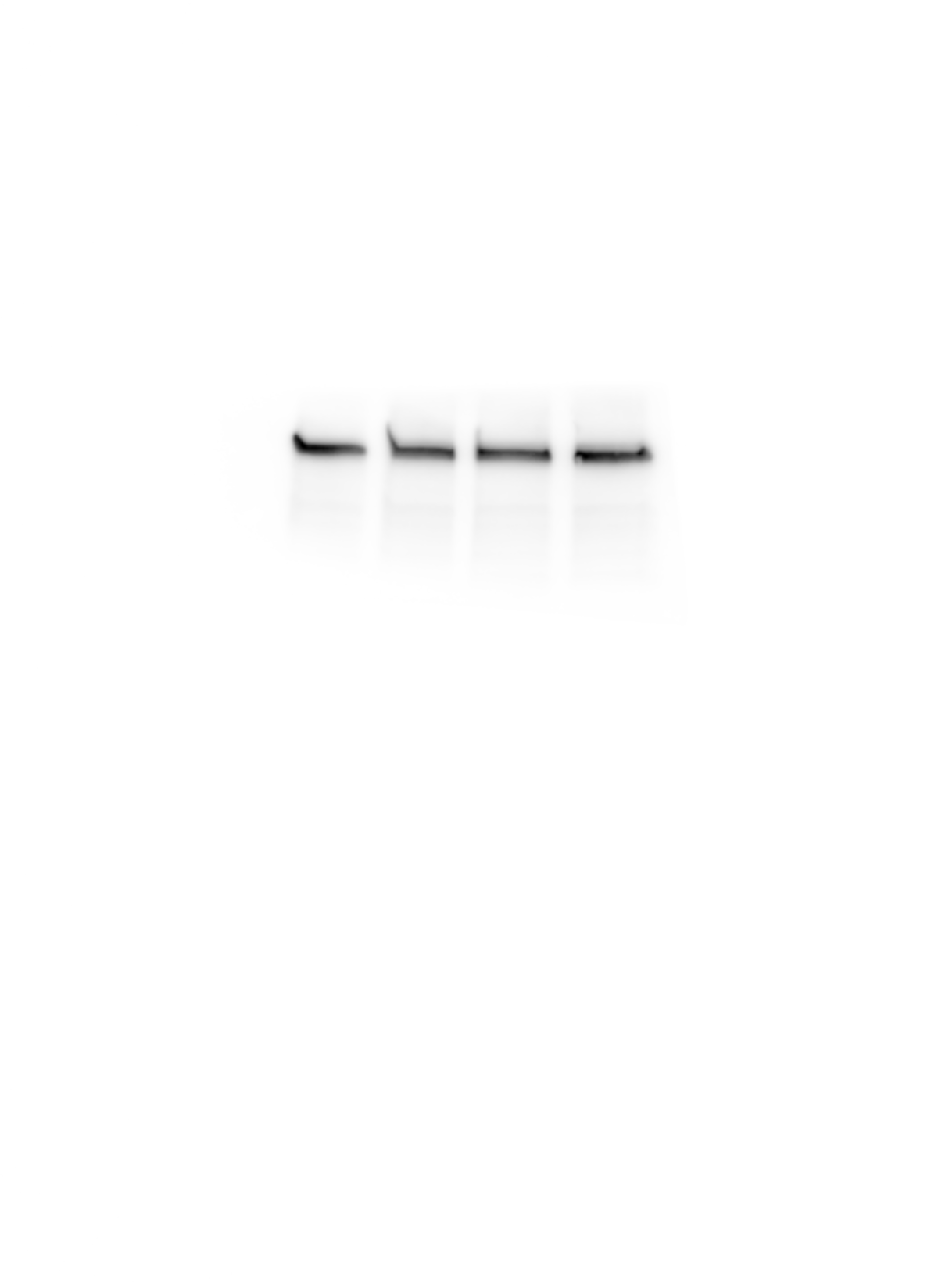

Supplement: Figure 1—figure supplement 1—source data 1. [file elife-105821-fig1-figsupp1-data1.zip › Figure 1-figure supplement 1-source data 1/Original files for western blot analysis displayed in Figure 1-figure supplement 1F/Clathrin SNAPexp_chemi 20230510_121318_Ch/Clathrin SNAPexp_chemi 20230510_121318_Ch_Chemi.tif]

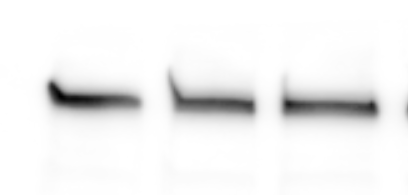

Supplement: Figure 1—figure supplement 1—source data 1. [file elife-105821-fig1-figsupp1-data1.zip › Figure 1-figure supplement 1-source data 1/Original files for western blot analysis displayed in Figure 1-figure supplement 1F/Clathrin SNAPexp_chemi 20230510_121318_Ch/Clathrin SNAPexp_chemi 20230510_121318_Ch_Chemi-1.tif]

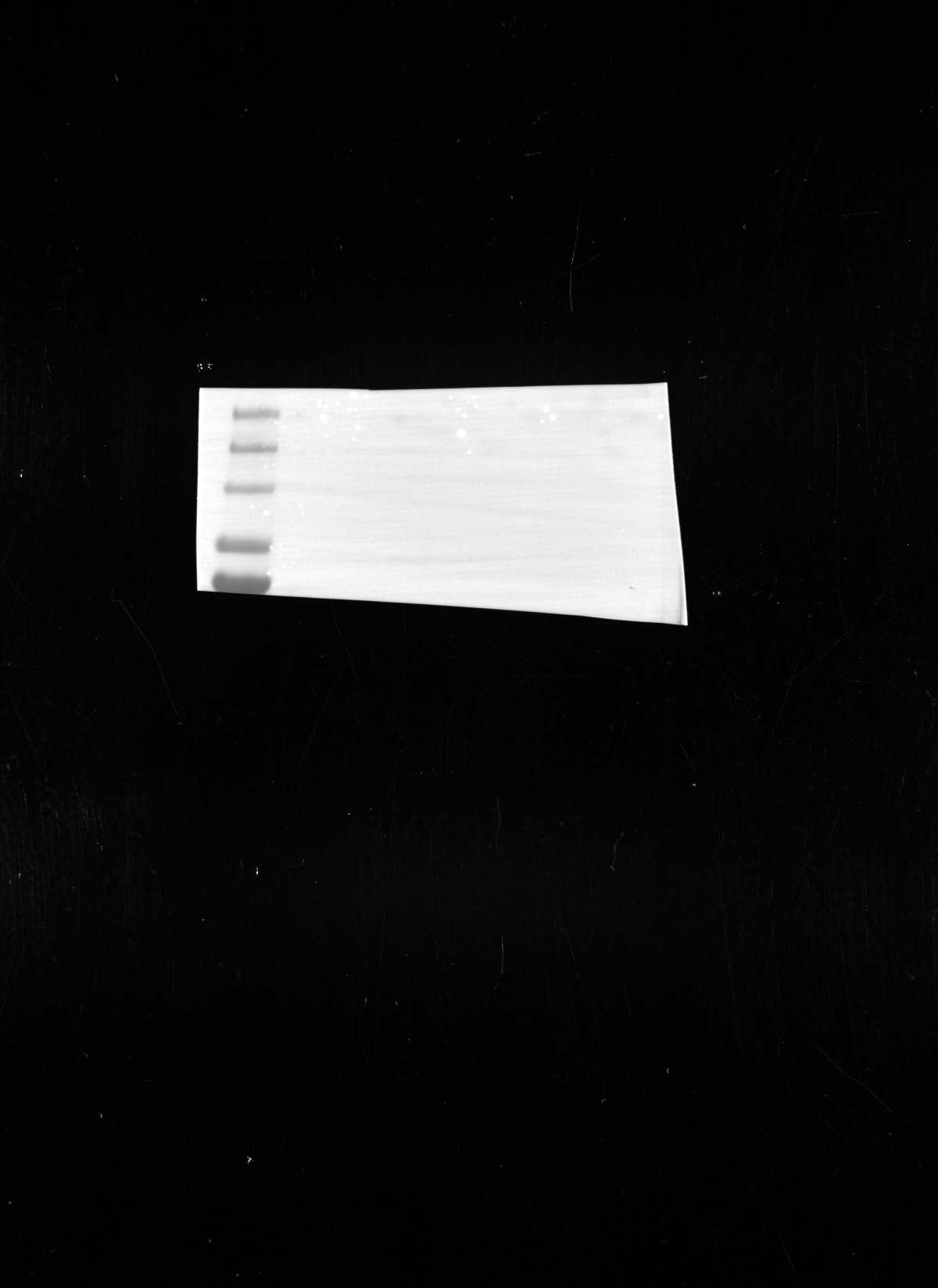

Supplement: Figure 1—figure supplement 1—source data 1. [file elife-105821-fig1-figsupp1-data1.zip › Figure 1-figure supplement 1-source data 1/Original files for western blot analysis displayed in Figure 1-figure supplement 1F/Clathrin SNAPexp_chemi 20230510_121318_Ch/Clathrin SNAPexp_chemi 20230510_121318_Ch-Marker.tif]

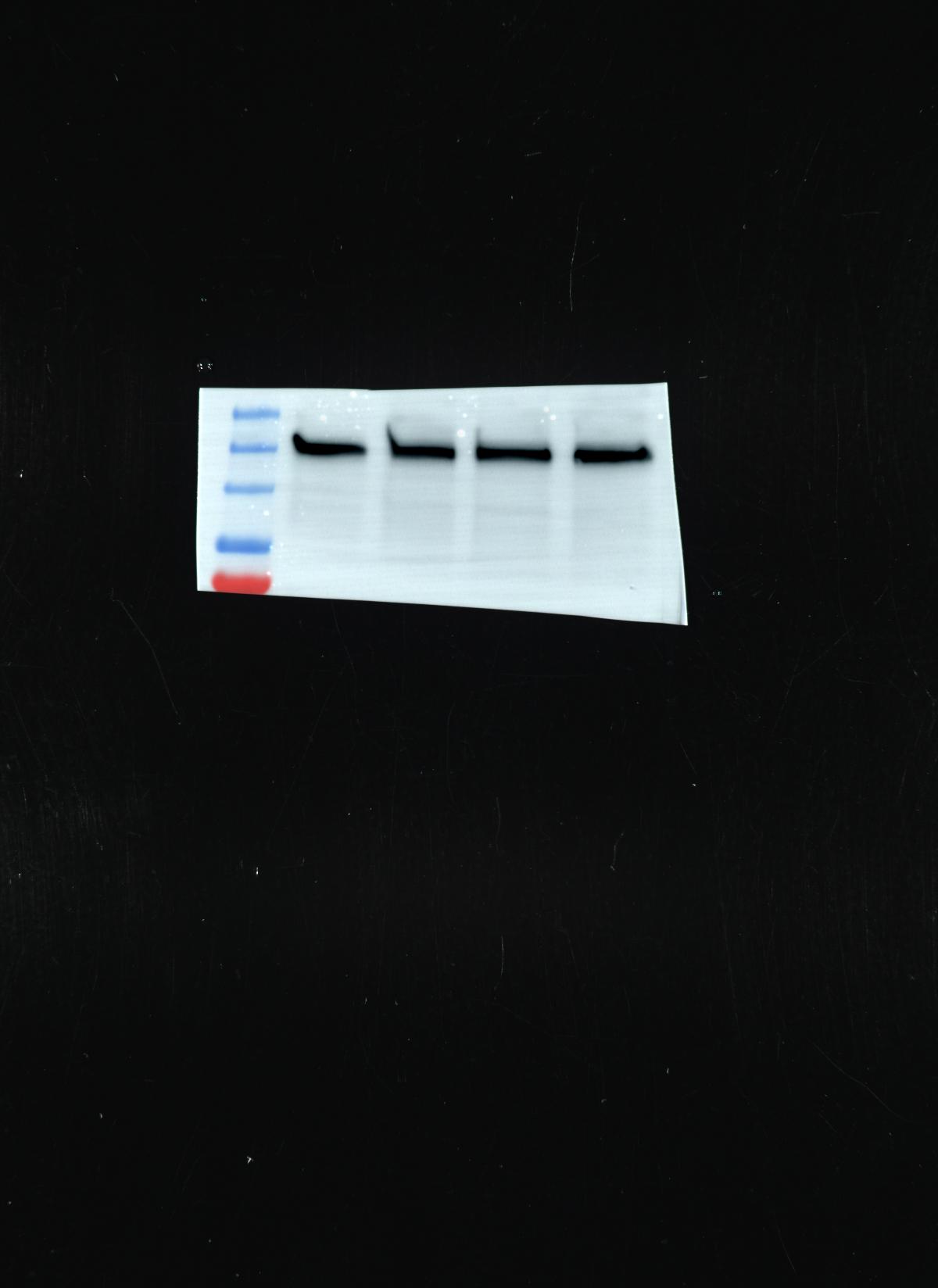

Supplement: Figure 1—figure supplement 1—source data 1. [file elife-105821-fig1-figsupp1-data1.zip › Figure 1-figure supplement 1-source data 1/Original files for western blot analysis displayed in Figure 1-figure supplement 1F/Clathrin SNAPexp_chemi 20230510_121318_Ch/Clathrin SNAPexp_chemi 20230510_121318_Ch_Chemi+Marker.jpg]

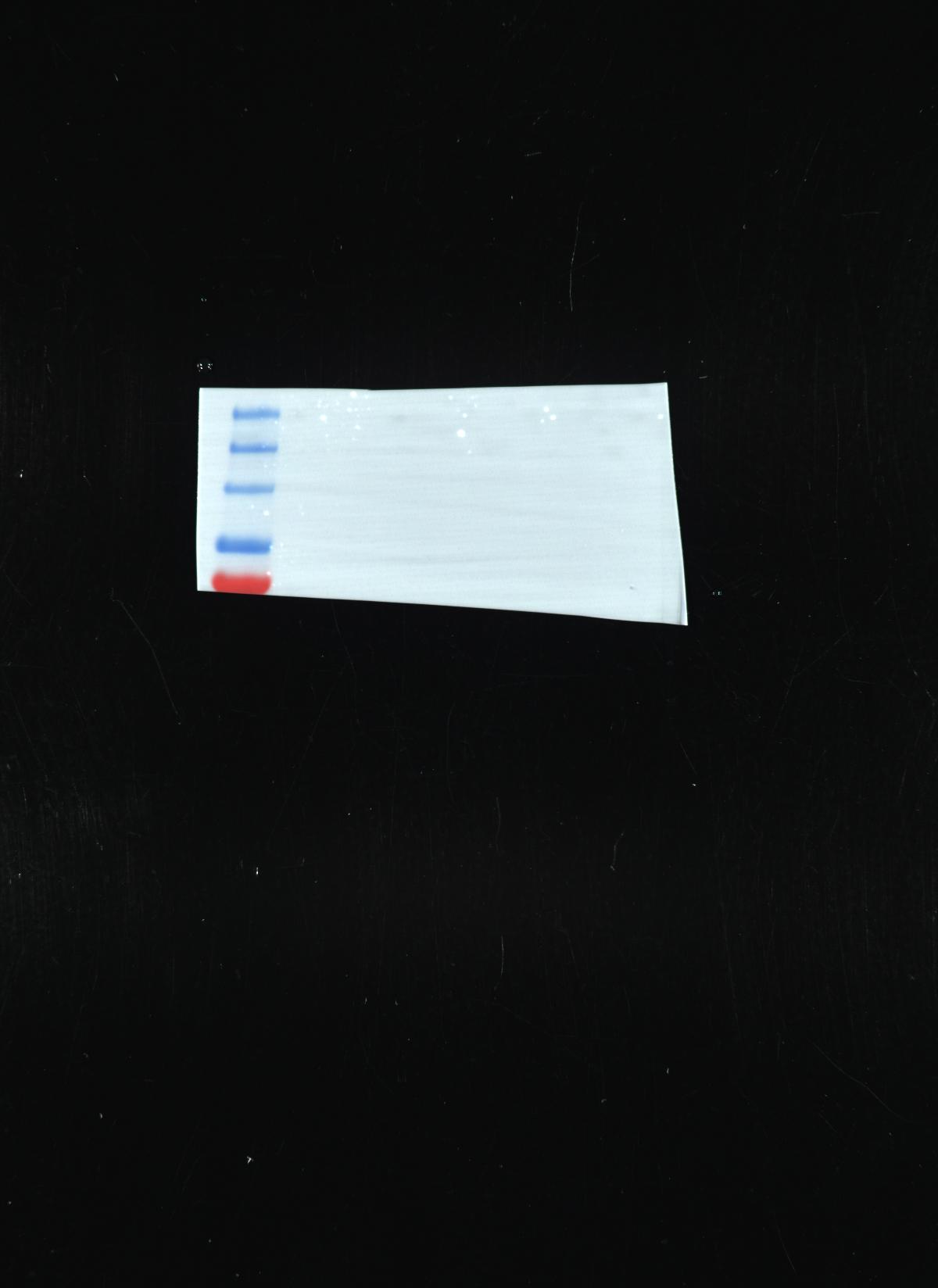

Supplement: Figure 1—figure supplement 1—source data 1. [file elife-105821-fig1-figsupp1-data1.zip › Figure 1-figure supplement 1-source data 1/Original files for western blot analysis displayed in Figure 1-figure supplement 1F/Clathrin SNAPexp_chemi 20230510_121318_Ch/Clathrin SNAPexp_chemi 20230510_121318_Ch-Marker.jpg]

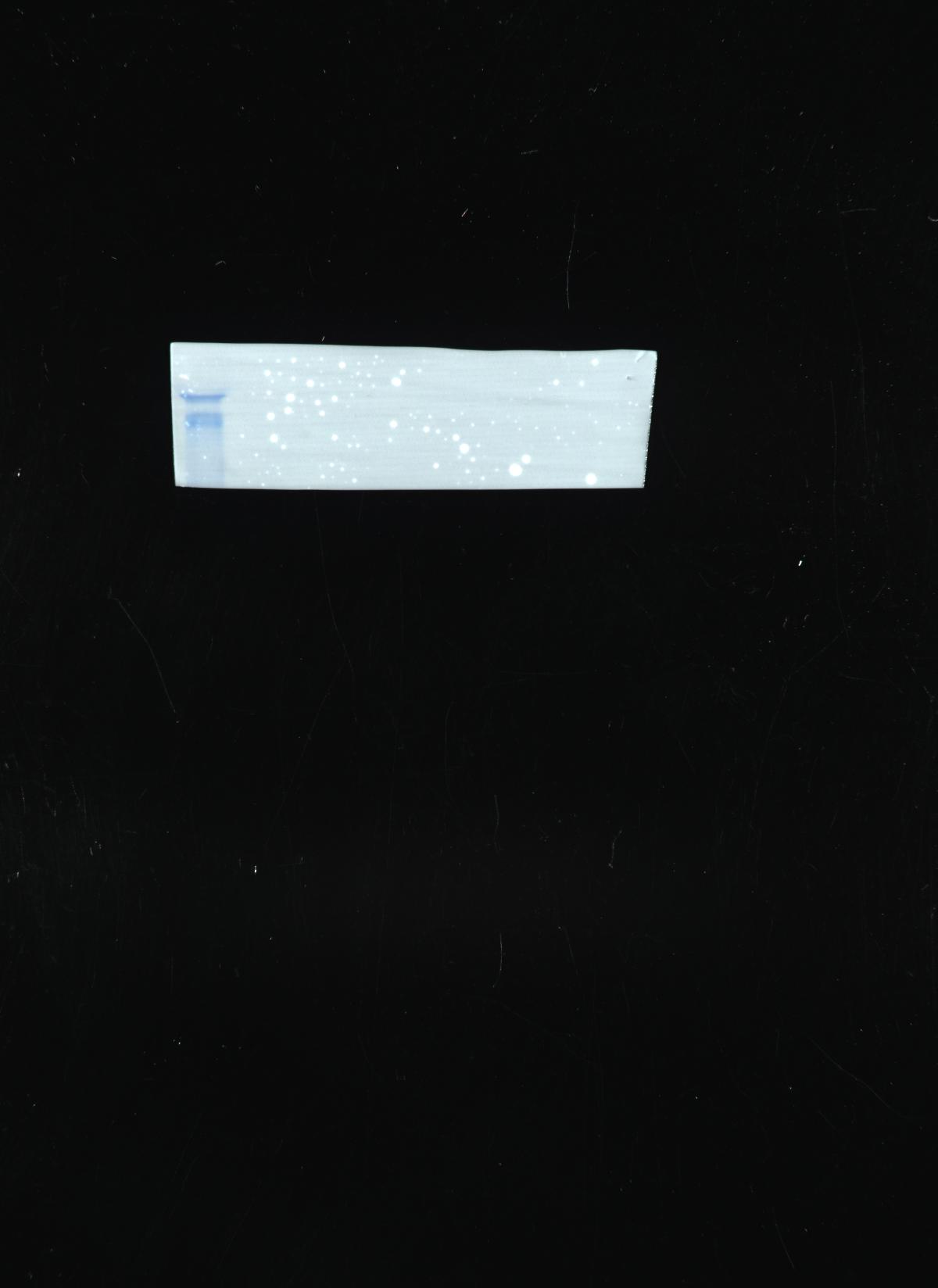

Supplement: Figure 1—figure supplement 1—source data 1. [file elife-105821-fig1-figsupp1-data1.zip › Figure 1-figure supplement 1-source data 1/Original files for western blot analysis displayed in Figure 1-figure supplement 1F/SNAP2 timeser SNAPexp_chemi 20230510_123238-05_Ch/SNAP2 timeser SNAPexp_chemi 20230510_123238-05_Ch-Marker.jpg]

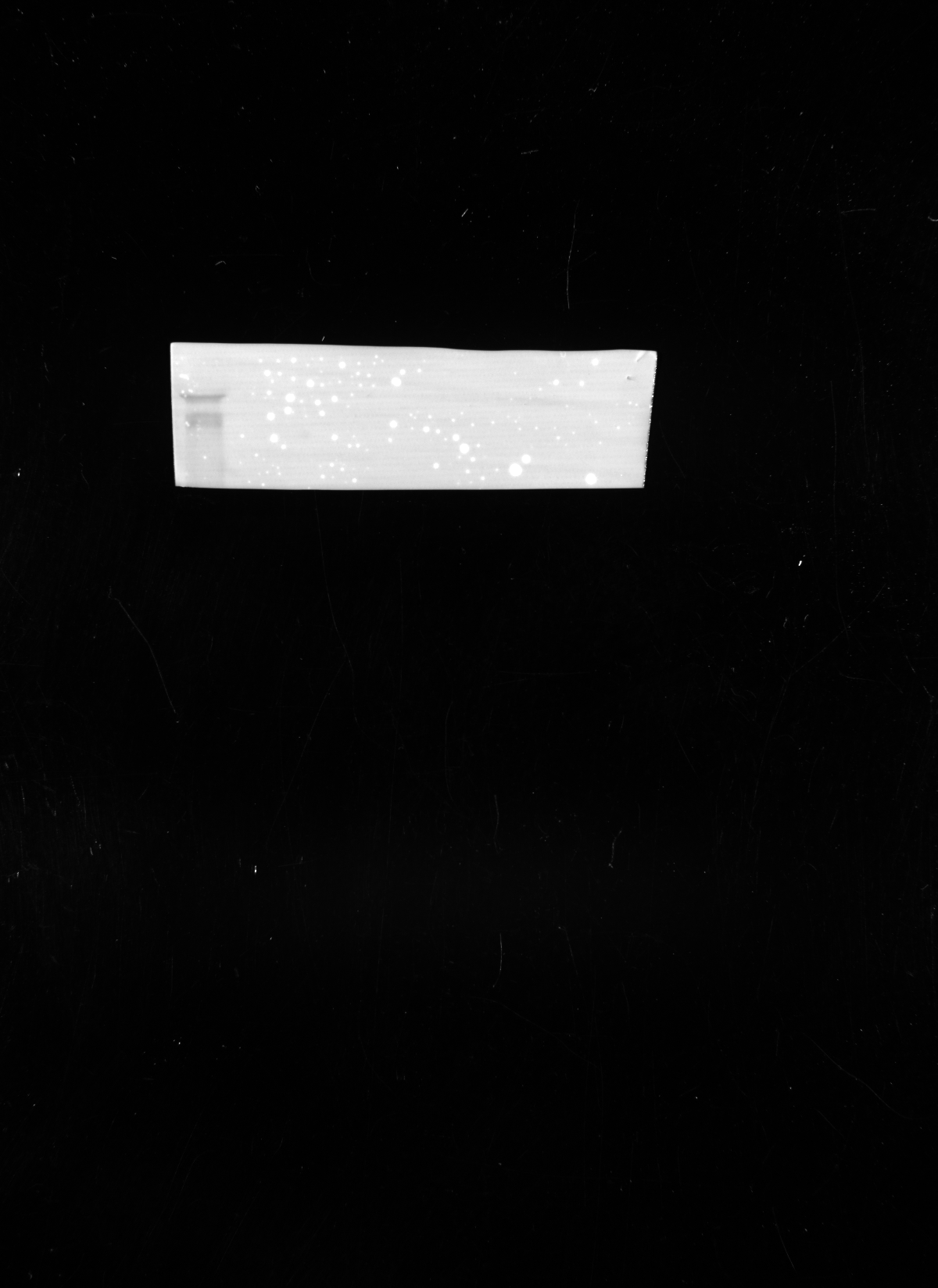

Supplement: Figure 1—figure supplement 1—source data 1. [file elife-105821-fig1-figsupp1-data1.zip › Figure 1-figure supplement 1-source data 1/Original files for western blot analysis displayed in Figure 1-figure supplement 1F/SNAP2 timeser SNAPexp_chemi 20230510_123238-05_Ch/SNAP2 timeser SNAPexp_chemi 20230510_123238-05_Ch-Marker.tif]

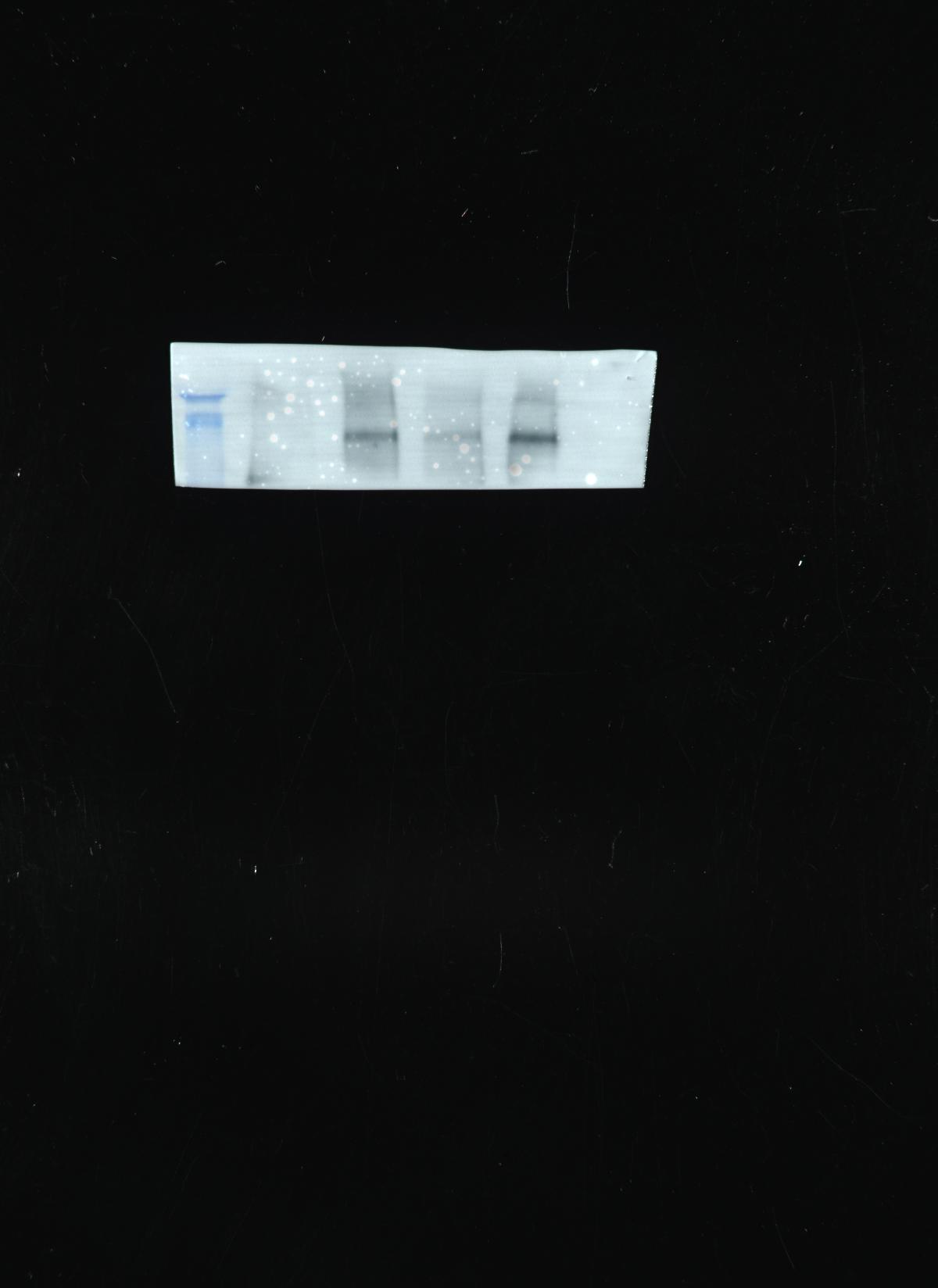

Supplement: Figure 1—figure supplement 1—source data 1. [file elife-105821-fig1-figsupp1-data1.zip › Figure 1-figure supplement 1-source data 1/Original files for western blot analysis displayed in Figure 1-figure supplement 1F/SNAP2 timeser SNAPexp_chemi 20230510_123238-05_Ch/SNAP2 timeser SNAPexp_chemi 20230510_123238-05_Ch_Chemi+Marker.jpg]

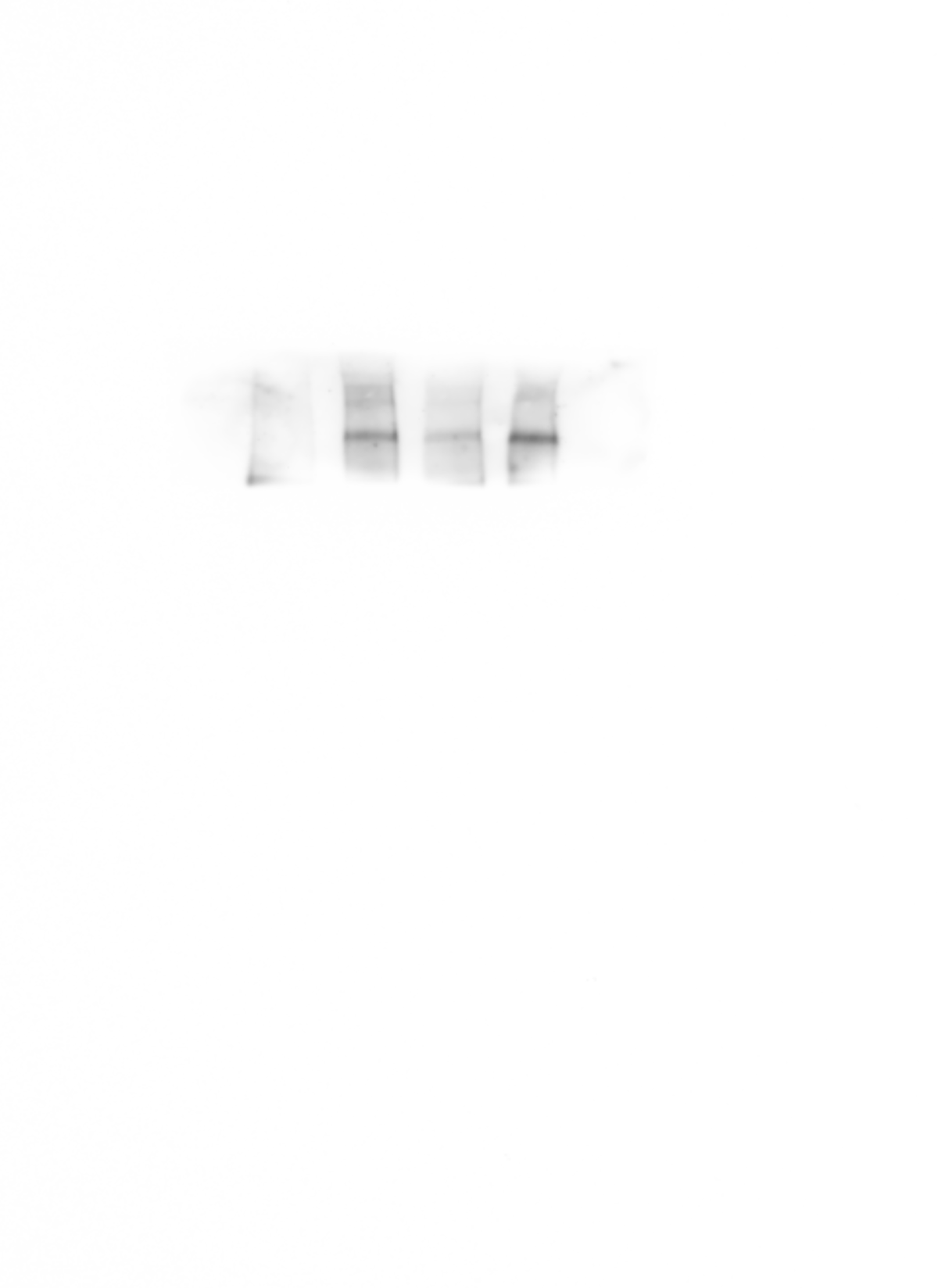

Supplement: Figure 1—figure supplement 1—source data 1. [file elife-105821-fig1-figsupp1-data1.zip › Figure 1-figure supplement 1-source data 1/Original files for western blot analysis displayed in Figure 1-figure supplement 1F/SNAP2 timeser SNAPexp_chemi 20230510_123238-05_Ch/SNAP2 timeser SNAPexp_chemi 20230510_123238-05_Ch_Chemi.tif]

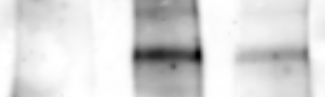

Supplement: Figure 1—figure supplement 1—source data 1. [file elife-105821-fig1-figsupp1-data1.zip › Figure 1-figure supplement 1-source data 1/Original files for western blot analysis displayed in Figure 1-figure supplement 1F/SNAP2 timeser SNAPexp_chemi 20230510_123238-05_Ch/SNAP2 timeser SNAPexp_chemi 20230510_123238-05_Ch_Chemi-1.tif]

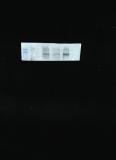

Supplement: Figure 1—figure supplement 1—source data 1. [file elife-105821-fig1-figsupp1-data1.zip › Figure 1-figure supplement 1-source data 1/Original files for western blot analysis displayed in Figure 1-figure supplement 1F/SNAP2 timeser SNAPexp_chemi 20230510_123238-05_Ch/SNAP2 timeser SNAPexp_chemi 20230510_123238-05_Ch_Thumb.jpg]

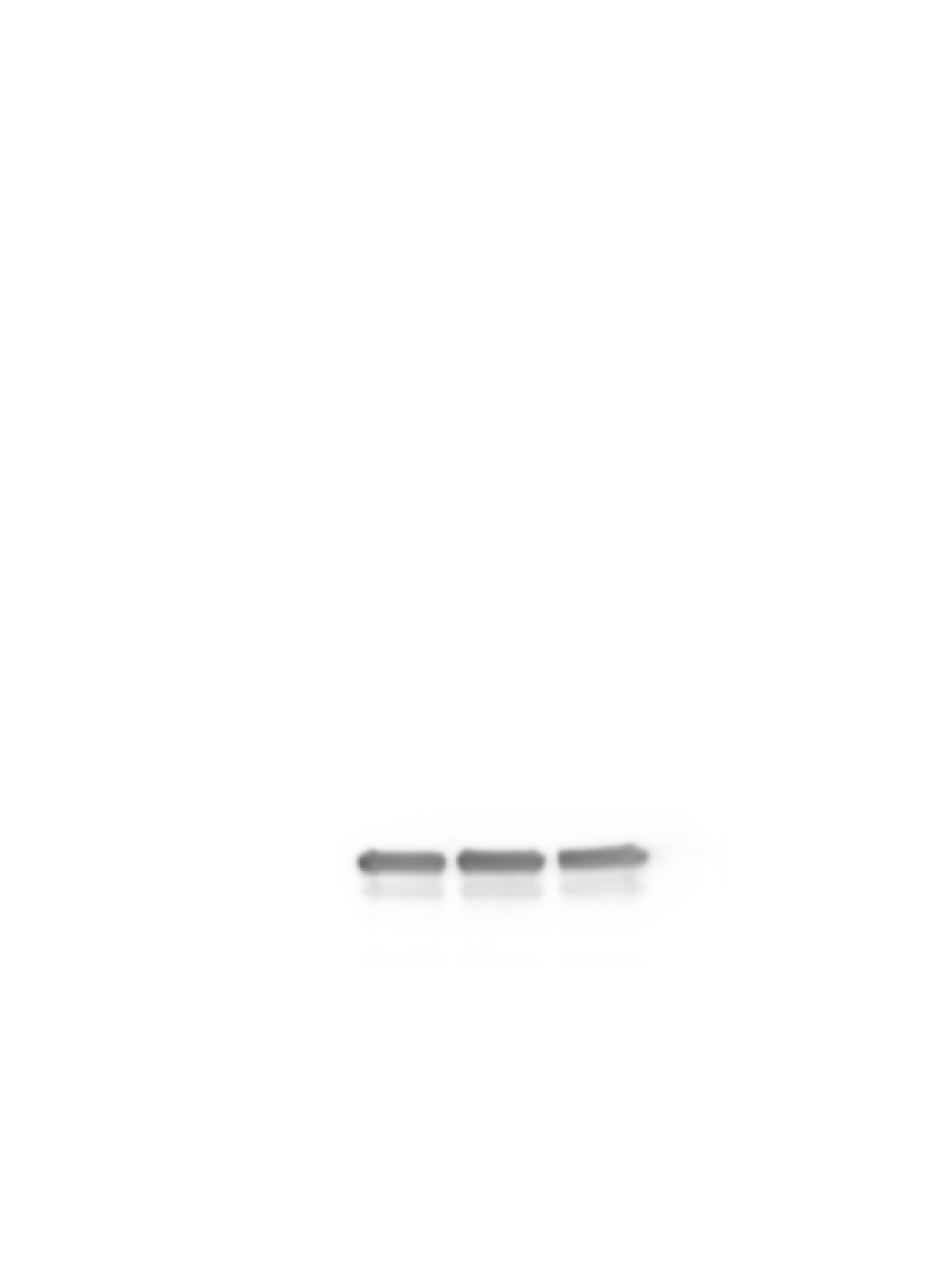

Supplement: Figure 1—figure supplement 1—source data 1. [file elife-105821-fig1-figsupp1-data1.zip › Figure 1-figure supplement 1-source data 1/Original files for western blot analysis displayed in Figure 1-figure supplement 1I/Tubulin 20230721_121244_Ch/Tubulin 20230721_121244_Ch_Chemi.tif]

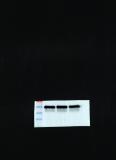

Supplement: Figure 1—figure supplement 1—source data 1. [file elife-105821-fig1-figsupp1-data1.zip › Figure 1-figure supplement 1-source data 1/Original files for western blot analysis displayed in Figure 1-figure supplement 1I/Tubulin 20230721_121244_Ch/Tubulin 20230721_121244_Ch_Thumb.jpg]

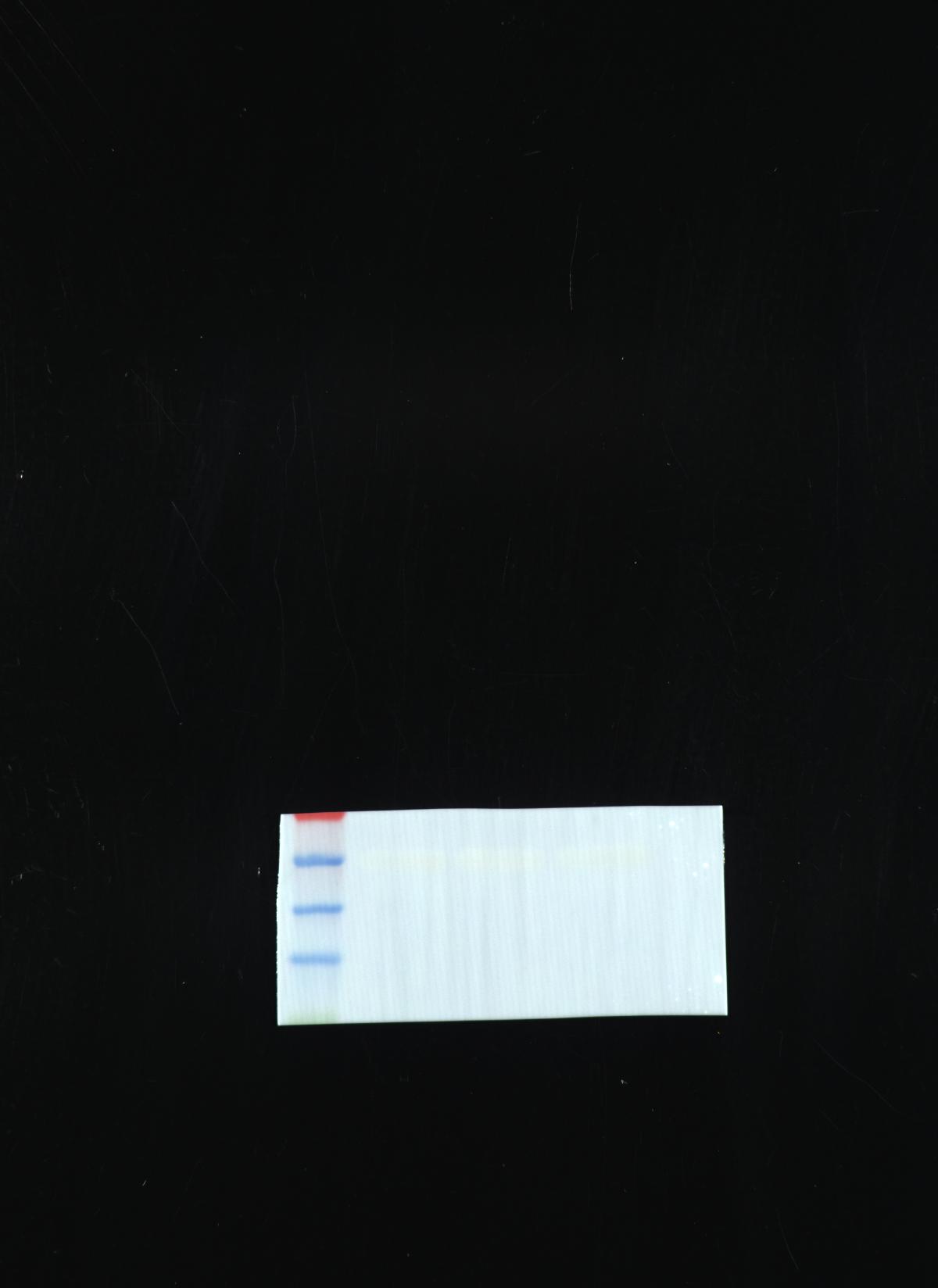

Supplement: Figure 1—figure supplement 1—source data 1. [file elife-105821-fig1-figsupp1-data1.zip › Figure 1-figure supplement 1-source data 1/Original files for western blot analysis displayed in Figure 1-figure supplement 1I/Tubulin 20230721_121244_Ch/Tubulin 20230721_121244_Ch-Marker.jpg]

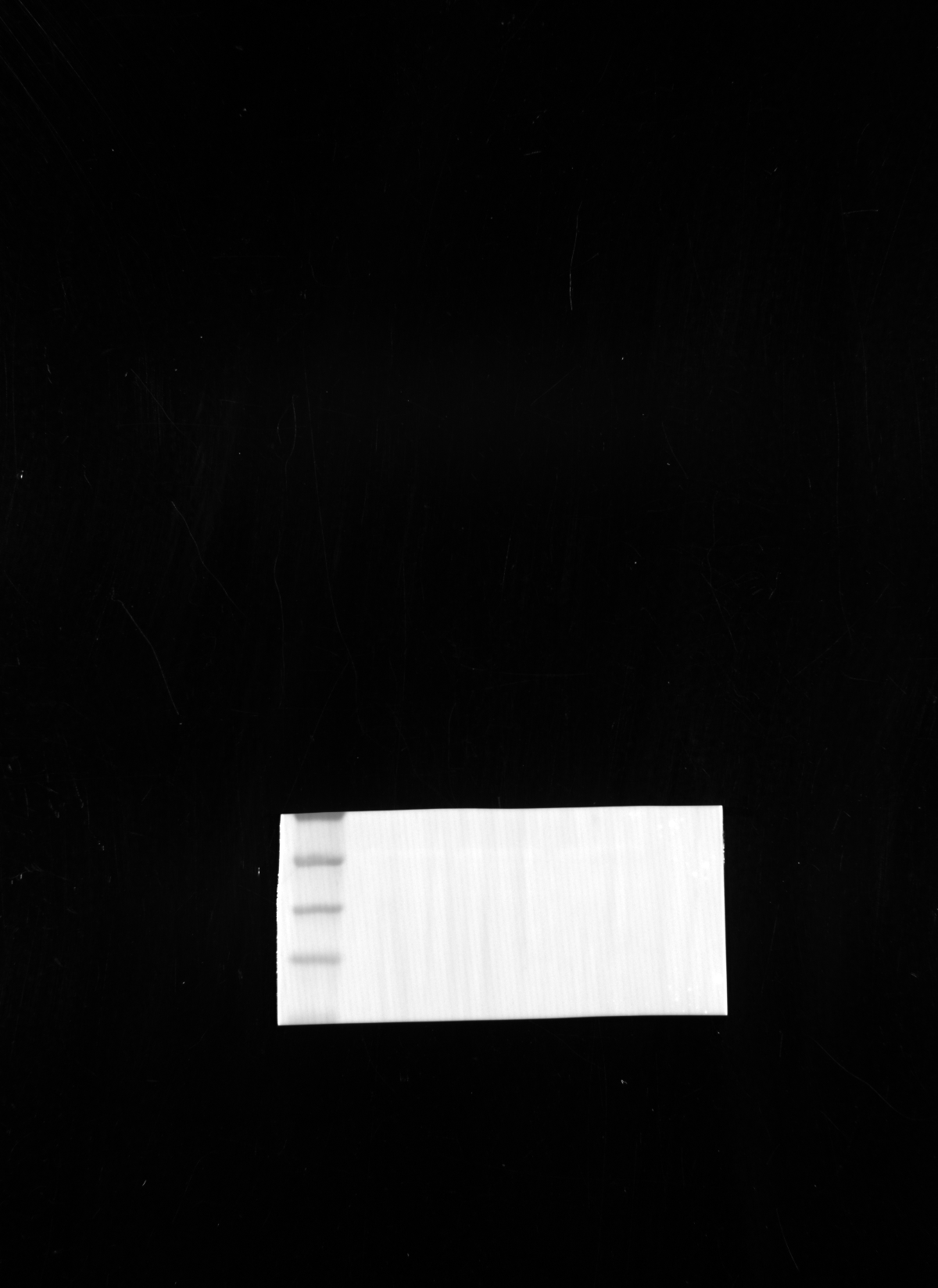

Supplement: Figure 1—figure supplement 1—source data 1. [file elife-105821-fig1-figsupp1-data1.zip › Figure 1-figure supplement 1-source data 1/Original files for western blot analysis displayed in Figure 1-figure supplement 1I/Tubulin 20230721_121244_Ch/Tubulin 20230721_121244_Ch-Marker.tif]

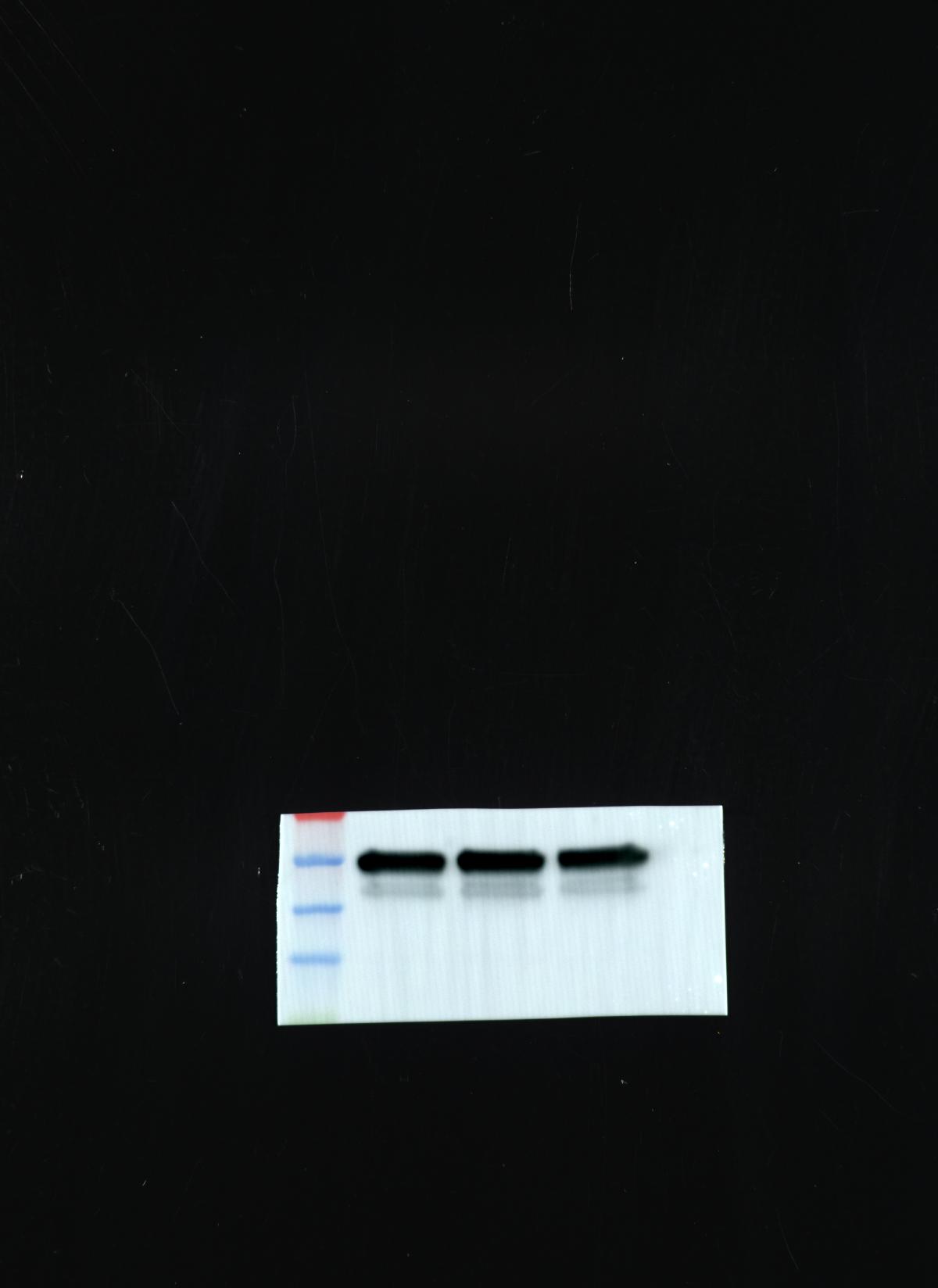

Supplement: Figure 1—figure supplement 1—source data 1. [file elife-105821-fig1-figsupp1-data1.zip › Figure 1-figure supplement 1-source data 1/Original files for western blot analysis displayed in Figure 1-figure supplement 1I/Tubulin 20230721_121244_Ch/Tubulin 20230721_121244_Ch_Chemi+Marker.jpg]

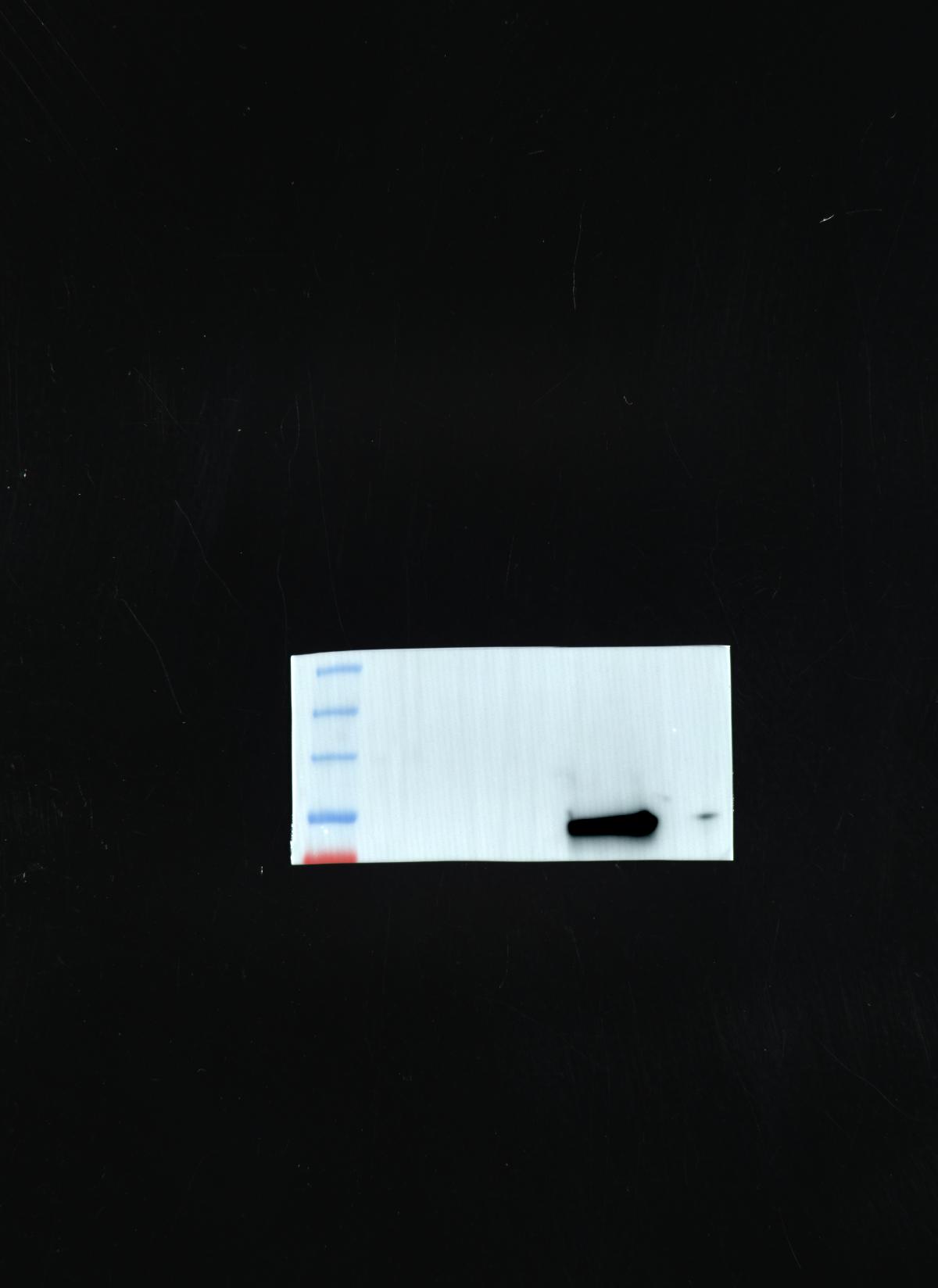

Supplement: Figure 1—figure supplement 1—source data 1. [file elife-105821-fig1-figsupp1-data1.zip › Figure 1-figure supplement 1-source data 1/Original files for western blot analysis displayed in Figure 1-figure supplement 1I/EA3 20230721_121536_Ch/EA3 20230721_121536_Ch_Chemi+Marker.jpg]

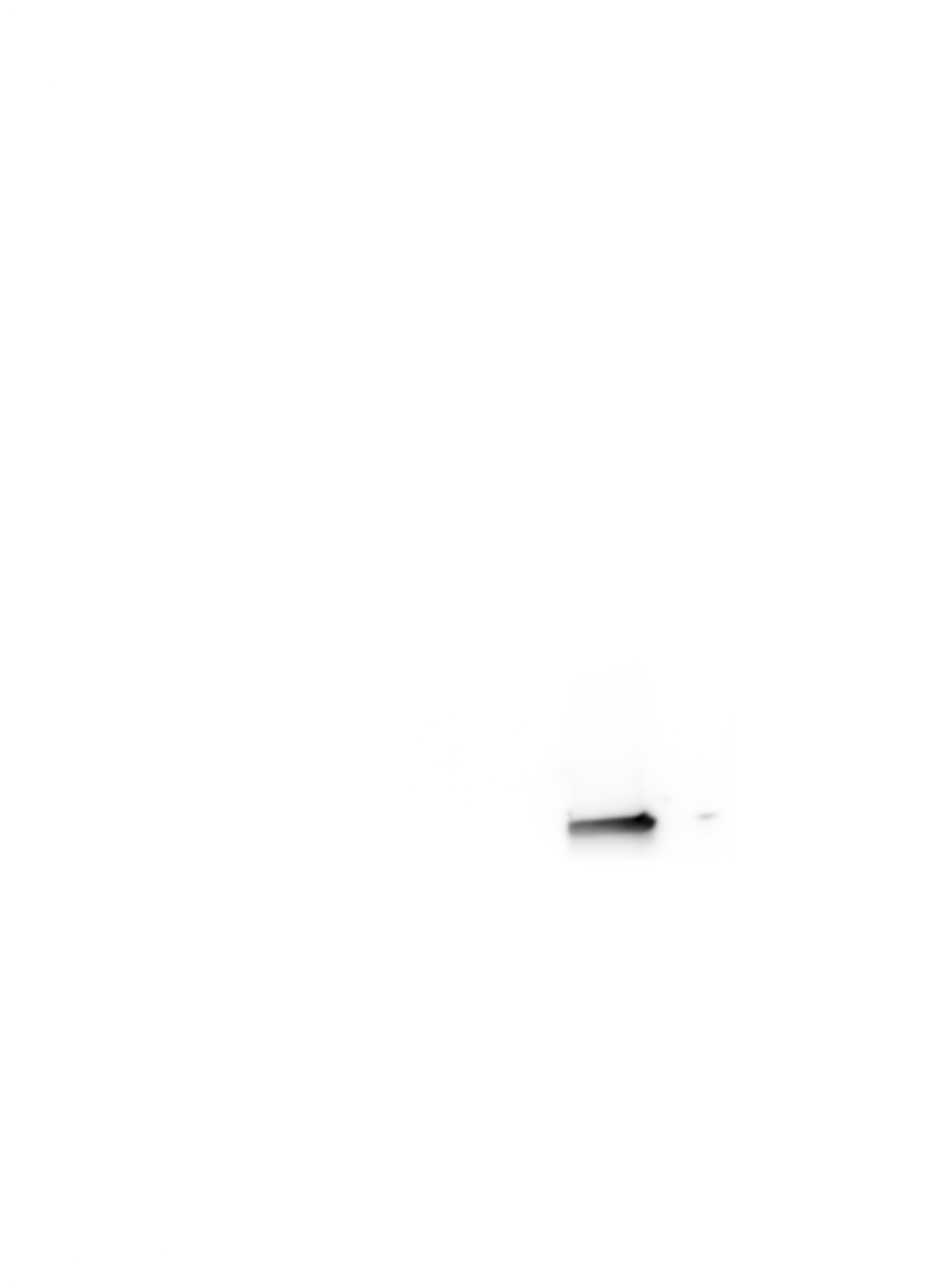

Supplement: Figure 1—figure supplement 1—source data 1. [file elife-105821-fig1-figsupp1-data1.zip › Figure 1-figure supplement 1-source data 1/Original files for western blot analysis displayed in Figure 1-figure supplement 1I/EA3 20230721_121536_Ch/EA3 20230721_121536_Ch_Chemi.tif]

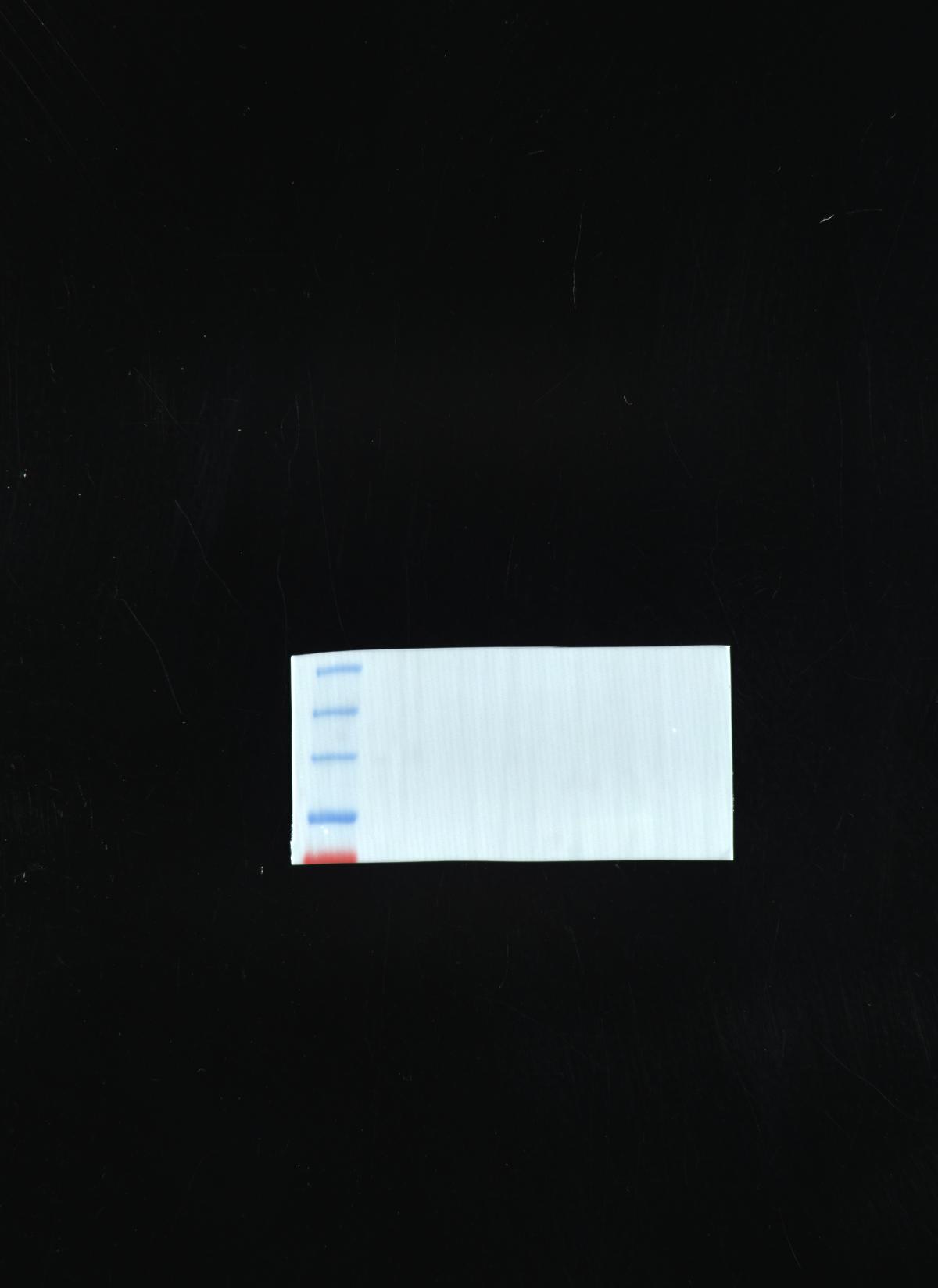

Supplement: Figure 1—figure supplement 1—source data 1. [file elife-105821-fig1-figsupp1-data1.zip › Figure 1-figure supplement 1-source data 1/Original files for western blot analysis displayed in Figure 1-figure supplement 1I/EA3 20230721_121536_Ch/EA3 20230721_121536_Ch-Marker.jpg]

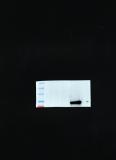

Supplement: Figure 1—figure supplement 1—source data 1. [file elife-105821-fig1-figsupp1-data1.zip › Figure 1-figure supplement 1-source data 1/Original files for western blot analysis displayed in Figure 1-figure supplement 1I/EA3 20230721_121536_Ch/EA3 20230721_121536_Ch_Thumb.jpg]

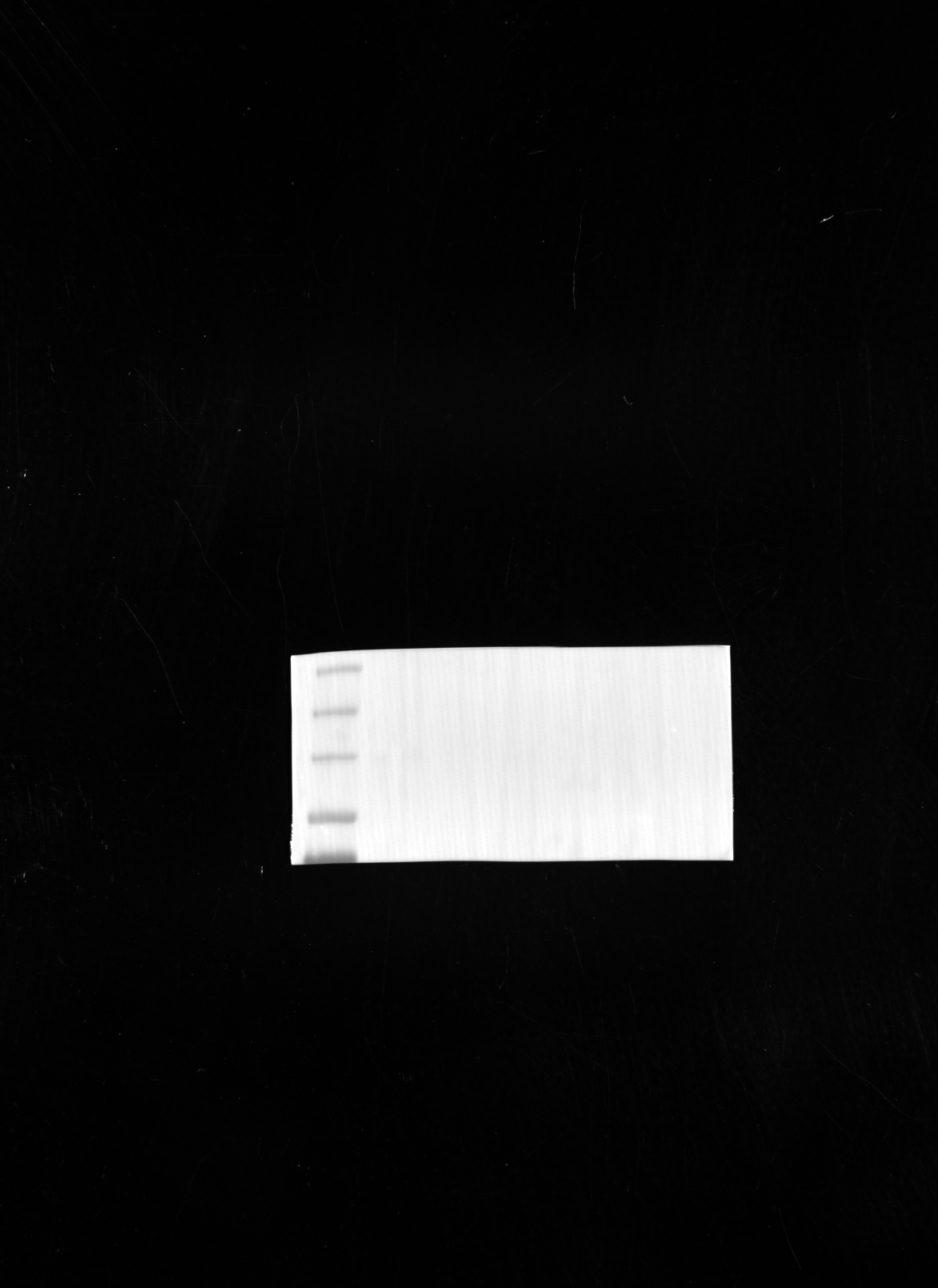

Supplement: Figure 1—figure supplement 1—source data 1. [file elife-105821-fig1-figsupp1-data1.zip › Figure 1-figure supplement 1-source data 1/Original files for western blot analysis displayed in Figure 1-figure supplement 1I/EA3 20230721_121536_Ch/EA3 20230721_121536_Ch-Marker.tif]

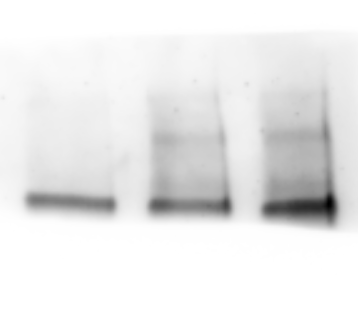

Supplement: Figure 1—figure supplement 1—source data 1. [file elife-105821-fig1-figsupp1-data1.zip › Figure 1-figure supplement 1-source data 1/Original files for western blot analysis displayed in Figure 1-figure supplement 1I/snap/SNAP crop RGB.tif]

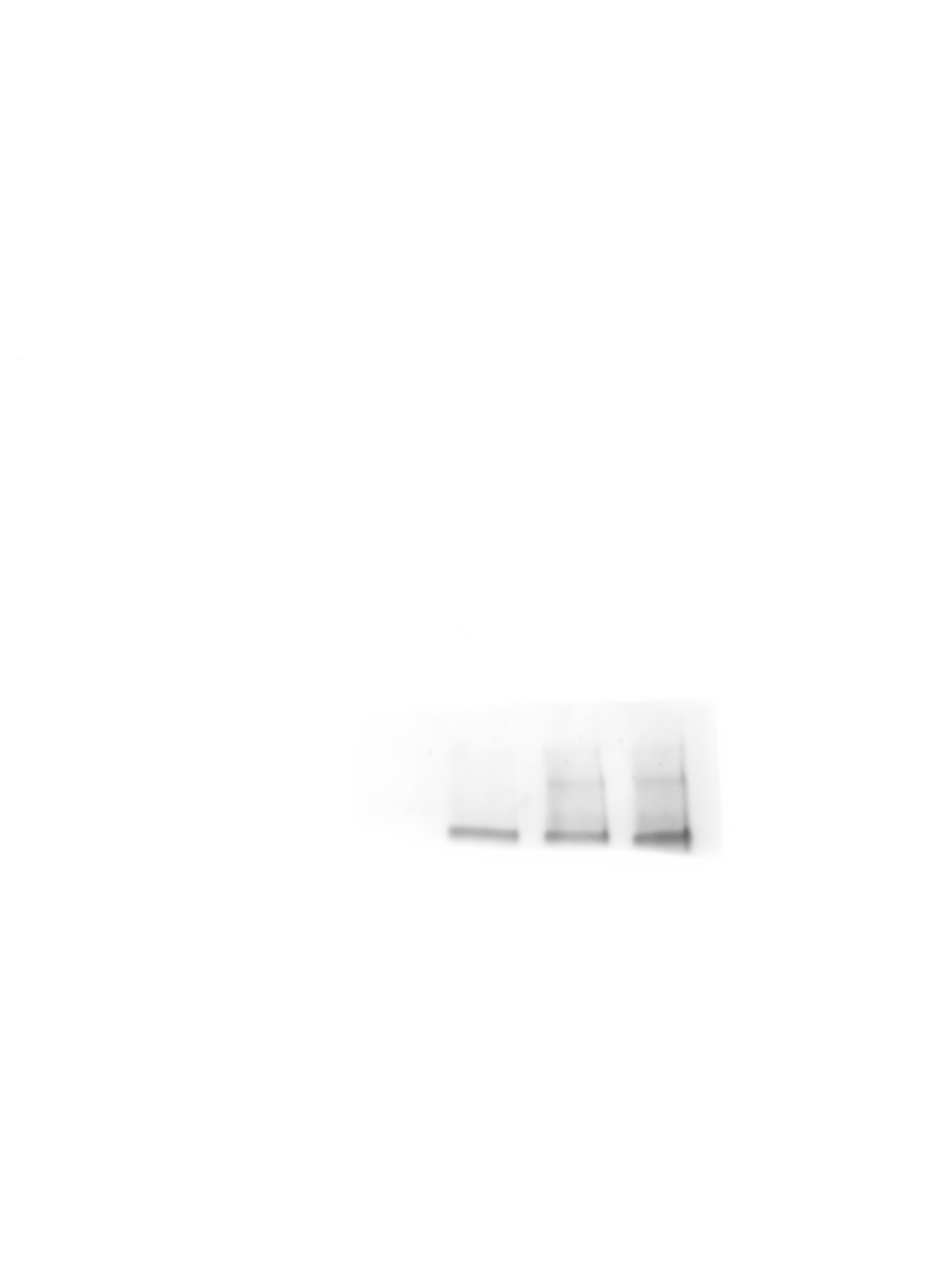

Supplement: Figure 1—figure supplement 1—source data 1. [file elife-105821-fig1-figsupp1-data1.zip › Figure 1-figure supplement 1-source data 1/Original files for western blot analysis displayed in Figure 1-figure supplement 1I/snap/SNAP 20230721_121822_Ch_Chemi.tif]

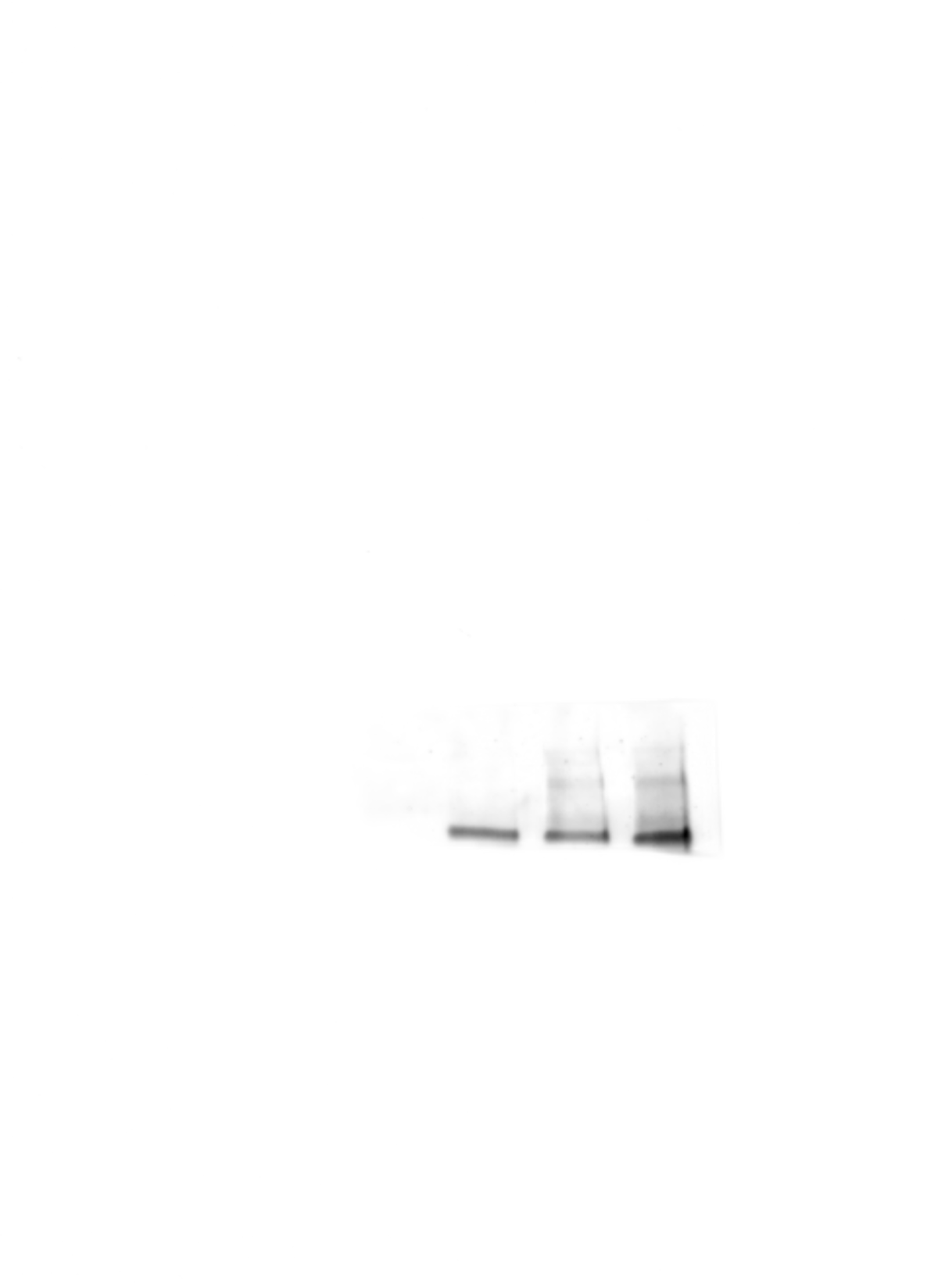

Supplement: Figure 1—figure supplement 1—source data 1. [file elife-105821-fig1-figsupp1-data1.zip › Figure 1-figure supplement 1-source data 1/Original files for western blot analysis displayed in Figure 1-figure supplement 1I/snap/SNAP RGB.tif]

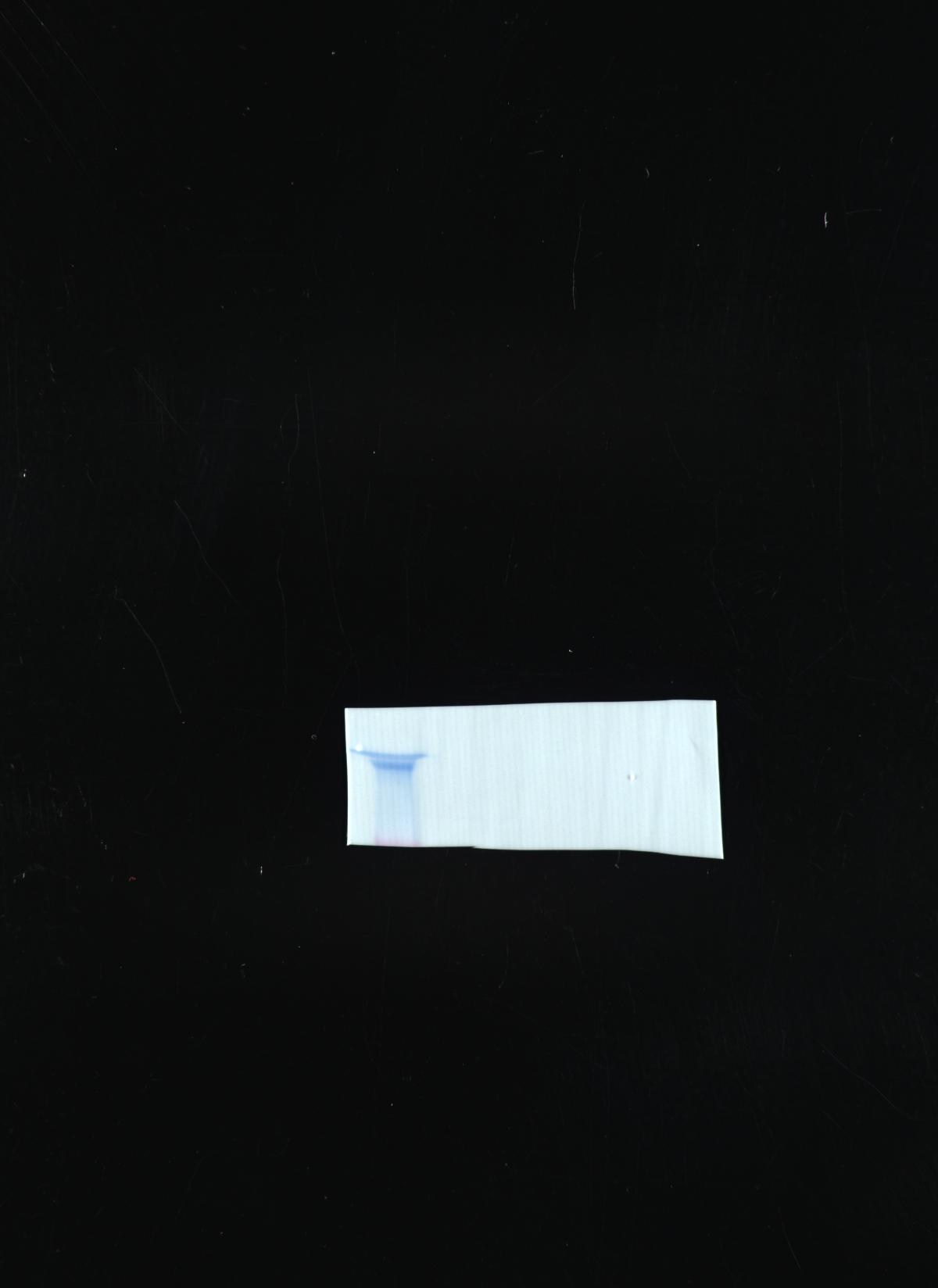

Supplement: Figure 1—figure supplement 1—source data 1. [file elife-105821-fig1-figsupp1-data1.zip › Figure 1-figure supplement 1-source data 1/Original files for western blot analysis displayed in Figure 1-figure supplement 1I/snap/SNAP 20230721_121822_Ch-Marker.jpg]

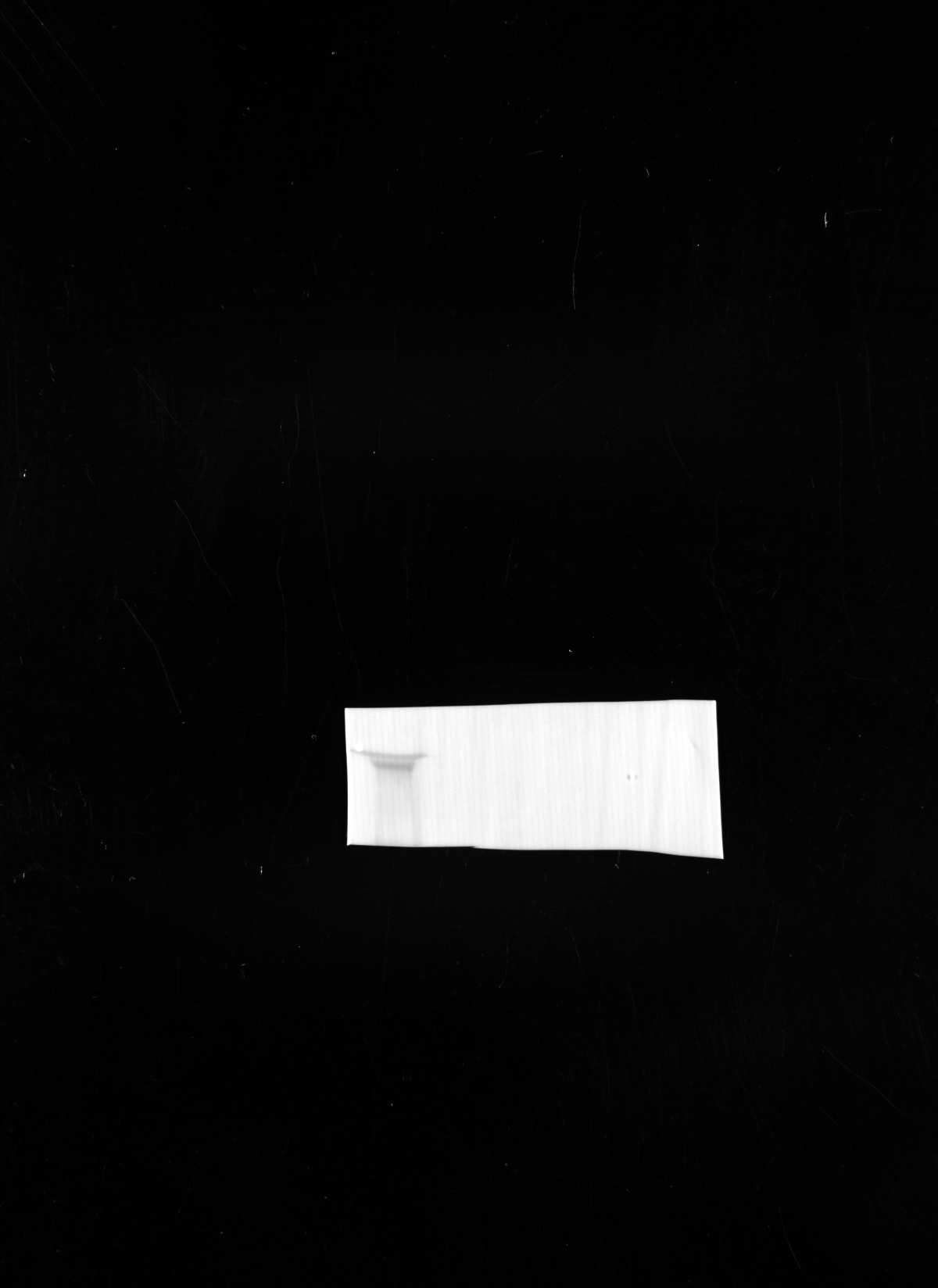

Supplement: Figure 1—figure supplement 1—source data 1. [file elife-105821-fig1-figsupp1-data1.zip › Figure 1-figure supplement 1-source data 1/Original files for western blot analysis displayed in Figure 1-figure supplement 1I/snap/SNAP 20230721_121822_Ch-Marker.tif]

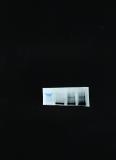

Supplement: Figure 1—figure supplement 1—source data 1. [file elife-105821-fig1-figsupp1-data1.zip › Figure 1-figure supplement 1-source data 1/Original files for western blot analysis displayed in Figure 1-figure supplement 1I/snap/SNAP 20230721_121822_Ch_Thumb.jpg]

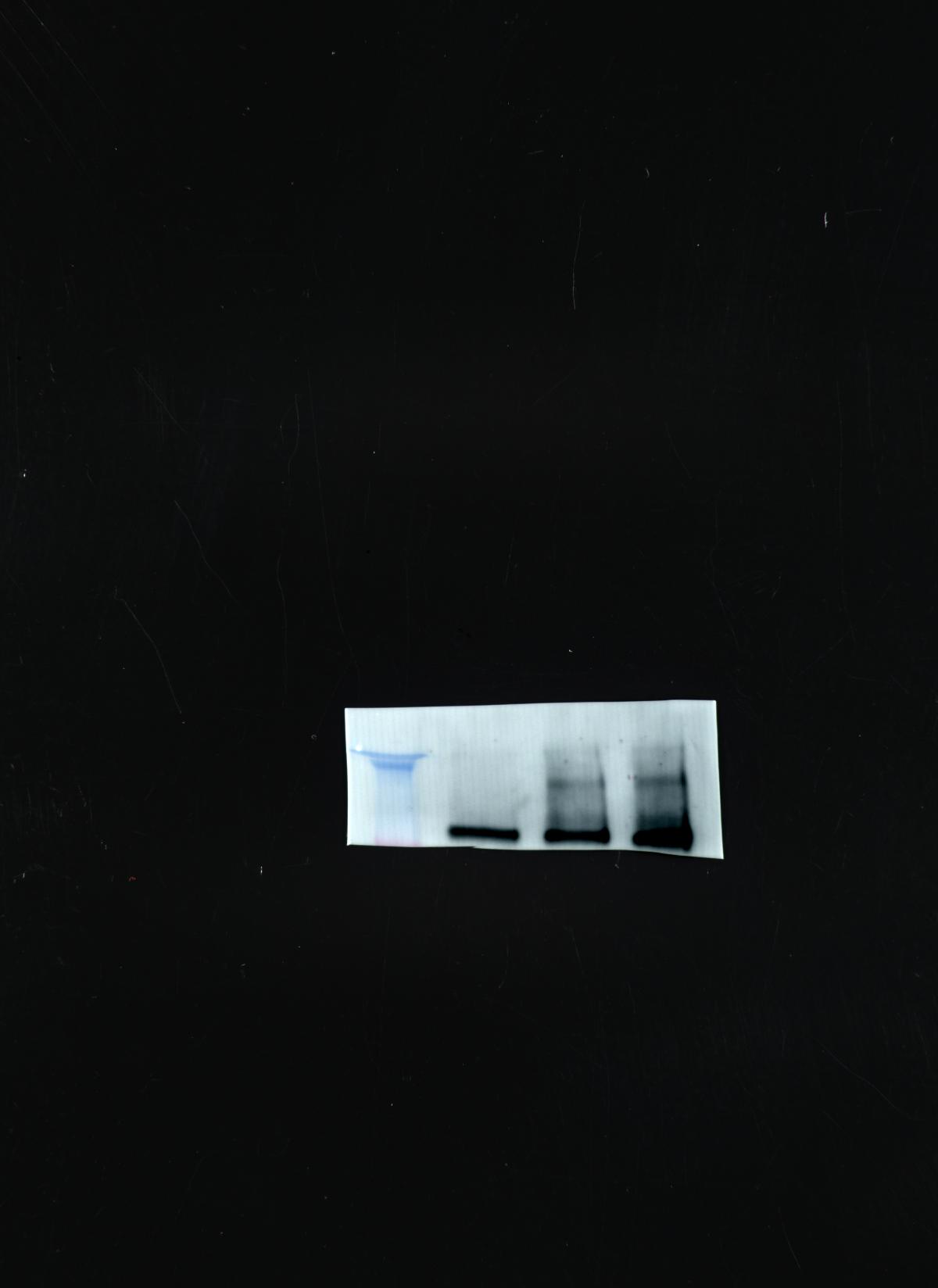

Supplement: Figure 1—figure supplement 1—source data 1. [file elife-105821-fig1-figsupp1-data1.zip › Figure 1-figure supplement 1-source data 1/Original files for western blot analysis displayed in Figure 1-figure supplement 1I/snap/SNAP 20230721_121822_Ch_Chemi+Marker.jpg]

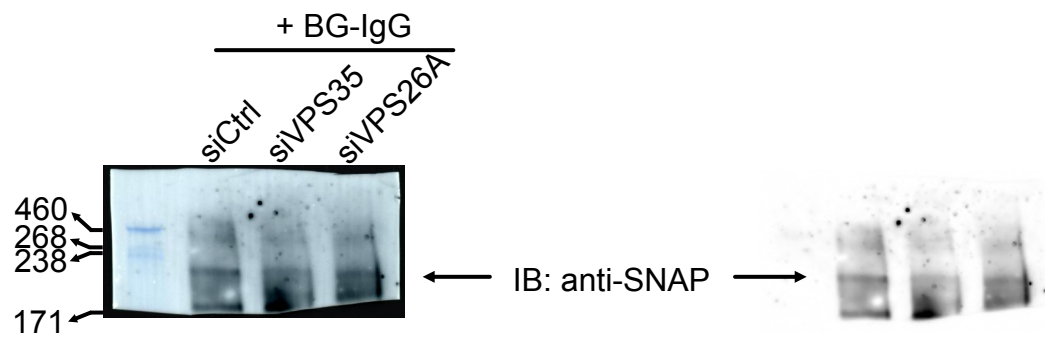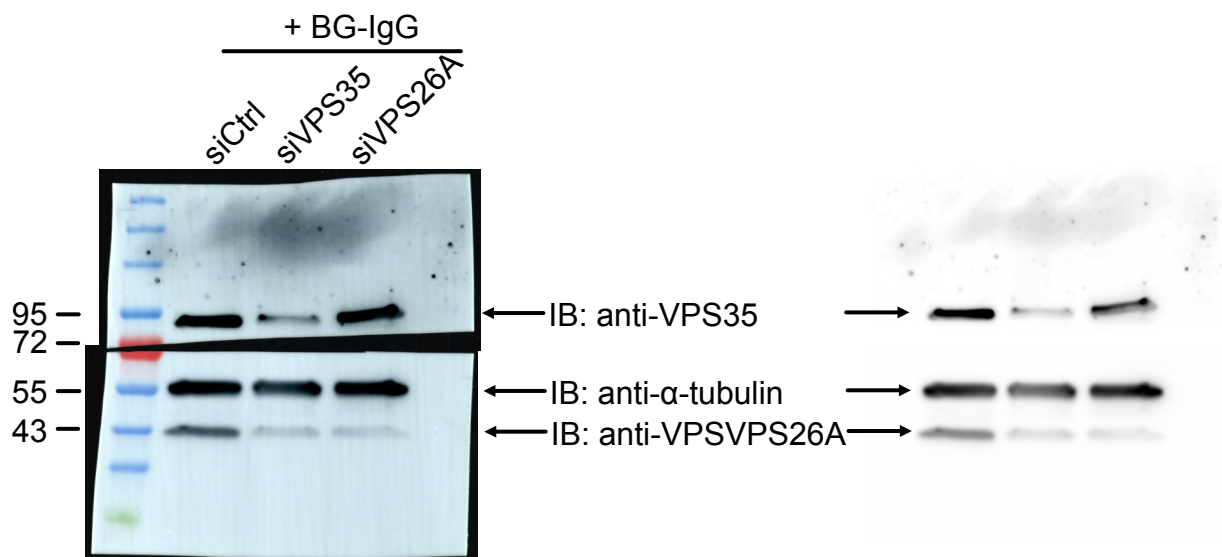

Supplement: Figure 1—figure supplement 1—source data 2. [file elife-105821-fig1-figsupp1-data2.zip › Figure 1-figure supplement 1-source data 2/PDF file containing original western blots for Figure 1-figure supplement 1D.pdf]

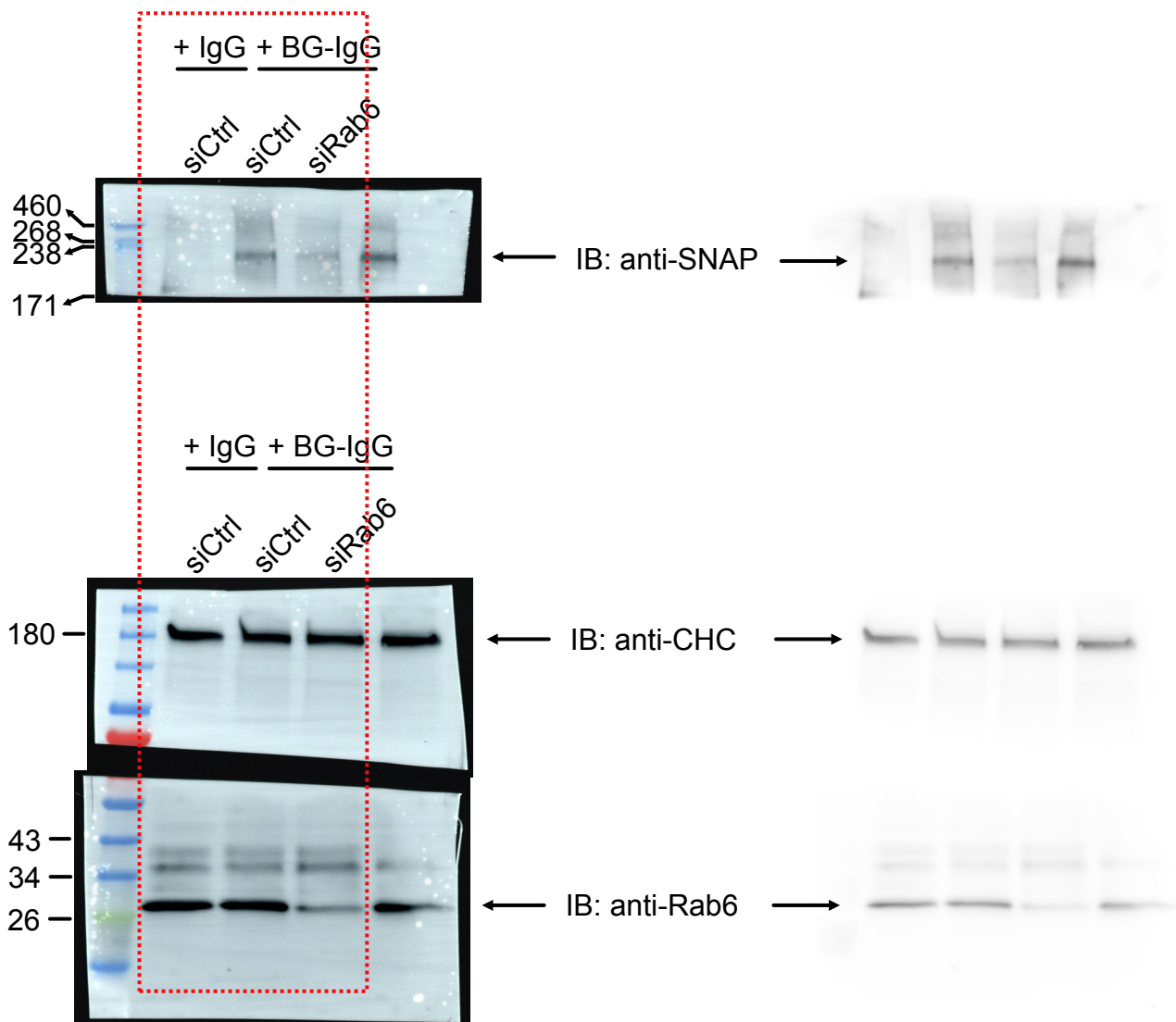

**Blots in the red frame were used in the manuscript.**

Supplement: Figure 1—figure supplement 1—source data 2. [file elife-105821-fig1-figsupp1-data2.zip › Figure 1-figure supplement 1-source data 2/PDF file containing original western blots for Figure 1-figure supplement 1F.pdf]

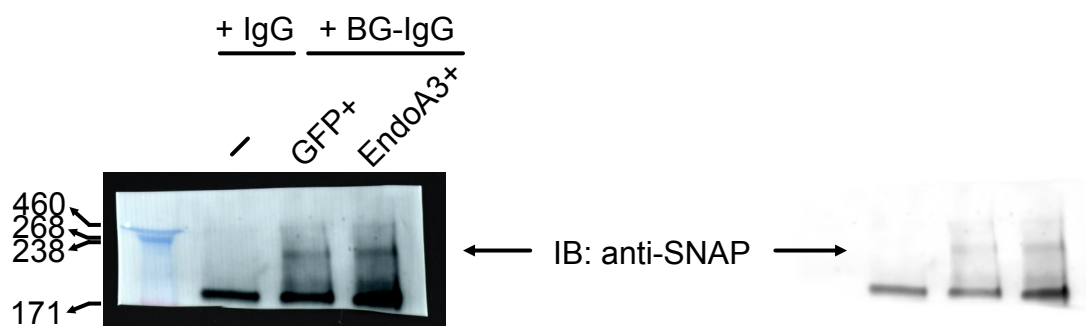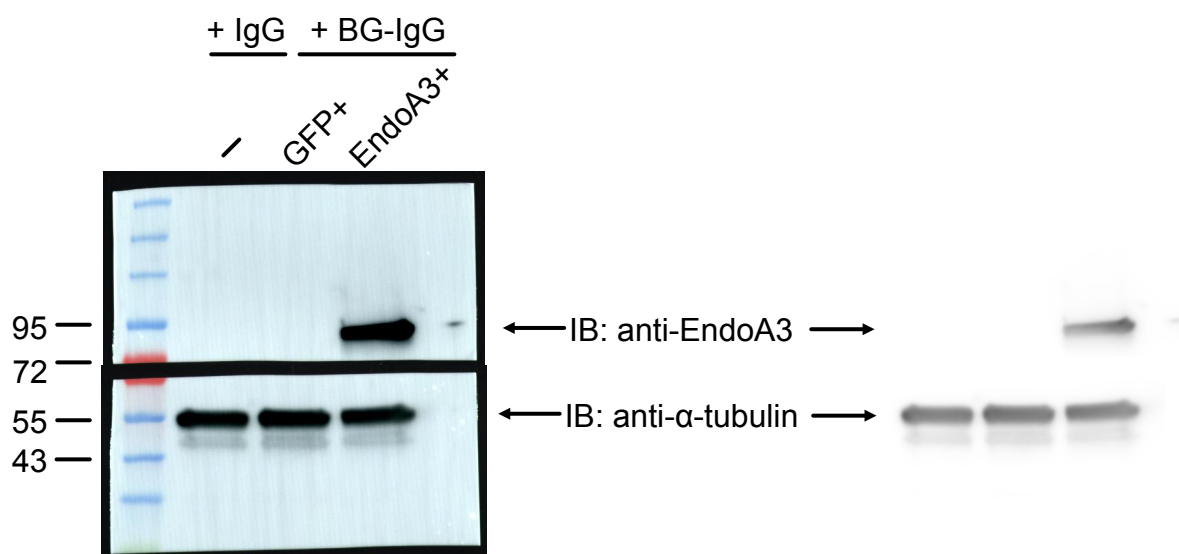

Supplement: Figure 1—figure supplement 1—source data 2. [file elife-105821-fig1-figsupp1-data2.zip › Figure 1-figure supplement 1-source data 2/PDF file containing original western blots for Figure 1-figure supplement 1I.pdf]

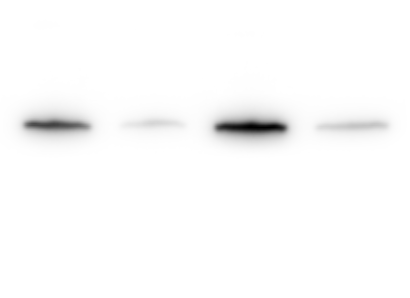

Supplement: Figure 2—figure supplement 2—source data 1. [file elife-105821-fig2-figsupp2-data1.zip › Figure 2-figure supplement 2-source data 1/Original files for western blot analysis displayed in Figure 2-figure supplement 2C/endoA3 1 2022.06.14_15.08.59_Ch/endoA3 1 2022.06.14_15.08.59_Ch.png]

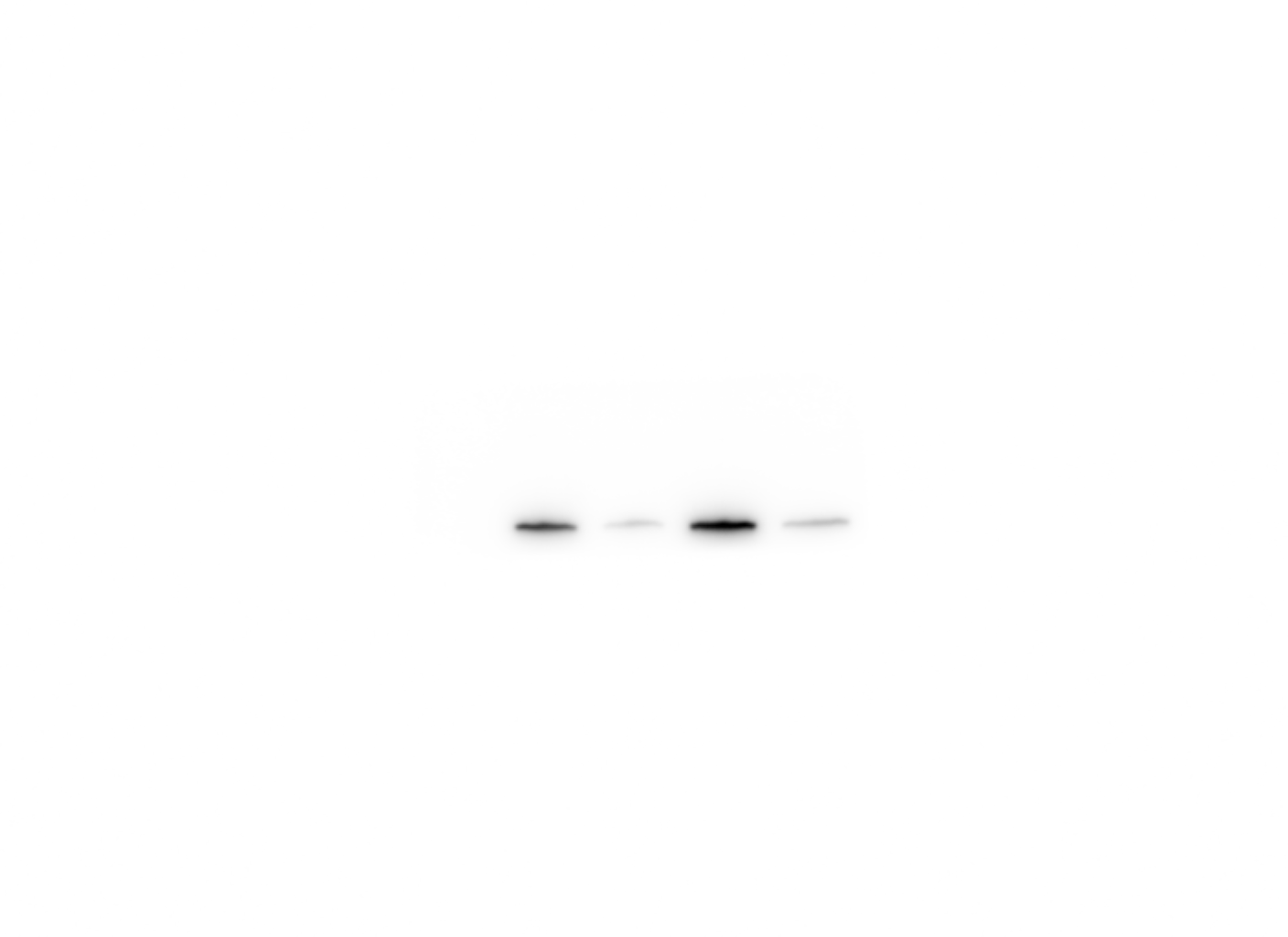

Supplement: Figure 2—figure supplement 2—source data 1. [file elife-105821-fig2-figsupp2-data1.zip › Figure 2-figure supplement 2-source data 1/Original files for western blot analysis displayed in Figure 2-figure supplement 2C/endoA3 1 2022.06.14_15.08.59_Ch/endoA3 1 2022.06.14_15.08.59_Ch.tif]

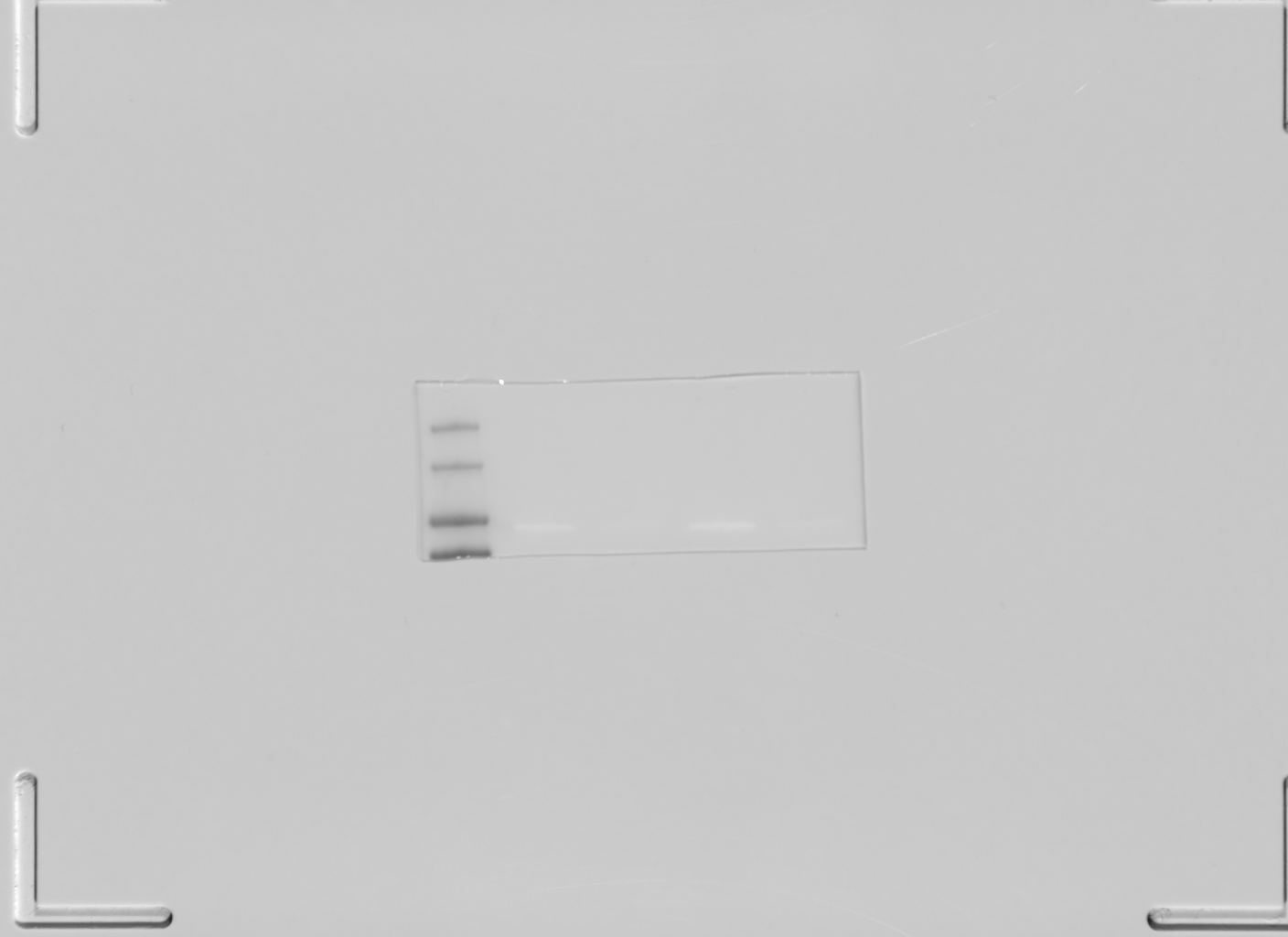

Supplement: Figure 2—figure supplement 2—source data 1. [file elife-105821-fig2-figsupp2-data1.zip › Figure 2-figure supplement 2-source data 1/Original files for western blot analysis displayed in Figure 2-figure supplement 2C/endoA3 1 2022.06.14_15.08.59_Ch/endoA3 1 2022.06.14_15.08.59_Ch-Marker.tif]

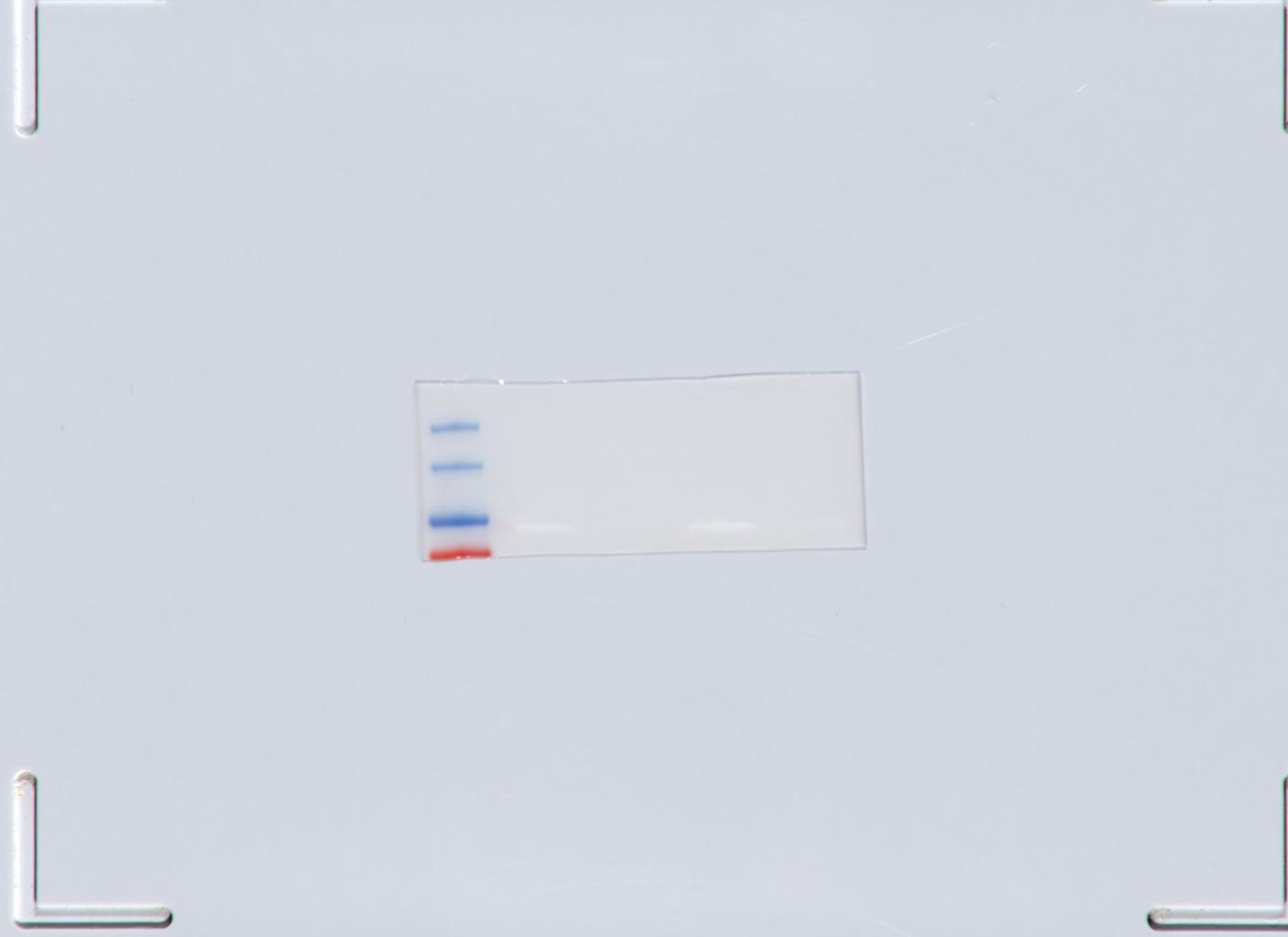

Supplement: Figure 2—figure supplement 2—source data 1. [file elife-105821-fig2-figsupp2-data1.zip › Figure 2-figure supplement 2-source data 1/Original files for western blot analysis displayed in Figure 2-figure supplement 2C/endoA3 1 2022.06.14_15.08.59_Ch/endoA3 1 2022.06.14_15.08.59_Ch-Marker.jpg]

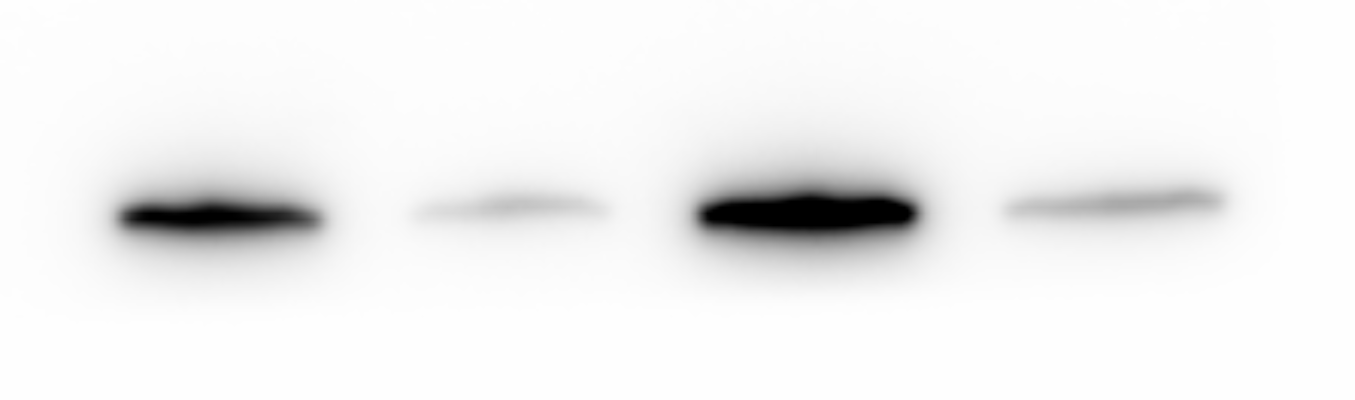

Supplement: Figure 2—figure supplement 2—source data 1. [file elife-105821-fig2-figsupp2-data1.zip › Figure 2-figure supplement 2-source data 1/Original files for western blot analysis displayed in Figure 2-figure supplement 2C/endoA3 1 2022.06.14_15.08.59_Ch/Fig EV1 WB.tif]

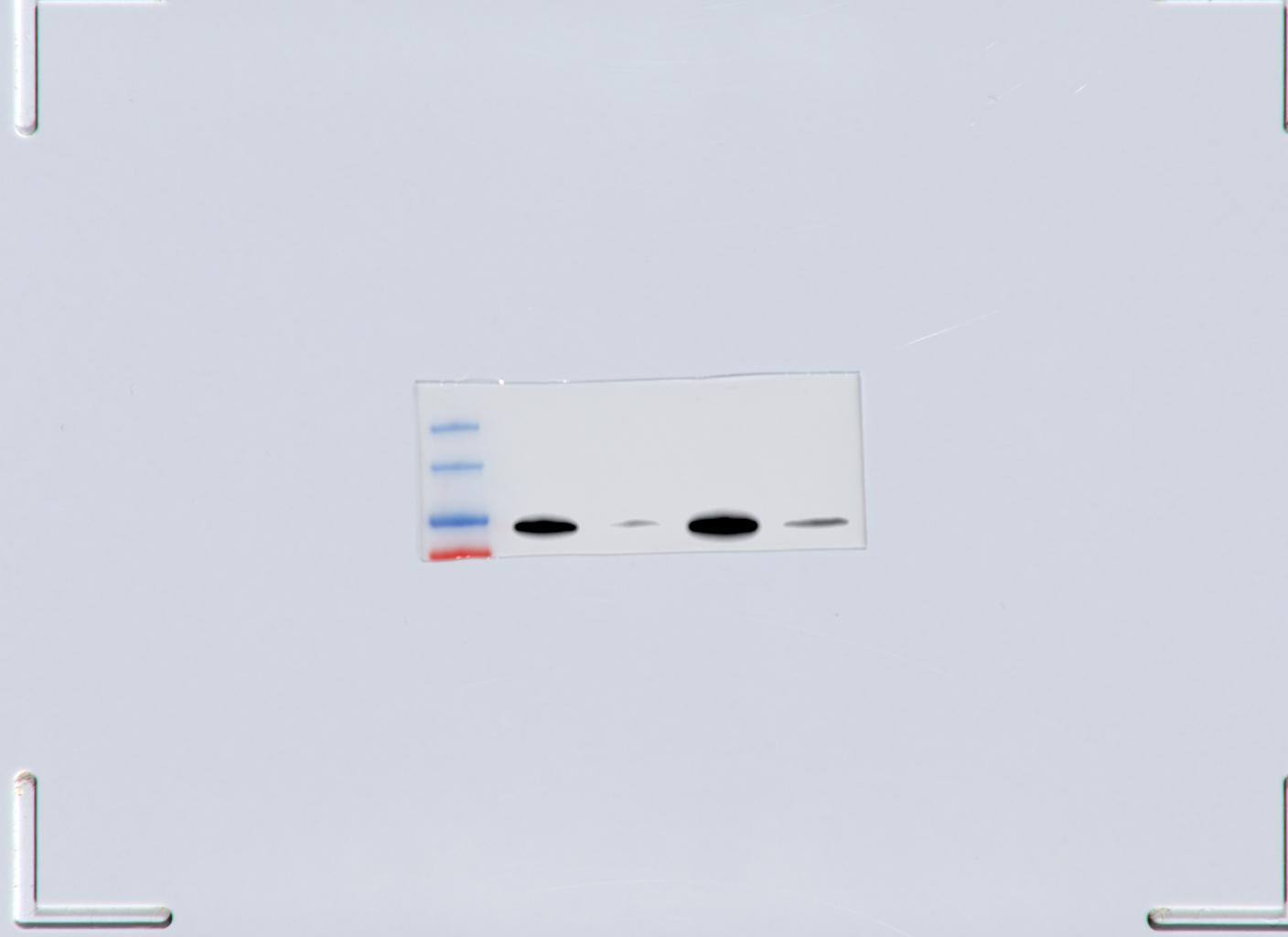

Supplement: Figure 2—figure supplement 2—source data 1. [file elife-105821-fig2-figsupp2-data1.zip › Figure 2-figure supplement 2-source data 1/Original files for western blot analysis displayed in Figure 2-figure supplement 2C/endoA3 1 2022.06.14_15.08.59_Ch/endoA3 1 2022.06.14_15.08.59_Ch+Marker.jpg]

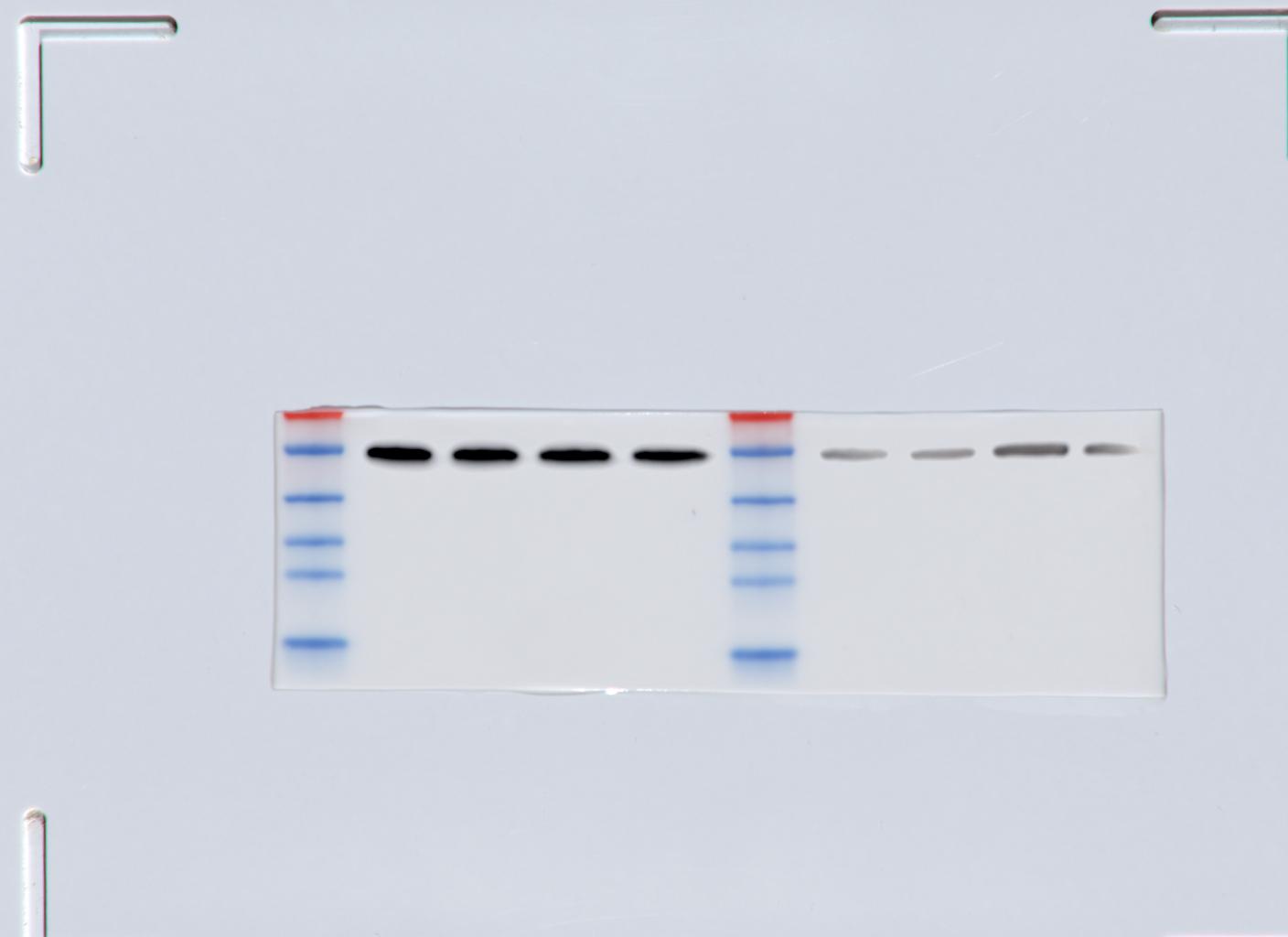

Supplement: Figure 2—figure supplement 2—source data 1. [file elife-105821-fig2-figsupp2-data1.zip › Figure 2-figure supplement 2-source data 1/Original files for western blot analysis displayed in Figure 2-figure supplement 2C/tubulin 1 2022.06.14_15.04.44_Ch/tubulin 1 2022.06.14_15.04.44_Ch+Marker.jpg]

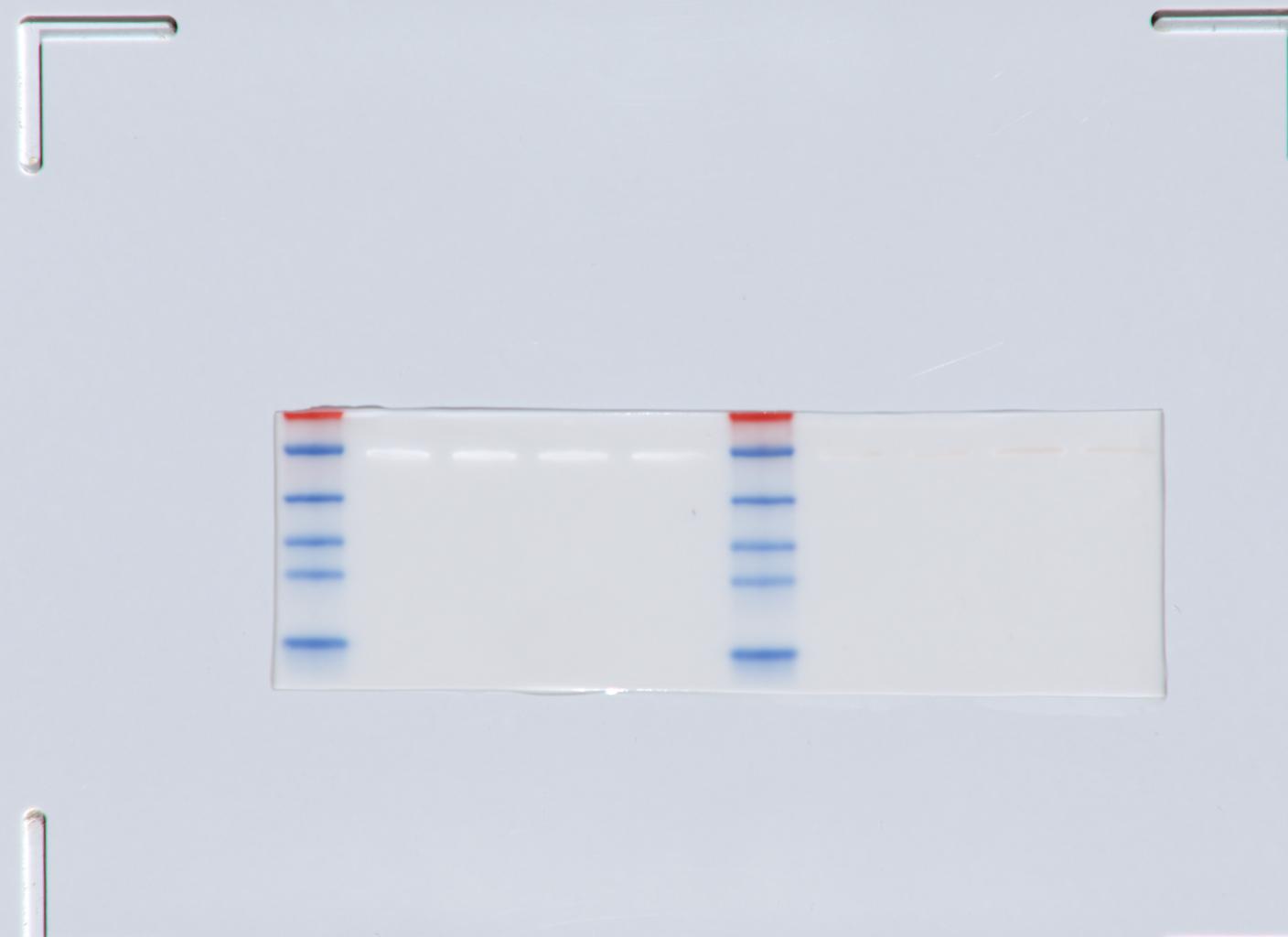

Supplement: Figure 2—figure supplement 2—source data 1. [file elife-105821-fig2-figsupp2-data1.zip › Figure 2-figure supplement 2-source data 1/Original files for western blot analysis displayed in Figure 2-figure supplement 2C/tubulin 1 2022.06.14_15.04.44_Ch/tubulin 1 2022.06.14_15.04.44_Ch-Marker.jpg]

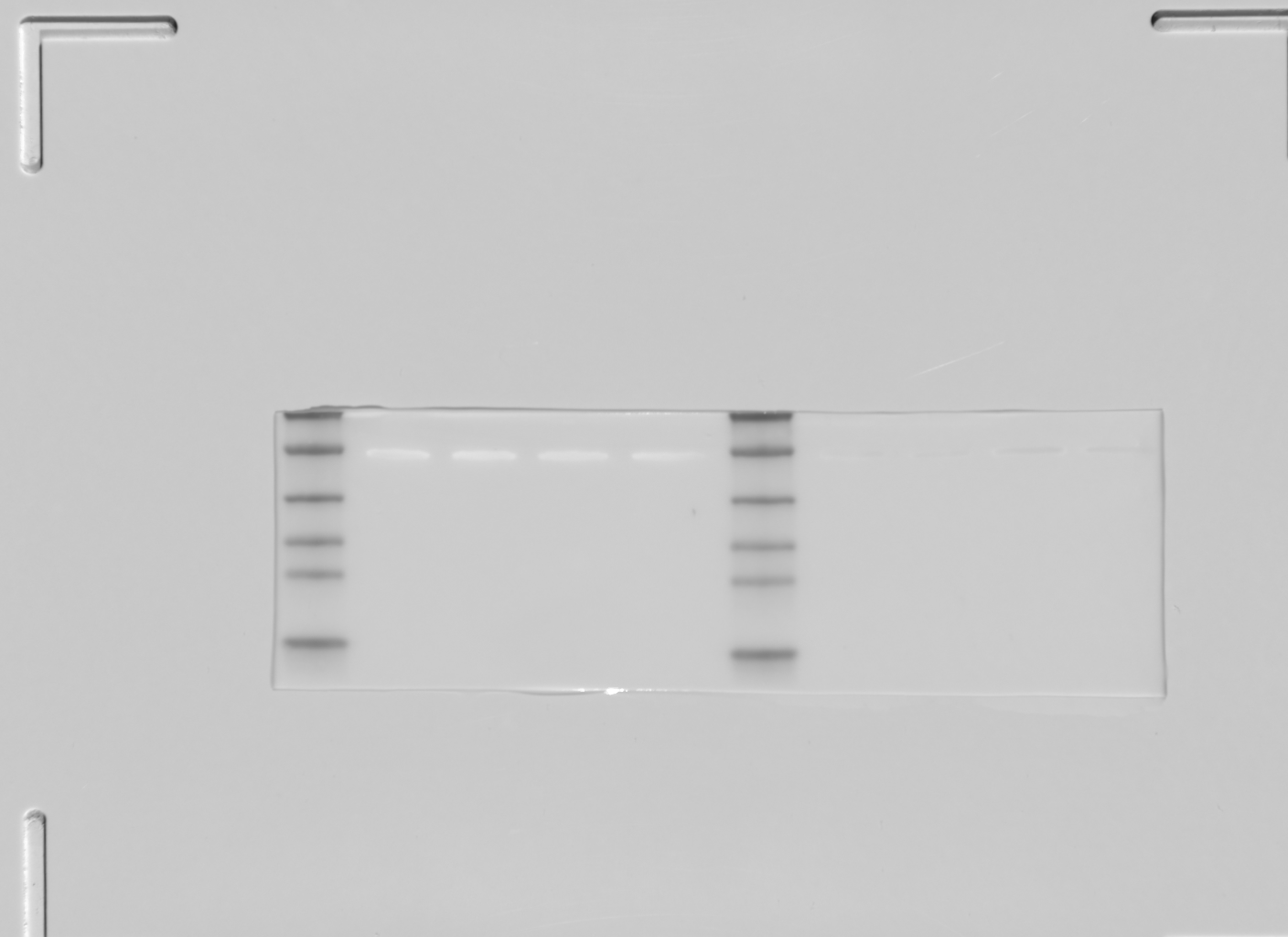

Supplement: Figure 2—figure supplement 2—source data 1. [file elife-105821-fig2-figsupp2-data1.zip › Figure 2-figure supplement 2-source data 1/Original files for western blot analysis displayed in Figure 2-figure supplement 2C/tubulin 1 2022.06.14_15.04.44_Ch/tubulin 1 2022.06.14_15.04.44_Ch-Marker.tif]

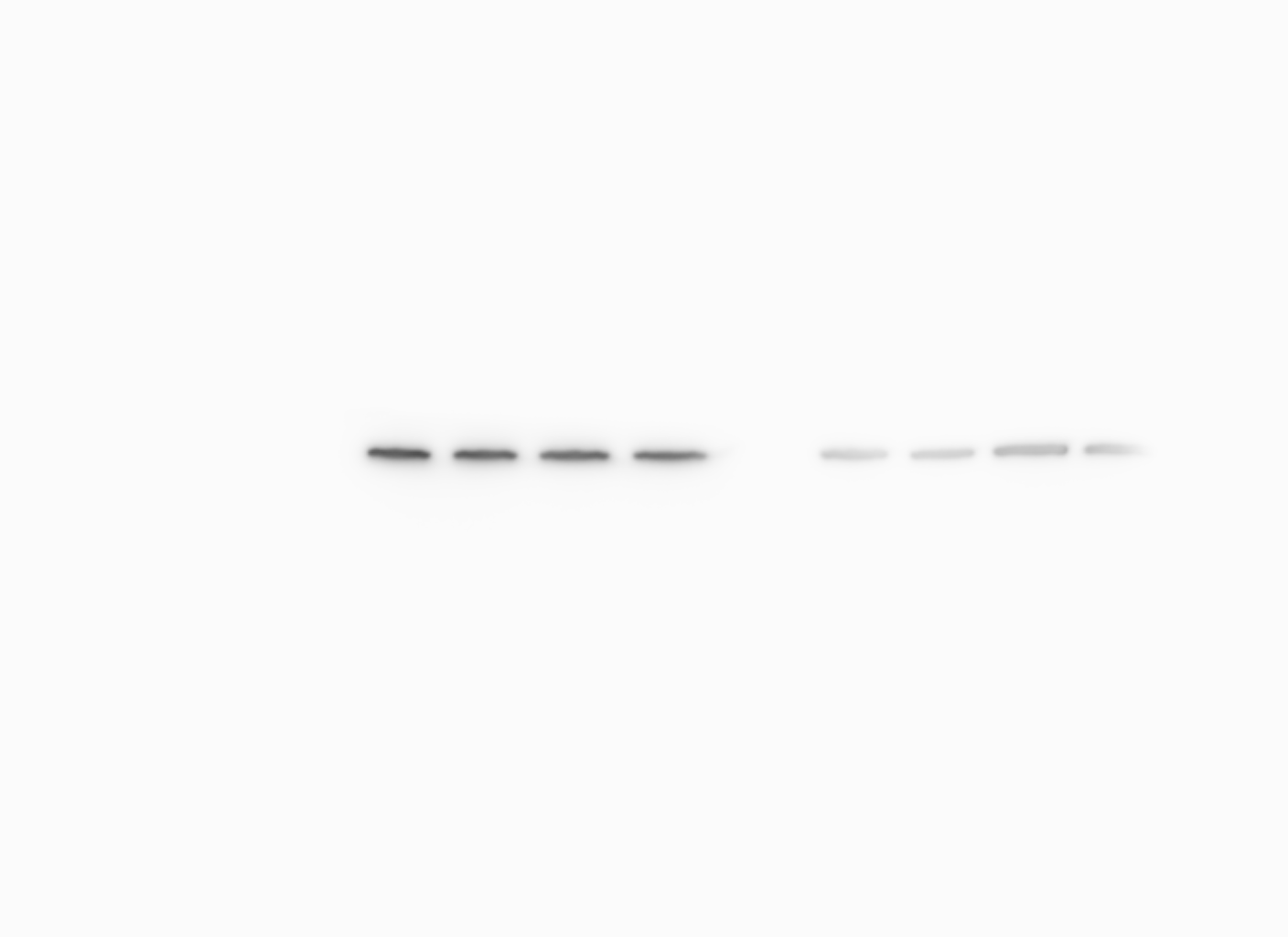

Supplement: Figure 2—figure supplement 2—source data 1. [file elife-105821-fig2-figsupp2-data1.zip › Figure 2-figure supplement 2-source data 1/Original files for western blot analysis displayed in Figure 2-figure supplement 2C/tubulin 1 2022.06.14_15.04.44_Ch/tubulin 1 2022.06.14_15.04.44_Ch.tif]

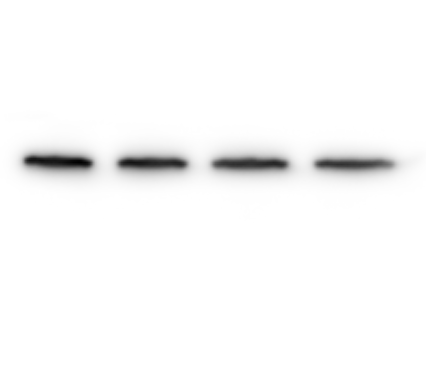

Supplement: Figure 2—figure supplement 2—source data 1. [file elife-105821-fig2-figsupp2-data1.zip › Figure 2-figure supplement 2-source data 1/Original files for western blot analysis displayed in Figure 2-figure supplement 2C/tubulin 1 2022.06.14_15.04.44_Ch/tubulin 1 2022.06.14_15.04.44_Ch.png]

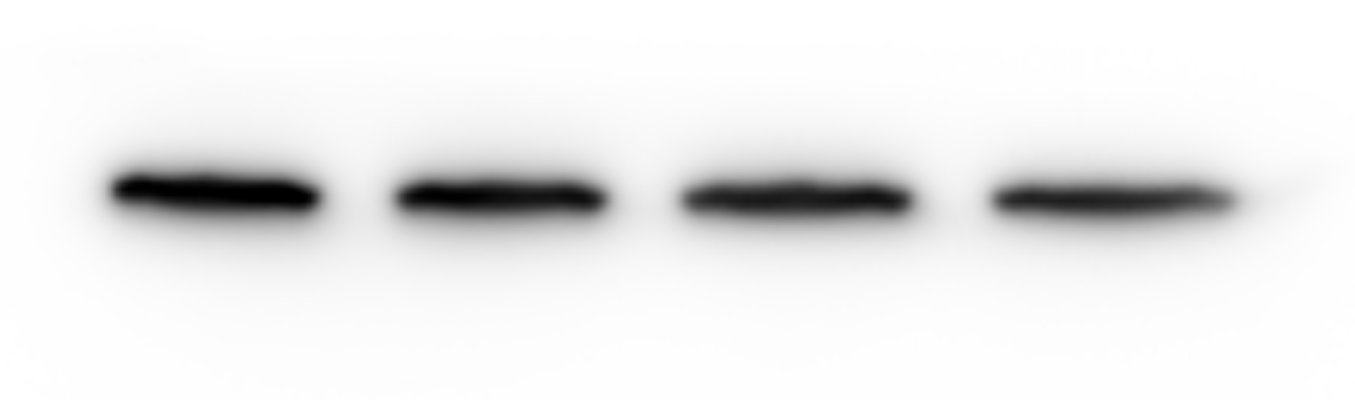

Supplement: Figure 2—figure supplement 2—source data 1. [file elife-105821-fig2-figsupp2-data1.zip › Figure 2-figure supplement 2-source data 1/Original files for western blot analysis displayed in Figure 2-figure supplement 2C/tubulin 1 2022.06.14_15.04.44_Ch/Fig EV1 WB.tif]

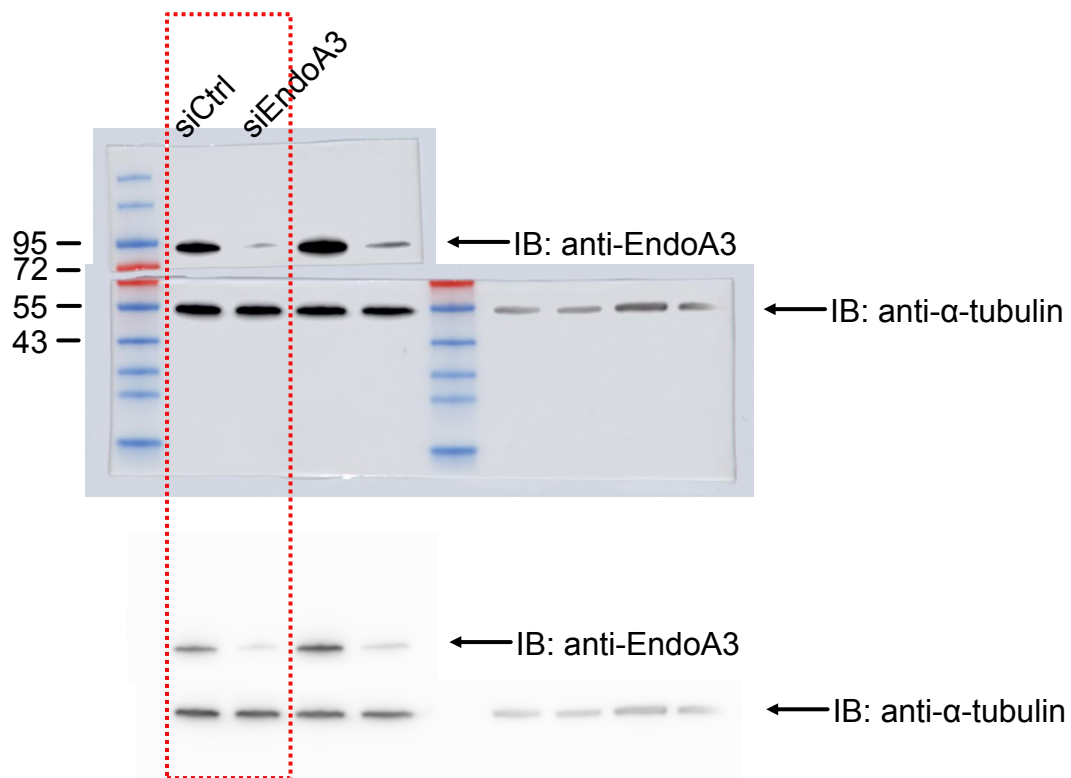

**Blots in the red frame were used in the manuscript.**

Supplement: Figure 2—figure supplement 2—source data 2. [file elife-105821-fig2-figsupp2-data2.zip › Figure 2-figure supplement 2-source data 2/PDF file containing original western blots for Figure 2-figure supplement 2C.pdf]

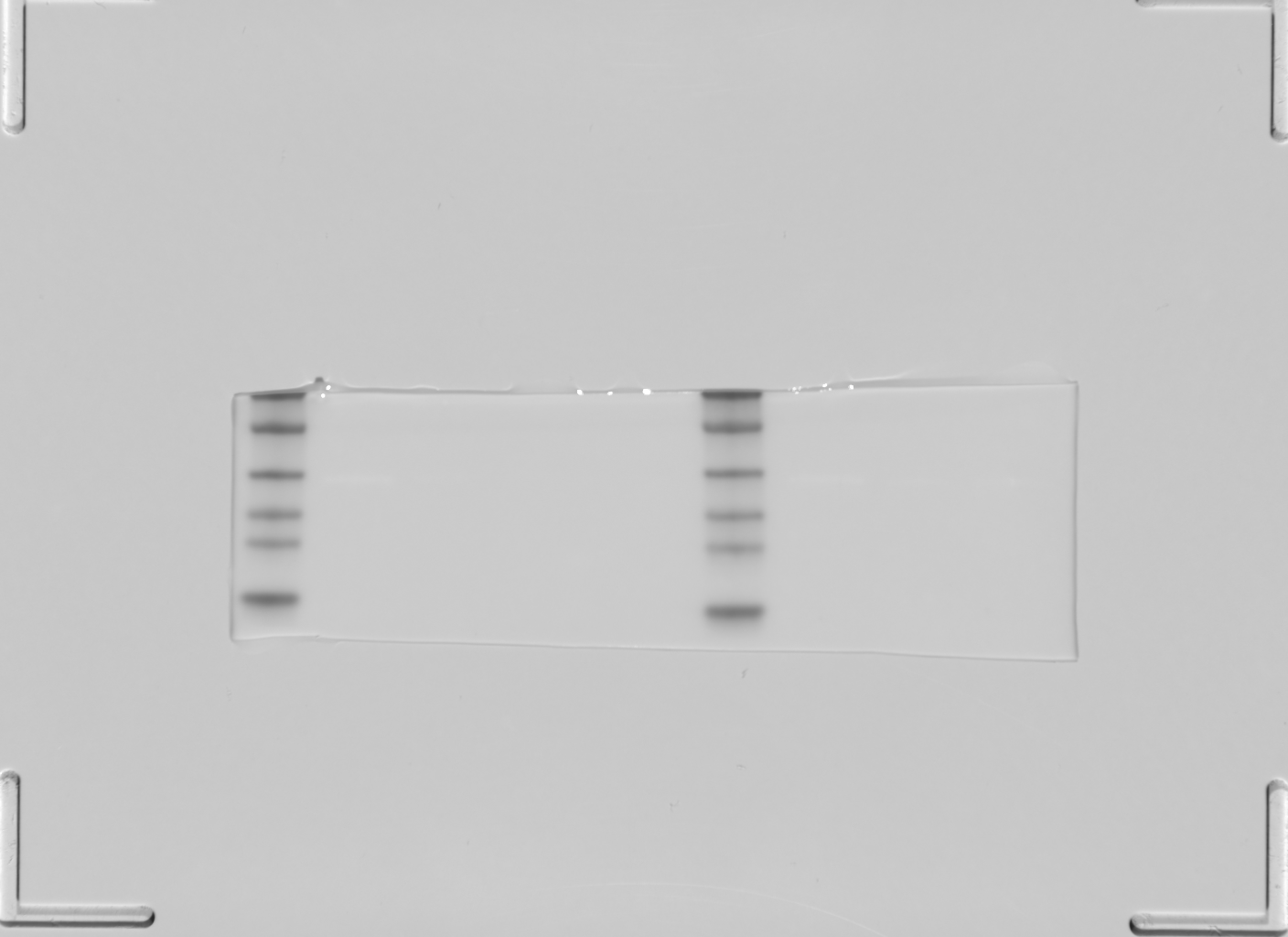

Supplement: Figure 3—figure supplement 1—source data 1. [file elife-105821-fig3-figsupp1-data1.zip › Figure 3-figure supplement 1-source data 1/Original files for western blot analysis displayed in Figure 3-figure supplement 1L/Vps26A 2 2022.05.31_16.25.40_Ch/Vps26A 2 2022.05.31_16.25.40_Ch-Marker.tif]

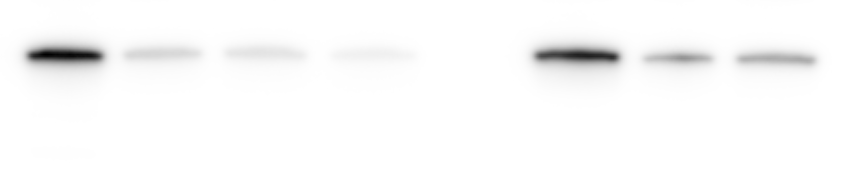

Supplement: Figure 3—figure supplement 1—source data 1. [file elife-105821-fig3-figsupp1-data1.zip › Figure 3-figure supplement 1-source data 1/Original files for western blot analysis displayed in Figure 3-figure supplement 1L/Vps26A 2 2022.05.31_16.25.40_Ch/Vps26A 2 2022.05.31_16.25.40_Ch.png]

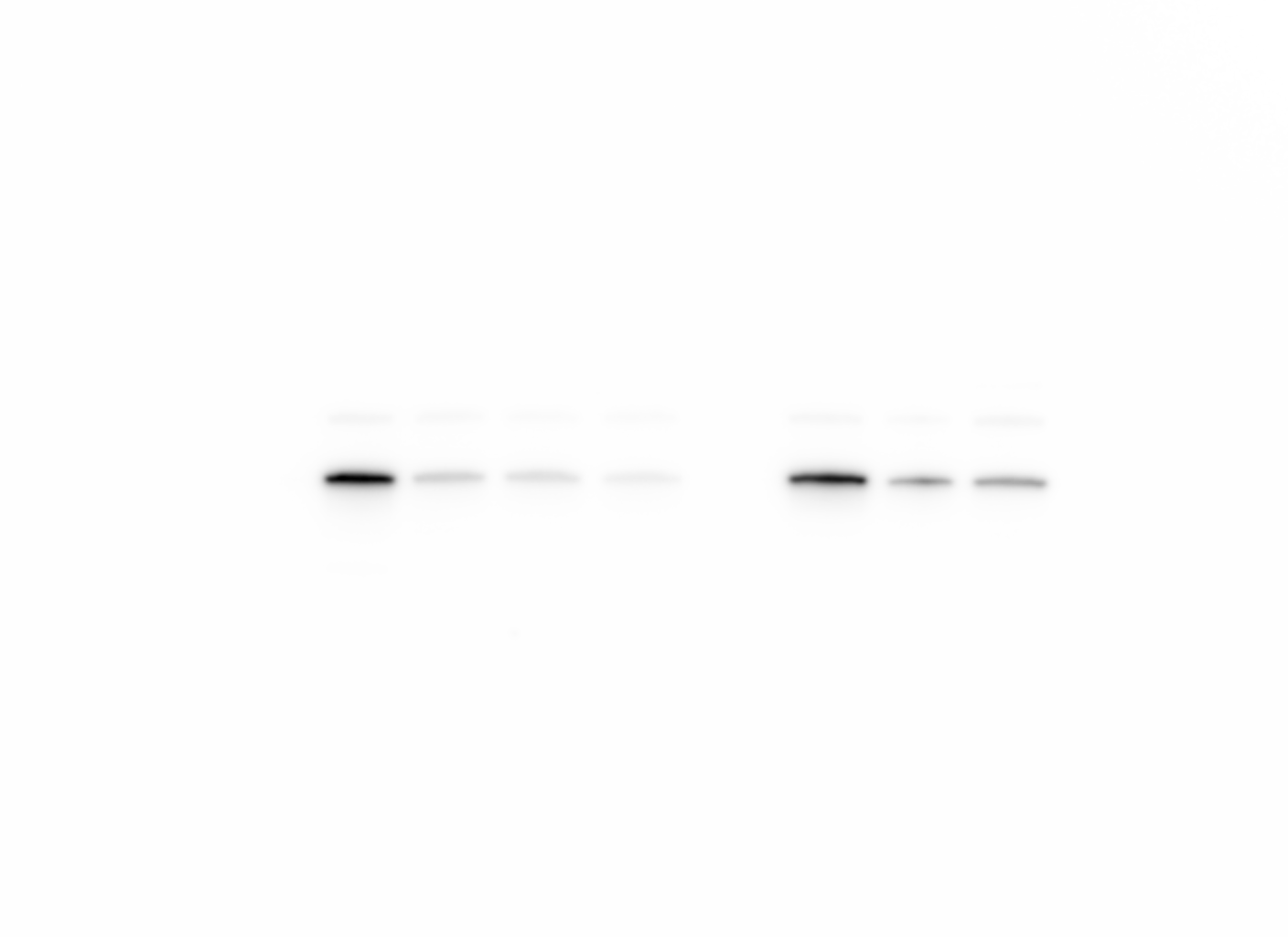

Supplement: Figure 3—figure supplement 1—source data 1. [file elife-105821-fig3-figsupp1-data1.zip › Figure 3-figure supplement 1-source data 1/Original files for western blot analysis displayed in Figure 3-figure supplement 1L/Vps26A 2 2022.05.31_16.25.40_Ch/Vps26A 2 2022.05.31_16.25.40_Ch.tif]

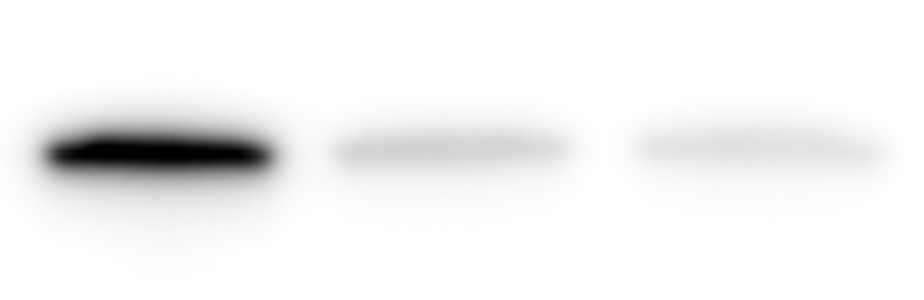

Supplement: Figure 3—figure supplement 1—source data 1. [file elife-105821-fig3-figsupp1-data1.zip › Figure 3-figure supplement 1-source data 1/Original files for western blot analysis displayed in Figure 3-figure supplement 1L/Vps26A 2 2022.05.31_16.25.40_Ch/Vps26A 2 2022.05.31_16.25.40_Ch-1.tif]

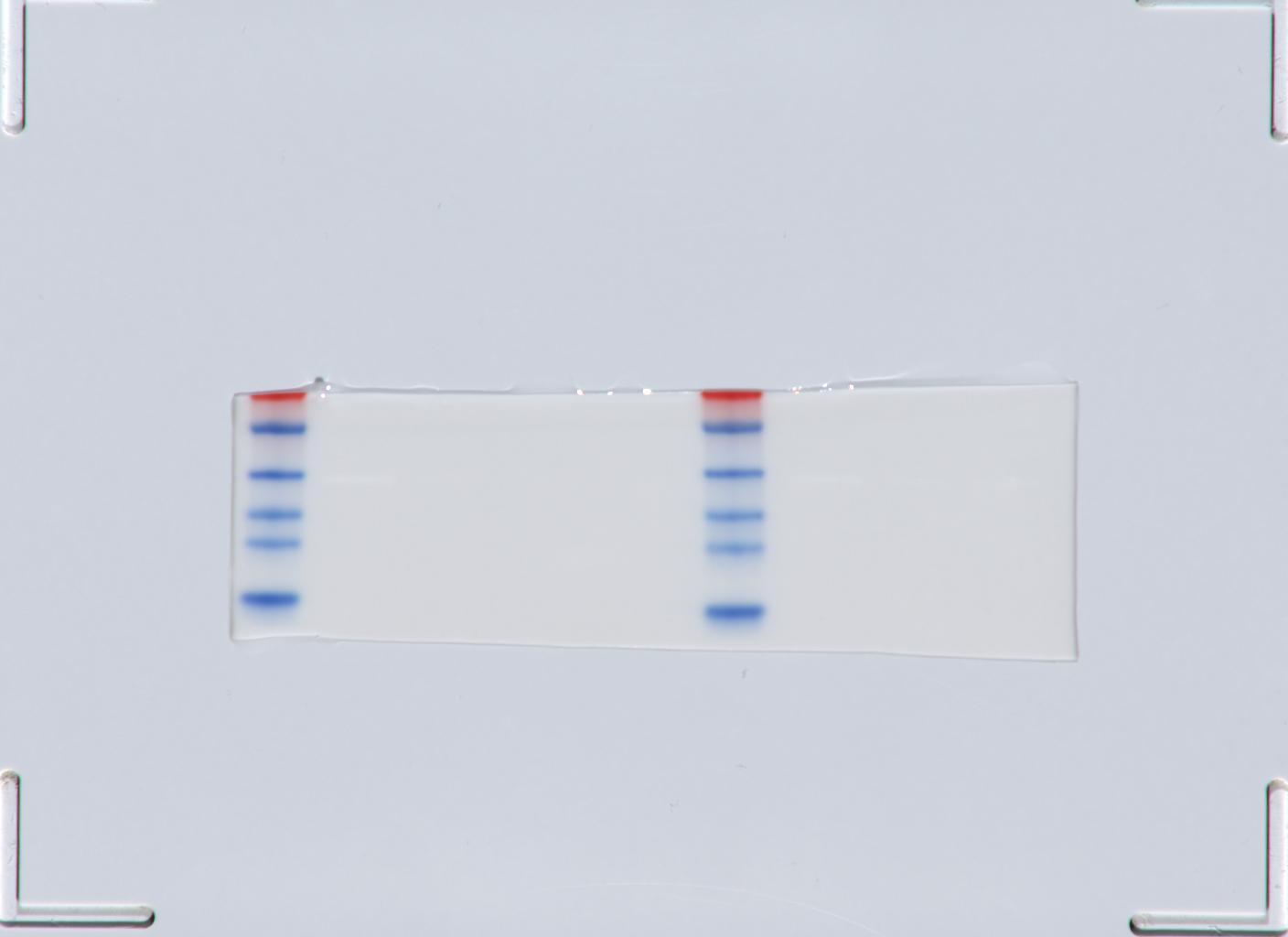

Supplement: Figure 3—figure supplement 1—source data 1. [file elife-105821-fig3-figsupp1-data1.zip › Figure 3-figure supplement 1-source data 1/Original files for western blot analysis displayed in Figure 3-figure supplement 1L/Vps26A 2 2022.05.31_16.25.40_Ch/Vps26A 2 2022.05.31_16.25.40_Ch-Marker.jpg]

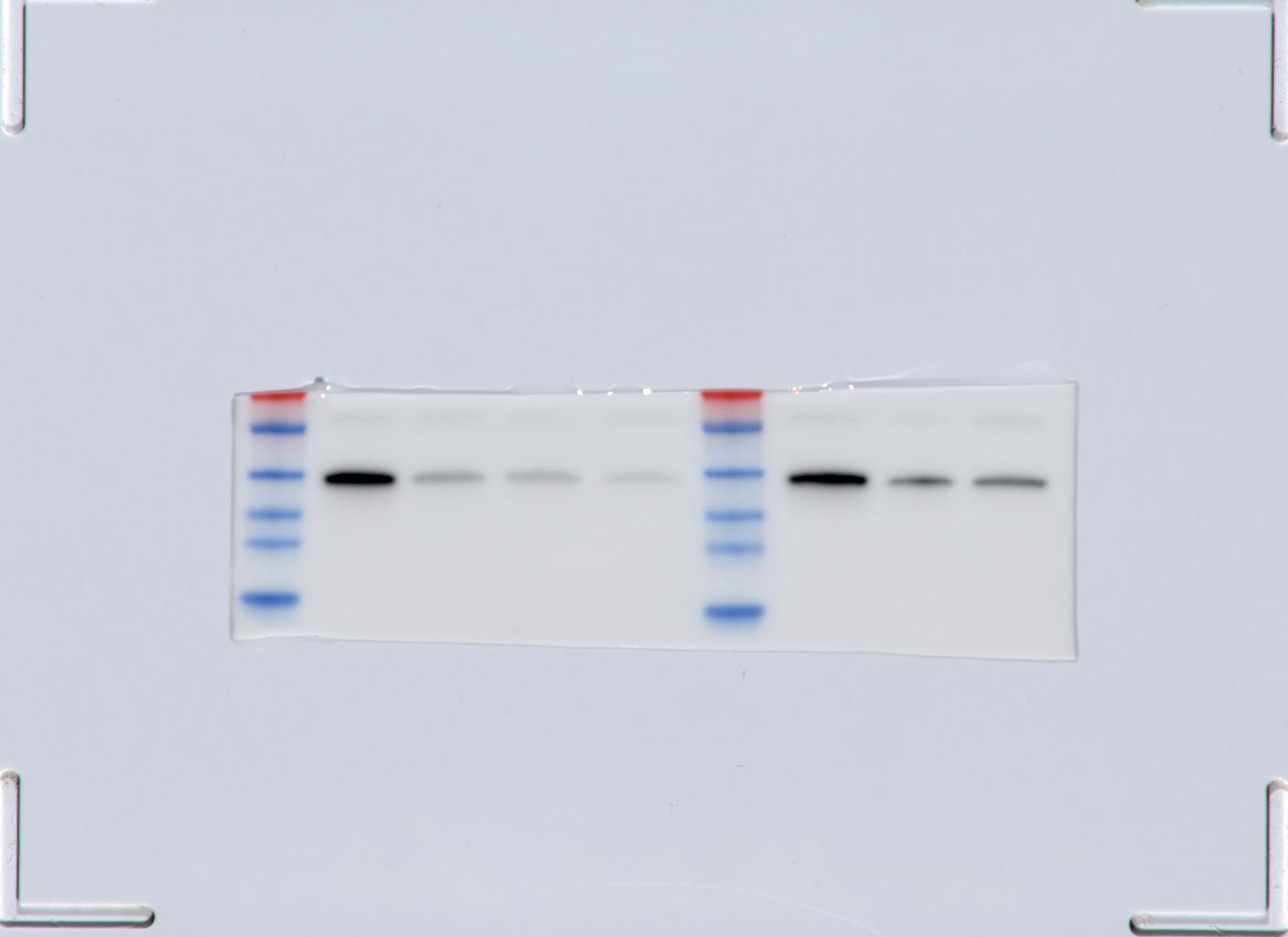

Supplement: Figure 3—figure supplement 1—source data 1. [file elife-105821-fig3-figsupp1-data1.zip › Figure 3-figure supplement 1-source data 1/Original files for western blot analysis displayed in Figure 3-figure supplement 1L/Vps26A 2 2022.05.31_16.25.40_Ch/Vps26A 2 2022.05.31_16.25.40_Ch+Marker.jpg]

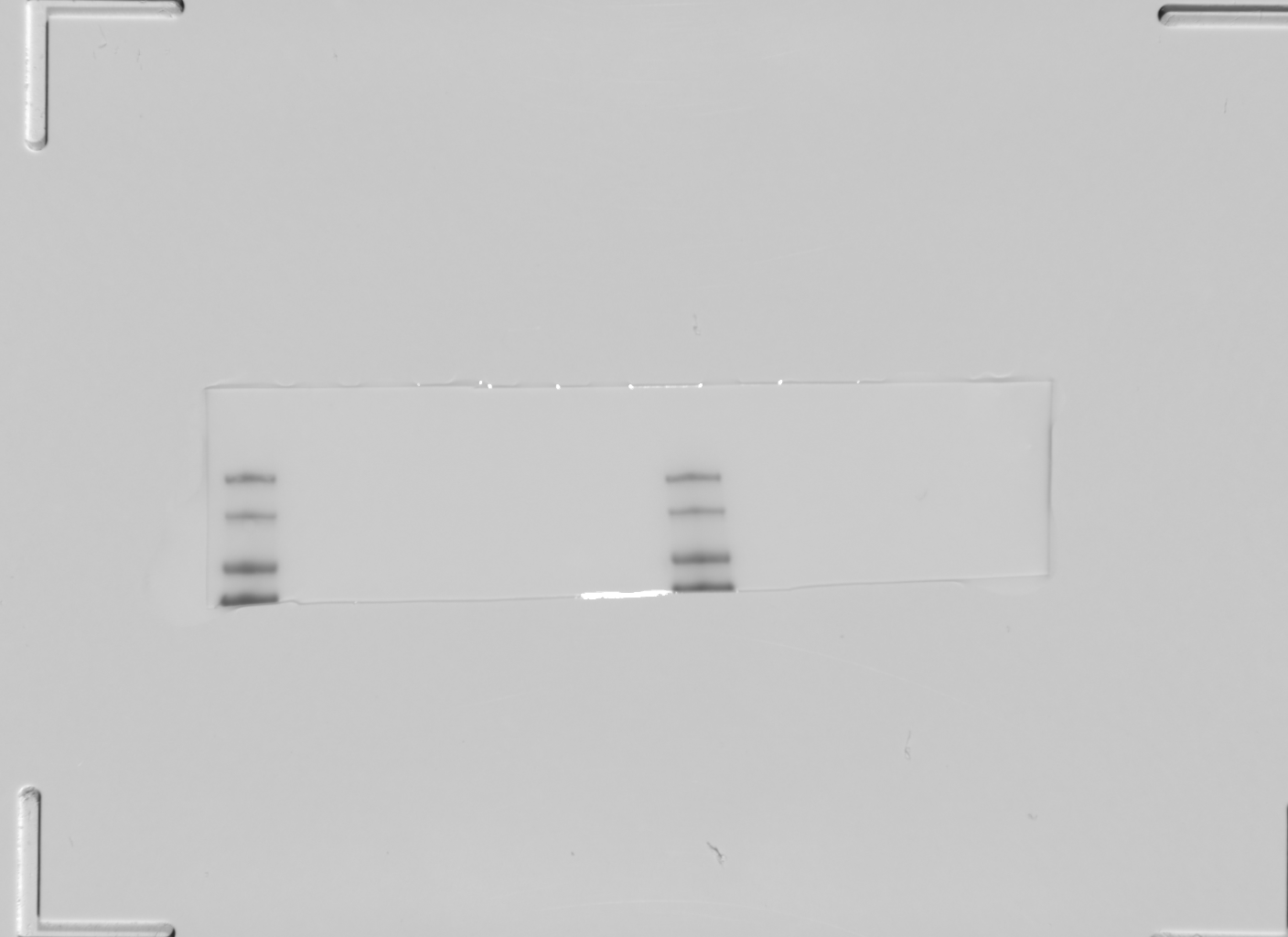

Supplement: Figure 3—figure supplement 1—source data 1. [file elife-105821-fig3-figsupp1-data1.zip › Figure 3-figure supplement 1-source data 1/Original files for western blot analysis displayed in Figure 3-figure supplement 1L/Vps35 2 2022.05.31_16.41.19_Ch/Vps35 2 2022.05.31_16.41.19_Ch-Marker.tif]

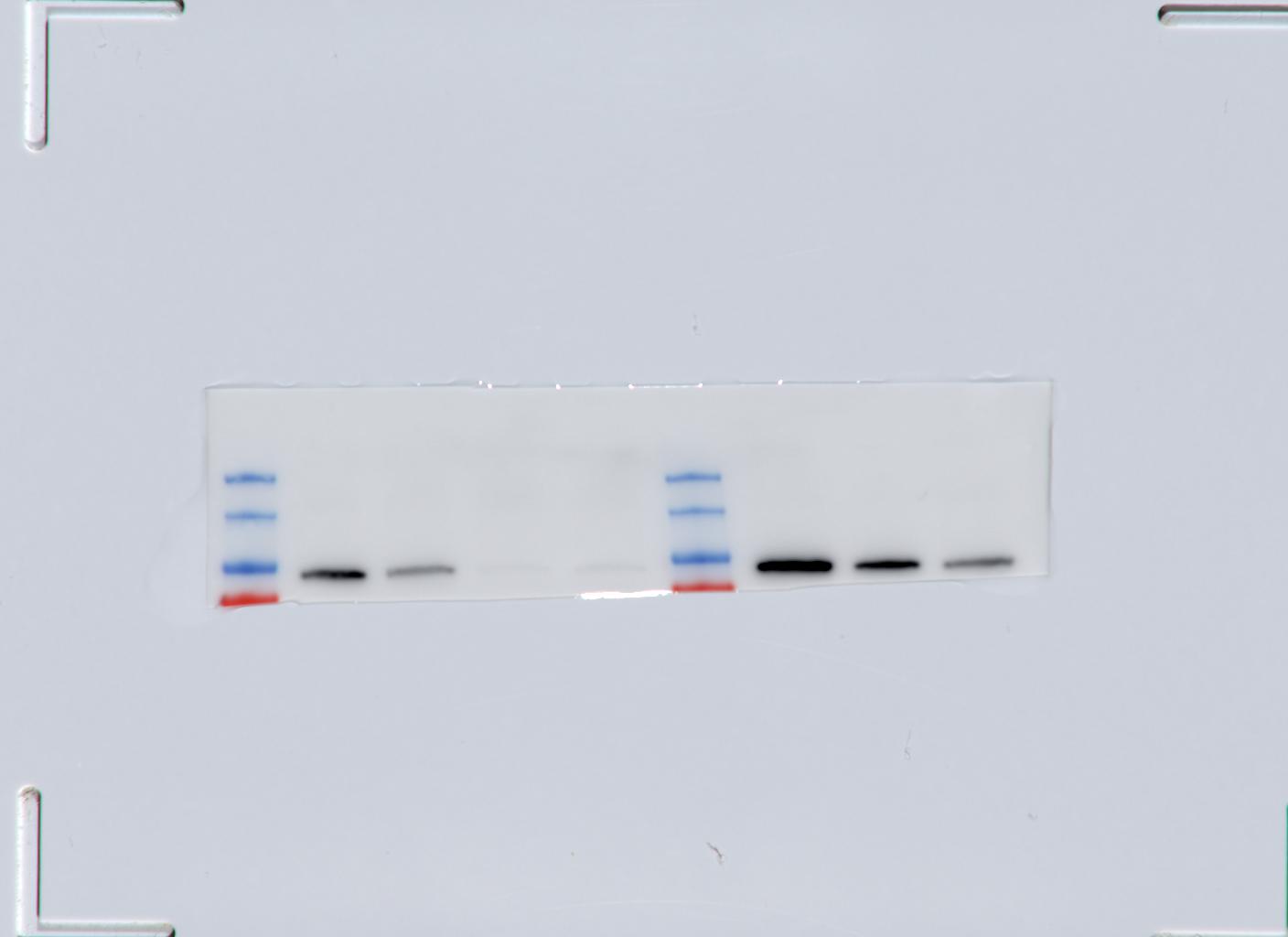

Supplement: Figure 3—figure supplement 1—source data 1. [file elife-105821-fig3-figsupp1-data1.zip › Figure 3-figure supplement 1-source data 1/Original files for western blot analysis displayed in Figure 3-figure supplement 1L/Vps35 2 2022.05.31_16.41.19_Ch/Vps35 2 2022.05.31_16.41.19_Ch+Marker.jpg]

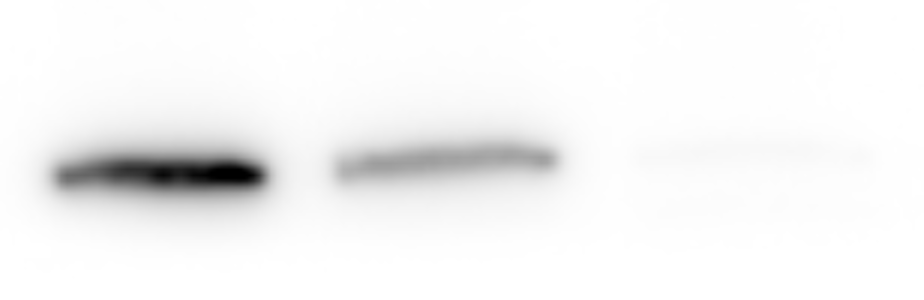

Supplement: Figure 3—figure supplement 1—source data 1. [file elife-105821-fig3-figsupp1-data1.zip › Figure 3-figure supplement 1-source data 1/Original files for western blot analysis displayed in Figure 3-figure supplement 1L/Vps35 2 2022.05.31_16.41.19_Ch/Vps35 2 2022.05.31_16.41.19_Ch-1.tif]

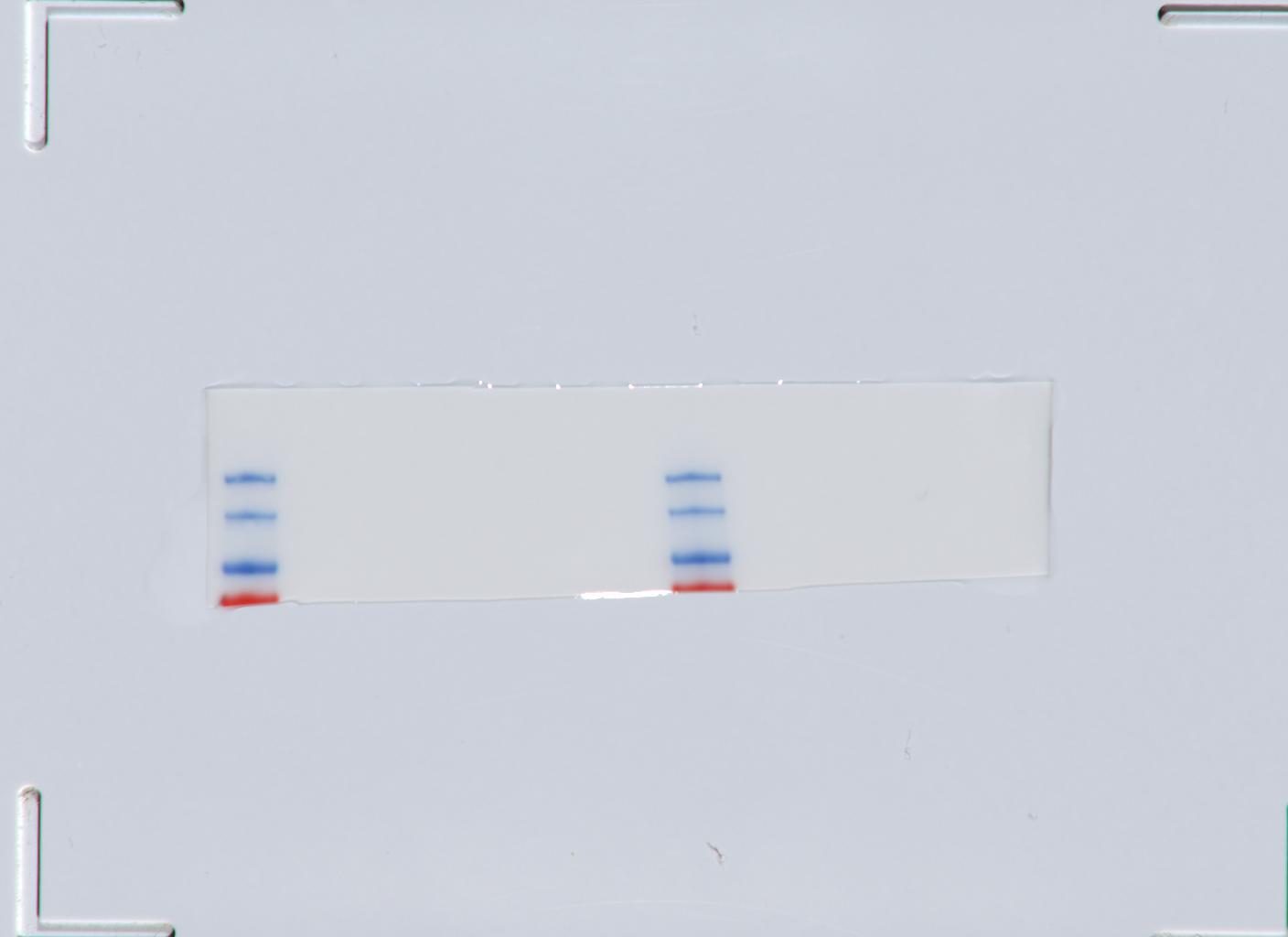

Supplement: Figure 3—figure supplement 1—source data 1. [file elife-105821-fig3-figsupp1-data1.zip › Figure 3-figure supplement 1-source data 1/Original files for western blot analysis displayed in Figure 3-figure supplement 1L/Vps35 2 2022.05.31_16.41.19_Ch/Vps35 2 2022.05.31_16.41.19_Ch-Marker.jpg]

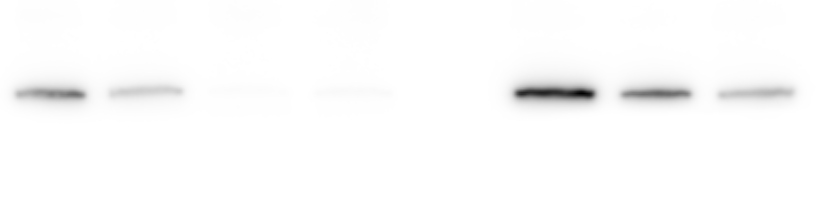

Supplement: Figure 3—figure supplement 1—source data 1. [file elife-105821-fig3-figsupp1-data1.zip › Figure 3-figure supplement 1-source data 1/Original files for western blot analysis displayed in Figure 3-figure supplement 1L/Vps35 2 2022.05.31_16.41.19_Ch/Vps35 2 2022.05.31_16.41.19_Ch.png]

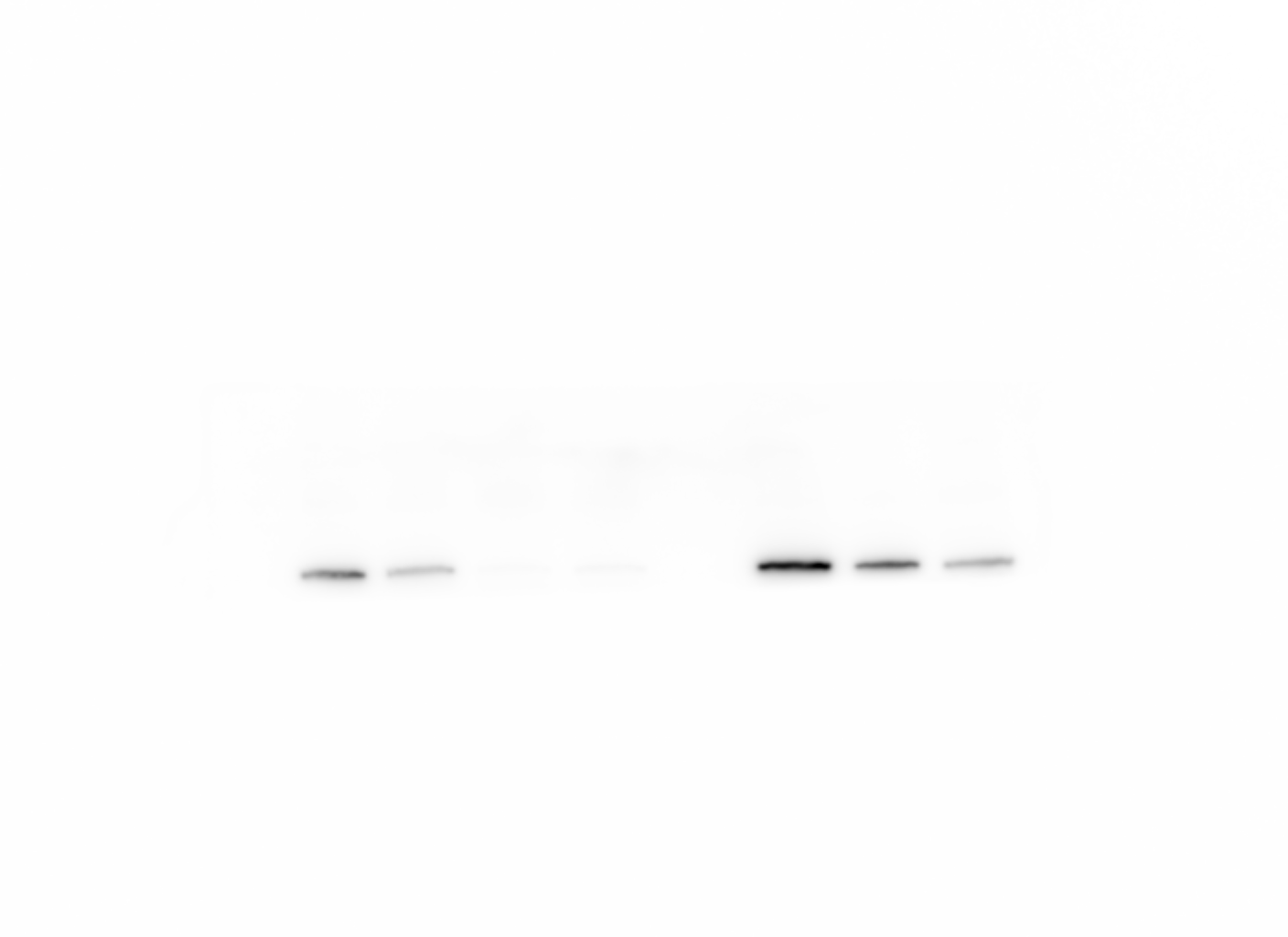

Supplement: Figure 3—figure supplement 1—source data 1. [file elife-105821-fig3-figsupp1-data1.zip › Figure 3-figure supplement 1-source data 1/Original files for western blot analysis displayed in Figure 3-figure supplement 1L/Vps35 2 2022.05.31_16.41.19_Ch/Vps35 2 2022.05.31_16.41.19_Ch.tif]

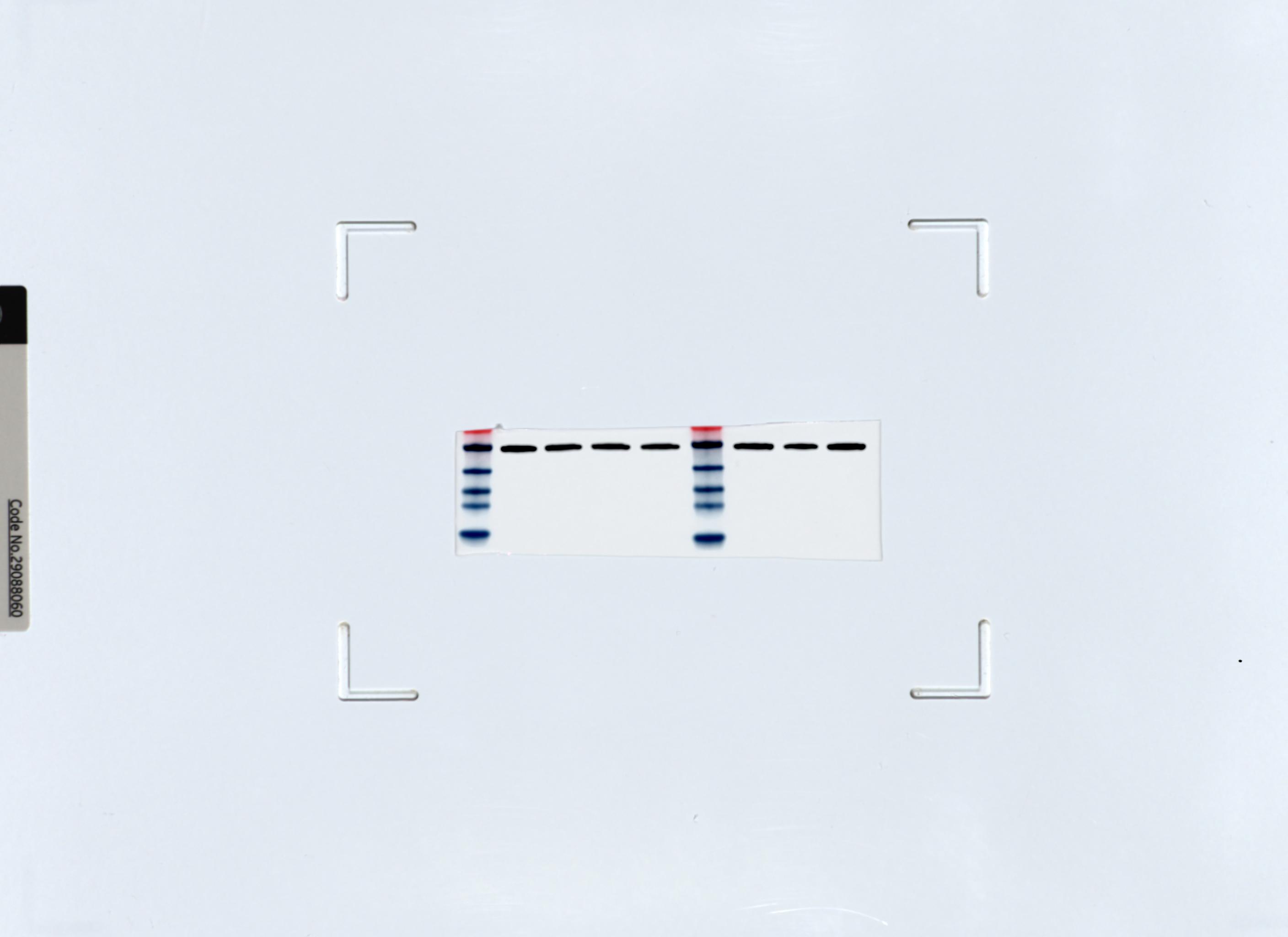

Supplement: Figure 3—figure supplement 1—source data 1. [file elife-105821-fig3-figsupp1-data1.zip › Figure 3-figure supplement 1-source data 1/Original files for western blot analysis displayed in Figure 3-figure supplement 1L/tubulin 2 2022.05.31_16.22.05_Fl-Red/tubulin 2 2022.05.31_16.22.05_Fl-Red+Marker.jpg]

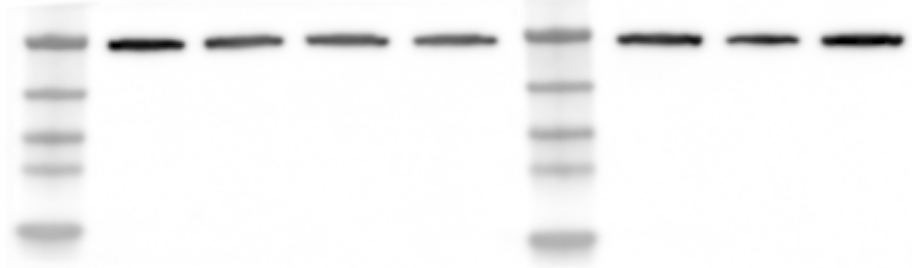

Supplement: Figure 3—figure supplement 1—source data 1. [file elife-105821-fig3-figsupp1-data1.zip › Figure 3-figure supplement 1-source data 1/Original files for western blot analysis displayed in Figure 3-figure supplement 1L/tubulin 2 2022.05.31_16.22.05_Fl-Red/tubulin 2 2022.05.31_16.22.05_Fl-Red.png]

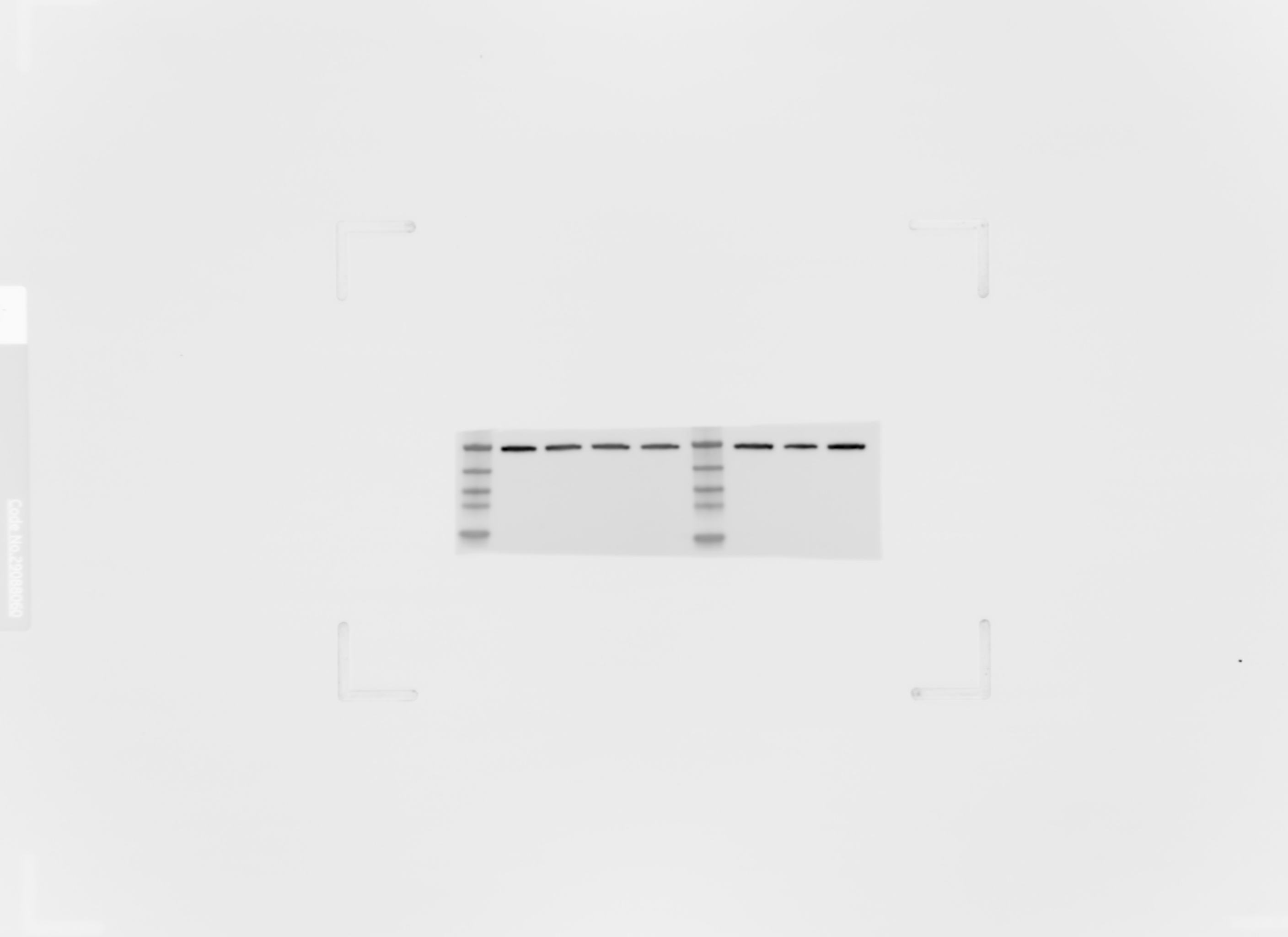

Supplement: Figure 3—figure supplement 1—source data 1. [file elife-105821-fig3-figsupp1-data1.zip › Figure 3-figure supplement 1-source data 1/Original files for western blot analysis displayed in Figure 3-figure supplement 1L/tubulin 2 2022.05.31_16.22.05_Fl-Red/tubulin 2 2022.05.31_16.22.05_Fl-Red.tif]

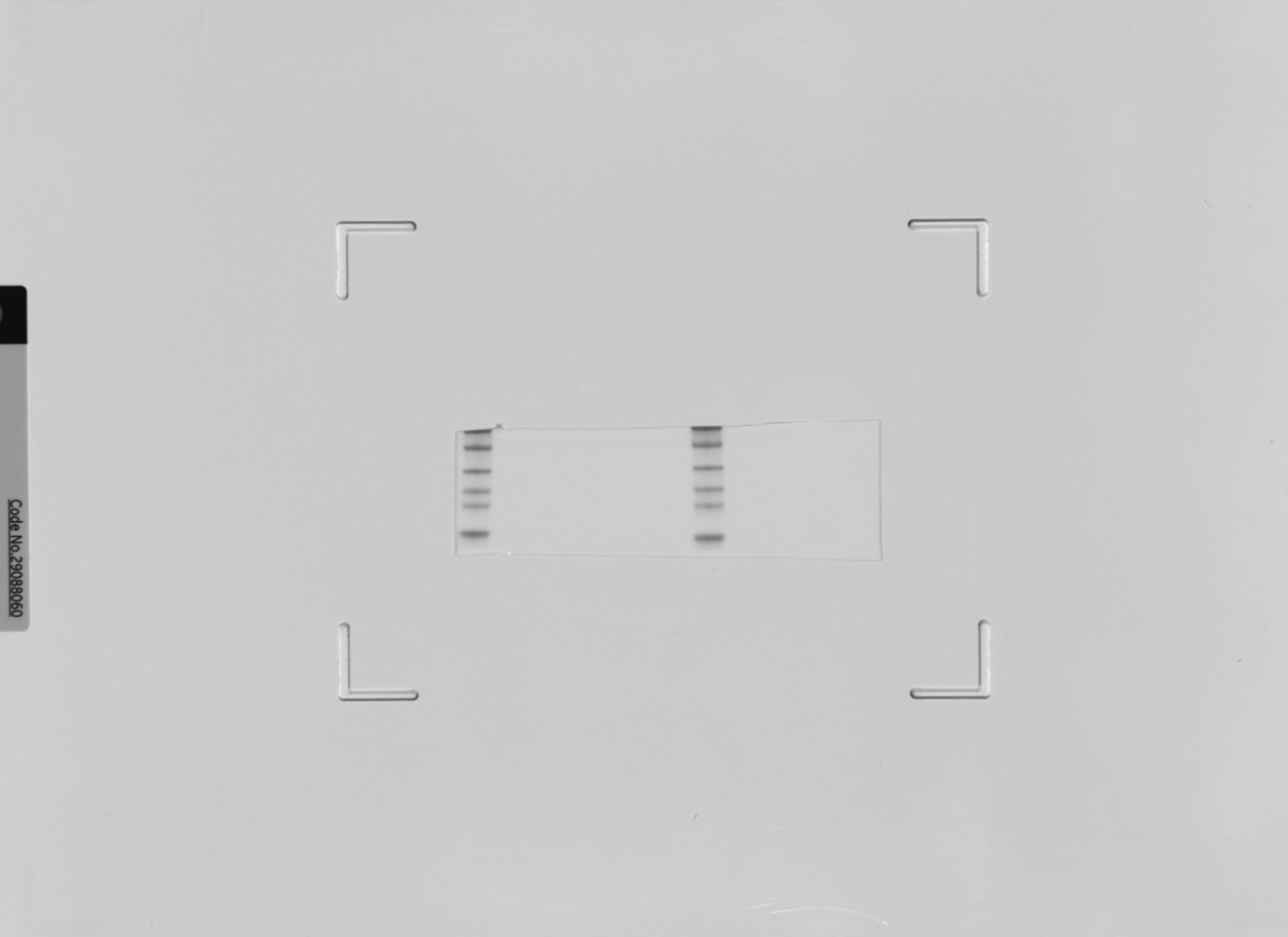

Supplement: Figure 3—figure supplement 1—source data 1. [file elife-105821-fig3-figsupp1-data1.zip › Figure 3-figure supplement 1-source data 1/Original files for western blot analysis displayed in Figure 3-figure supplement 1L/tubulin 2 2022.05.31_16.22.05_Fl-Red/tubulin 2 2022.05.31_16.22.05_Fl-Red-Marker.tif]

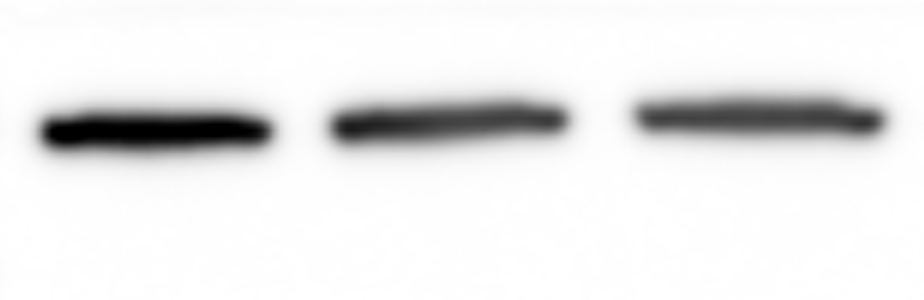

Supplement: Figure 3—figure supplement 1—source data 1. [file elife-105821-fig3-figsupp1-data1.zip › Figure 3-figure supplement 1-source data 1/Original files for western blot analysis displayed in Figure 3-figure supplement 1L/tubulin 2 2022.05.31_16.22.05_Fl-Red/tubulin 2 2022.05.31_16.22.05_Fl-Red-1.tif]

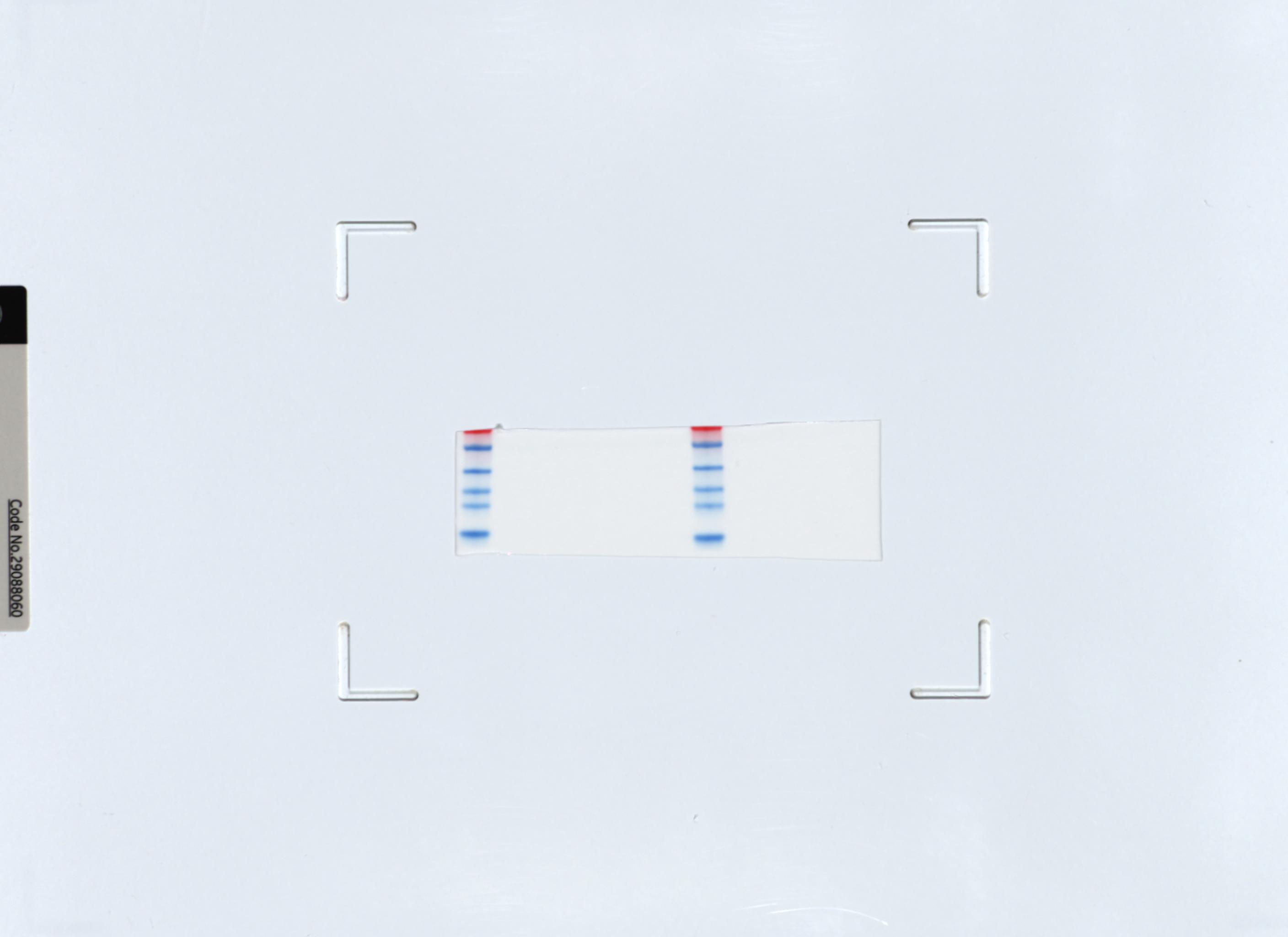

Supplement: Figure 3—figure supplement 1—source data 1. [file elife-105821-fig3-figsupp1-data1.zip › Figure 3-figure supplement 1-source data 1/Original files for western blot analysis displayed in Figure 3-figure supplement 1L/tubulin 2 2022.05.31_16.22.05_Fl-Red/tubulin 2 2022.05.31_16.22.05_Fl-Red-Marker.jpg]

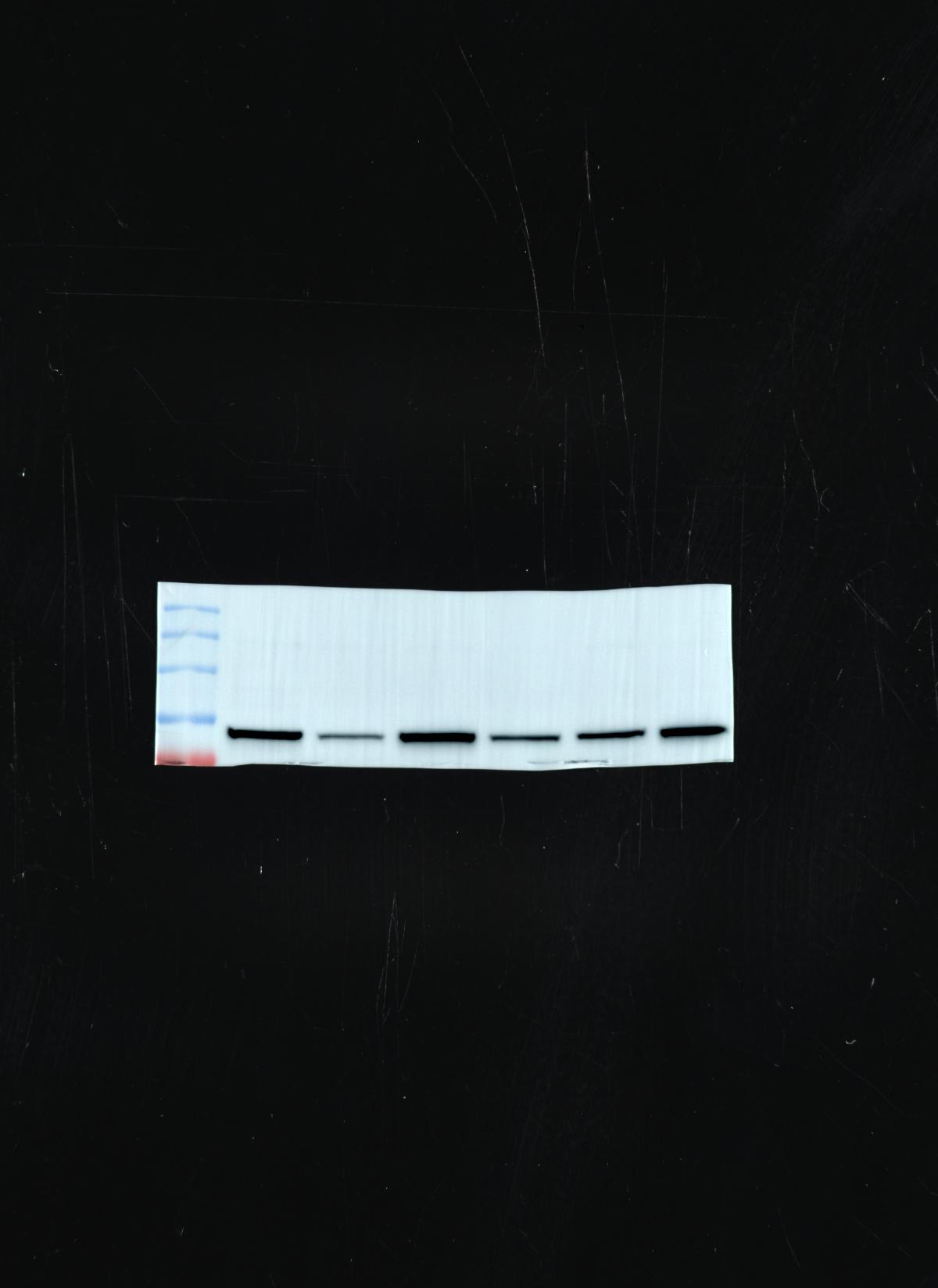

Supplement: Figure 5—figure supplement 1—source data 1. [file elife-105821-fig5-figsupp1-data1.zip › Figure 5-figure supplement 2-source data 1/Original files for western blot analysis displayed in Figure 5-figure supplement 2C/EA3_GFP 20240830_120110_Ch/EA3_GFP 20240830_120110_Ch_Chemi+Marker.jpg]

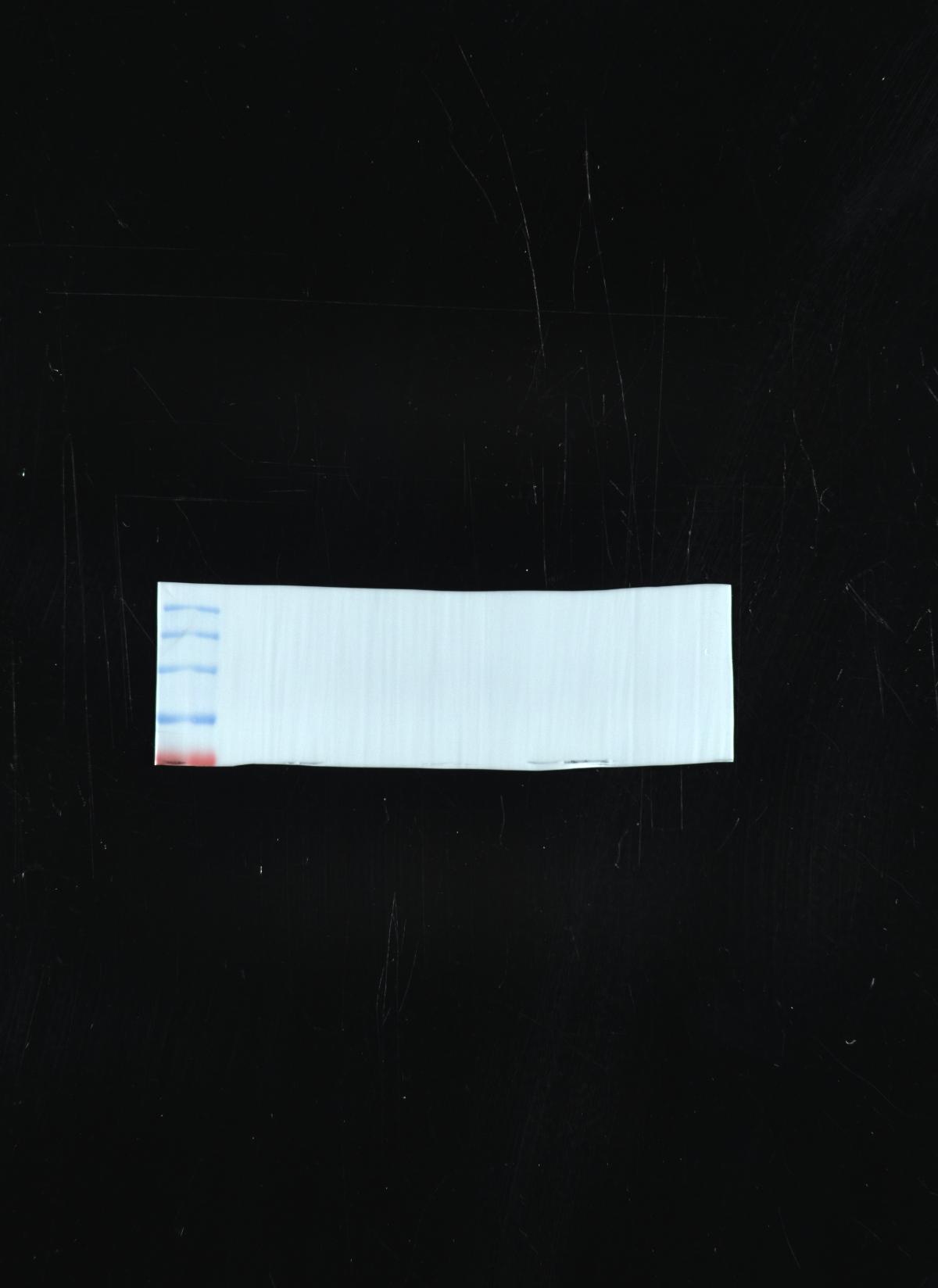

Supplement: Figure 5—figure supplement 1—source data 1. [file elife-105821-fig5-figsupp1-data1.zip › Figure 5-figure supplement 2-source data 1/Original files for western blot analysis displayed in Figure 5-figure supplement 2C/EA3_GFP 20240830_120110_Ch/EA3_GFP 20240830_120110_Ch-Marker.jpg]

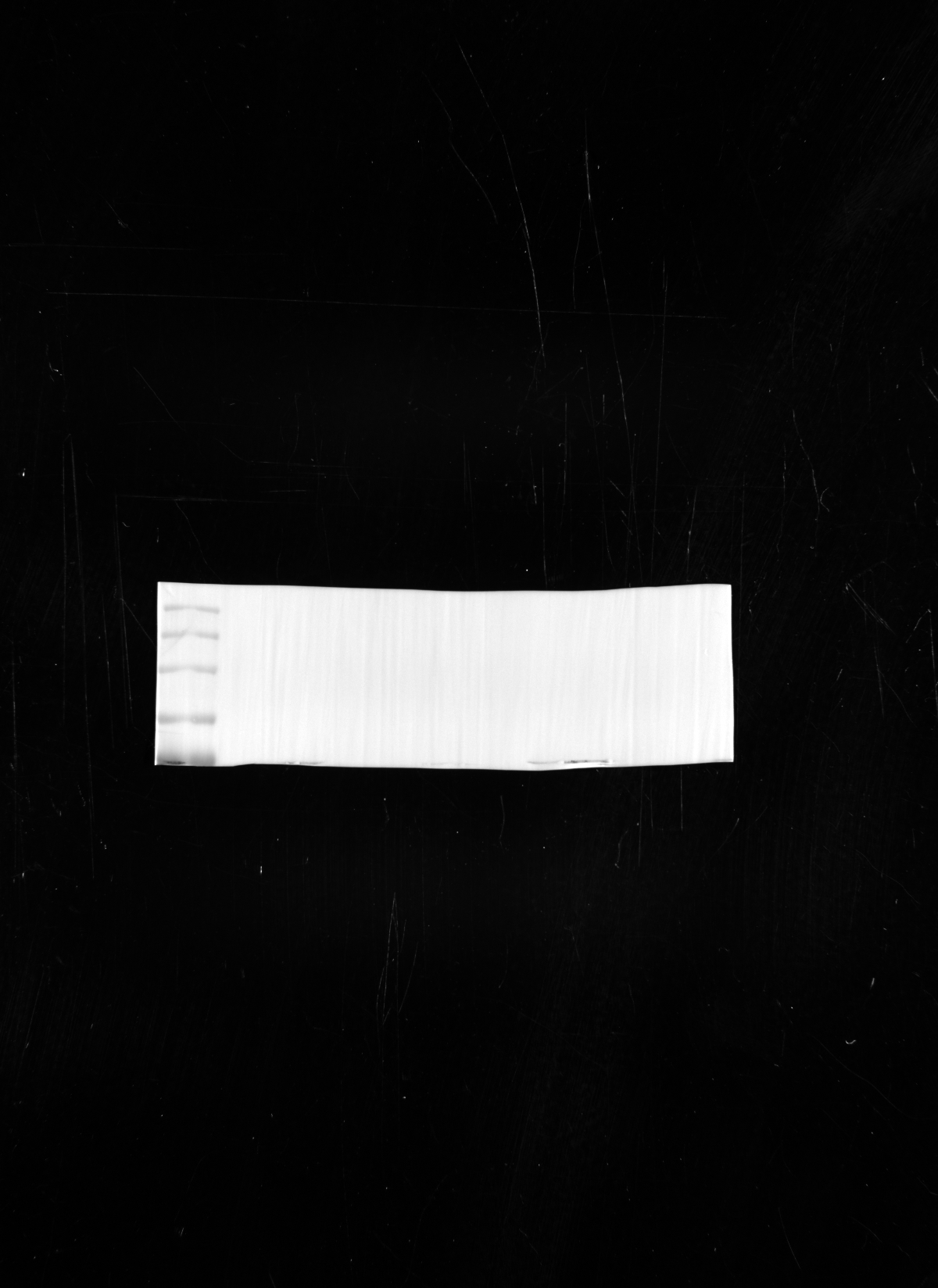

Supplement: Figure 5—figure supplement 1—source data 1. [file elife-105821-fig5-figsupp1-data1.zip › Figure 5-figure supplement 2-source data 1/Original files for western blot analysis displayed in Figure 5-figure supplement 2C/EA3_GFP 20240830_120110_Ch/EA3_GFP 20240830_120110_Ch-Marker.tif]

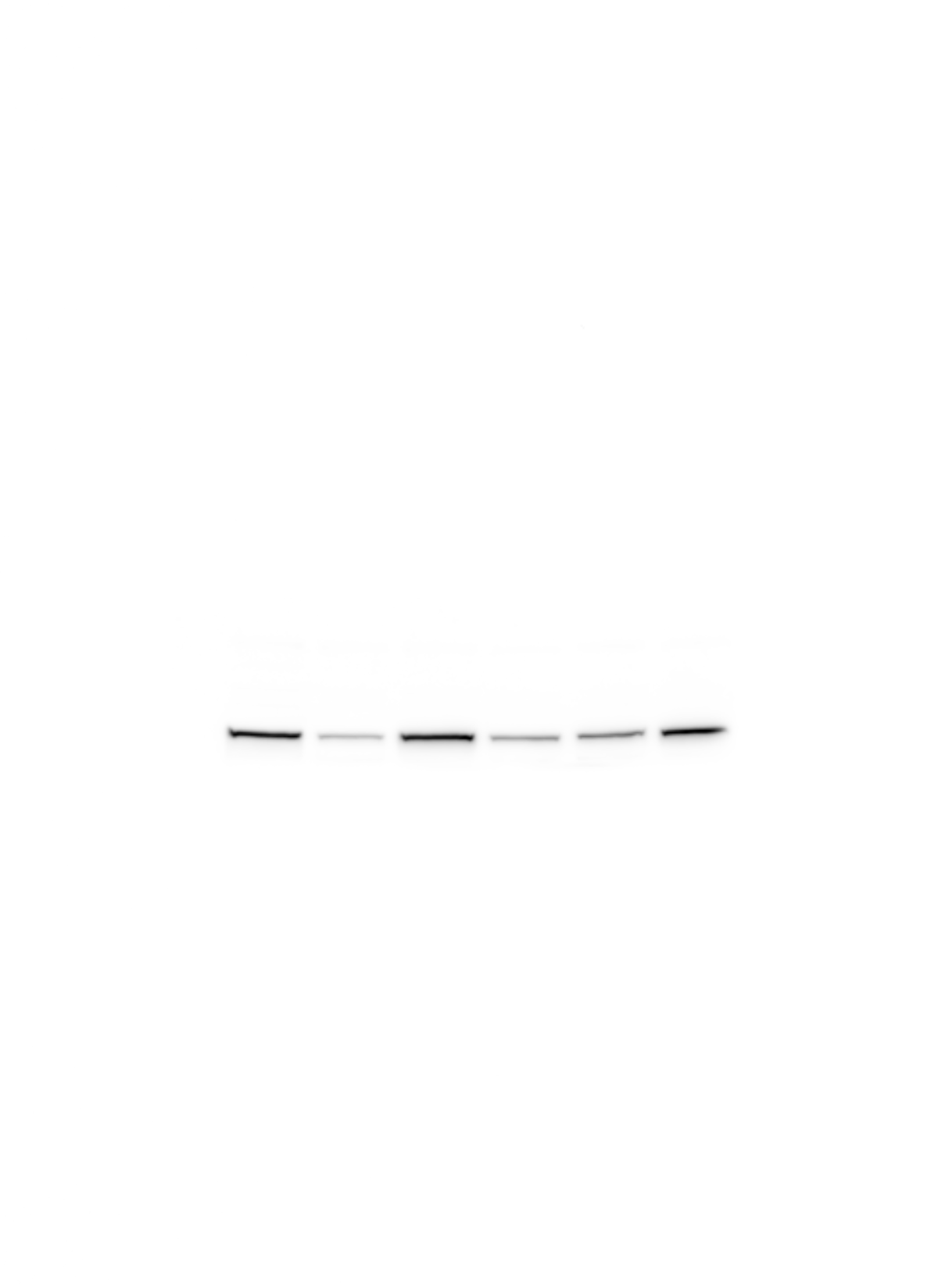

Supplement: Figure 5—figure supplement 1—source data 1. [file elife-105821-fig5-figsupp1-data1.zip › Figure 5-figure supplement 2-source data 1/Original files for western blot analysis displayed in Figure 5-figure supplement 2C/EA3_GFP 20240830_120110_Ch/EA3_GFP 20240830_120110_Ch_Chemi.tif]

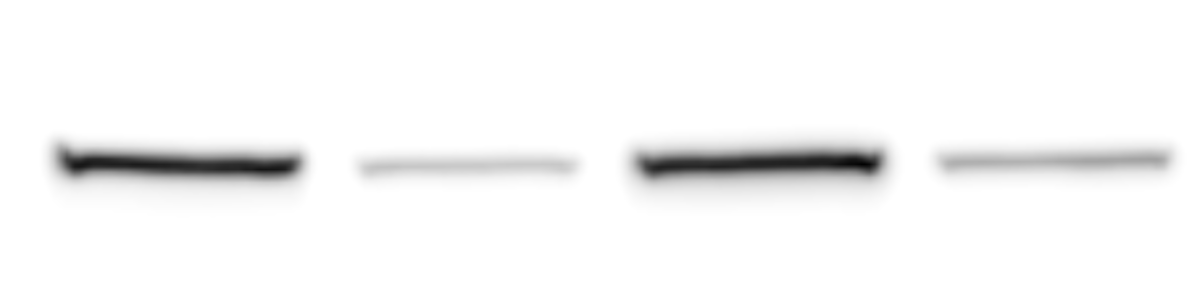

Supplement: Figure 5—figure supplement 1—source data 1. [file elife-105821-fig5-figsupp1-data1.zip › Figure 5-figure supplement 2-source data 1/Original files for western blot analysis displayed in Figure 5-figure supplement 2C/EA3_GFP 20240830_120110_Ch/EA3_GFP 20240830_120110_Ch_Chemi-1.tif]

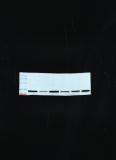

Supplement: Figure 5—figure supplement 1—source data 1. [file elife-105821-fig5-figsupp1-data1.zip › Figure 5-figure supplement 2-source data 1/Original files for western blot analysis displayed in Figure 5-figure supplement 2C/EA3_GFP 20240830_120110_Ch/EA3_GFP 20240830_120110_Ch_Thumb.jpg]

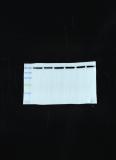

Supplement: Figure 5—figure supplement 1—source data 1. [file elife-105821-fig5-figsupp1-data1.zip › Figure 5-figure supplement 2-source data 1/Original files for western blot analysis displayed in Figure 5-figure supplement 2C/tubulin 20240830_120507_Ch/tubulin 20240830_120507_Ch_Thumb.jpg]

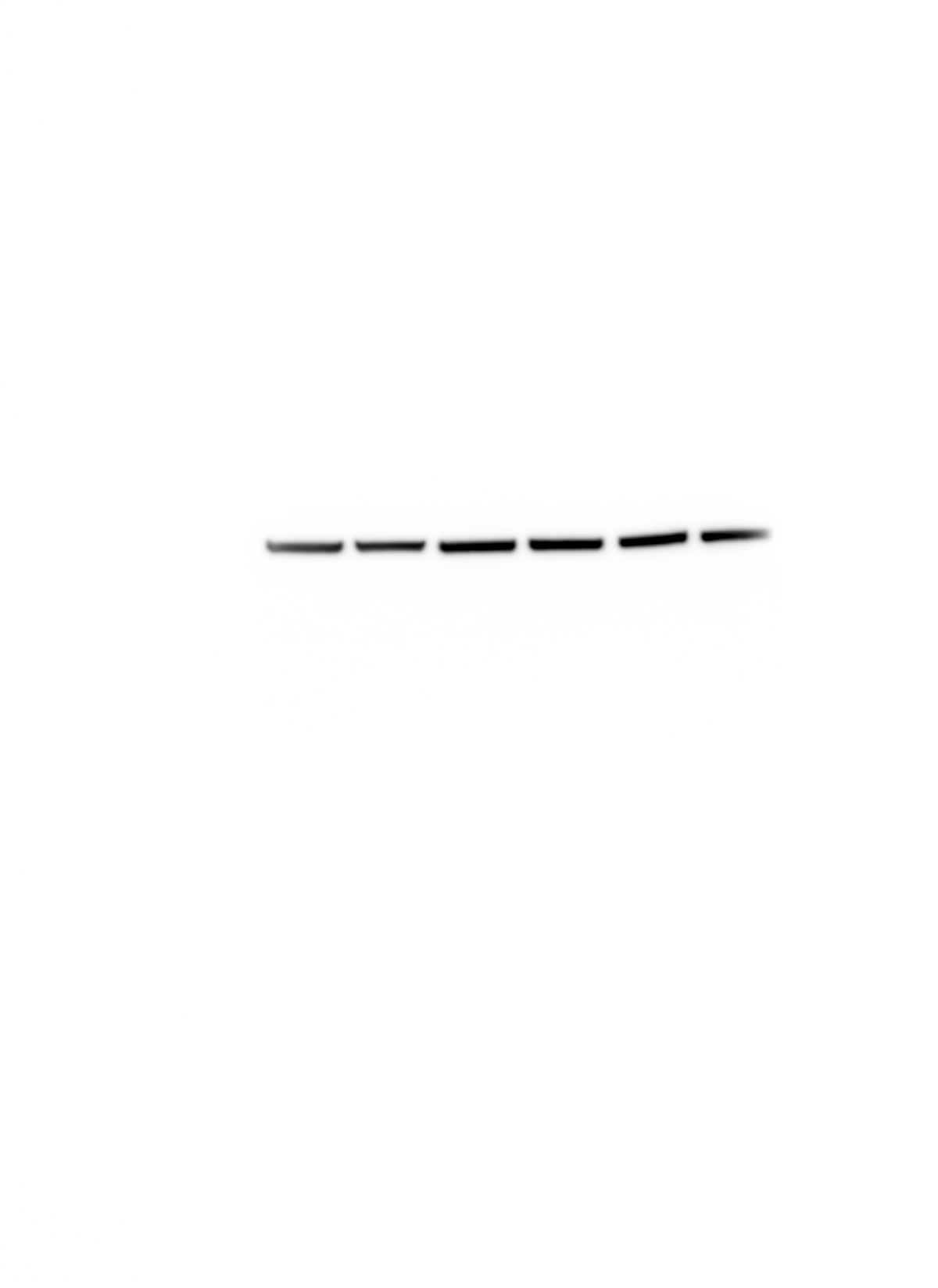

Supplement: Figure 5—figure supplement 1—source data 1. [file elife-105821-fig5-figsupp1-data1.zip › Figure 5-figure supplement 2-source data 1/Original files for western blot analysis displayed in Figure 5-figure supplement 2C/tubulin 20240830_120507_Ch/tubulin 20240830_120507_Ch_Chemi.tif]

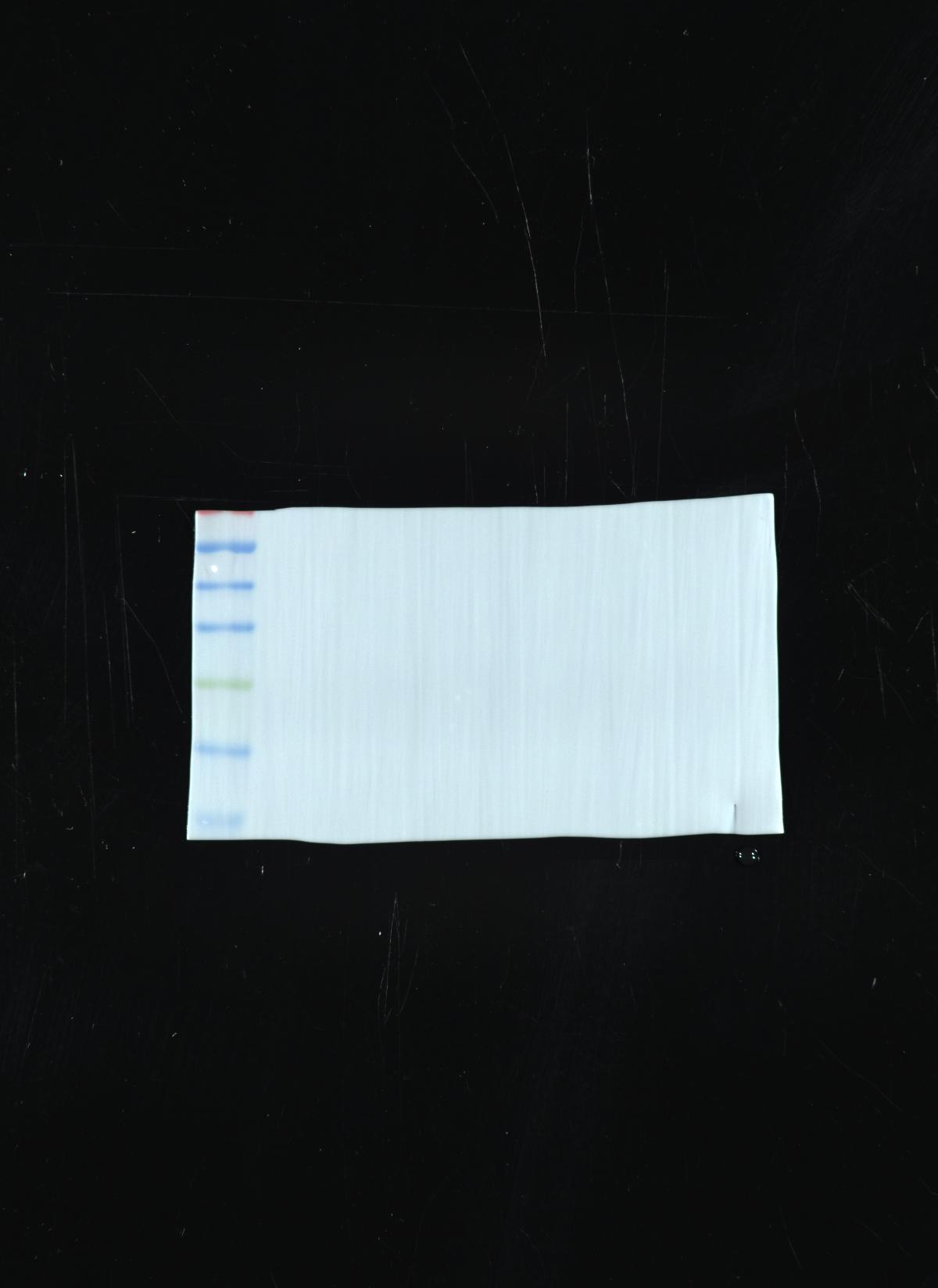

Supplement: Figure 5—figure supplement 1—source data 1. [file elife-105821-fig5-figsupp1-data1.zip › Figure 5-figure supplement 2-source data 1/Original files for western blot analysis displayed in Figure 5-figure supplement 2C/tubulin 20240830_120507_Ch/tubulin 20240830_120507_Ch-Marker.jpg]

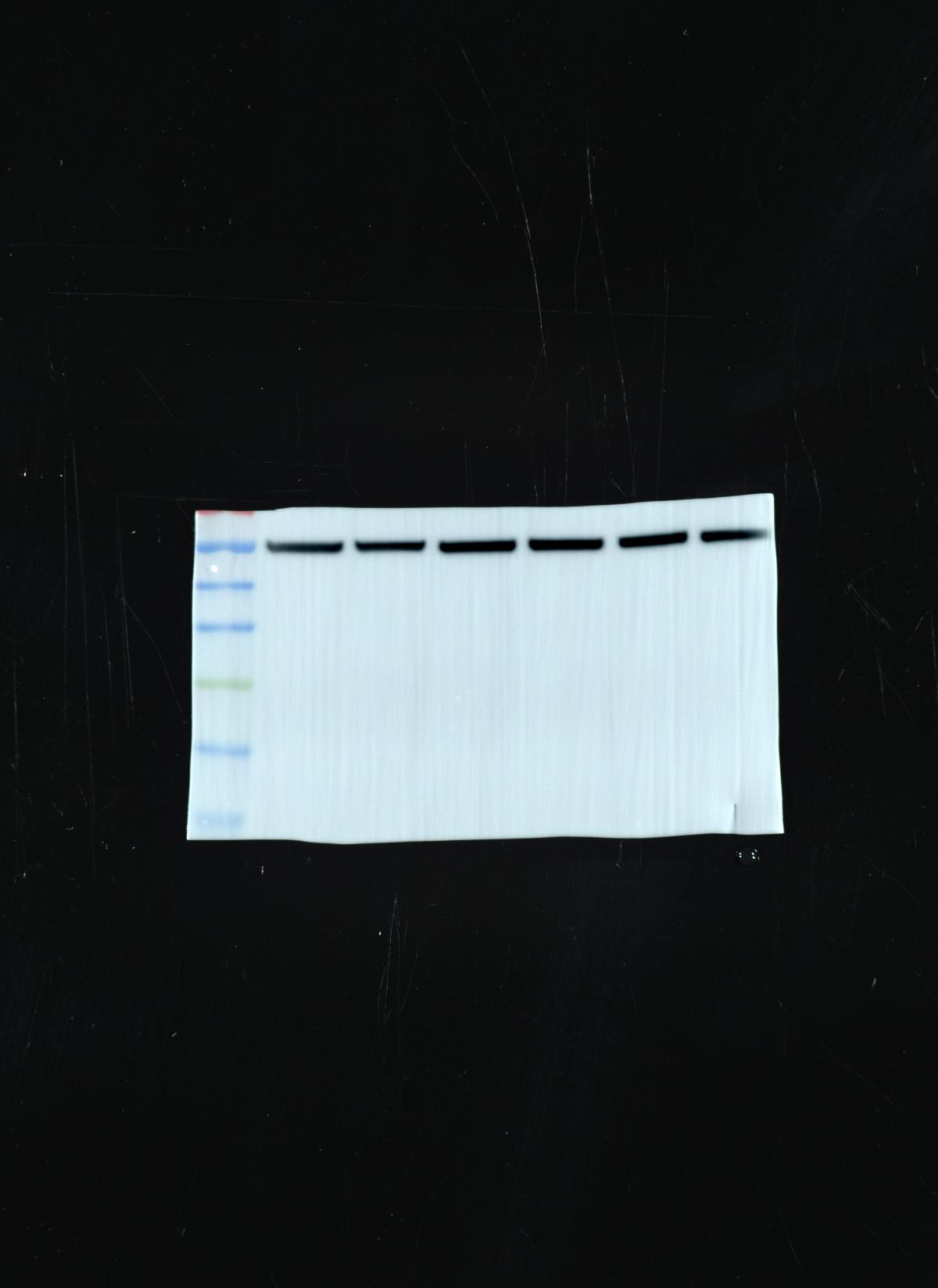

Supplement: Figure 5—figure supplement 1—source data 1. [file elife-105821-fig5-figsupp1-data1.zip › Figure 5-figure supplement 2-source data 1/Original files for western blot analysis displayed in Figure 5-figure supplement 2C/tubulin 20240830_120507_Ch/tubulin 20240830_120507_Ch_Chemi+Marker.jpg]

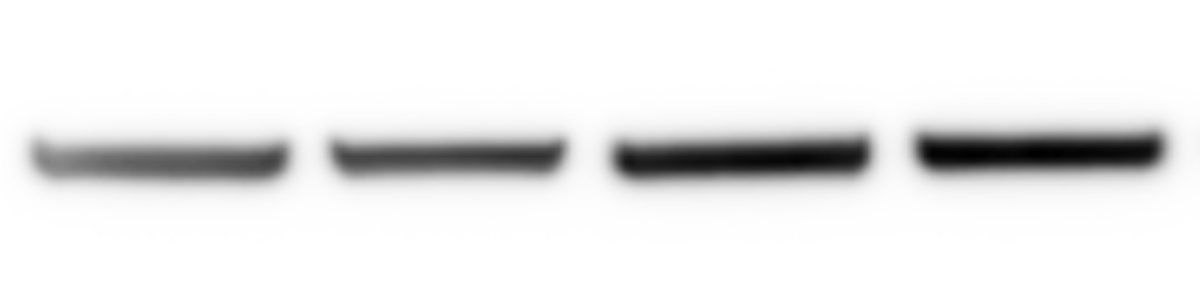

Supplement: Figure 5—figure supplement 1—source data 1. [file elife-105821-fig5-figsupp1-data1.zip › Figure 5-figure supplement 2-source data 1/Original files for western blot analysis displayed in Figure 5-figure supplement 2C/tubulin 20240830_120507_Ch/tubulin 20240830_120507_Ch_Chemi-1.tif]

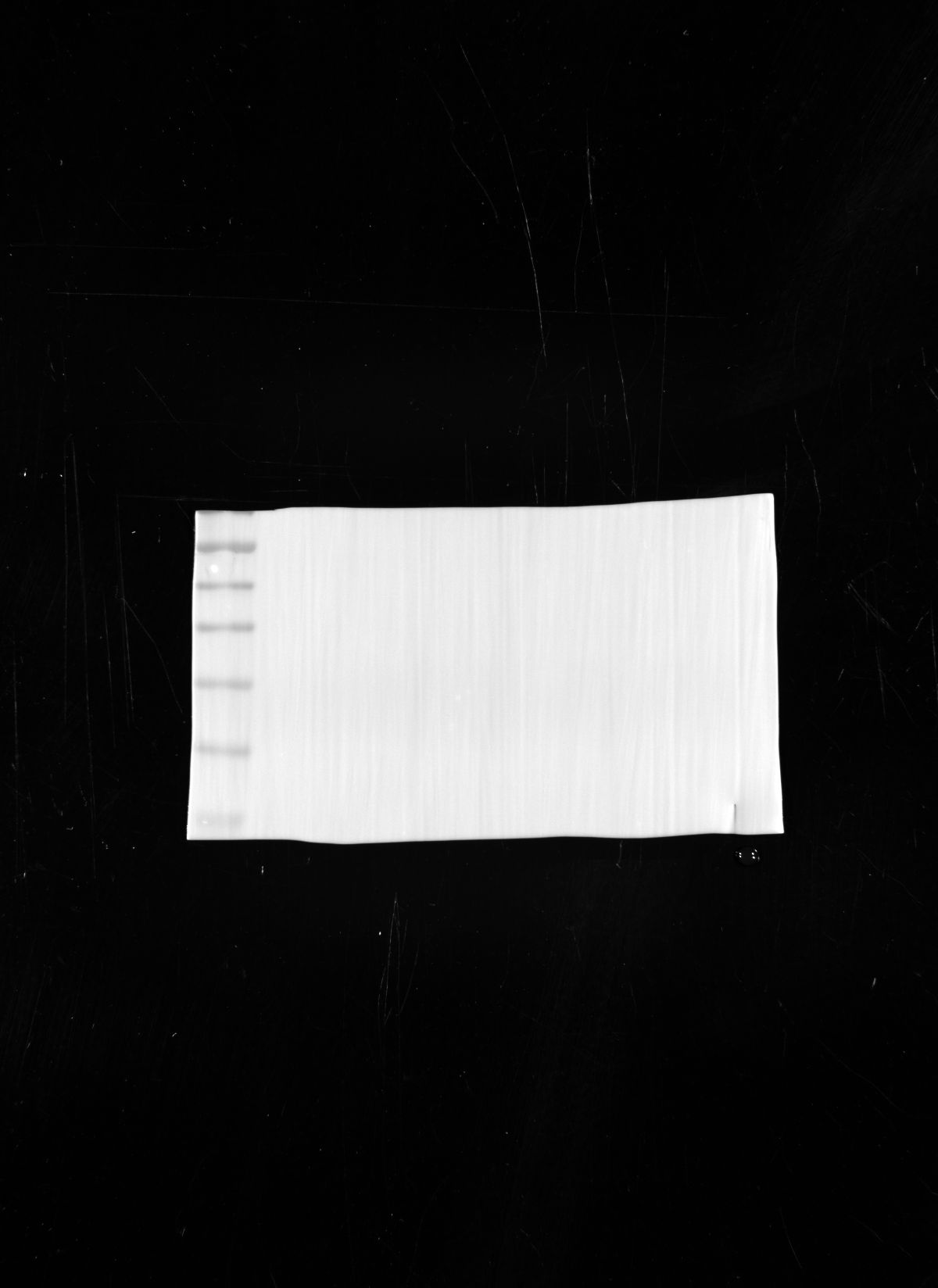

Supplement: Figure 5—figure supplement 1—source data 1. [file elife-105821-fig5-figsupp1-data1.zip › Figure 5-figure supplement 2-source data 1/Original files for western blot analysis displayed in Figure 5-figure supplement 2C/tubulin 20240830_120507_Ch/tubulin 20240830_120507_Ch-Marker.tif]

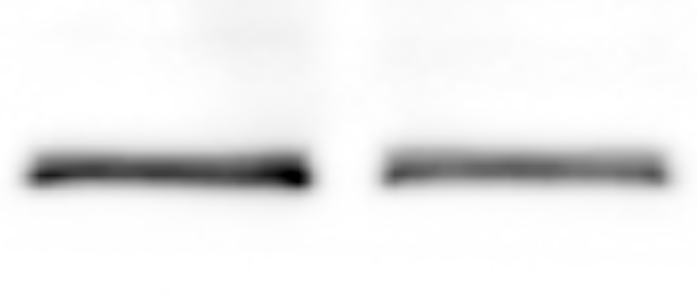

Supplement: Figure 5—figure supplement 1—source data 1. [file elife-105821-fig5-figsupp1-data1.zip › Figure 5-figure supplement 2-source data 1/Original files for western blot analysis displayed in Figure 5-figure supplement 2D/Vps35 20240606_120926_Ch/Vps35 20240606_120926_Ch_Chemi-1.tif]

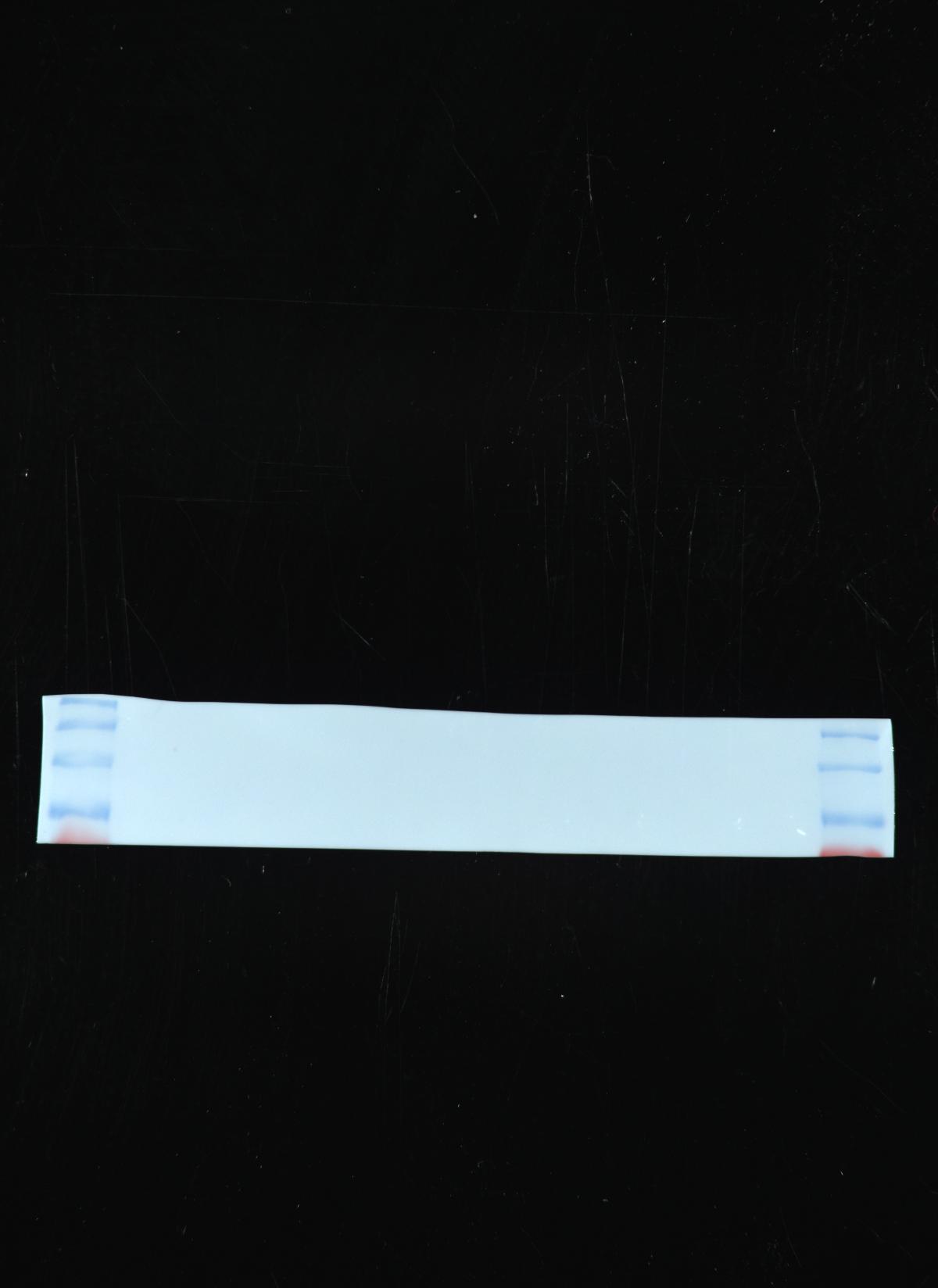

Supplement: Figure 5—figure supplement 1—source data 1. [file elife-105821-fig5-figsupp1-data1.zip › Figure 5-figure supplement 2-source data 1/Original files for western blot analysis displayed in Figure 5-figure supplement 2D/Vps35 20240606_120926_Ch/Vps35 20240606_120926_Ch-Marker.jpg]

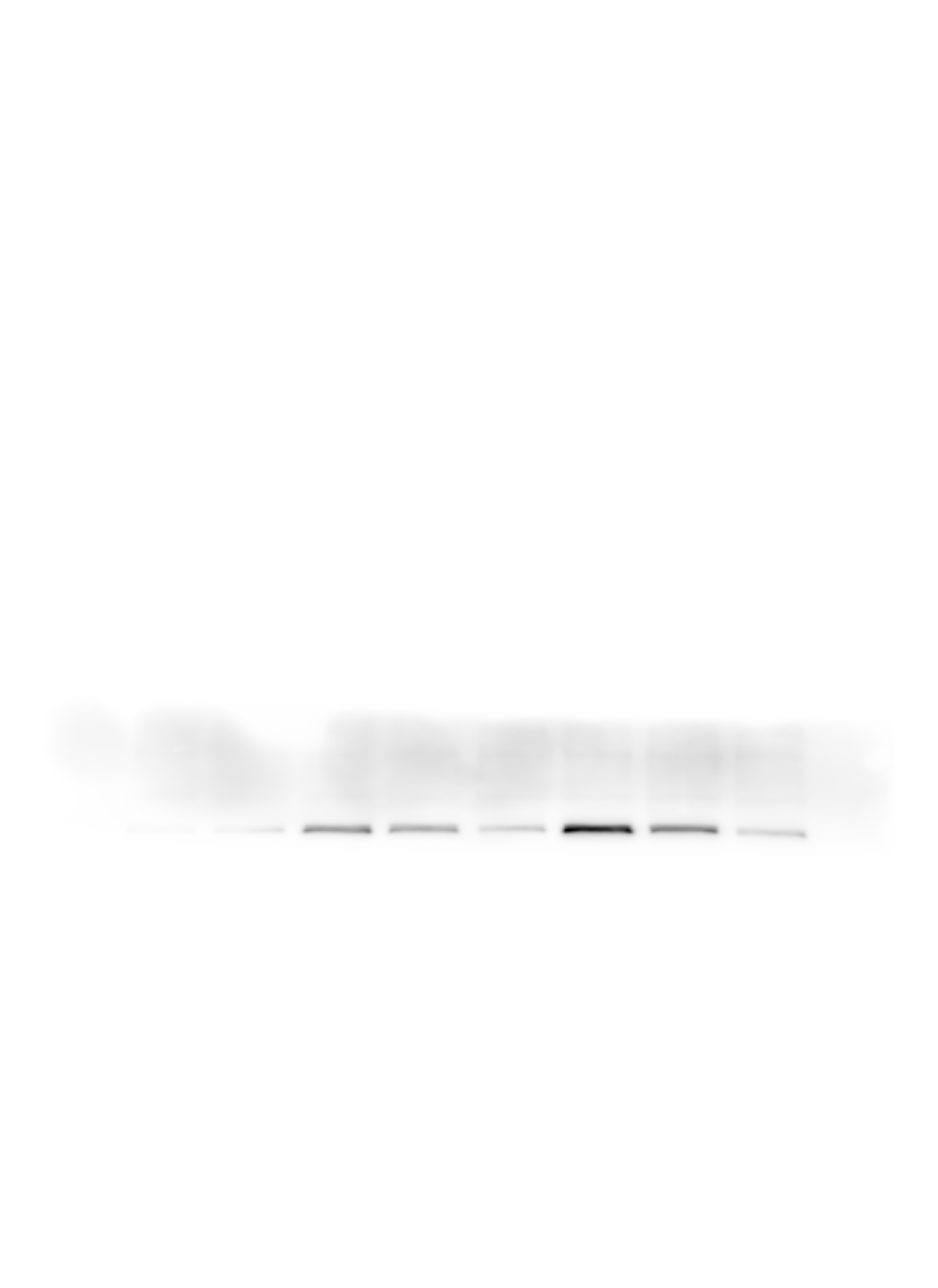

Supplement: Figure 5—figure supplement 1—source data 1. [file elife-105821-fig5-figsupp1-data1.zip › Figure 5-figure supplement 2-source data 1/Original files for western blot analysis displayed in Figure 5-figure supplement 2D/Vps35 20240606_120926_Ch/Vps35 20240606_120926_Ch_Chemi.tif]

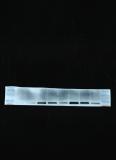

Supplement: Figure 5—figure supplement 1—source data 1. [file elife-105821-fig5-figsupp1-data1.zip › Figure 5-figure supplement 2-source data 1/Original files for western blot analysis displayed in Figure 5-figure supplement 2D/Vps35 20240606_120926_Ch/Vps35 20240606_120926_Ch_Thumb.jpg]

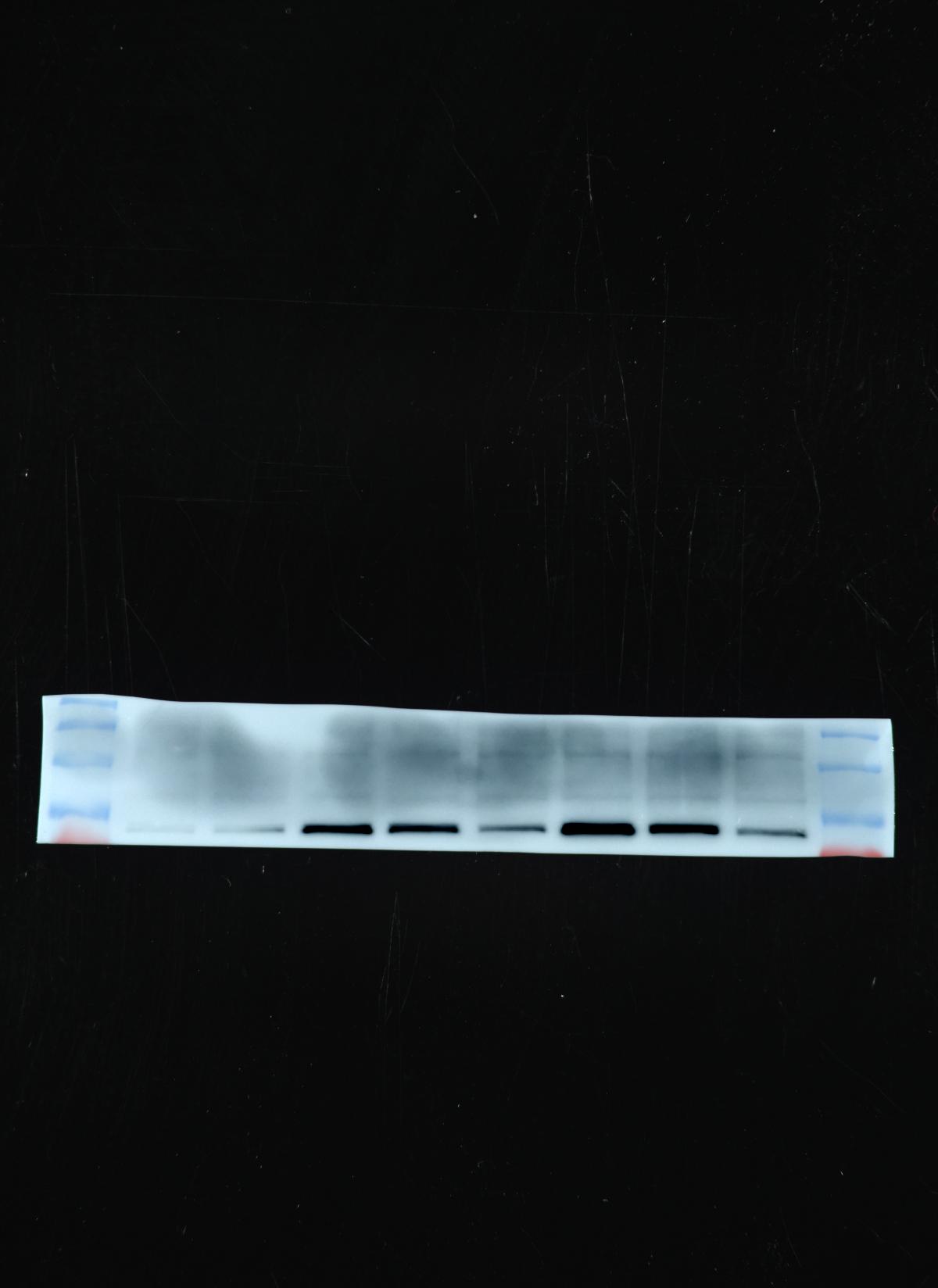

Supplement: Figure 5—figure supplement 1—source data 1. [file elife-105821-fig5-figsupp1-data1.zip › Figure 5-figure supplement 2-source data 1/Original files for western blot analysis displayed in Figure 5-figure supplement 2D/Vps35 20240606_120926_Ch/Vps35 20240606_120926_Ch_Chemi+Marker.jpg]

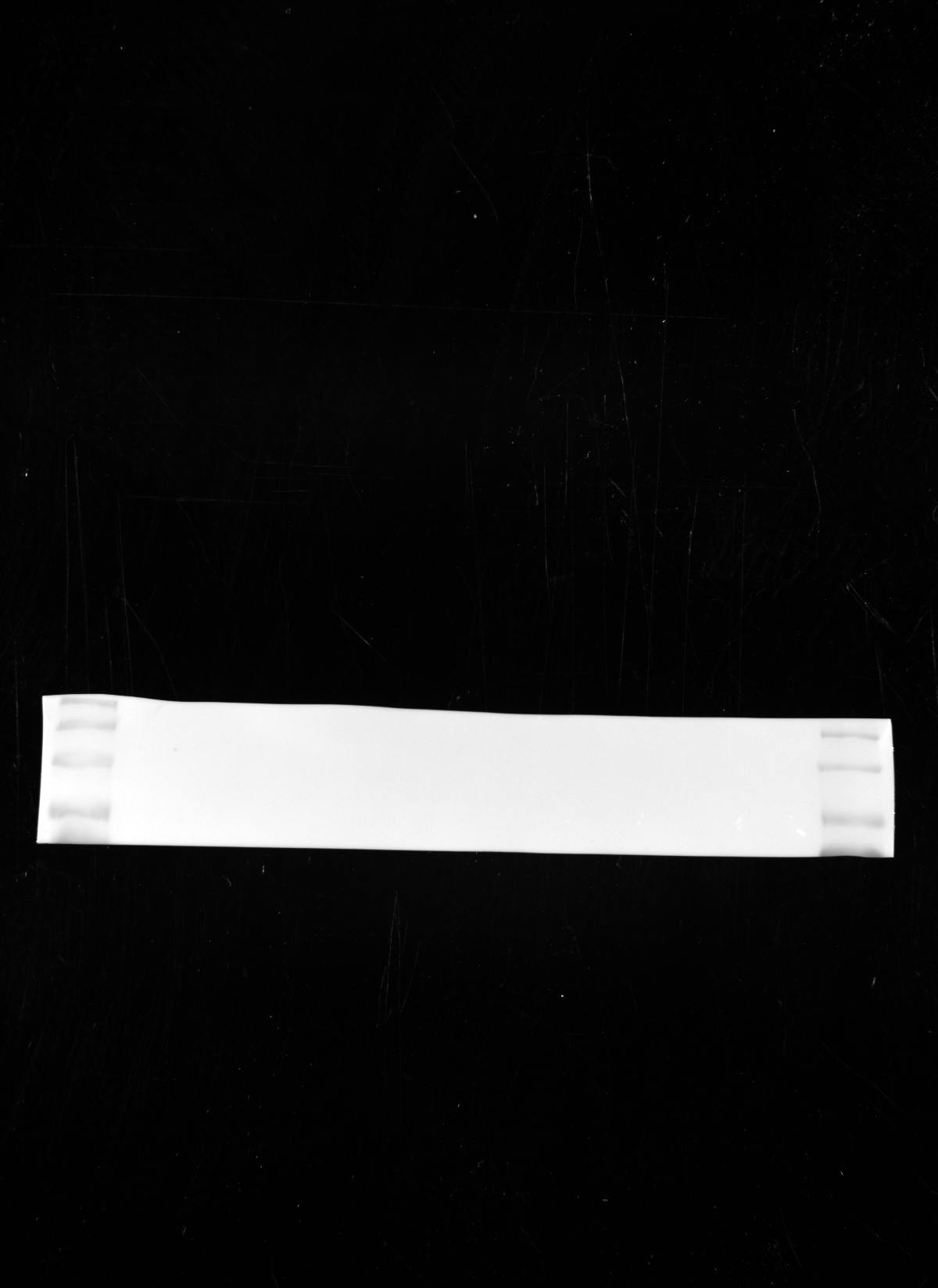

Supplement: Figure 5—figure supplement 1—source data 1. [file elife-105821-fig5-figsupp1-data1.zip › Figure 5-figure supplement 2-source data 1/Original files for western blot analysis displayed in Figure 5-figure supplement 2D/Vps35 20240606_120926_Ch/Vps35 20240606_120926_Ch-Marker.tif]

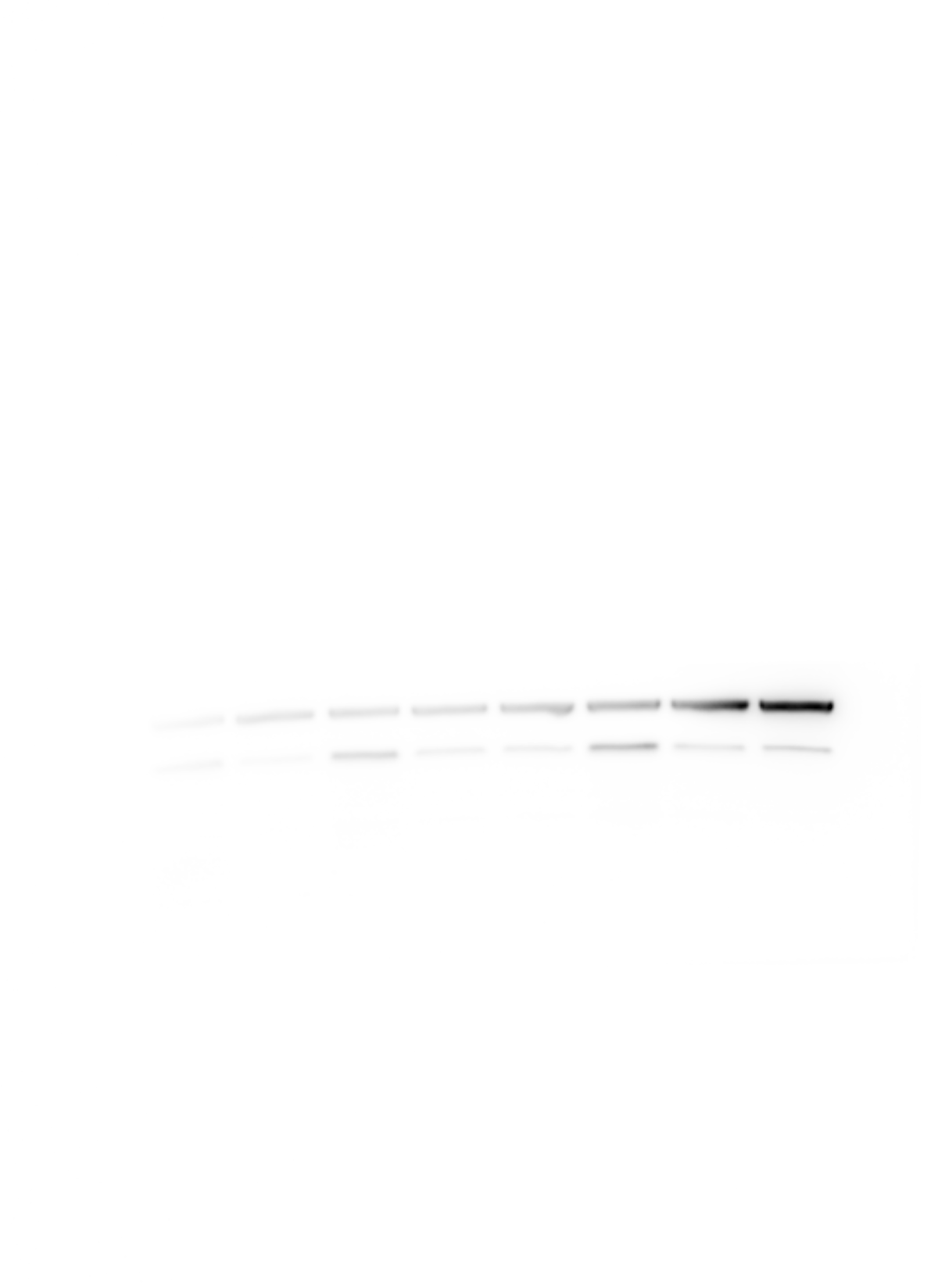

Supplement: Figure 5—figure supplement 1—source data 1. [file elife-105821-fig5-figsupp1-data1.zip › Figure 5-figure supplement 2-source data 1/Original files for western blot analysis displayed in Figure 5-figure supplement 2D/Tubulin Vps26 20240606_121347_Ch/Tubulin Vps26 20240606_121347_Ch_Chemi.tif]

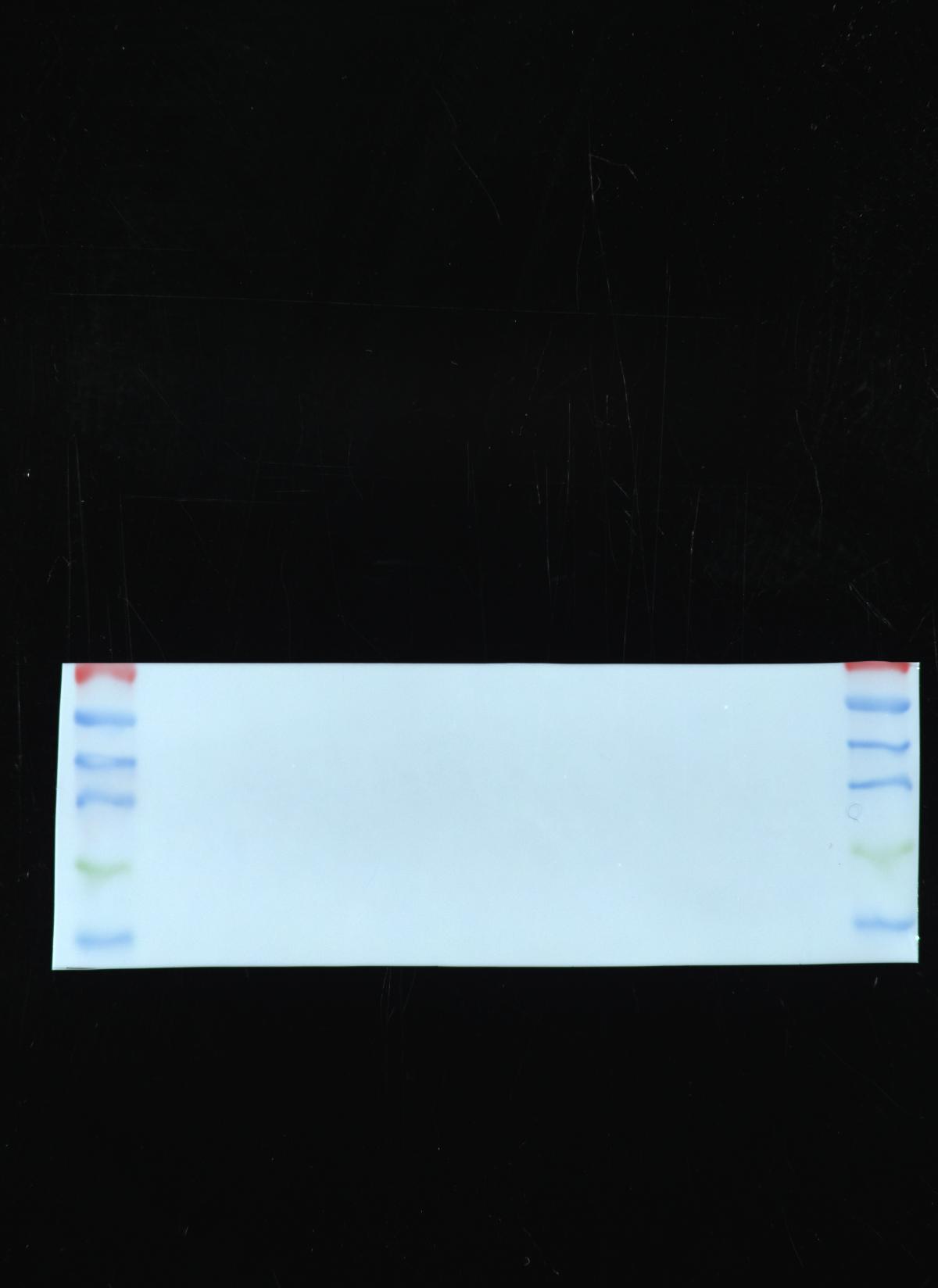

Supplement: Figure 5—figure supplement 1—source data 1. [file elife-105821-fig5-figsupp1-data1.zip › Figure 5-figure supplement 2-source data 1/Original files for western blot analysis displayed in Figure 5-figure supplement 2D/Tubulin Vps26 20240606_121347_Ch/Tubulin Vps26 20240606_121347_Ch-Marker.jpg]

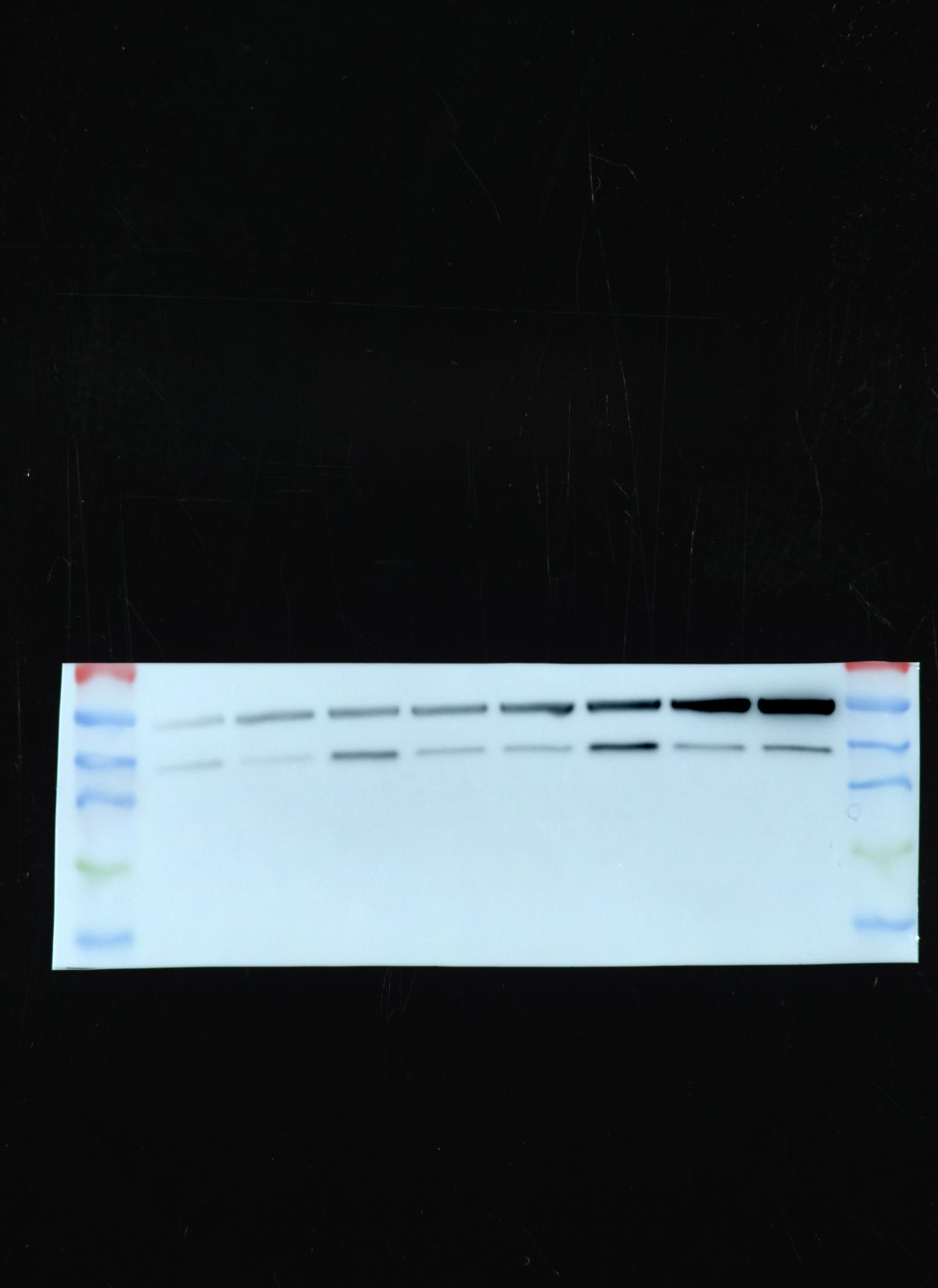

Supplement: Figure 5—figure supplement 1—source data 1. [file elife-105821-fig5-figsupp1-data1.zip › Figure 5-figure supplement 2-source data 1/Original files for western blot analysis displayed in Figure 5-figure supplement 2D/Tubulin Vps26 20240606_121347_Ch/Tubulin Vps26 20240606_121347_Ch_Chemi+Marker.jpg]

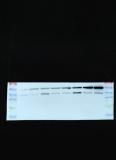

Supplement: Figure 5—figure supplement 1—source data 1. [file elife-105821-fig5-figsupp1-data1.zip › Figure 5-figure supplement 2-source data 1/Original files for western blot analysis displayed in Figure 5-figure supplement 2D/Tubulin Vps26 20240606_121347_Ch/Tubulin Vps26 20240606_121347_Ch_Thumb.jpg]

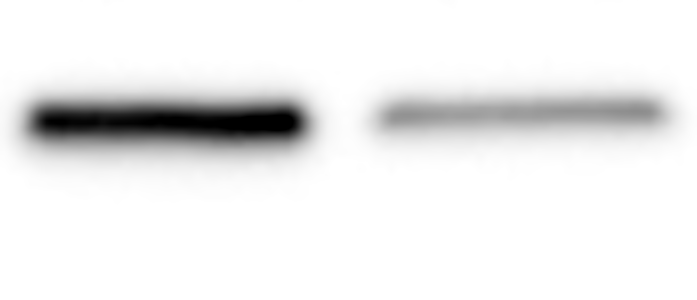

Supplement: Figure 5—figure supplement 1—source data 1. [file elife-105821-fig5-figsupp1-data1.zip › Figure 5-figure supplement 2-source data 1/Original files for western blot analysis displayed in Figure 5-figure supplement 2D/Tubulin Vps26 20240606_121347_Ch/Tubulin Vps26 20240606_121347_Ch_Chemi-2.tif]

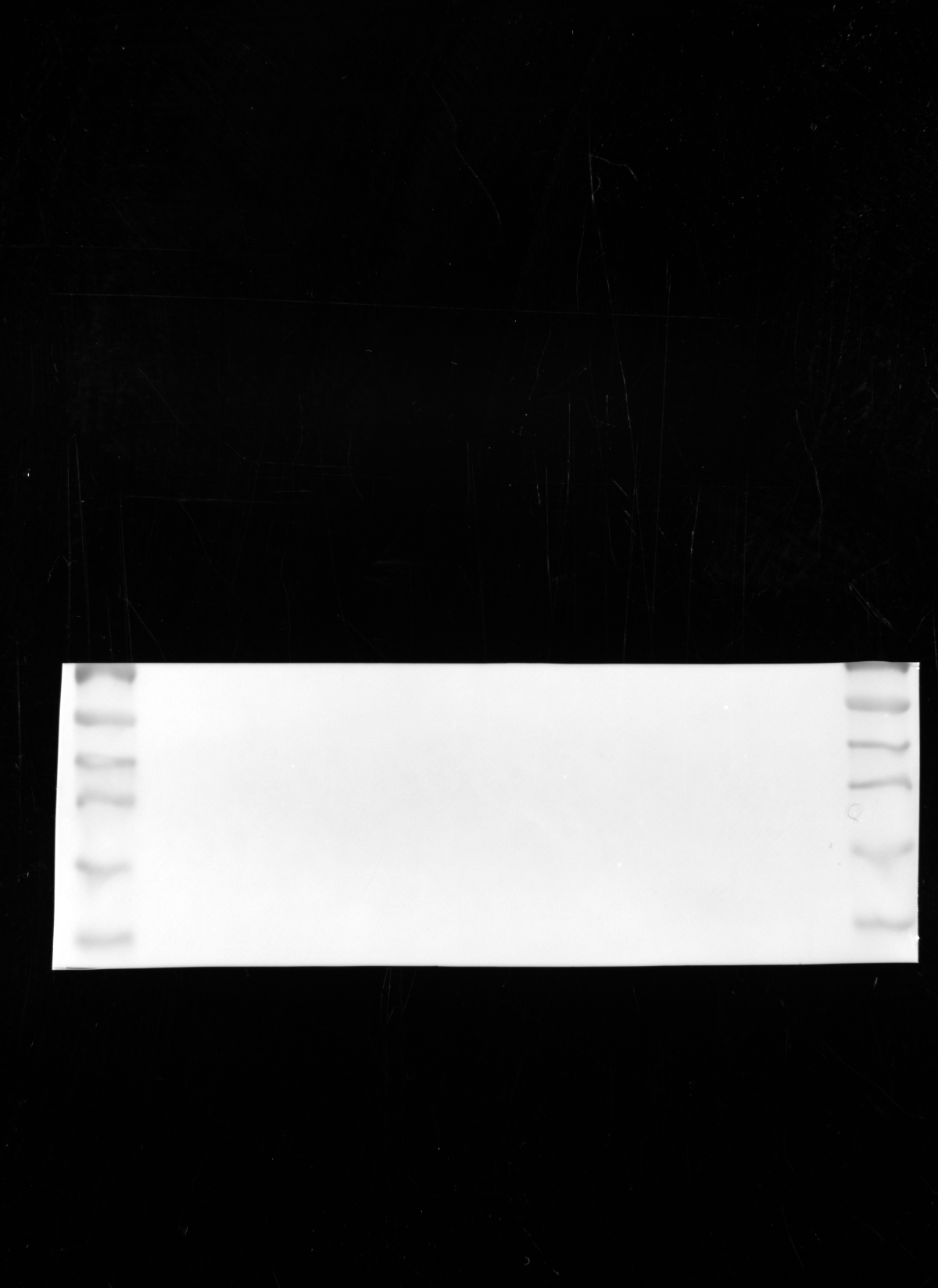

Supplement: Figure 5—figure supplement 1—source data 1. [file elife-105821-fig5-figsupp1-data1.zip › Figure 5-figure supplement 2-source data 1/Original files for western blot analysis displayed in Figure 5-figure supplement 2D/Tubulin Vps26 20240606_121347_Ch/Tubulin Vps26 20240606_121347_Ch-Marker.tif]

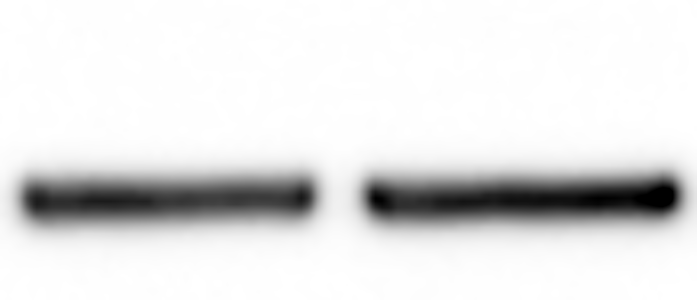

Supplement: Figure 5—figure supplement 1—source data 1. [file elife-105821-fig5-figsupp1-data1.zip › Figure 5-figure supplement 2-source data 1/Original files for western blot analysis displayed in Figure 5-figure supplement 2D/Tubulin Vps26 20240606_121347_Ch/Tubulin Vps26 20240606_121347_Ch_Chemi-1.tif]

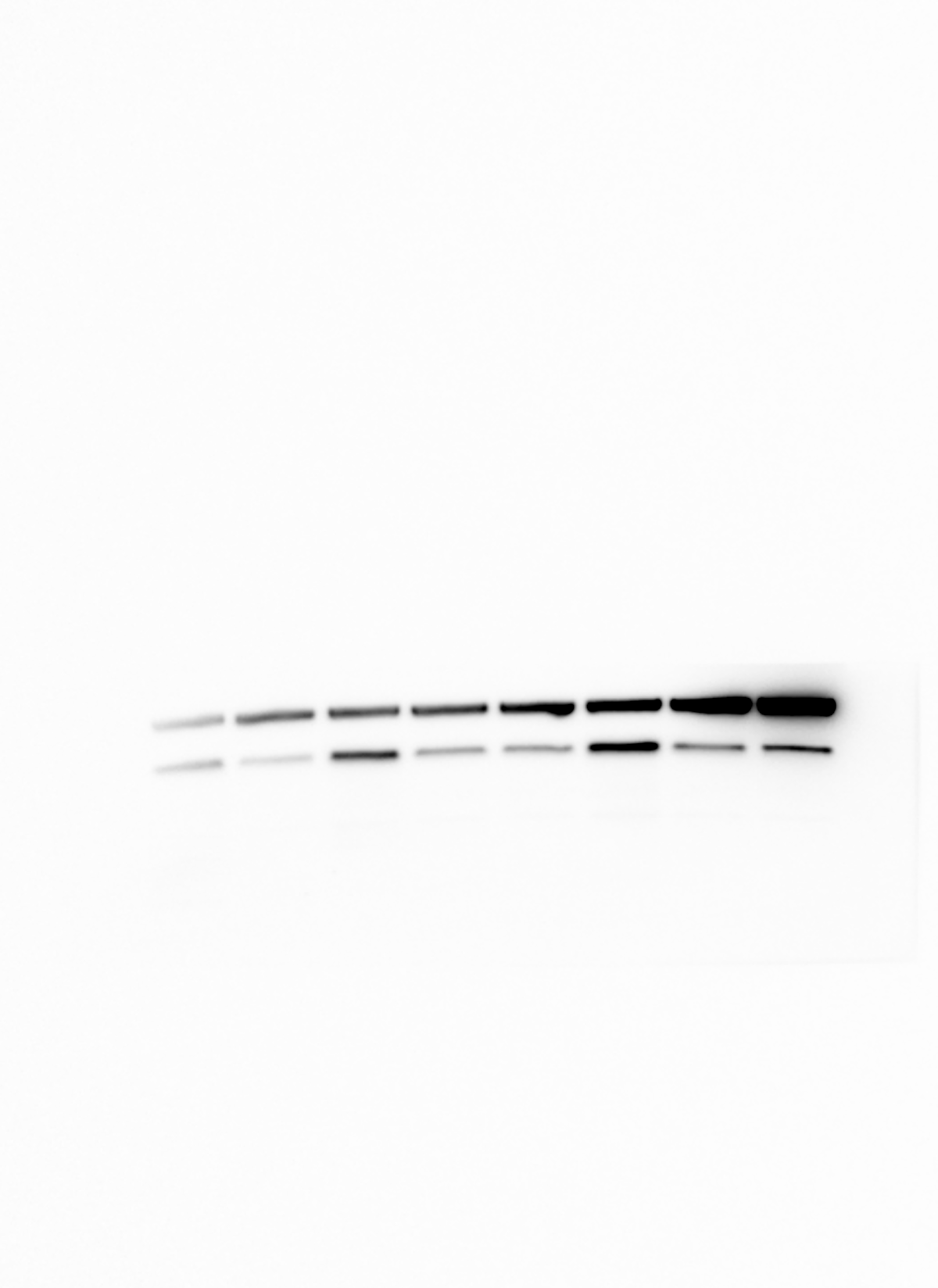

Supplement: Figure 5—figure supplement 1—source data 1. [file elife-105821-fig5-figsupp1-data1.zip › Figure 5-figure supplement 2-source data 1/Original files for western blot analysis displayed in Figure 5-figure supplement 2D/Tubulin Vps26 20240606_121347_Ch/Tubulin Vps26 20240606_121347_Ch_Chemi-3.tif]

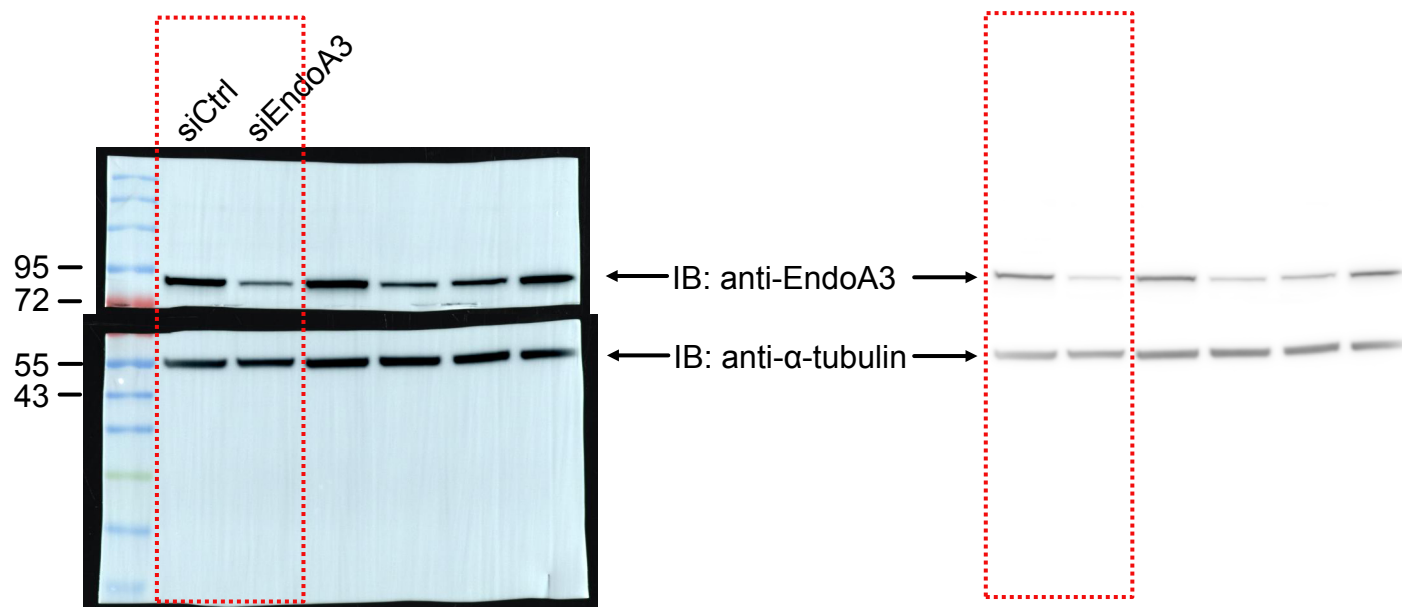

**Blots in the red frame were used in the manuscript.**

Supplement: Figure 5—figure supplement 1—source data 2. [file elife-105821-fig5-figsupp1-data2.zip › Figure 5-figure supplement 2-source data 2/PDF file containing original western blots for Figure 5-figure supplement 2C.pdf]

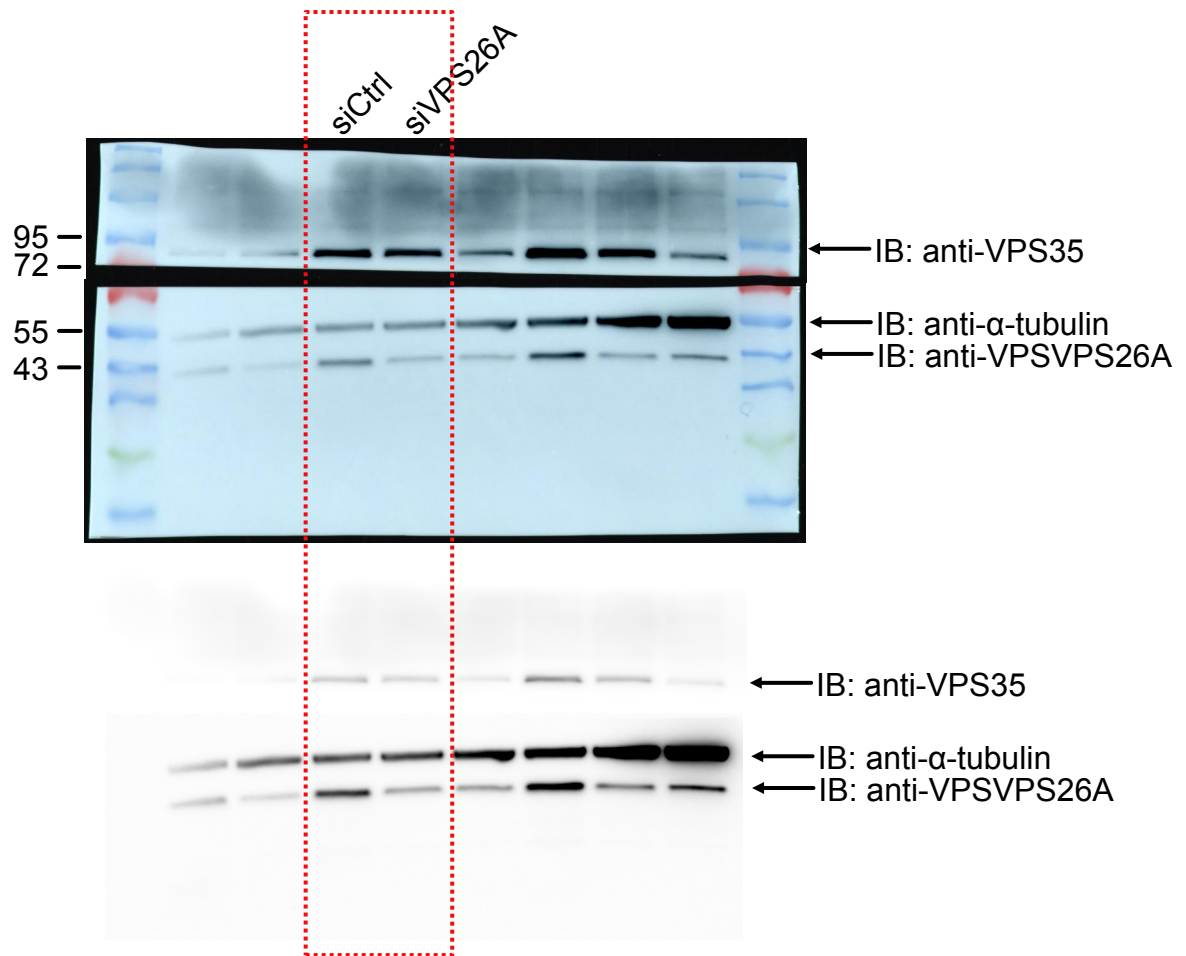

**Blots in the red frame were used in the manuscript.**

Supplement: Figure 5—figure supplement 1—source data 2. [file elife-105821-fig5-figsupp1-data2.zip › Figure 5-figure supplement 2-source data 2/PDF file containing original western blots for Figure 5-figure supplement 2D.pdf]
