# Supplementary material for: Clathrin-independent endocytosis and retrograde transport in cancer cells tune immune synapse organization and CD8 T cell response
Source: eLife. 2026 Apr 22;14:RP105821. doi: 10.7554/eLife.105821 (PMC13102394; doi:10.7554/eLife.105821)
Supplement: Figure 3—figure supplement 1—source data 2. [file elife-105821-fig3-figsupp1-data2.zip › Figure 3-figure supplement 1-source data 2/PDF file containing original western blots for Figure 3-figure supplement 1L.pdf]

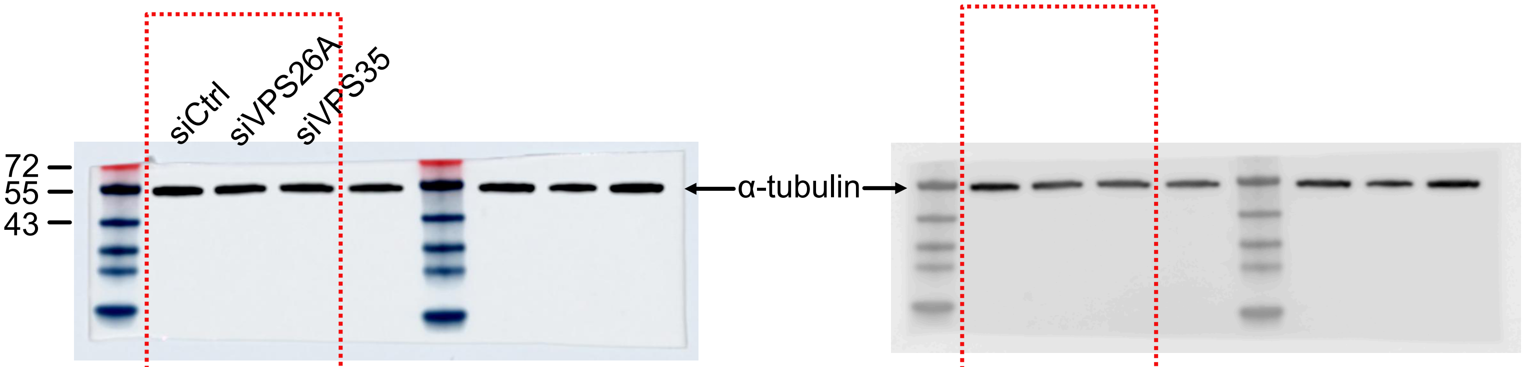

This is the same blot as for VPS26A (below) but detected with fluorescence for tubulin, not HRP.

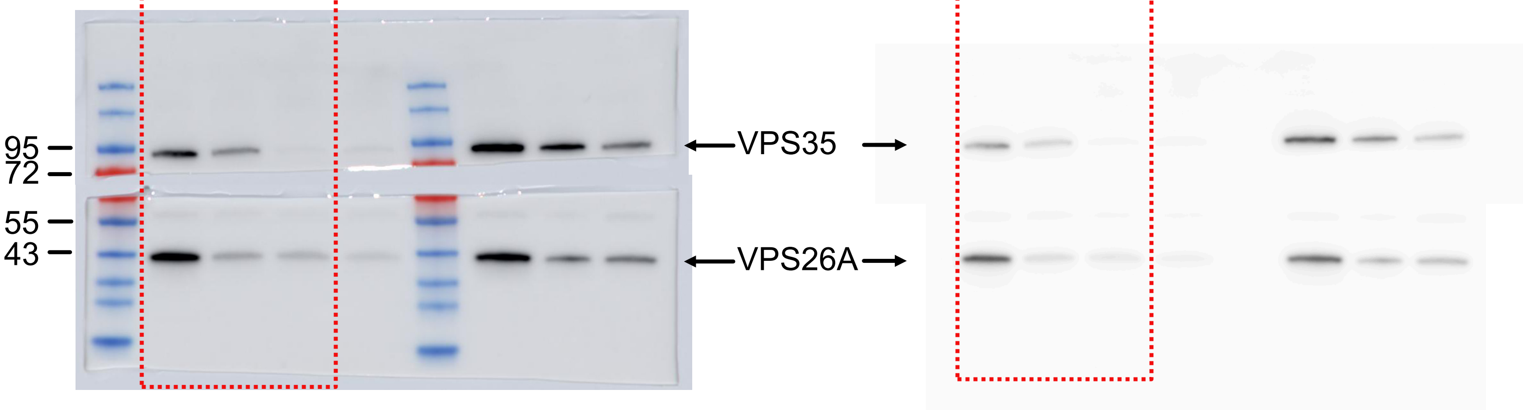

Blots in the red frame were used in the manuscript.
